# Photomediated reductive coupling of nitroarenes with aldehydes for amide synthesis†

**DOI:** 10.1039/d2sc03047k

**Published:** 2022-08-03

**Authors:** Qingyao Li, Peng Dai, Haidi Tang, Muliang Zhang, Jie Wu

**Affiliations:** Department of Chemistry, National University of Singapore 3 Science Drive 3 117543 Singapore muliang0206@foxmail.com chmjie@nus.edu.sg

## Abstract

In view of the widespread significance of amide functional groups in organic synthesis and pharmaceutical studies, an efficient and practical synthetic protocol that avoids the use of stoichiometric activating reagents or metallic reductants is highly desirable. A straight-forward pathway to access amides from abundant chemical feedstock would offer a strategic advantage in the synthesis of complex amides. We herein disclose a direct reductive amidation reaction using readily available aldehydes and nitroarenes enabled by photo-mediated hydrogen atom transfer catalysis. It avoids the use of metallic reductants and production of toxic chemical waste. While aldehydes represent a classic class of electrophilic synthons, the corresponding nucleophilic acyl radicals could be directly accessed by photo hydrogen atom transfer catalysis, enabling polarity inversion. Our method provides an orthogonal strategy to conventional amide couplings, tolerating nucleophilic substituents such as free alcohols and sensitive functional groups to amines such as carbonyl or formyl groups. The synthetic utilization of this reductive amidation is demonstrated by the late-stage modification of complex biologically active molecules and direct access of drug molecules leflunomide and lidocaine.

## Abstract 2

In view of the widespread significance of amide functional groups in organic synthesis and pharmaceutical studies, an efficient and practical synthetic protocol that avoids the use of stoichiometric activating reagents or metallic reductants is highly desirable.

## Graphical abstract

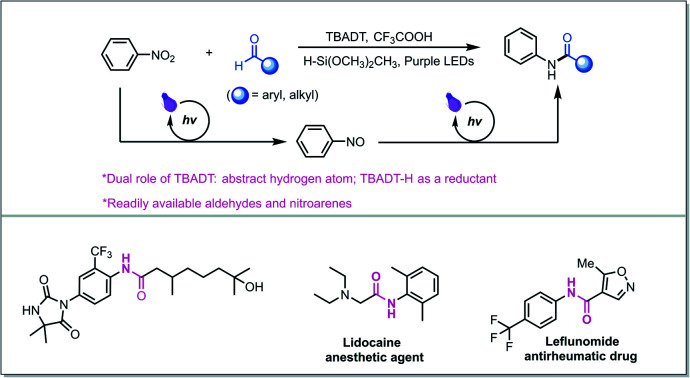

## Introduction

**Fig. 1:**
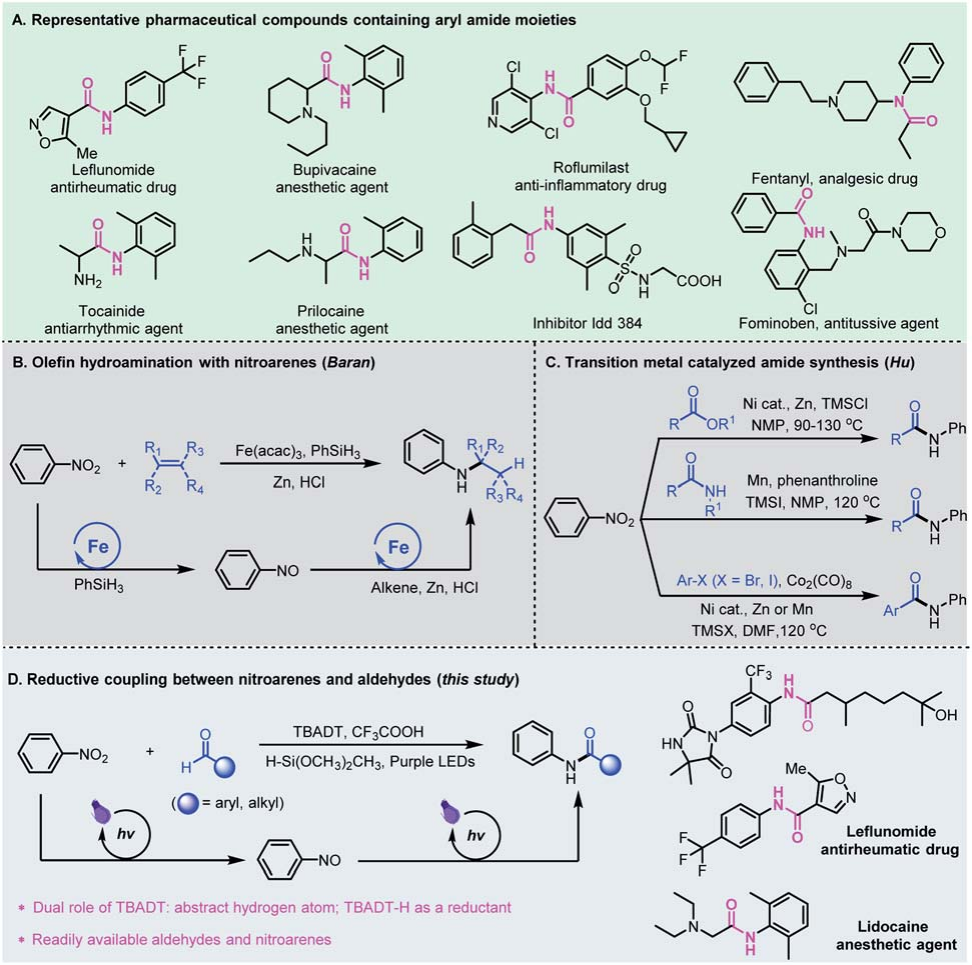
Representative aryl amides and their proposed synthesis from aldehydes and nitroarenes.

The amide functional group is ubiquitous in nature and the significance of amides in chemical biology has been well recognized and widely investigated in the past century.^1^ The physiological properties of amides and their unique ability to obstruct natural neurotransmission pathways have made them prevalent pharmaceutical agents.^2^ About 25% of natural and synthetic medicines contain amide linkages, which can be found in widely used drugs such as leflunomide, bupivacaine, roflumilast, fentanyl, and fominoben (Fig. 1A).^3^ Conventionally, the synthesis of amides is generally based on activated carboxylic acid derivatives such as acid chlorides and anhydrides or rearrangement reactions which require tedious extra prefunctionalization steps, or from carboxylic acids assisted by stoichiometric amounts of activating agents that usually lead to toxic chemical waste byproducts.^4^ These processes are also typically water or air sensitive, and require conditions which are incompatible with sensitive functional groups, limiting their use in organic synthesis and chemical biology studies.

The need for amide-containing molecules has led to the development of straightforward pathways to access complex amides from readily available feedstock chemicals. Recent attractive protocols include P^III^/P^V^-catalyzed three-component condensation of amines, carboxylic acids and pyridine *N*-oxides,^5^ photocatalyzed oxime isomerization and subsequent Beckmann rearrangement,^6^ and re-routing of unactivated *N*,*N*-dialkylamide bonds through reactive acyl iodide intermediates.^7^ Chang and co-workers developed a photoinduced amidation of arylboronic acids using 1,4,2-dioxazol-5-ones as an amide coupling partner.^8^ Later, preparation of functionalized amides using dicarbamoylzincs was also reported by Knochel.^9^ Alternatively, the cross coupling for amide synthesis has been realized from alkynes,^10^ alcohols,^11^ or α-keto acids^12^ and aldehydes^13^ using amines as the starting materials. Compared to amines, nitroarenes as the nitrogen source are often more accessible, less expensive and more stable.^14^ In 2015, Baran *et al.* reported an efficient hydroamination protocol which synthesizes aryl amines from nitroarenes and alkenes with an iron salt as a catalyst, PhSiH_3_ as a hydrogen source, and zinc metal as the reductant (Fig. 1B).^15^ Later, Zhu and co-workers developed nickel-catalyzed remote C(sp^3^)–H amination of alkenes with nitroarenes for the synthesis of amines.^16^ In these catalytic cycles, the transition metal-hydrides formed *in situ* play an important role, and promote the initial coordination of the Lewis acidic metal center with unsaturated bonds, followed by reductive coupling with nitrosoarenes. Recently, Hu *et al.* developed a series of novel amidation reactions of esters,^17^ halides^18^ or amides^19^ with nitroarenes as the nitrogen source (Fig. 1C). However, these reductive amidation reactions usually require stoichiometric amounts of metallic reductants such as Zn or Mn, and proceed only at high temperatures.

Photomediated hydrogen atom transfer (HAT) catalysis is a powerful strategy for aldehydic C–H functionalization.^20^ We have developed direct HAT photocatalysis with aldehydes to access the corresponding acyl radicals, which could participate in a series of useful transformations such as asymmetric 1,4-addition with Michael acceptors,^21^ radical Smiles rearrangements,^22^ and nucleophilic substitution reactions with sulfone reagents.^23^ Among the reported photo-HAT catalysts, tetrabutylammonium decatungstate (TBADT) catalytically cleaves the aldehydic C–H bond under near-UV light irradiation, giving rise to acyl radicals and TBADT–H species.^24^ We envisioned that the TBADT–H species generated *in situ* could be regarded as an analogue of transition metal-hydrides,^25^ which could trigger the reduction of nitroarenes, realizing reductive coupling of nitroarenes with aldehydes (Fig. 1D). Specifically, tetrabutylammonium decatungstate abstracts the aldehydic C–H bond to deliver nucleophilic acyl radicals by direct photo hydrogen atom transfer catalysis, enabling polarity inversion. The nucleophilic acyl radicals generated could further undergo a radical addition–elimination process for nitroarene reduction. Such a reductive process is independent of the reductive potential of nitroarenes, thus broadening the substrate scope of nitroarenes without redox limitation. The TBADT plays critical and multifaceted roles in this reductive catalytic process, not only activating the aldehydic C–H bond to deliver acyl radicals, but also using the transferred hydrogen to facilitate the overall reductive coupling. Aldehydes and nitroarenes are both widely used, inexpensive and readily available feedstock chemicals. The reductive coupling of aldehydes with nitroarenes represents an attractive and sustainable strategy for the direct generation of bioactive aryl amide compounds. Even though direct access to amides from aromatic aldehydes and nitroarenes has been reported,^26^ the pathways relied heavily on ionic reactions using much excess metal reductants with very limited substrate scope and no practical applications.

## Results and discussion

We began our investigation with nitrobenzene (1a) and cyclopropanecarbaldehyde (2a) as the model reactants. As shown in Table 1, with 2 mol% TBADT, 2.5 equiv. of dimethoxy-(methyl)silane, 30 mol% trifluoroacetic acid and CH_3_CN as the solvent, the desired amide product (3a) was obtained in 78% yield under 390 nm light irradiation (entry 1). The use of other silanes such as TMS_3_Si–H, PhSiH_3_, and Et_3_SiH led to inferior reaction yields (entry 2, also see ESI Table S2†). When eosin Y was used as the HAT photocatalyst,^27^ the reductive amidation did not occur, highlighting the unique role of the TBADT–H generated *in situ* (Table 1, entry 3). With TFA absent or replaced by another acid such as acetic acid a substantial decrease of the yield was noted (entries 4 and 5). We believe that the addition of TFA could accelerate the photocatalytic process and increase the reactivity.^28^ A higher reaction temperature resulted in more over-reduced amine byproducts, and a negative influence on the reaction outcome (entry 6). Control experiments demonstrated that both the photocatalyst and light irradiation are essential for this reductive amidation (entries 7 and 8).

**Table 1:** Reaction optimization of reductive amidation

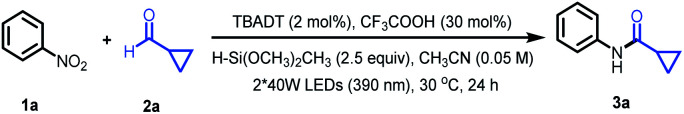		
Entry	Variation from standard conditions^a^	Yield^b^ (%)
1	None	81 (78)^c^
2	TMS_3_Si–H instead of CH_3_(OCH_3_)_2_Si–H	60
3	Eosin Y instead of TBADT	<2
4	Without CF_3_COOH	40
5	CH_3_COOH instead of CF_3_COOH	41
6	60 °C instead of 30 °C	58
7	Without TBADT	6
8	Without light irradiation	0

a Standard conditions: 1a (0.1 mmol), 2a (3 equiv.), TBADT (2 mol%), CF_3_COOH (30 mol%), H–Si(OCH_3_)_2_CH_3_ (2.5 equiv.), CH_3_CN (2 mL), 2 × 40 W LEDs (390 nm), 30 °C, 24 h.

b Yields are based on the analysis of the ^1^H NMR spectra of the crude product mixture using CH_2_Br_2_ as an internal standard.

c Isolated yields.

**Fig. 2:**
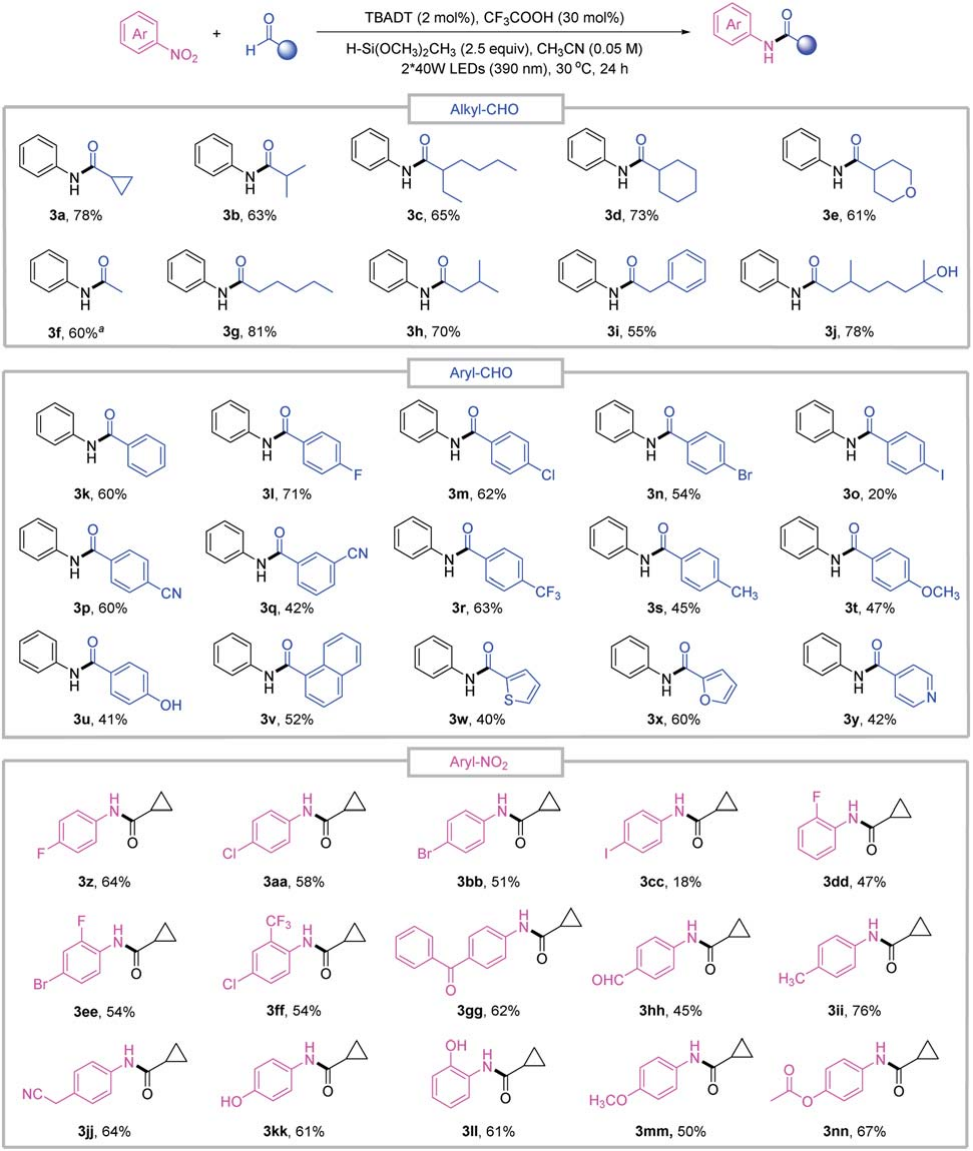
Substrate scope of reductive amidation. Standard conditions: nitroarene 1 (0.1 mmol), aldehyde 2 (3 equiv.), TBADT (2 mol%), CF_3_COOH (30 mol%), H–Si(OCH_3_)_2_CH_3_ (2.5 equiv.), CH_3_CN (2 mL), purple LEDs (390 nm), 30 °C, 24 h. ^*a*^The amount of aldehyde was 20 equiv.

With the optimized conditions in hand, we sought to evaluate the scope of this reductive amidation. A variety of amides were effectively synthesized from the corresponding nitroarenes and aldehydes as shown in Fig. 2. Secondary and primary alkyl aldehydes proved to be competent starting materials, offering the desired amides (3a–3j) in moderate to good yields. However, tertiary alkyl aldehydes failed to produce the desired product, and this was complicated by the facile decarbonylation of the tertiary acyl radicals generated *in situ*. A series of aromatic aldehydes and nitroarene partners participated smoothly in the reductive amidation reaction, tolerating a wide range of functional groups, such as halo (3l–3o, 3z–3ff), cyano (3p–3q, 3jj), methoxy (3t, 3mm), trifluoromethyl (3r) and ester (3nn) derivatives. Heteroaromatic aldehydes such as furfural (3x), pyridine (3y) and thiophene (3w) delivered the amide products in moderate to good yields, and reduction-sensitive functional groups such as aldehyde (3hh) and ketone (3gg) and nucleophilic groups such as the hydroxyl group (3j, 3u, 3kk, 3ll) were well tolerated under the reaction conditions. These functionalities may cause compatibility issues when preparing the required aryl amines or activated acid derivatives in conventional amide coupling methods.

**Fig. 3:**
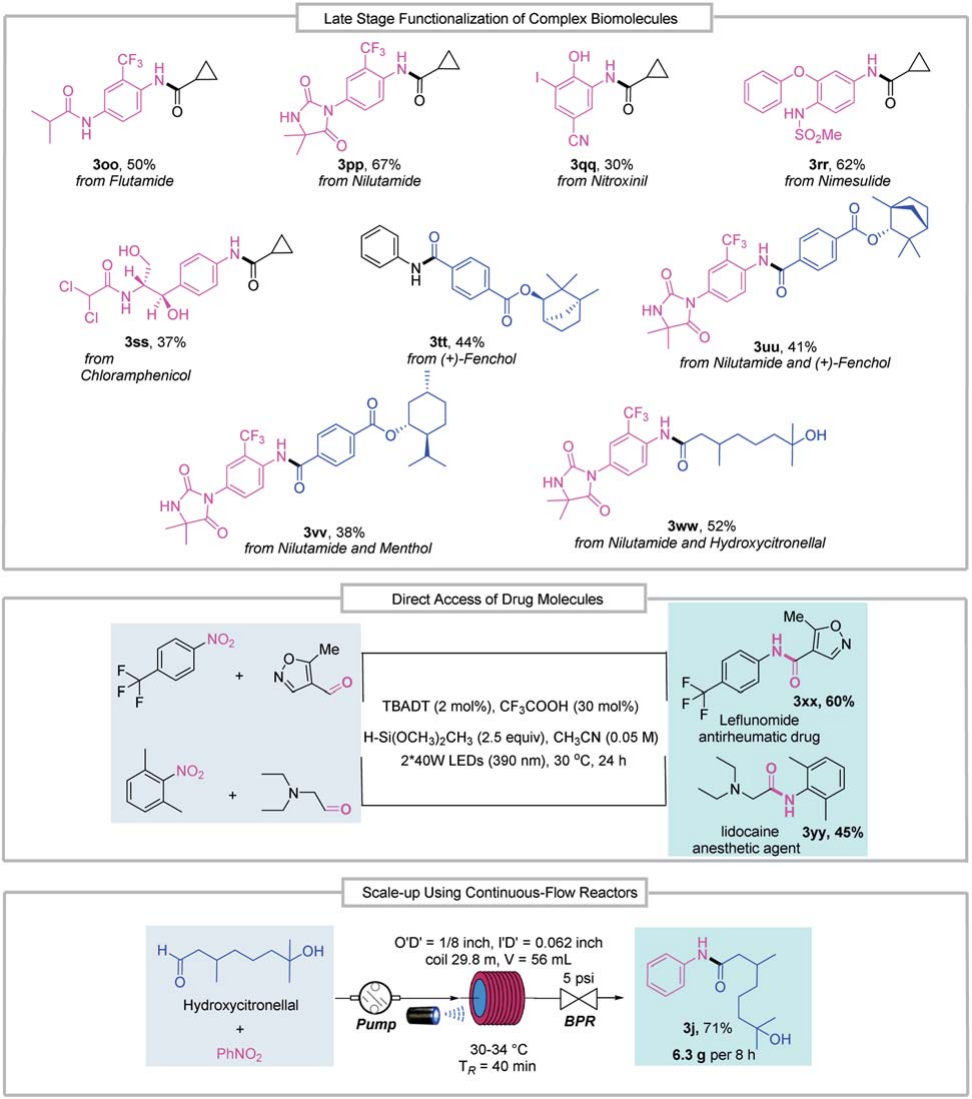
Late-stage functionalization of complex biomolecules, direct access to drug molecules and reaction scale-up in continuous-flow reactors. BPR = back pressure regulator.

The good functional group tolerance enables the synthesis of complex amides through late-stage functionalization of biologically active molecules (Fig. 3). This amide bond formation method can be used in the synthesis of pharmaceutical nitroarene compounds, such as flutamide (3oo), nilutamide (3pp), nitroxinil (3qq), nimesulide (3rr), and chloramphenicol (3ss), which are formed in moderate to good yields. Aldehydes derived from natural products such as (+)-fenchol (3tt and 3uu) and menthol (3vv) participated in the reductive coupling smoothly. Notably, the direct coupling of the nitroarene drug molecule nilutamide and the commercially available odiferous hydroxycitronellal delivered the amide product (3ww) in 52% yield. Next, by taking advantage of aldehyde and nitroarene feedstock, leflunomide (3xx), an antirheumatic drug, and lidocaine (3yy), an anesthetic agent, were directly accessed using our protocol. These examples suggest the potential wide utility of this method in medicinal chemistry. Finally, the reductive amidation reaction was transferred to an operationally simple continuous-flow reactor to achieve the amide product (3j) with 6.3 g per 8 h from readily available nitrobenzene and hydroxycitronellal, indicating the potential for large-scale synthesis.

**Fig. 4:**
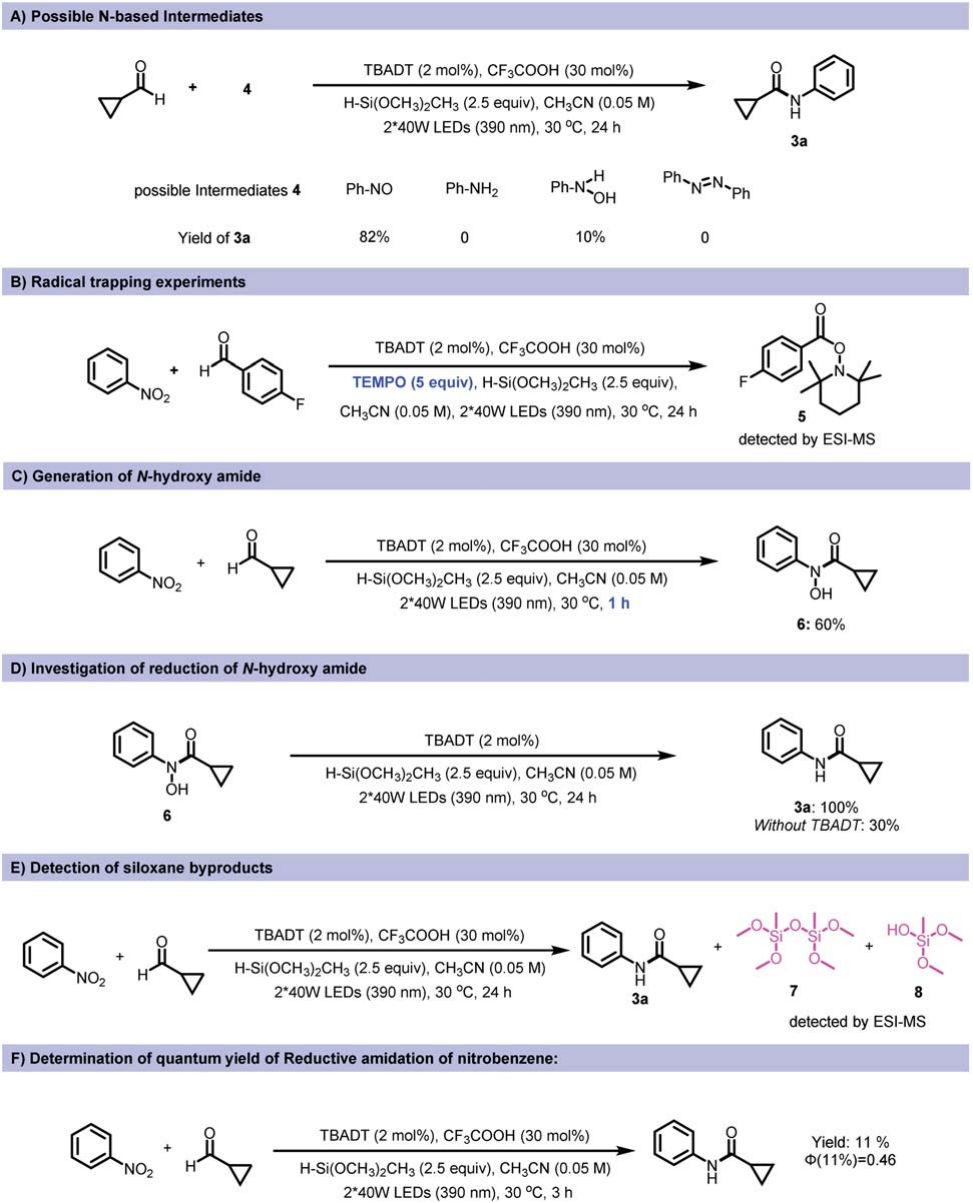
Control experiments to elucidate the mechanisms.

Various control experiments were performed to investigate the mechanism of this transformation. To probe the possible nitrogen-containing intermediates involved in this reductive coupling, several nitrogen-based compounds were tested as the starting materials under the standard reaction conditions. Among the reagents tested, nitrosobenzene afforded the desired amide product in 82% yield (Fig. 4A). However, anilines, azobenzene and phenylhydroxyamine gave a significantly lower yield (10%) or no product. This indicates that nitrosobenzene is one of the active intermediates during the reaction process. The addition of TEMPO (2,2,6,6-tetramethyl-1-piperidi-nyloxy) inhibited the transformation, and the TEMPO-acyl radical adduct was detected by ESI-MS, supporting the presence of acyl radicals during the reaction process (Fig. 4B). Moreover, *N*-hydroxy amide (6) could be obtained if the transformation was quenched after 1 h reaction time (Fig. 4C). Subjecting the *N*-hydroxy amide (6) to the standard reaction conditions produced the amide quantitatively (Fig. 4D), and this suggested that an *N*-hydroxy amide was also involved in the reaction process. After reaction, siloxane or silanol derived from the silane was detected (Fig. 4E). The quantum yield of a model reaction was determined to be 0.46 (Fig. 4F).

## Mechanistic rationale

**Fig. 5:**
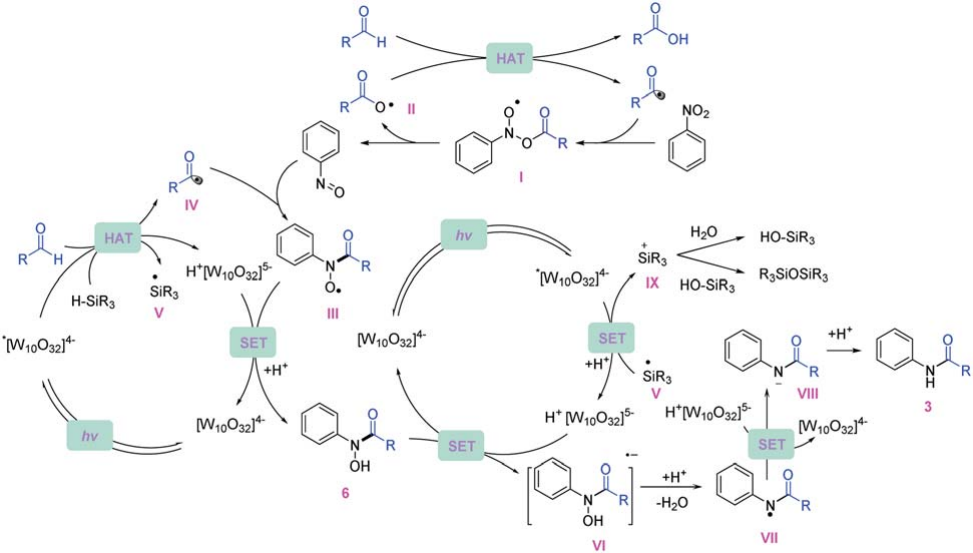
Proposed plausible mechanisms of the reductive amidation reaction.

Based on the experimental data, a plausible mechanistic pathway of the reductive coupling is proposed in Fig. 5. Under 390 nm light irradiation, the excited photocatalyst TBADT abstracts a hydrogen atom from the aldehydes or the silanes to generate the corresponding acyl radicals, silyl radicals and reductive intermediate H^+^[W_10_O_32_]^5−^ (*E*_red_ = −1.27 V *vs.* Ag/Ag^+^ in MeCN).^29^ The acyl radicals could directly undergo a radical addition on nitroarenes to give the intermediates (I), followed by decomposition to offer nitrosoarenes and carboxyl radicals (II).^30^ This carboxyl radical species (II) is capable of abstracting another hydrogen atom from aldehydes to offer acyl radicals.^20^ Next, the nitrosoarenes will react with the acyl radicals to form radical species (III), followed by a SET process with H^+^[W_10_O_32_]^5−^ to give rise to the *N*-hydroxy amide.^31^ The highly reducing H^+^[W_10_O_32_]^5−^ can reduce the *N*-hydroxy amide (*E*_red_ = −0.65 V *vs.* Ag/Ag^+^ in MeCN, ESI Fig. S12†) to the corresponding radical anion intermediate (VI), which will release OH^−^ to generate an amidyl radical (VII), followed by another SET with H^+^[W_10_O_32_]^5−^ and protonation to accomplish the final amide product. The *in situ* generated silyl radical may undergo a single electron oxidation by the excited *[W_10_O_32_]^4−^ (*E* ∼ +2.5 V *vs.* SCE in MeCN)^32^ to furnish a silyl cation intermediate,^33^ which is captured by nucleophiles such as H_2_O and silanols to give silanols or siloxane after deprotonation (for a more detailed description of the plausible mechanism, see ESI Fig. S14†).

## Conclusions

We have developed an efficient TBADT-based photocatalytic system for a reductive amidation of aldehydes and nitroarenes. A broad range of readily available aldehydes and nitroarenes are competent coupling partners with high functional group tolerance, and furnish a broad scope of functionally diverse aryl amides. This reductive amidation method is an improvement on the conventional amide bond-forming pathways, which require acid activation and pre-reduction of nitroarenes to amines. Moreover, the nitro group exhibits orthogonal reactivity to the amine group, tolerating nucleophilic substituents such as free alcohols. Functional groups sensitive to amines such as carbonyl or formyl groups are also well-tolerated when using nitroarenes as the starting materials, which are difficult to directly access by the conventional amide coupling strategy. This method is amenable to late-stage functionalization of complex molecules and provides direct access to pharmaceuticals, making it promising to support applications in organic materials science and medicinal chemistry.

## Data availability

The ESI† contains method description, product characterization data, NMR spectra, and mechanism study details.

## Author contributions

QL, MZ and JW conceived and designed the project. QL, PD, and HT performed all experimental and mechanism studies. QL, MZ, and JW wrote the manuscript.

## Conflicts of interest

There are no conflicts to declare.

## Supplementary Material

SC-013-D2SC03047K-s001
