# Supplementary material for: Photomediated reductive coupling of nitroarenes with aldehydes for amide synthesis
Source: Chem Sci. 2022 Aug 3;13(32):9361–5. doi: 10.1039/d2sc03047k (PMC9384791; doi:10.1039/d2sc03047k)
Supplement: SC-013-D2SC03047K-s001 [file SC-013-D2SC03047K-s001.pdf]

Electronic Supplementary Material (ESI) for Chemical Science.
This journal is © The Royal Society of Chemistry 2022

Supplementary Materials for

Photomediated Reductive Coupling of Nitroarenes with Aldehydes for Amide Synthesis

Qingyao Li,[a] Peng Dai,[a] Haidi Tang,[a] Muliang Zhang,*[a] and Jie Wu*[a]

[a] Department of Chemistry, National University of Singapore, 3 Science Drive 3, 117543, Singapore

*Corresponding author. Email: chmjie@nus.edu.sg; muliang0206@foxmail.com

Contents

I. General Information 2
II. Optimization for Reductive Amidation 2
III. General Procedures for TBADT-Catalyzed Reductive Amidation 4
IV. Mechanistic Studies 5
Evaluation of potential *N*-based intermediates 5
Radical trapping experiments: 5
Detection of *N*-hydroxyamide 6
Evaluation of the reduction of *N*-hydroxyamide. 8
Time scale study 11
Cyclic Voltammetry data 13
Calculation of redox potential of silyl radical 15
Determination of Quantum Yield 17
Kinetic Isotope Effect study 18
Proposed mechanisms 19
V. Scale Up in Continuous-Flow Reactors 22
Flow reactor setup 22
Reductive amidation in flow 22
VI. Characterization Data of Products 24
VII. NMR Spectra 39
VIII. Supplementary References 96

I. General Information

Chemicals and anhydrous solvents were purchased from commercial suppliers and used as received. Commercial unavailable substrates were synthesized according to literature (**S-3tt**,[1] **S-3vv**[1]). ^{1}H NMR, ^{13}C NMR, ^{19}F NMR spectra were recorded on a Bruker AV-III400 (400 MHZ) or AMX500 (500 MHz) spectrometer. Chemical shifts were calibrated using residual undeuterated solvent as an internal reference ($CDCl_3$: 7.26 ppm ^{1}H NMR, 77.16 ppm ^{13}C NMR; DMSO-d_6: ^{1}H NMR, 2.50 ppm ^{13}C NMR 40.00 ppm). Multiplicity was indicated as follows: s (singlet), d (doublet), t (triplet), q (quartet), m (multiplet), dd (doublet of doublet), brs (broad singlet). High-resolution mass spectra (HRMS) were obtained on a Finnigan/MAT 95XL-T spectrometer. GC analysis was performed on Aglilent 7820A & 5977E GC-MS. The Kessil PR160 series (λ_{max} = 390 nm, 40 W) were used as the purple LED light source for the batch and continuous-flow reactions. All catalytic reactions were carried out in Schlenk tubes (20 mL) under an argon atmosphere with magnetic stirring after repeated freeze-pump-thaw. The isolated yield was the purified state by flash chromatography over silica gel.

II. Optimization for Reductive Amidation

Supplementary Table S1. Optimization of solvents.

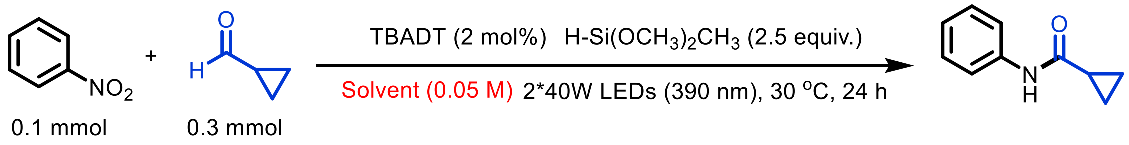

Entry	Solvent	Conv. (%)[(a)]	Yield (%)[(a)]
1	CH_3CN	100	40
2	acetone	70	<10
3	t-BuOH	56	<10
4	EtOAc	20	<10
5	DCM	30	<10
6	DMA	15	<10

[a]Conversions and yields based on analysis of the ^{1}H NMR spectra of the crude product mixture using CH_2Br_2 as an internal standard.

Supplementary Table S2. Evaluation of different silanes as the reductants.

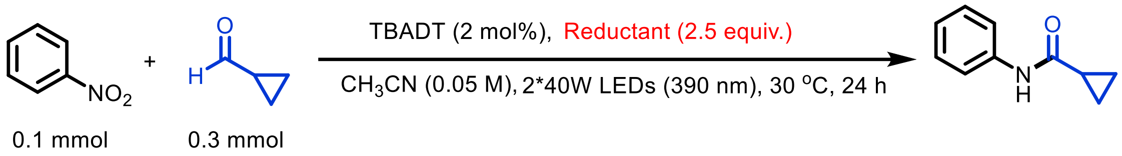

Entry	Reductant	Conv. (%)[(a)]	Yield (%)[(a)]
1	H-SiTMS$_3$	100	25
2	Ph_2SiH_2	100	24
3	$PhSiH_3$	100	15
4	H-Si(OCH_3)$_3$	100	14
5	H-SiEt$_3$	100	36
6	H-Si(OCH_3)$_2CH_3$	100	40

[a]Conversions and yields based on analysis of the ^{1}H NMR spectra of the crude product mixture using CH_2Br_2 as an internal standard.

Supplementary Table S3. Evaluation of additives.

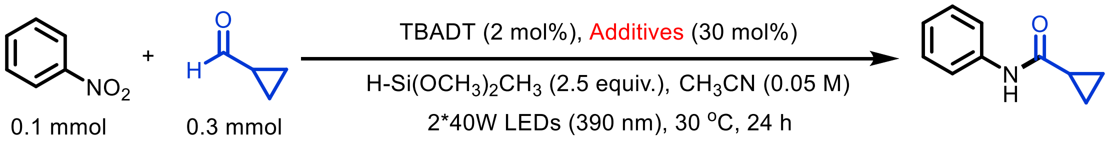

Entry	Additives	Conv. (%)[(a)]	Yield (%)[(a)]
1	CF_3COOH	100	81
2	H_2SO_4	30	21
3	HCl	35	23
4	CF_3SO_3H	25	15
5	HCOOH	100	43
6	CH_3COOH	100	41
7	Na_2CO_3	100	40
8	$NaHCO_3$	100	38

[a]Conversions and yields based on the analysis of the ^{1}H NMR spectra of the crude product mixture using CH_2Br_2 as an internal standard.

Supplementary Table S4. Evaluation of the equivalent of additives and reaction temperatures.

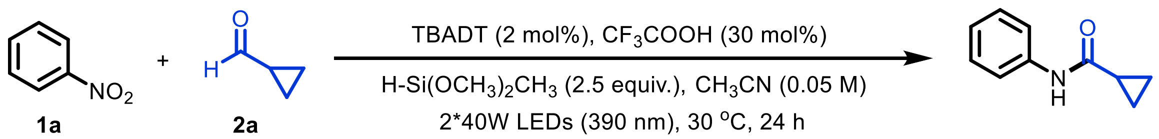

1a **2a**

Entry	Variation from standard conditions[a]	Conv (%)[b]	Yield (%)[b]
1	none	100	81
2	10 mol% TFA	100	74
3	20 mol% TFA	100	76
4	40 °C instead of 30 °C	100	72
5	50 °C instead of 30 °C	100	68
6	60 °C instead of 30 °C	100	58

[a]Standard conditions: **1a** (0.1 mmol), **2a** (3 equiv), TBADT (2 mol%), CF_3COOH (30 mol%), H-Si$(OCH_3)_2CH_3$ (2.5 equiv.), CH_3CN (2mL), purple LEDs, 30°C, 24h.
[b]Conversions and yields based on the analysis of the ^{1}H NMR spectra of the crude product mixture using CH_2Br_2 as an internal standard.

III. General Procedures for TBADT-Catalyzed Reductive Amidation

General procedure:

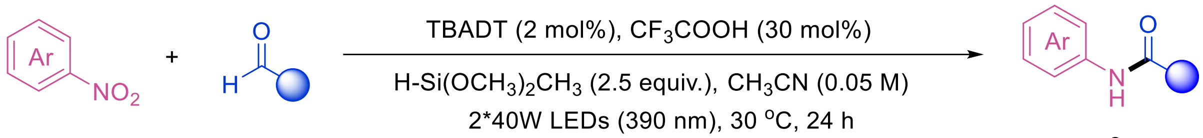

1 **2** **3**

A 20 mL Schlenk tube equipped with a magnetic stir bar was charged with TBADT (0.002 mmol, 6.6 mg), nitroarene **1** (0.1 mmol), aldehyde **2** (0.3 mmol), dimethoxy(methyl)silane (0.25 mmol, 31 μL) and trifluoroacetic acid (0.03 mmol, 2.5 μL). Then, 2.0 mL of anhydrous acetonitrile was added. The Schlenk tube was connected to Schlenk line and freeze-pump-thaw was performed for three times to completely remove air inside the reaction mixture. Eventually the Schlenk tube was refilled with an atmosphere of argon at room temperature and sealed. The reactor was irradiated by 2 sets of 390 nm Kessil lamps. Then the reaction was running at ambient temperature (~30 °C) using a fan to cool down the reaction mixture and stopped after 24 h. The reaction was quenched by the triethylamine and then remove the solvent under reduced pressure, the crude mixture was purified by silica gel column chromatography (eluent: hexane/diethyl ether or hexane/ethyl acetate; 10/1 – 1/1) to give the corresponding product **3**.

IV. Mechanistic Studies

Evaluation of potential *N*-based intermediates

Supplementary Table S5. Control experiments using different *N*-based starting material.

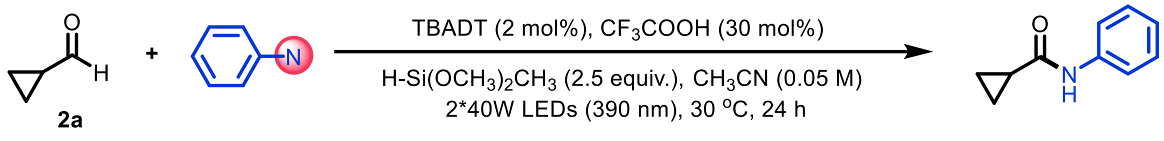

Entry	Variation from standard conditions[(a)]	Conv. (%)[(b)]	Yield (%)[(b)]
1	none	100	81
2	PhNO	100	82
3	Ph-NH_2	0	0
4	Ph-N(H)OH	20	10
5	Ph-N=N-Ph	31	0

[a]Standard conditions: *N*-based intermediate (0.1 mmol), **2a** (3 equiv.), TBADT (2 mol%), CF_3COOH (30 mol%), H-Si$(OCH_3)_2CH_3$ (2.5 equiv.), CH_3CN (2mL), purple LEDs, 30°C, 24h.
[b]Conversions and yields based on analysis of the ^{1}H NMR spectra of the crude product mixture using CH_2Br_2 as an internal standard.

Radical trapping experiments:

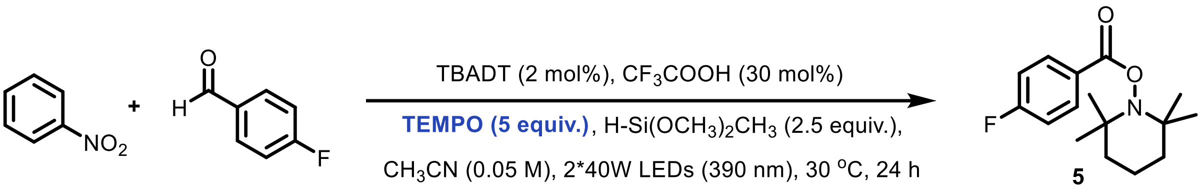

5

detected by ESI-MS

A 20 mL Schlenk tube equipped with a magnetic stir bar was charged with TBADT (0.002 mmol, 6.6 mg), nitrobenzene (0.1 mmol, 10 μL), 4-Fluorobenzaldehyde (0.3 mmol, 32 μL), dimethoxy(methyl)silane (0.25 mmol, 31 μL), trifluoroacetic acid (0.03 mmol, 2.5 μL) and TEMPO (0.5 mmol, 78.1 mg). Then, 2.0 mL of anhydrous acetonitrile was added. The Schlenk tube was connected to Schlenk line and freeze-pump-thaw was performed for three times to completely remove air inside the reaction mixture. Eventually the Schlenk tube was refilled with an atmosphere of argon at room temperature and sealed. The reactor was irradiated by 2 sets of 390 nm Kessil lamps. Then the reaction was running at ambient temperature (~30 °C) using a fan to cool down the reaction mixture and stopped after 24 h. The reaction was quenched by the triethylamine and then remove the solvent under reduced pressure, the crude mixture was analyzed by ESI-MS.

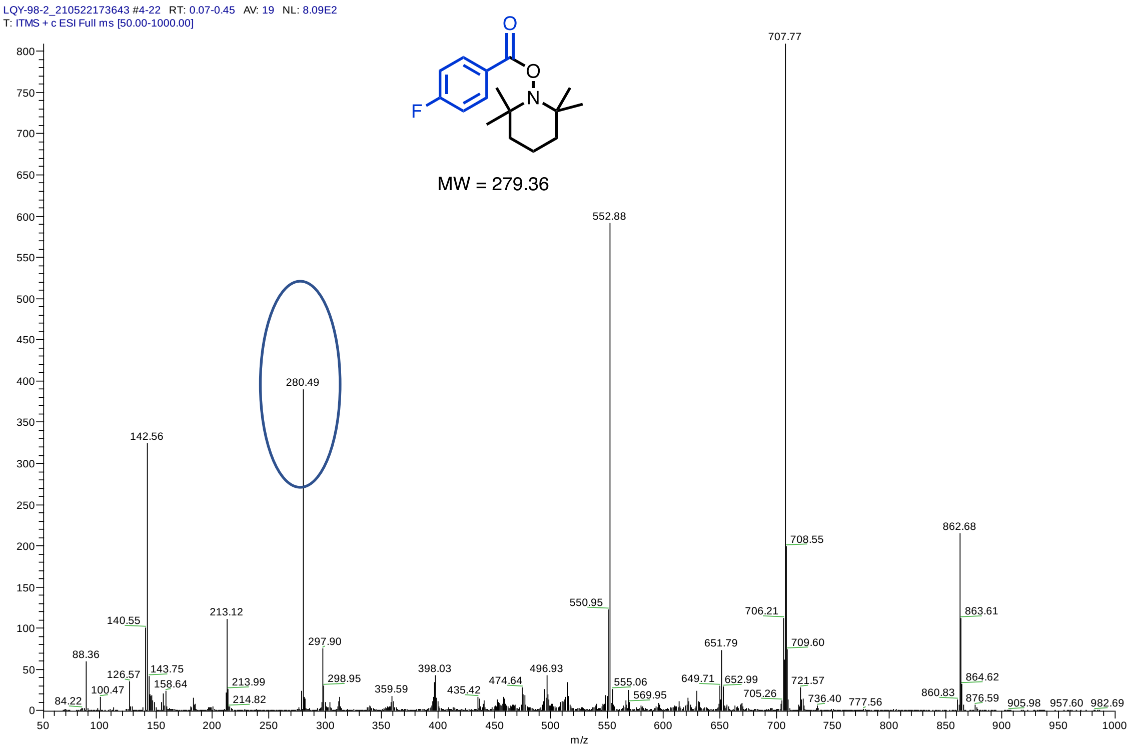

Supplementary Figure S1. Trapping acyl radical by TEMPO.

Detection of *N*-hydroxyamide.

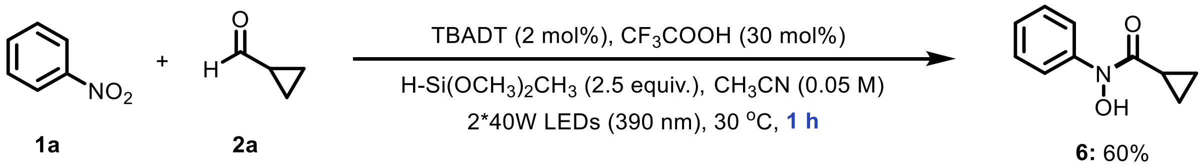

Supplementary Figure S2. Detection of *N*-hydroxyamide after 1 h reaction.

A 20 mL Schlenk tube equipped with a magnetic stir bar was charged with TBADT (0.002 mmol, 6.6 mg), nitrobenzene **1a** (0.1 mmol, 10 μL), and aldehyde **2a** (0.3 mmol, 25 μL), dimethoxy(methyl)silane (0.25 mmol, 31 μL) and trifluoroacetic acid (0.03 mmol, 2.5 μL). Then, 2.0 mL of anhydrous acetonitrile was added. The Schlenk tube was connected to Schlenk line and freeze-pump-thaw was performed for three times to completely remove air inside the reaction mixture. Eventually the Schlenk tube was refilled with an atmosphere of argon at room temperature and sealed. The reactor was irradiated by 2 sets of 390 nm Kessil lamps. Then the reaction was running at ambient temperature (~30 ℃) using a fan to cool down the reaction mixture and stopped after 1 h. The reaction was quenched by the triethylamine and then remove the solvent under reduced pressure, the crude mixture was purified by silica gel column chromatography to give the *N*-hydroxyamide **6** in 60% yield.

Supplementary Table S6. Further control experiments.

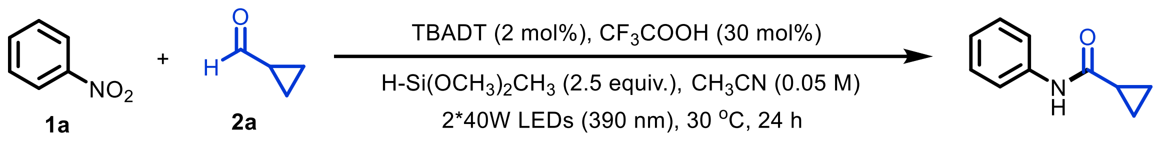

Entry	Variation from standard conditions[a]	Conv (%)[b]	Yield (%)[b]
1	without Silane	>90	50
2	without CF_3COOH	>90	40
3	without Aldehyde	0	0
4	without TBADT	12	6
5	without light irridation	>90	0

[a]Standard conditions: **1a** (0.1 mmol), **2a** (3 equiv), TBADT (2 mol%), CF_3COOH (30 mol%), H-Si(OCH_3)$_2$$CH_3$ (2.5 equiv.), CH_3CN (2mL), purple LEDs, 30°C, 24h.

[b]Conversions and yields based on analysis of the ^{1}H NMR spectra of the crude product mixture using CH_2Br_2 as an internal standard.

Notably, without adding CF_3COOH in the reaction (**Table S6**, entry 2), we isolate a major byproduct **9.** The addition of CF_3COOH improves the yield and inhibits side reaction between amide anion and aldehyde, demonstrating CF_3COOH could accelerate the process and increase the reactivity.

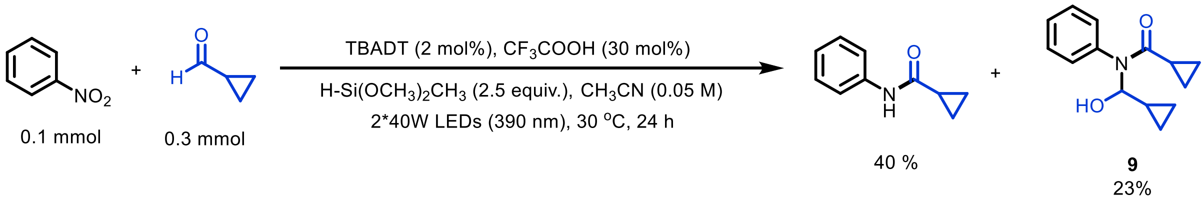

Evaluation of the reduction of *N*-hydroxyamide.

Supplementary Table S7. Evaluation the reduction of *N*-hydroxyamide.

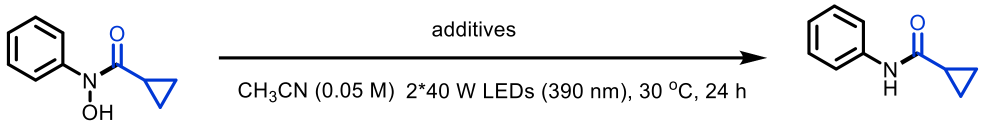

Entry	Additives	Yield (%)[a]
1	$H\text{-}Si(OCH_3)_2CH_3$ (2.5 equiv.)	30
2	cyclopropanecarboxaldehyde (3 equiv.)	35
3	TBADT (2 mol%), $H\text{-}Si(OCH_3)_2CH_3$ (2.5 equiv.)	100
4	TBADT (2 mol%), cyclopropanecarboxaldehyde (3 equiv.)	100
5	$H\text{-}Si(OCH_3)_2CH_3$ (2.5 equiv.), cyclopropanecarboxaldehyde (3 equiv.)	68
6	$H\text{-}Si(OCH_3)_2CH_3$ (2.5 equiv.), without light	<5
7	only with 390 nm UV-light	10

[a]Yields based on analysis of the ^1H NMR spectra of the crude product mixture using CH_2Br_2 as an internal standard.

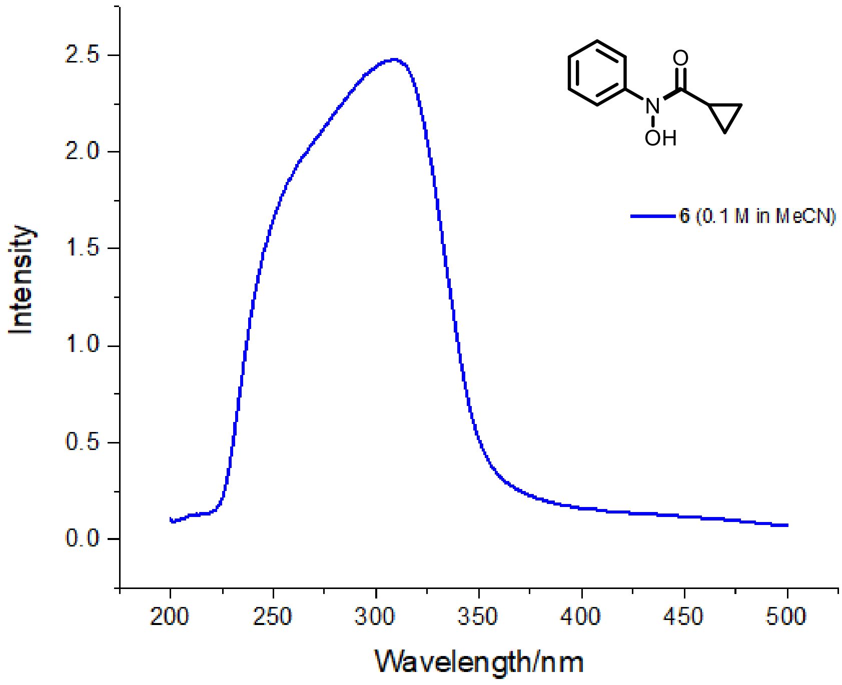

Supplementary Figure S3. UV-Vis spectrum of *N*-hydroxyamide in MeCN

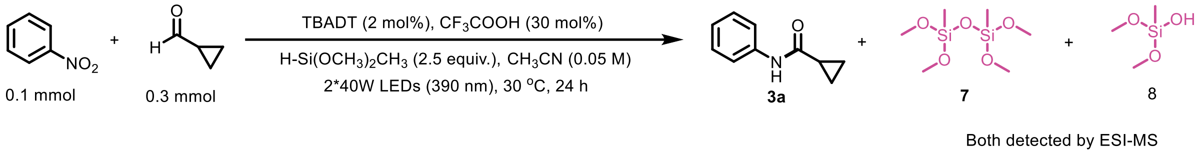

Supplementary Figure S4. Detection of siloxane and silanol byproducts.

A 20 mL Schlenk tube equipped with a magnetic stir bar was charged with TBADT (0.002 mmol, 6.6 mg), nitrobenzene **1a** (0.1 mmol, 10 μL), and aldehyde **2a** (0.3 mmol, 25 μL), dimethoxy(methyl)silane (0.25 mmol, 31 μL) and trifluoroacetic acid (0.03 mmol, 2.5 μL). Then, 2.0 mL of anhydrous acetonitrile was added. The Schlenk tube was connected to Schlenk line and freeze-pump-thaw was performed for three times to completely remove air inside the reaction mixture. Eventually the Schlenk tube was refilled with an atmosphere of argon at room temperature and sealed. The reactor was irradiated by 2 sets of 390 nm Kessil lamps. Then the reaction was running at ambient temperature (~30 ℃) using a fan to cool down the reaction mixture and stopped after 24 h. The reaction was quenched by the triethylamine and then remove the solvent under reduced pressure, the crude mixture was analyzed by ESI-MS.

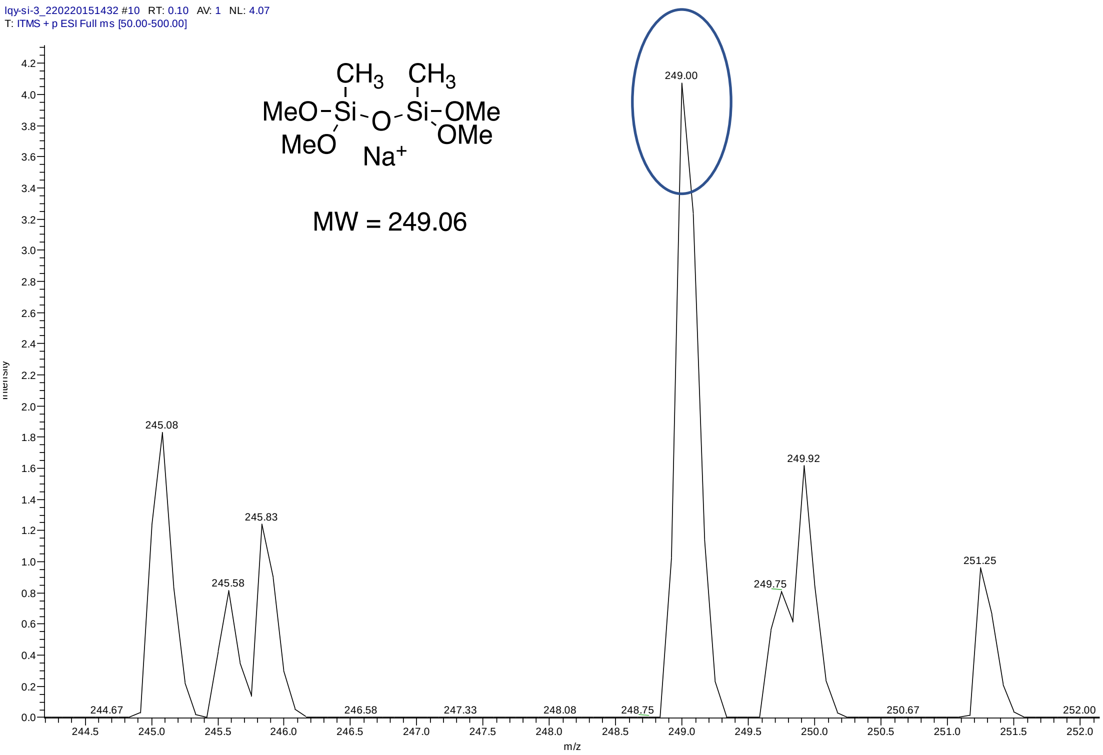

Supplementary Figure S5. Detection of siloxane by ESI-MS.

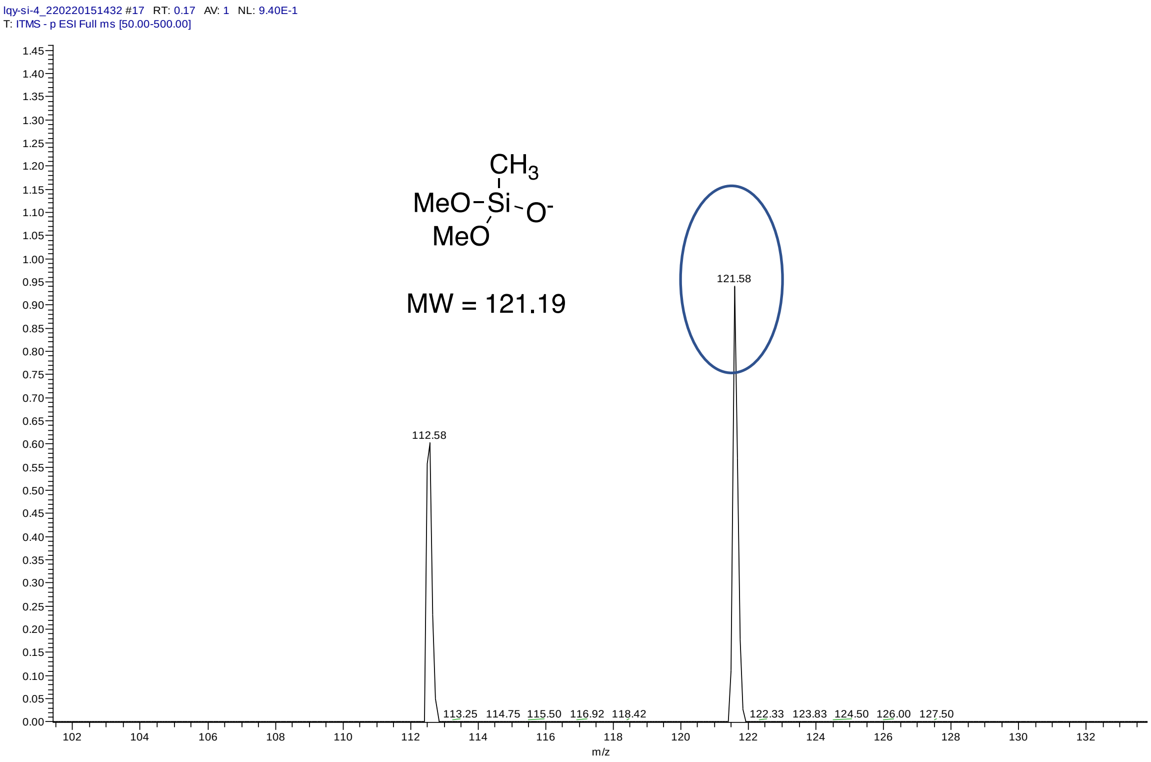

Supplementary Figure S6. Detection of silanol by ESI-MS.

Time scale study

To examine the impact of light, we conducted experiments under alternating periods of irradiation and darkness (**Figure S7-S8**). These resulted in a total interruption of the reaction progress in the absence of light and recuperation of reactivity on further illumination, which allows precise temporal control over the entire reaction period. These results demonstrate that light is a necessary component of the reaction. Even though they do not definitively rule out a radical-chain process, the data show that any chain-propagation process must be short-lived.

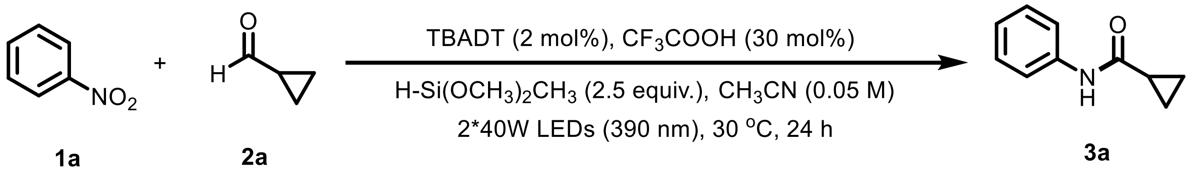

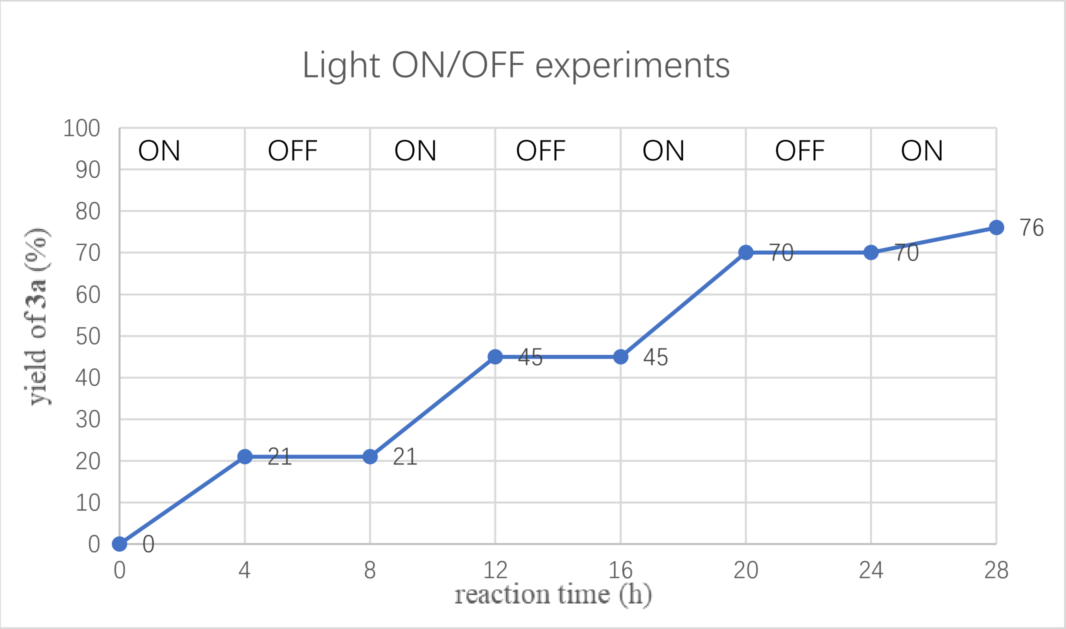

Supplementary Figure S7. Time profile of the Generation of **3a** with the light ON/OFF over time.

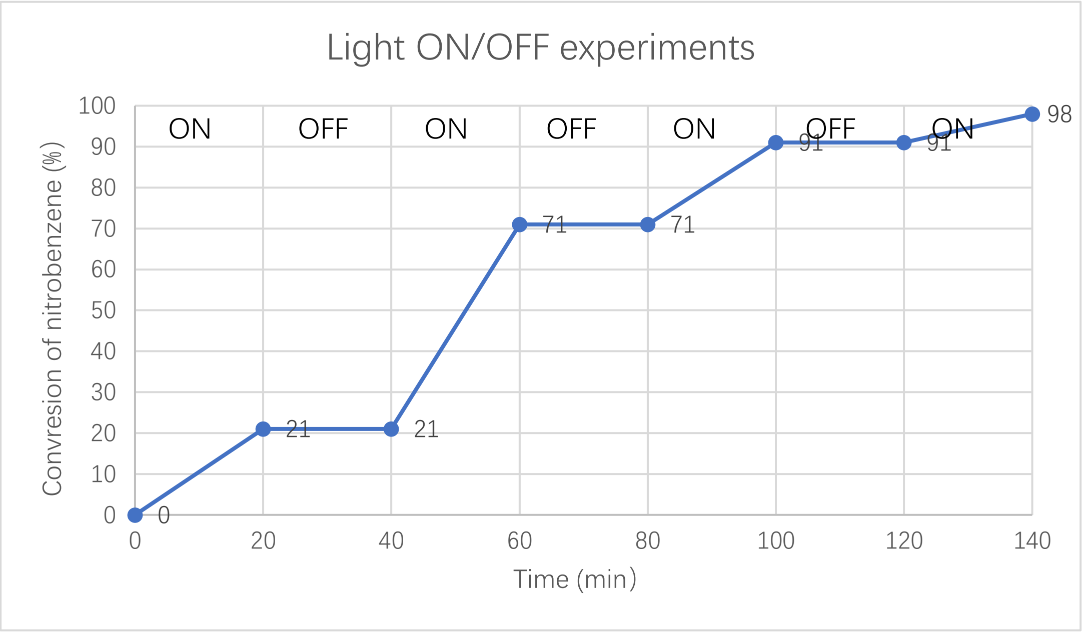

Supplementary Figure S8. Time profile of the Conversion of Nitrobenzene with the light ON/OFF over time.

Cyclic Voltammetry data

Cyclic voltammetry (CV) was performed using a VersaSTAT 3 Potentiostat Galvanostat from Princeton Applied Research. A three-electrode setup was used, consisting of a glassy carbon working electrode, Pt wire counter electrode, and a Ag/AgCl reference electrode. Data were acquired in MeCN, with a scan rate of 100 mV/s, in the presence of Bu_4NPF_6 electrolyte (0.1 M), and reported with reference to Ag/AgCl. The samples were sparged with Argon for at least 5 minutes prior to data collection.

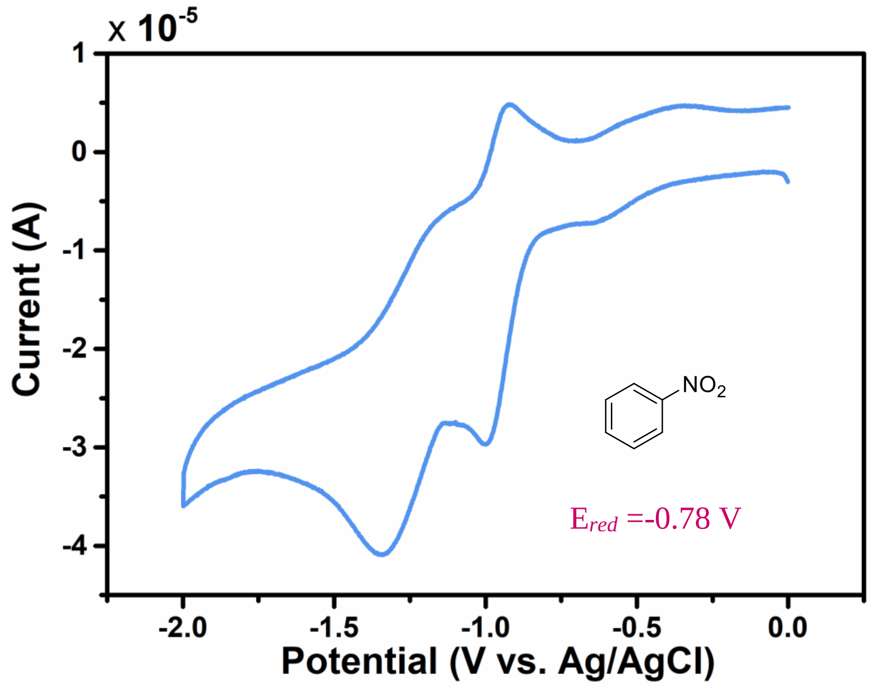

Supplementary Figure S9. CV of nitrobenzene (5 mM) in MeCN

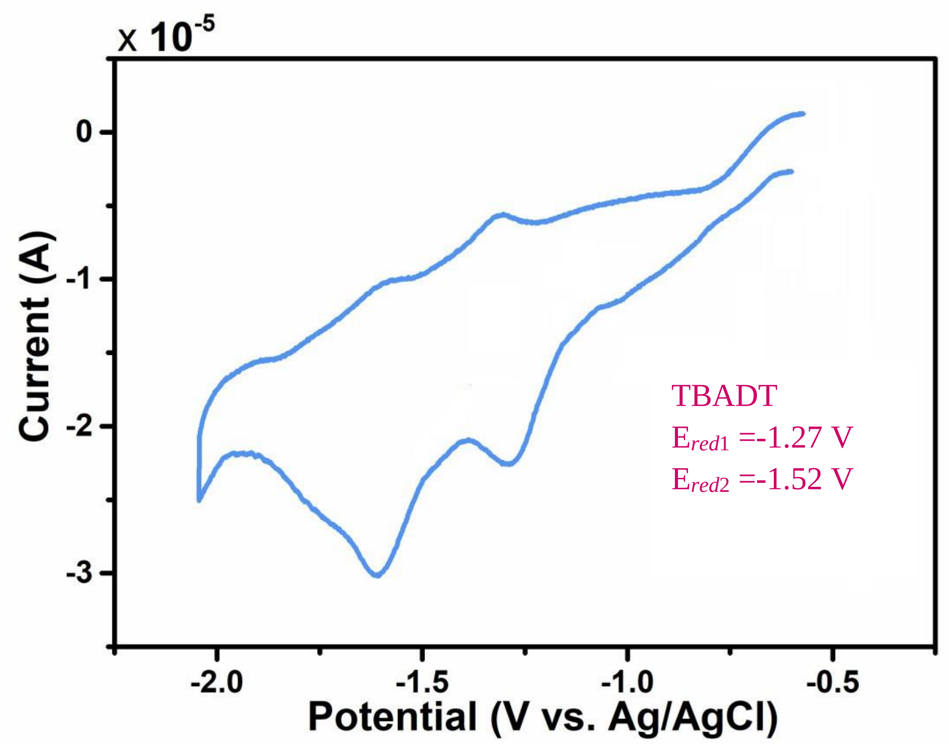

Supplementary Figure S10. CV of TBADT (2 mM) in MeCN

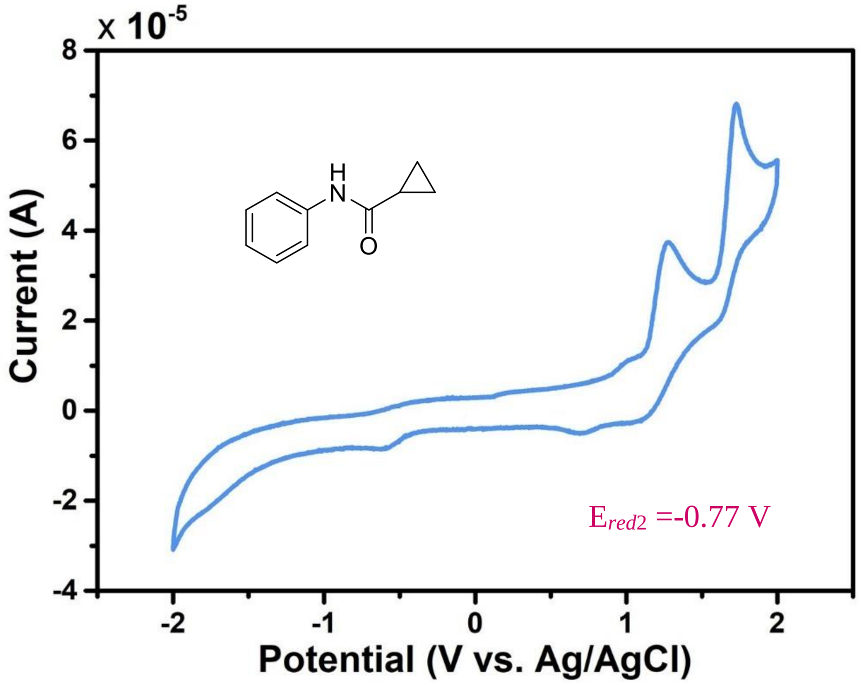

Supplementary Figure S11. CV of *N*-phenylbenzamide (5 mM) in MeCN

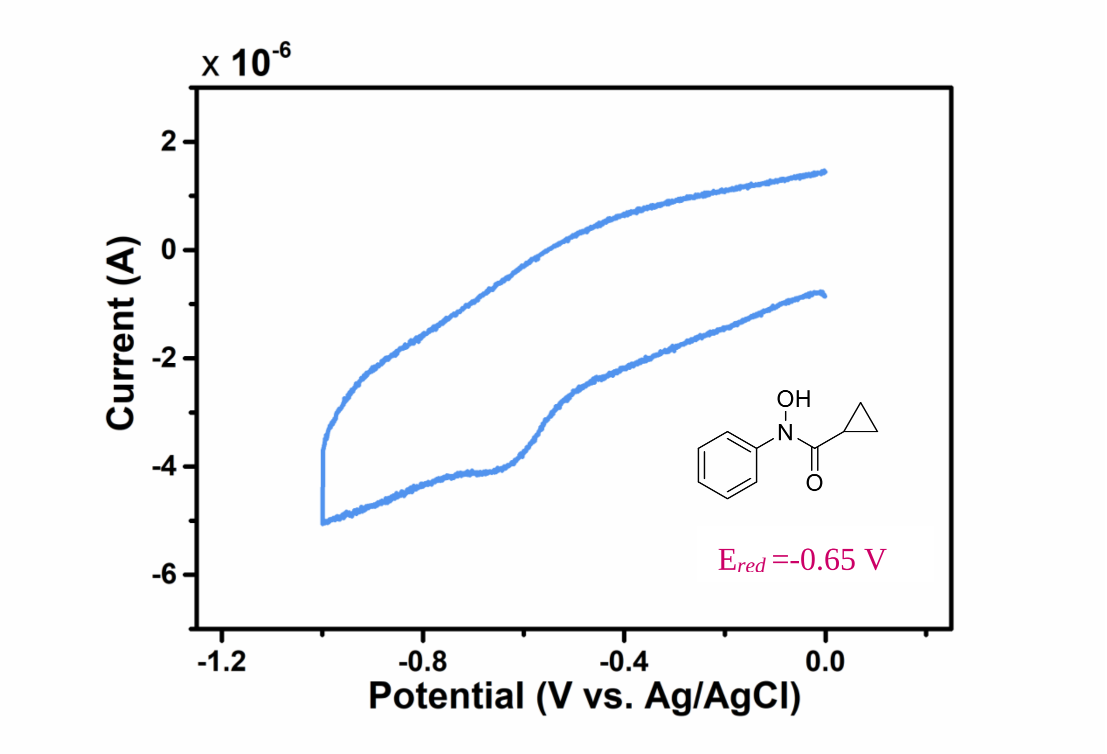

Supplementary Figure S12. CV of *N*-hydroxy-*N*-phenylbenzamide (5 mM) in MeCN

Calculation of redox potential of silyl radical

All DFT calculations were performed with the Gaussian 16 package. Geometry optimizations were performed for the reduced and oxidized forms of each molecule at B97D37-8 level of theory with the Def2QZVPP[2] basis set by using CPCM[3] solvation model (solvent = acetonitrile) under 298.15 K and 1 atm pressure. Frequency analysis was conducted at the same level of theory to verify the stationary points with no imaginary frequencies. The solution phase electrochemical redox potentials are calculated based on ***eq.* S1**.[4]

$$E_{1/2}^{o,cacl} = -\frac{G_{298}[reduced] - G_{298}[oxidized]}{n_e F} - E_{1/2}^{o,SHE} + E_{1/2}^{o,SCE} \quad (\boldsymbol{eq.S1})$$

n_e , The number of electrons transferred
F =23.061 kcal mol^{-1} V^{-1} , Faraday constant
$E_{1/2}^{o,SHE}$ = 4.281 V, Absolute value for the standard hydrogen electrode (SHE)
$E_{1/2}^{o,SCE}$ = - 0.141 V, the potential of saturated calomel electrode (SCE) relative to SHE in acetonitrile
$G_{298}[reduced]$ and $G_{298}[oxidized]$ are the Gibbs free energies in acetonitrile as gathered from DFT calculations.

Supplementary Table S8. Energies and Redox potentials

	G_{298}[reduced] (a.u)	G_{298}[oxidized] (a.u)	$E^{o,calc}_{1/2}$
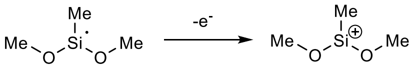 "reduced" "oxidized"	-559.619241	-559.475875	$E^{o,calc}_{1/2}$ = -0.52 V vs SCE

Cartesian Coordinates

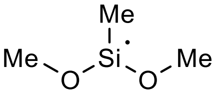

H	-2.83308400	0.56167500	-0.62480500
H	-2.91345200	-0.19647500	0.98792100
H	-3.28947600	-1.15251500	-0.46528400
H	2.91341300	-0.19514300	0.98745600
H	2.83272100	0.56068300	-0.62634000
H	3.28928900	-1.15324000	-0.46442300
H	-0.88593900	2.38100400	0.20626800
H	0.00065900	1.87546800	-1.25533900
H	0.88691800	2.38060900	0.20661300
C	-2.65158700	-0.36207800	-0.06363800
C	2.65139000	-0.36228000	-0.06382300
O	-1.28946500	-0.78604200	-0.20367100
C	0.00044300	1.85464300	-0.15849400
Si	-0.00007800	0.05263500	0.42981800
O	1.28928600	-0.78654000	-0.20305200

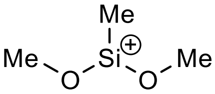

H	-2.94253400	0.18314800	-0.87184600
H	-2.96554100	0.08632100	0.91939100
H	-3.26213400	-1.37914000	-0.06200300
H	2.96449600	0.09113000	0.91635200
H	2.94267100	0.17762800	-0.87543100
H	3.26181500	-1.37993100	-0.05630900
H	-0.89606500	2.31697000	0.49560700
H	0.00237900	2.27789800	-1.04033800
H	0.89673200	2.31594400	0.49797000
C	-2.71966500	-0.43764500	-0.00409300
C	2.71924400	-0.43815400	-0.00426000
O	-1.30364200	-0.78678200	-0.00087300
C	0.00076200	1.93791300	0.00111900
Si	-0.00003600	0.10932600	0.00930800
O	1.30322200	-0.78736900	-0.00041400

Determination of Quantum Yield

Determination of light intensity at 390 nm:

the photon flux of the spectrophotometer was determined by standard ferrioxalate actinometry. A 0.15 M solution of ferrioxalate was prepared by dissolving 2.21 g of potassium ferrioxalate hydrate in 30 mL of 0.05 M H_2SO_4. A buffered solution of phenanthroline was prepared by dissolving 50 mg of phenanthroline and 11.25 g of sodium acetate in 50 mL of 0.5 M H_2SO_4. Both solutions were stored in the dark. To determine the photon flux of the spectrophotometer, 2.0 mL of the ferrioxalate solution was placed in a cuvette and irradiated for 90.0 seconds at λ = 390 nm with an emission slit width at 10.0 nm. After irradiation, 0.35 mL of the phenanthroline solution was added to the cuvette. The solution was then allowed to rest for 1 h to allow the ferrous ions to completely coordinate to the phenanthroline. The absorbance of the solution was measured at 510 nm. A non-irradiated sample was also prepared and the absorbance at 510 nm measured.

Conversion was calculated using ***eq.* S2**.

***eq.* S2**

$$\text{mol Fe}^{2+} = \frac{V * \Delta A}{l * \varepsilon}$$

V: total volume (0.00235 L)

Δ : difference in absorbance at 510 nm between irradiated and non-irradiated solution

l: path length

ε: molar absorptivity at 510 nm (11100 L/ mol^{-1} cm^{-1})

$$\text{mol Fe}^{2+} = \frac{V * \Delta A}{l * \varepsilon} = \frac{0.00235L * 1.052}{1\ cm * 11100\ L/mol/cm} = 2.23 * 10^{-7}\ \text{mol}$$

Where V is the total volume (0.00235 L) of the solution after addition of phenanthroline, ΔA is the difference in absorbance at 510 nm between the irradiated and non-irradiated solutions, l is the path and ε is the molar absorptivity at 510 nm (11100 L mol^{-1} cm^{-1}).

The photon flux can be calculated using ***eq.* S3**.

***eq* S3**

$$\text{Photon flux} = \frac{mol\, Fe^{2+}}{\Phi * t * f}$$

Φ: Quantum yield for the ferrioxalate actinometer (1.13 for 0.15 M solution at λ =390 nm)

t : irradiation time (90.0 s)

f: fraction of light at λ =390 nm

$$\text{Photon flux} = \frac{mol\, Fe^{2+}}{\Phi * t * f} = \frac{2.23 * 10^{-7} mol}{1.13 * 90.0 * 1} = 2.19 * 10^{-9}\ \text{Einstein s}^{-1}$$

Where Φ is the quantum yield for the ferrioxalate actinometer (1.13 for a 0.15 M solution at λ = 390 nm), t is the time (90.0 s), and f is the fraction of light absorbed at λ =390 nm (1.0). The photon flux was calculated (average of three experiments) to be 2.19 × 10^{-9} einstein s^{-1}.

Determination of quantum yield of Reductive amidation of nitrobenzene:

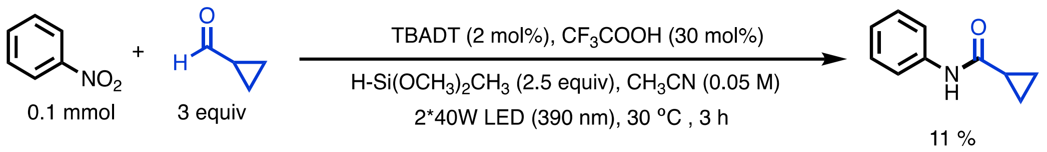

A cuvette was charged with nitrobenzene (10 uL, 0.1 mmol), Cyclopropanecarboxaldehyde (24 uL, 0.3 mmol), TBADT (6.6 mg, 0.002 mmol), dimethoxy(methyl)silane (31 uL, 0.25 mmol) and TFA (1.5 uL, 0.03 mmol) in anhydrous acetonitrile (2 mL). The cuvette was then capped with a PTFE stopper. The sample was stirred and irradiated ($\lambda = 390$ nm) for 21600 s (3 h). After irradiation, the solvent was removed. The yield of product formed was determined as 11% by crude ^{1}H NMR based on a dibromomethane standard. The quantum yield was determined using ***eq.* S4**.

Eq. S4

$$\text{yield of reaction } \Phi \text{ (11 \%)} = \frac{mol\,product}{flux*t*f} = 0.46$$

Φ (11%) = 0.46.

Kinetic Isotope Effect study.

We conducted the KIE experiments with initial rates, and the KIEs were determined from two parallel reactions for 6 h, giving $K_H/K_D = 1.13$. These results suggested that aldehydic C(sp^2)-H activation might not be involved in the rate-determining step.

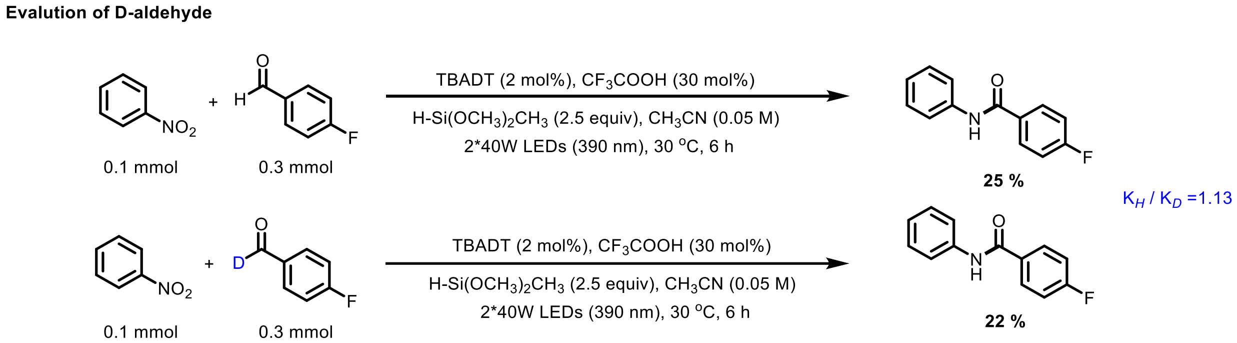

Supplementary Figure S13. Kinetic Isotope Effect measured from two parallel reactions.

Proposed mechanisms

Redox potentials of photocatalyst TBADT ($E_{1/2}^{red}$ ($[W_{10}O_{32}]^{4-}/[W_{10}O_{32}]^{5-}$) = -1.27 V vs Ag/Ag^+ in acetonitrile) and hydroxyamide (E_{red} = -0.65 V vs Ag/Ag^+ in acetonitrile) using cyclic voltommetry (CV) measurements have been obtained and the KIE study (K_H/K_D = 1.13) and quantum yield calculation (Φ=0.46) has been conducted. Carboxylic acids as byproducts are detected by ^{1}H-NMR and silanols or silyl ethers are also dectected by ESI-MS.

Based on the experimental data and previous literature, a more detailed mechanism is proposed as shown below (**Figure S14**). The excited photocatalyst TBADT abstracts a hydrogen atom from aldehydes or silanes to generate the corresponding acyl radicals or silyl radicals and $H^+[W_{10}O_{32}]^{5-}$. The acyl radicals could directly undergo a radical addition on nitroarenes to give the intermediates **I**, followed by decomposition to offer nitrosoarenes and carboxyl radicals **II**.[5] This carboxyl radical species **II** is capable of abstracting another hydrogen atom from aldehydes to deliver acyl radicals.[6] Then nitrosobenzene will react with acyl radicals to form radical species **III**, followed by a SET process with $H^+[W_{10}O_{32}]^{5-}$ to give rise to the *N*-hydroxy amide.[7] The highly reductive species $H^+[W_{10}O_{32}]^{5-}$ ($E_{1/2}^{red}$ ($[W_{10}O_{32}]^{4-}/[W_{10}O_{32}]^{5-}$) = -1.27V vs Ag/Ag^+ in acetonitrile) can also reduce the hydroxyamide (E_{red} = -0.65 V vs Ag/Ag^+ in acetonitrile) to the corresponding radical anion intermediate **VI**, which will release OH^- to generate amidyl radical **VII** followed by another SET with $[W_{10}O_{32}]^{5-}$ to give the amide anion **VIII**. Then the amide anion (**VIII**) undergoes protonation to accomplish the amide product. The addition of trifluoroacetic acid (TFA) improves the product yield and inhibits side reaction between amide anion **VIII** and aldehyde. We speculated TFA could accelerate the protonation step (see **Table S6**). In this reductive amidation, silanes are used as the reductant to improve the yield, however, it's not essential. In fact, silanes can be replaced by an excess amount of aldehydes (**Table S7**). To accomplish the catalytic cycle, at least 2.0 euqiv of reductants were required, which were consistent with our experimental results. The in situ generated silyl radical may undergo a single electron oxidation by the excited $*[W_{10}O_{32}]^{4-}$ (E ~ + 2.5 V vs SCE in MeCN[8]) to furnish a silyl cation intermediate, which is captured by nucleophiles such as H_2O and silanols to give silanols or silyl ethers as byproducts after deprotonation.[9] Density functional theory (DFT) calculations support a SET step from the silyl radical to silyl cation, as the reductive potential of dimethoxy-(methyl)silyl radical is calculated to be -0.52 V versus SCE in MeCN (**Table S8**). The detection of the silane byproducts silanols or silyl ethers also supported this process (**Figures S5-S6**).

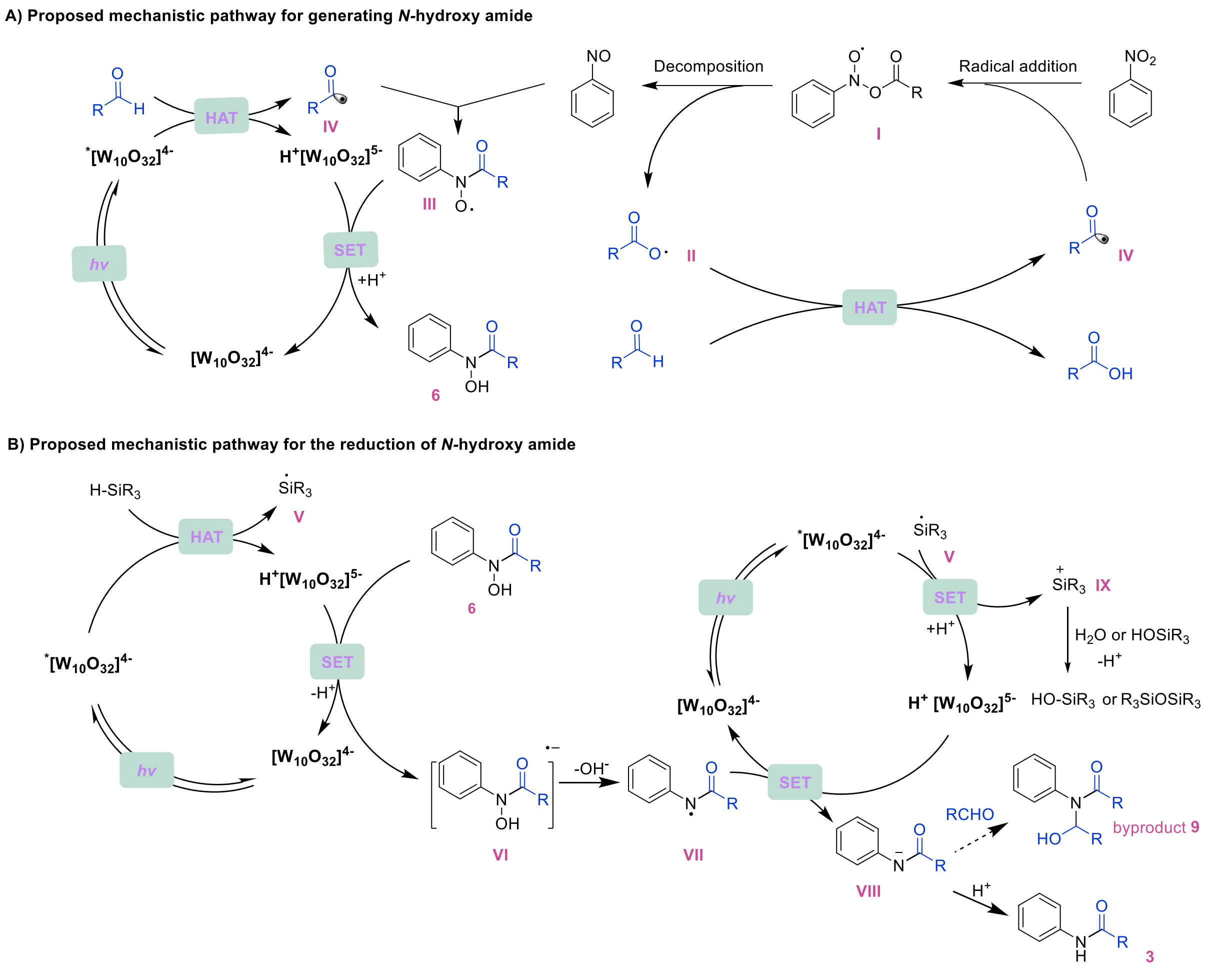

Supplementary Figure S14. Proposed mechanisms of the reductive amidation.

As for the mechanism details of the reduction of *N*-hydroxy amide, when TBADT is not used, there is still 68% conversion of *N*-hydroxy amide using 2.5 equiv of hydrosilanes and 3 equiv of aldehydes as the reductants under 390 nm light irradiation after 24 h (**Table S7**, entry 5), indicating that *N*-hydroxy amide could be directly reduced by the reductants silanes or aldehydes. Without light irradiation, the reduction of *N*-hydroxyamide barely occurred although 2.5 equiv of hydrosilanes was added (entry 6). Without any reductant, only light irradiation could not efficiently promote the reduction (entry 7). These control experiments indicated the importance of both light and reductants. Furthermore, the UV-Vis experiment shows *N*-hydroxy amide has a weak absorbance at 390 nm (**Figure S3**). A relevant report revealed that *N*-hydroxy amide could undergo homolytic cleavage under photo-irradiation conditions.[10] This may explain why without TBADT, the *N*-hydroxy amide could still be reduced to the amide under 390 nm light irradiation in the presence of reductants such as silanes or aldehydes (**Figure S15**). However, in the presence of TBADT, it only consistitutes a minor pathway to the final amide product.

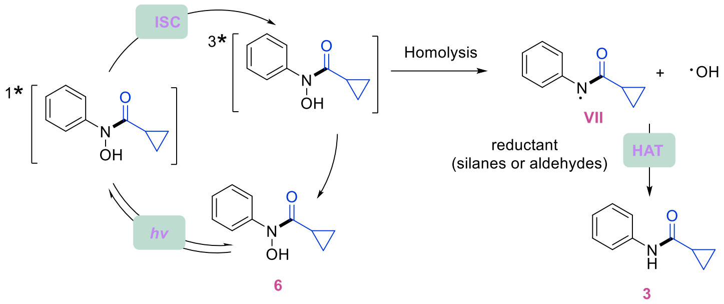

Supplementary Figure S15. Proposed mechinasms of reduction of *N*-hydroxyamide without TBADT.

V. Scale Up in Continuous-Flow Reactors

Images of flow reactors are shown in **Figure S17**. Back pressure regulators (BPRs), high-purity perfluoroalkoxy polymer (HPFA) tubing, fittings were purchased from IDEX Health & Science Technologies. The Asia syringe pump was purchased from Syrris Ltd. Selection valves were purchased from Valco Instruments Co. Inc.

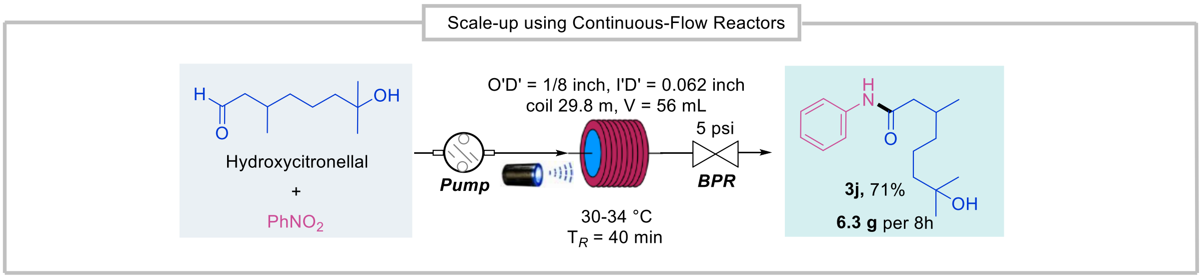

Supplementary Figure S16. The reaction scale-up in continuous-flow reactors.

Flow reactor setup

A 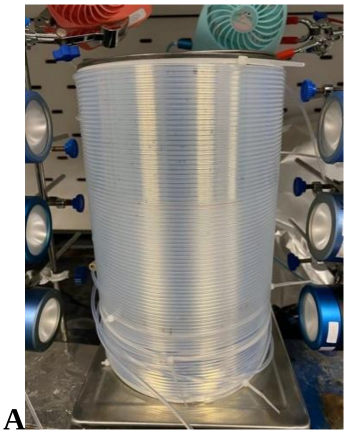 B 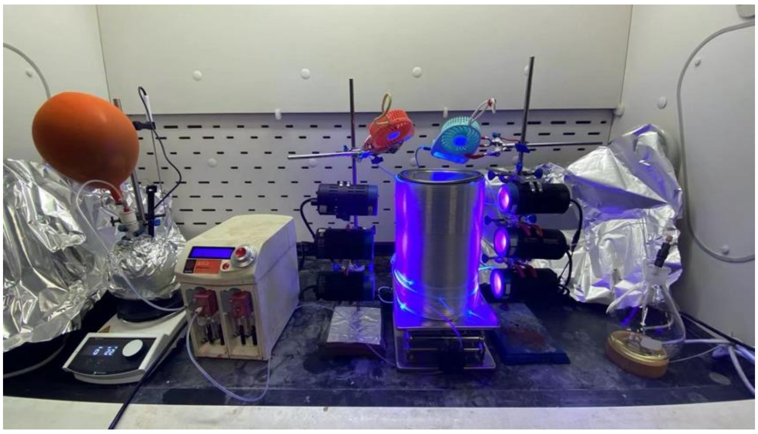

Supplementary Figure S17. The flow apparatus. **A**. The flow tubing on a stainless steel container. **B**. The flow set-up with light irradiation.

Reductive amidation in flow

Inside a glove-box, nitrobenzene **1a** (4 mL, 40 mmol), 7-hydroxy-3,7-dimethyloctanal **2j** (120 mmol, 22.4 ml), TBADT (0.8mmol, 2.7g) dimethoxy(methyl)silane (100 mmol, 12.4 mL) and trifluoroacetic acid (1 mL, 12 mmol) were added to a 1 L round bottom flask. The reagents were dissolved in degassed acetonitrile and the total volume of the solution was adjusted to 800 mL. The reaction solution was introduced to the flow apparatus (**Figure S17**). The flow apparatus was purged with degassed acetonitrile to remove the air first. An Asian pump was then connected to the reaction mixture and the tubing with 5 psi back-pressure regulator (BPR). The HPFA (high purity

perfluoroalkoxyalkane) tubing (O.D. = 1/8 inch, I.D. = 0.062 inch, length = 29.8 m, volume = 56 mL) was rounded on a stainless steel container (O.D. = 10 cm). The tubing was then irradiated by six sets of 390 nm Kessil lamps (the light intensity for each was set 40 W). The flow apparatus was cooled by two fans, keeping the ambient temperature around 30-34 °C. The flow apparatus itself was set up with residence time (T_R) = 40 min, flow rate = 1.4 mL/min. After 80 min of equilibration, the product mixture was collected for 8h. A crude sample (5.6 mL) was taken from the collected solution and analyzed by ^{1}H NMR spectroscopy using CH_2Br_2 (14 μL, 0.2 mmol) as an internal standard. Full conversion of nitrobenzene (**1a**) was observed and the ^{1}H NMR yield of product **3j** was 75%. The crude NMR sample was recovered and combined with the reaction mixture. The combined crude was concentrated and purified by column chromatography to give product **3j** (6.3 g) in 71% yield.

VI. Characterization Data of Products

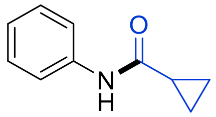

***N*-phenylcyclopropanecarboxamide (3a).** Following the general procedure, the title compound (12.6 mg) was obtained in 78%. **^{1}H NMR** (400 MHz, Chloroform-*d*) δ 7.51 (d, *J* = 7.9 Hz, 2H), 7.32 (q, *J* = 9.2, 7.9 Hz, 2H), 7.09 (t, *J* = 7.4 Hz, 1H), 1.50 (s, 1H), 1.10 – 1.07 (m, 2H), 0.87 – 0.83 (m, 2H). **^{13}C NMR** (100 MHz, DMSO-d_6) δ 172.1, 139.9, 129.2, 123.4, 119.5, 15.0, 7.6. HRMS ESI $[M+H]^+$ Calculated for $C_{10}H_{12}NO$ 162.0919, found 162.0917.

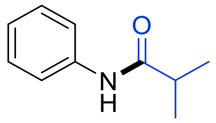

***N*-phenylisobutyramide (3b).** Following the general procedure, the title compound (10.3 mg) was obtained in 63%. **^{1}H NMR** (400 MHz, Chloroform-*d*) δ 7.53 (d, *J* = 9.7 Hz, 2H), 7.37 – 7.27 (m, 2H), 7.11 (d, *J* = 6.2 Hz, 2H), 2.51 (p, *J* = 6.9 Hz, 1H), 1.26 (d, *J* = 6.9 Hz, 6H). **^{13}C NMR** (100 MHz, Chloroform-*d*) δ 175.0, 137.3, 129.0, 124.1, 119.7, 36.8, 19.6. HRMS ESI $[M+H]^+$ Calculated for $C_{10}H_{14}NO$ 164.1075, found 164.1078.

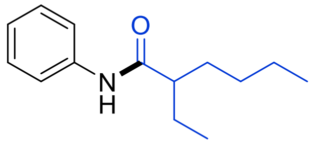

2-ethyl-*N*-phenylhexanamide (3c). Following the general procedure, the title compound (18.9 mg) was obtained in 65%. **^{1}H NMR** (400 MHz, DMSO-d_6) δ 9.97 (s, 1H), 7.64 (d, *J* = 8.0 Hz, 2H), 7.27 (dd, *J* = 8.5, 7.4 Hz, 2H), 7.16 – 6.96 (m, 1H), 2.32 (tt, *J* = 9.5, 5.2 Hz, 1H), 1.70 – 1.48 (m, 2H), 1.47 – 1.29 (m, 2H), 1.26 (d, *J* = 8.6 Hz, 4H), 0.84 (td, *J* = 7.3, 2.2 Hz, 6H). **^{13}C NMR** (100 MHz, DMSO-d_6) δ 175.1, 140.2, 129.0, 123.4, 119.7, 52.5, 32.4, 29.8, 26.2, 14.4, 12.3, 7.7. HRMS ESI $[M+H]^+$ Calculated for $C_{14}H_{22}NO$ 220.1701, found 220.1701.

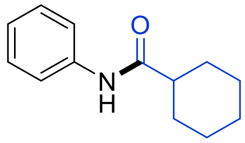

***N*-phenylcyclohexanecarboxamide (3d).** Following the general procedure, the title compound (14.8 mg) was obtained in 73%. **^{1}H NMR** (400 MHz, DMSO-d_6) δ 9.80 (s, 1H), 7.88 – 7.53 (m, 2H), 7.44 – 7.20 (m, 2H), 7.06 – 6.84 (m, 1H), 2.33 (tt, *J* = 11.7, 3.4 Hz, 1H), 1.77 (ddt, *J* = 18.2, 15.0, 3.0 Hz, 4H), 1.70 – 1.59 (m, 1H), 1.42 (qd, *J* = 12.2, 2.7 Hz, 2H), 1.37 – 1.15 (m, 3H). **^{13}C NMR** (100 MHz, DMSO-d_6) δ 179.6, 144.8, 133.8, 128.0, 124.3, 50.1, 34.4, 30.6, 30.5. HRMS ESI $[M+H]^+$ Calculated for $C_{13}H_{18}NO$ 204.1388, found 204.1386.

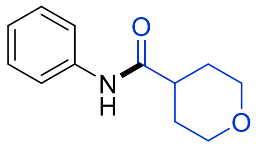

3e

***N*-phenyltetrahydro-2*H*-pyran-4-carboxamide (3e).** Following the general procedure, the title compound (12.5 mg) was obtained in 61%. **^{1}H NMR** (400 MHz, DMSO-d_6) δ 9.86 (s, 1H), 7.76 – 7.50 (m, 2H), 7.46 – 7.19 (m, 2H), 7.10 – 6.74 (m, 1H), 4.05 – 3.84 (m, 2H), 3.44 – 3.34 (m, 2H), 2.58 (tt, *J* = 10.4, 5.4 Hz, 1H), 1.70 – 1.63 (m, 4H). **^{13}C NMR** (100 MHz, Chloroform-*d*) δ 172.4, 129.0, 124.3, 119.8, 112.9, 71.1, 37.2, 29.2. HRMS ESI [M+H]$^+$ Calculated for $C_{12}H_{16}NO_2$ 206.1181, found 206.1184.

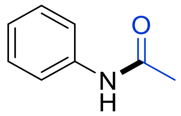

***N*-phenylacetamide (3f).** Following the general procedure but adding 20 equivalent aldehyde, the title compound (12.5 mg) was obtained in 60%. **^{1}H NMR** (400 MHz, Chloroform-*d*) δ 7.49 (d, *J* = 7.4 Hz, 2H), 7.32 (t, *J* = 7.9 Hz, 2H), 7.23 (s, 1H), 7.10 (t, *J* = 7.4 Hz, 1H), 2.18 (s, 3H). **^{13}C NMR** (100 MHz, DMSO-d_6) δ 168.8, 139.8, 129.1, 123.4, 119.5, 24.5. HRMS ESI [M+H]$^+$ Calculated for $C_8H_{10}NO$ 136.0762, found 136.0761.

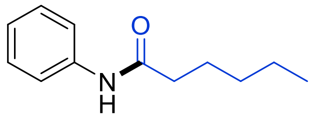

***N*-phenylhexanamide (3g).** Following the general procedure, the title compound (15.5 mg) was obtained in 81%. **^{1}H NMR** (400 MHz, Chloroform-*d*) δ 7.51 (d, *J* = 7.9 Hz, 2H), 7.37 – 7.28 (m, 2H), 7.15 – 7.05 (m, 2H), 2.35 (t, *J* = 7.6 Hz, 2H), 1.73 (q, *J* = 7.4 Hz, 2H), 1.36 (h, *J* = 3.8 Hz, 4H), 1.07 – 0.79 (m, 3H). **^{13}C NMR** (100 MHz, DMSO-d_6) δ 172.0, 140.0, 129.0, 123.3, 119.5, 36.8, 31.4, 25.4, 22.4, 14.4. HRMS ESI [M+H]$^+$ Calculated for $C_{12}H_{18}NO$ 192.1388, found 192.1390.

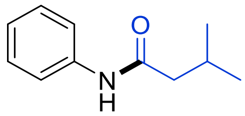

3-methyl-*N*-phenylbutanamide (3h). Following the general procedure, the title compound (12.3 mg) was obtained in 70%. **^{1}H NMR** (400 MHz, Chloroform-*d*) δ 7.59 – 7.46 (m, 2H), 7.41 – 7.28 (m, 2H), 7.10 (t, *J* = 7.4 Hz, 2H), 2.24 – 2.20 (m, 3H), 1.03 – 1.01 (m, 6H). **^{13}C NMR** (126 MHz, DMSO-d_6) δ 171.2, 139.7, 129.1, 123.5, 119.6, 46.1, 26.1, 22.7. HRMS ESI [M+H]$^+$ Calculated for $C_{11}H_{16}NO$ 178.1232, found 178.1235.

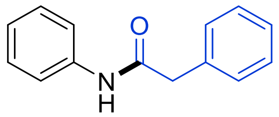

***N*,2-diphenylacetamide (3i).** Following the general procedure, the title compound (11.6 mg) was obtained in 55%. **^{1}H NMR** (400 MHz, DMSO-d_6) δ 10.52 (s, 1H), 8.01 – 7.44 (m, 2H), 7.49 – 7.16 (m, 7H), 7.16 – 6.71 (m, 1H), 3.68 (s, 2H). **^{13}C NMR** (100 MHz, DMSO-d_6) δ 169.7, 139.9, 136.7, 129.6, 129.1, 128.7, 127.0, 123.6, 119.6, 45.8. HRMS ESI $[M+H]^+$ Calculated for $C_{14}H_{14}NO$ 212.1075, found 212.1077.

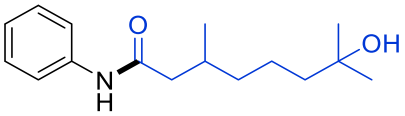

7-hydroxy-3,7-dimethyl-*N*-phenyloctanamide (3j). Following the general procedure, the title compound (20.5 mg) was obtained in 78%. **^{1}H NMR** (400 MHz, Chloroform-*d*) δ 7.51 (d, *J* = 8.0 Hz, 2H), 7.32 (t, *J* = 7.9 Hz, 2H), 7.17 (s, 1H), 7.10 (t, *J* = 7.4 Hz, 1H), 2.25 – 2.01 (m, 3H), 1.99-1.94 (m, 2H), 1.40 – 1.35 (m, 2H), 1.26 – 1.25 (m, 2H), 1.21 (s, 6H), 1.01 (d, *J* = 6.3 Hz, 3H). **^{13}C NMR** (126 MHz, Chloroform-*d*) δ 170.5, 130.9, 129.0, 124.2, 119.8, 68.2, 45.5, 43.8, 37.2, 29.4, 29.1, 21.6, 19.8. HRMS ESI $[M+H]^+$ Calculated for $C_{16}H_{26}NO_2$ 264.1964, found 264.1963.

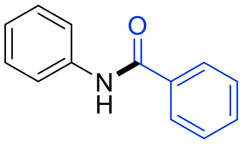

***N*-phenylbenzamide (3k).** Following the general procedure, the title compound (11.8 mg) was obtained in 60%. **^{1}H NMR** (400 MHz, DMSO-d_6) δ 10.29 (s, 1H), 8.01 – 7.97 (m, 2H), 7.85 – 7.81 (m, 2H), 7.62 – 7.51 (m, 3H), 7.39 – 7.34 (m, 2H), 7.14 – 7.09 (m, 1H). **^{13}C NMR** (126 MHz, DMSO-d_6) δ 166.1, 139.7, 135.5, 132.0, 129.1, 128.8, 128.1, 124.1, 120.9. HRMS ESI $[M+H]^+$ Calculated for $C_{13}H_{12}NO$ 198.0919, found 198.0916.

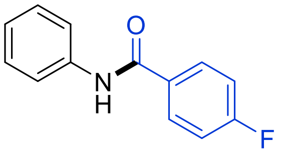

4-fluoro-*N*-phenylbenzamide (3l). Following the general procedure, the title compound (15.2 mg) was obtained in 71%. **^{1}H NMR** (400 MHz, Chloroform-*d*) δ 7.91 – 7.87 (m, 2H), 7.72 (s, 1H), 7.64 – 7.61 (m, 2H), 7.41 – 7.36 (m, 2H), 7.20 – 7.15 (m, 3H). **^{13}C NMR** (100 MHz, Chloroform-*d*) δ 166.1, 163.9 (d, J = 9 Hz),137.2, 129.8, 129.6, 129.0, 124.1, 120.2, 116.6, 115.4. HRMS ESI $[M+H]^+$ Calculated for $C_{13}H_{11}FNO$ 216.0825 found 216.0826.

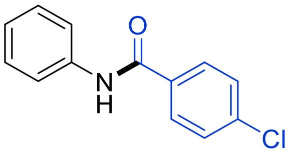

4-chloro-*N*-phenylbenzamide (3m). Following the general procedure, the title compound (14.3 mg) was obtained in 62%. **^{1}H NMR** (400 MHz, DMSO-*d*$_6$) δ 10.33 (s, 1H), 8.00 – 7.97 (m, 2H), 7.78 – 7.75 (m, 2H), 7.72 – 7.60 (m, 2H), 7.37 – 7.33 (m, 2H), 7.13 – 7.09 (m, 1H). **^{13}C NMR** (126 MHz, DMSO-*d*$_6$) δ 164.9, 139.4, 136.9, 134.1, 130.1, 129.1, 128.9, 124.3, 120.9. HRMS ESI $[M+H]^+$ Calculated for $C_{13}H_{11}ClNO$ 232.0529 found 232.0531.

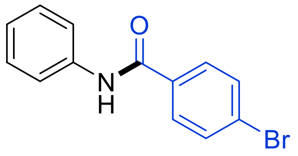

4-bromo-*N*-phenylbenzamide (3n). Following the general procedure, the title compound (14.8 mg) was obtained in 54%. **^{1}H NMR** (400 MHz, Chloroform-*d*) δ 7.76 – 7.73 (m, 3H), 7.65 – 7.61 (m, 4H), 7.41 – 7.36 (m, 2H), 7.19 – 7.15 (m, 1H). **^{13}C NMR** (126 MHz, Chloroform-*d*) δ 164.3, 138.1, 133.0, 131.3, 129.9, 129.0, 125.4, 124.1, 119.7. HRMS ESI $[M+H]^+$ Calculated for $C_{13}H_{11}BrNO$ 276.0024 found 2176.0021.

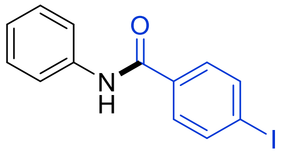

4-iodo-*N*-phenylbenzamide (3o). Following the general procedure, the title compound (6.4 mg) was obtained in 20%. **^{1}H NMR** (400 MHz, DMSO-*d*$_6$) δ 10.29 (s, 1H), 7.92 (d, *J* = 8.5 Hz, 2H), 7.78 – 7.73 (m, 4H), 7.37 – 7.33 (m, 2H), 7.13 – 7.08 (m, 1H). **^{13}C NMR** (126 MHz, DMSO-*d*$_6$) δ 164.9, 141.1, 138.7, 131.6, 128.4, 128.1, 127.2, 123.1, 119.9. HRMS ESI $[M+H]^+$ Calculated for $C_{13}H_{11}INO$ 323.9885 found 323.9887.

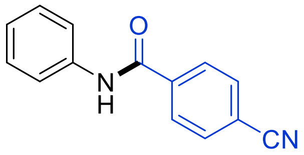

4-cyano-*N*-phenylbenzamide (3p). Following the general procedure, the title compound (13.3 mg) was obtained in 60%. **^{1}H NMR** (400 MHz, Chloroform-*d*) δ 7.98 (d, *J* = 8.4 Hz, 2H), 7.82 – 7.78 (m, 3H), 7.64 – 7.62 (m, 2H), 7.42 – 7.38 (m, 2H), 7.22 – 7.18 (m, 1H). **^{13}C NMR** (126 MHz, DMSO-*d*$_6$) δ 167.5, 139.9, 131.9, 130.1, 129.1, 127.5, 123.9, 120.9, 114.3, 114.1. HRMS ESI $[M+H]^+$ Calculated for $C_{14}H_{11}N_2O$ 223.0871 found 223.0872.

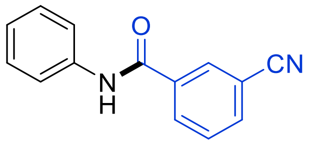

3-cyano-*N*-phenylbenzamide (3q). Following the general procedure, the title compound (9.3 mg) was obtained in 42%. **^{1}H NMR** (400 MHz, DMSO-*d*$_6$) δ 10.58 (s, 1H), 8.46 (d, *J* = 1.9 Hz, 1H), 8.31 (dt, *J* = 7.9, 1.6 Hz, 1H), 8.06 (dd, *J* = 7.8, 1.4 Hz, 1H), 7.84 – 7.81 (m, 2H), 7.75 (t,

J = 7.8 Hz, 1H), 7.37 (t, J = 7.8 Hz, 2H), 7.15 – 7.11 (m, 1H). **^{13}C NMR** (126 MHz, DMSO-d_6) δ 164.1, 139.3, 136.4, 135.4, 133.1, 131.9, 130.3, 129.1, 124.5, 121.0, 118.8, 112.0. HRMS ESI $[M+H]^+$ Calculated for $C_{14}H_{11}N_2O$ 223.0871 found 223.0873.

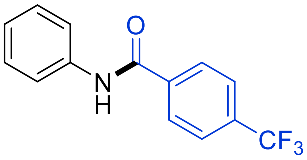

***N*-phenyl-4-(trifluoromethyl)benzamide (3r).** Following the general procedure, the title compound (16.7 mg) was obtained in 63%. **^{1}H NMR** (400 MHz, Chloroform-*d*) δ 7.99 (d, J = 8.1 Hz, 2H), 7.78 – 7.76 (m, 3H), 7.64 (d, J = 7.6 Hz, 2H), 7.40 (dd, J = 8.5, 7.4 Hz, 2H), 7.22 – 7.17 (m, 1H). **^{13}C NMR** (126 MHz, DMSO-d_6) δ 164.9, 139.3, 131.8 (q, J = 18.75 Hz), 129.2, 129.1, 125.9, 124.5, 124.4 (q, J = 270 Hz), 120.9. HRMS ESI $[M+H]^+$ Calculated for $C_{14}H_{11}F_3NO$ 266.0793 found 266.0791.

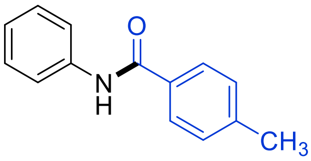

4-methyl-*N*-phenylbenzamide (3s). Following the general procedure, the title compound (9.5 mg) was obtained in 45%. **^{1}H NMR** (400 MHz, Chloroform-*d*) δ 7.79 – 7.76 (m, 2H), 7.64 (d, J = 7.4 Hz, 2H), 7.37 (dd, J = 8.5, 7.4 Hz, 2H), 7.29 (d, J = 7.7 Hz, 2H), 7.17 – 7.12 (m, 1H), 2.43 (s, 3H). **^{13}C NMR** (126 MHz, DMSO-d_6) δ 165.8, 142.0, 139.7, 132.6, 129.37, 129.0, 128.2, 124.0, 120.8, 21.5. HRMS ESI $[M+H]^+$ Calculated for $C_{14}H_{14}NO$ 212.1075 found 212.1079.

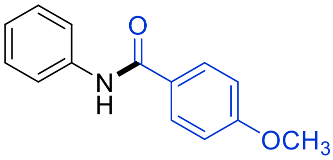

4-methoxy-*N*-phenylbenzamide (3t). Following the general procedure, the title compound (10.7 mg) was obtained in 47%. **^{1}H NMR** (400 MHz, DMSO-d_6) δ 10.31 (s, 1H), 7.93 – 7.89 (m, 2H), 7.78 – 7.73 (m, 4H), 7.37 – 7.33 (m, 2H), 7.13 – 7.09 (m, 1H), 3.34 (s, 3H). **^{13}C NMR** (126 MHz, DMSO-d_6) δ 163.3, 131.8, 130.1, 129.0, 123.9, 123.4, 120.8, 114.3, 114.1, 55.9. HRMS ESI $[M+H]^+$ Calculated for $C_{14}H_{14}NO_2$ 228.1025 found 228.1027.

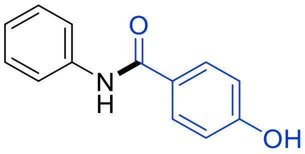

4-hydroxy-*N*-phenylbenzamide (3u). Following the general procedure, the title compound (8.7 mg) was obtained in 41%. **^{1}H NMR** (400 MHz, Chloroform-*d*) δ 8.66 (s, 1H), 7.38 (dt, J

= 8.4, 7.5 Hz, 2H), 7.31 – 7.26 (m, 2H), 7.16 – 7.13 (m, 4H), 6.92 (d, *J* = 8.7 Hz, 1H). **^{13}C NMR** (126 MHz, DMSO-*d*$_6$) δ 165.8, 160.9, 139.5, 129.8, 129.0, 126.6, 124.3, 120.5, 117.5. HRMS ESI [M+H]$^+$ Calculated for $C_{13}H_{12}NO_2$ 214.0868 found 214.0869.

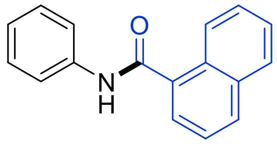

***N*-phenyl-1-naphthamide (3v).** Following the general procedure, the title compound (12.8 mg) was obtained in 52%. **^{1}H NMR** (400 MHz, Chloroform-*d*) δ 7.97 – 7.85 (m, 3H), 7.59 – 7.42 (m, 4H), 7.37 – 7.26 (m, 4H), 7.19 – 7.17 (m, 1H). **^{13}C NMR** (126 MHz, DMSO-*d*$_6$) δ 167.8, 139.9, 135.2, 133.7, 130.6, 130.2, 129.2, 128.8, 127.5, 126.8, 126.0, 125.6, 125.5, 124.2, 120.4. HRMS ESI [M+H]$^+$ Calculated for $C_{17}H_{14}NO$ 248.1075 found 248.1077.

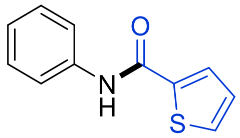

***N*-phenylthiophene-2-carboxamide (3w).** Following the general procedure, the title compound (8.1 mg) was obtained in 40%. **^{1}H NMR** (400 MHz, Chloroform-*d*) δ 7.69 (s, 1H), 7.64 – 7.54 (m, 3H), 7.55 (dd, *J* = 5.0, 1.2 Hz, 1H), 7.39 – 7.34 (m, 2H), 7.17 – 7.12 (m, 2H). **^{13}C NMR** (126 MHz, DMSO-*d*$_6$) δ 160.4, 139.2, 132.3, 129.6, 129.2, 129.1, 128.5, 124.3, 120.9. HRMS ESI [M+H]$^+$ Calculated for $C_{11}H_{10}NOS$ 204.0483 found 204.0479.

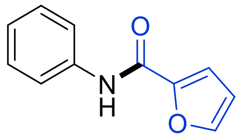

***N*-phenylfuran-2-carboxamide (3x).** Following the general procedure, the title compound (11.2 mg) was obtained in 60%. **^{1}H NMR** (400 MHz, Chloroform-*d*) δ 8.10 (s, 1H), 7.65 (dt, *J* = 7.8, 1.1 Hz, 2H), 7.51 (dd, *J* = 1.7, 0.8 Hz, 1H), 7.39 – 7.34 (m, 2H), 7.24 (dd, *J* = 3.5, 0.8 Hz, 1H), 7.16 – 7.12 (m, 1H), 6.56 (dd, *J* = 3.5, 1.8 Hz, 1H). **^{13}C NMR** (126 MHz, DMSO-*d*$_6$) δ 156.8, 148.1, 146.2, 139.1, 129.2, 124.3, 123.0, 115.3, 112.7. HRMS ESI [M+H]$^+$ Calculated for $C_{11}H_{10}NO_2$ 188.0712 found 188.0715.

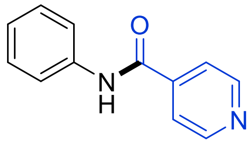

***N*-phenylisonicotinamide (3y).** Following the general procedure, the title compound (8.3 mg) was obtained in 42%. **^{1}H NMR** (400 MHz, Chloroform-*d*) δ 8.80 (s, 1H), 8.77 – 8.75 (m, 2H), 7.86 – 7.84 (m, 2H), 7.77 – 7.75 (m, 2H), 7.38 – 7.34 (m, 2H), 7.17 – 7.13 (m, 1H). **^{13}C NMR** (126 MHz, DMSO-*d*$_6$) δ 164.5, 150.7, 142.4, 139.1, 129.2, 124.7, 122.1, 121.0. HRMS ESI [M+H]$^+$ Calculated for $C_{12}H_{11}N_2O$ 199.0871 found 199.0870.

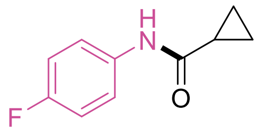

***N*-(4-fluorophenyl)cyclopropanecarboxamide (3z).** Following the general procedure, the title compound (11.1 mg) was obtained in 64%. **^{1}H NMR** (400 MHz, Chloroform-*d*) δ 7.48 – 7.43 (m, 3H), 6.99 (t, *J* = 8.7 Hz, 2H), 1.48 (tt, *J* = 8.0, 4.5 Hz, 1H), 1.10 – 1.06 (m, 2H), 0.84 (dq, *J* = 7.3, 4.0 Hz, 2H). **^{13}C NMR** (126 MHz, DMSO-*d*$_6$) δ 172.0, 159.2, 157.3, 136.3, 121.2 (d, *J* = 7.7 Hz), 115.7 (d, *J* = 22.1 Hz), 15.0, 7.6. **^{19}F NMR** (377 MHz, DMSO-*d*$_6$) δ -119.88. HRMS ESI $[M+H]^+$ Calculated for $C_{10}H_{11}FNO$ 180.0825, found 180.0826.

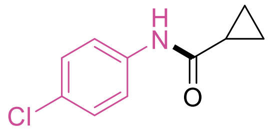

***N*-(4-chlorophenyl)cyclopropanecarboxamide (3aa).** Following the general procedure, the title compound (11.3 mg) was obtained in 58%. **^{1}H NMR** (400 MHz, Chloroform-*d*) δ 7.23 (d, *J* = 7.1 Hz, 2H), 7.07 – 7.03 (m, 2H), 1.27 (tt, *J* = 8.0, 4.5 Hz, 1H), 0.88 – 0.85 (m, 2H), 0.67 – 0.63 (m, 2H). **^{13}C NMR** (100 MHz, DMSO-*d*$_6$) δ 172.5, 139.5, 130.1, 120.9, 114.7, 14.9, 8.0. HRMS ESI $[M+H]^+$ Calculated for $C_{10}H_{11}ClNO$ 196.0529 found 196.0529.

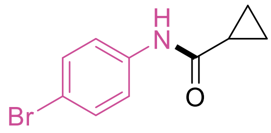

***N*-(4-bromophenyl)cyclopropanecarboxamide (3bb).** Following the general procedure, the title compound (12.1 mg) was obtained in 51%. **^{1}H NMR** (400 MHz, DMSO-*d*$_6$) δ 10.30 (s, 1H), 7.60 – 7.56 (m, 2H), 7.46 – 7.42 (m, 2H), 1.77 (tt, *J* = 7.7, 4.7 Hz, 1H), 0.83 – 0.78 (m, 4H). **^{13}C NMR** (126 MHz, DMSO-*d*$_6$) δ 172.6, 139.3, 132.1, 121.6, 115.2, 15.3, 8.0. HRMS ESI $[M+H]^+$ Calculated for $C_{10}H_{11}BrNO$ 240.0024 found 240.0028.

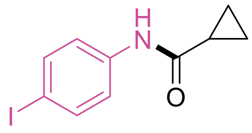

***N*-(4-iodophenyl)cyclopropanecarboxamide (3cc).** Following the general procedure, the title compound (5.2 mg) was obtained in 18%. **^{1}H NMR** (400 MHz, Chloroform-*d*) δ 7.77 (d, *J* = 8.6 Hz, 1H), 7.61 (d, *J* = 8.7 Hz, 1H), 7.31 (t, *J* = 8.3 Hz, 2H), 7.24 (s, 1H), 1.48 (t, *J* = 4.3 Hz, 1H), 1.17 – 1.07 (m, 2H), 0.86 (ddt, *J* = 7.4, 6.4, 3.7 Hz, 2H). **^{13}C NMR** (126 MHz, DMSO-*d*$_6$) δ 172.3, 139.6, 137.8, 121.6, 86.7, 15.1, 7.8. HRMS ESI $[M+H]^+$ Calculated for $C_{10}H_{11}INO$ 287.9885 found 287.9886.

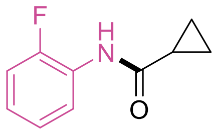

***N*-(2-fluorophenyl)cyclopropanecarboxamide (3dd).** Following the general procedure, the title compound (8.4 mg) was obtained in 47%. **^{1}H NMR** (400 MHz, Chloroform-*d*) δ 8.70 (s, 1H), 7.53 (td, *J* = 7.6, 1.8 Hz, 1H), 7.49 – 7.36 (m, 1H), 7.33 – 7.13 (m, 2H), 1.44 (s, 1H), 1.14 – 1.11 (m, 2H), 0.83 – 0.78 (m, 2H), **^{13}C NMR** (126 MHz, DMSO-*d*6) δ 173.7, 128.8 (d, J = 3.5 Hz), 127.5 (d, J = 11.2 Hz), 126.7, 120.3, 120.1, 116.6, 15.5, 9.0. HRMS ESI $[M+H]^+$ Calculated for $C_{10}H_{11}FNO$ 180.0825, found 180.0827.

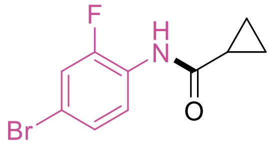

***N*-(4-bromo-2-fluorophenyl)cyclopropanecarboxamide (3ee).** Following the general procedure, the title compound (13.8 mg) was obtained in 54%. **^{1}H NMR** (400 MHz, DMSO-*d*6) δ 10.08 (s, 1H), 7.89 (t, *J* = 8.6 Hz, 1H), 7.58 (dd, *J* = 10.6, 2.2 Hz, 1H), 7.34 (ddd, *J* = 8.8, 2.3, 1.2 Hz, 1H), 2.03 – 1.96 (m, 1H), 0.81 – 0.78 (m, 4H). **^{13}C NMR** (126 MHz, DMSO-*d*6) δ 172.7, 127.8 (d, *J* = 3.5 Hz), 126.4, 125.7, 124.7, 119.3, 119.1, 14.5, 7.9 (d, *J* = 24.8 Hz). **^{19}F NMR** (377 MHz, DMSO-*d*6) δ -121.86. HRMS ESI $[M+H]^+$ Calculated for $C_{10}H_{10}BrFNO$ 257.9930, found 257.9931.

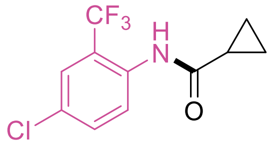

***N*-(4-chloro-2-(trifluoromethyl)phenyl)cyclopropanecarboxamide (3ff).** Following the general procedure, the title compound (14.2 mg) was obtained in 54%. **^{1}H NMR** (400 MHz, Chloroform-*d*) δ 8.21 (s, 1H), 7.58 (d, *J* = 2.6 Hz, 2H), 7.49 (dd, *J* = 8.9, 2.5 Hz, 1H), 1.52 (tt, *J* = 7.8, 4.5 Hz, 1H), 1.13 – 1.09 (m, 2H), 0.92 (dt, *J* = 7.9, 3.5 Hz, 2H). **^{13}C NMR** (126 MHz, DMSO-*d*6) δ 173.4, 135.1, 133.3, 132.5, 131.1, 126.7 (q, *J* = 5.4 Hz), 124.3, 122.1, 14.2, 7.8. **^{19}F NMR** (377 MHz, DMSO-*d*6) δ -59.69. HRMS ESI $[M+H]^+$ Calculated for $C_{11}H_{10}ClF_3NO$ 264.0403, found 264.0401.

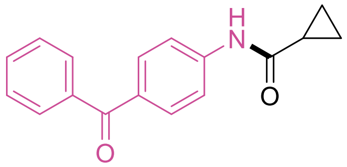

***N*-(4-benzoylphenyl)cyclopropanecarboxamide (3gg).** Following the general procedure, the title compound (16.4 mg) was obtained in 62%. **^{1}H NMR** (400 MHz, Chloroform-*d*) δ 7.83 – 7.80 (m, 2H), 7.78 – 7.75 (m, 2H), 7.68 (s, 1H), 7.66 – 7.63 (m, 2H), 7.60 – 7.56 (m, 1H), 7.50

– 7.45 (m, 2H), 1.55 (tt, *J* = 7.8, 4.5 Hz, 1H), 1.15 – 1.11 (m, 2H), 0.92 – 0.88 (m, 2H). **^{13}C NMR** (126 MHz, DMSO-d_6) δ 195.0, 172.8, 144.0, 138.1, 132.7, 131.7, 131.5, 129.8, 129.0, 118.7, 15.2, 8.1. HRMS ESI [M+H]$^+$ Calculated for $C_{17}H_{16}NO_2$ 266.1181, found 266.1178.

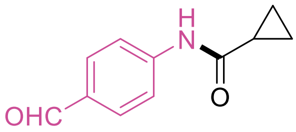

***N*-(4-formylphenyl)cyclopropanecarboxamide (3hh).** Following the general procedure, the title compound (8.5 mg) was obtained in 45%. **^{1}H NMR** (400 MHz, Chloroform-*d*) δ 9.91 (s, 1H), 7.86– 7.83 (m, 2H), 7.72 – 7.68 (m, 2H), 7.64 (s, 1H), 1.55 (tt, *J* = 7.8, 4.5 Hz, 1H), 1.15 – 1.11 (m, 2H), 0.92 (dt, *J* = 7.9, 3.5 Hz, 2H). **^{13}C NMR** (126 MHz, DMSO-d_6) δ 192.0, 172.9, 145.3, 131.5, 131.3, 119.1, 15.3, 8.2. HRMS ESI [M+H]$^+$ Calculated for $C_{11}H_{12}NO_2$ 190.0868, found 190.0869.

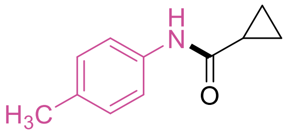

***N*-(p-tolyl)cyclopropanecarboxamide (3ii).** Following the general procedure, the title compound (13.3 mg) was obtained in 76%. **^{1}H NMR** (400 MHz, Chloroform-*d*) δ 7.38 (d, *J* = 8.3 Hz, 2H), 7.11 (d, *J* = 8.0 Hz, 2H), 2.30(s, 3H),1.47 (d, *J* = 4.2 Hz, 1H), 1.09 – 1.05 (m, 2H), 0.82 (dd, *J* = 7.7, 3.2 Hz, 2H). **^{13}C NMR** (126 MHz, DMSO-d_6) δ 172.8, 137.3, 132.3, 128.8, 119.8, 20.9, 15.5, 7.9. HRMS ESI [M+H]$^+$ Calculated for $C_{11}H_{14}NO$ 176.1075, found 176.1079.

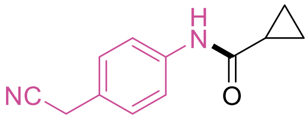

***N*-(4-(cyanomethyl)phenyl)cyclopropanecarboxamide (3jj).** Following the general procedure, the title compound (12.8 mg) was obtained in 64%. **^{1}H NMR** (400 MHz, Chloroform-*d*) δ 7.47 (d, *J* = 8.2 Hz, 2H), 7.35 (s, 1H), 7.21 (s, 1H), 7.19 (s, 1H), 3.64 (s, 2H), 1.43 (dq, *J* = 8.1, 4.5, 4.1 Hz, 1H), 1.04 – 1.01 (m, 2H), 0.80 (dt, *J* = 7.9, 3.5 Hz, 2H). **^{13}C NMR** (126 MHz, DMSO-d_6) δ 172.2, 139.2, 128.9, 125.8, 119.9, 119.8, 22.3, 15.0, 7.7. HRMS ESI [M+H]$^+$ Calculated for $C_{12}H_{13}N_2O$ 201.1028, found 201.1029.

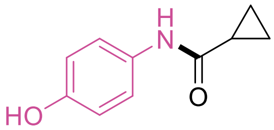

***N*-(4-hydroxyphenyl)cyclopropanecarboxamide (3kk).** Following the general procedure, the title compound (10.8 mg) was obtained in 61%. **^{1}H NMR** (400 MHz, Chloroform-*d*) δ 7.32 (d, *J* = 8.5 Hz, 2H), 7.27 (s, 1H), 6.77 (d, *J* = 8.6 Hz, 2H), 1.48 – 1.42 (m, 1H), 1.09 – 1.05 (m, 2H), 0.83 (dt, *J* = 7.2, 3.7 Hz, 2H). **^{13}C NMR** (126 MHz, DMSO-d_6) δ 170.3, 153.5, 131.6,

121.2, 115.5, 14.8, 7.2. HRMS ESI $[M+H]^+$ Calculated for $C_{10}H_{12}NO_2$ 178.0868, found 178.0869.

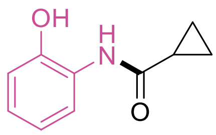

***N*-(2-hydroxyphenyl)cyclopropanecarboxamide (3ll).** Following the general procedure, the title compound (10.8 mg) was obtained in 61%. **^{1}H NMR** (400 MHz, Chloroform-*d*) δ 8.99 (s, 1H), 7.63 (s, 1H), 7.12 (ddd, *J* = 8.1, 7.2, 1.6 Hz, 1H), 7.00 (ddd, *J* = 17.4, 8.0, 1.6 Hz, 2H), 6.85 (ddd, *J* = 7.9, 7.2, 1.5 Hz, 1H), 1.66 – 1.61 (m, 1H), 1.17 – 1.14 (m, 2H), 0.95 (dt, *J* = 7.9, 3.4 Hz, 2H). **^{13}C NMR** (126 MHz, DMSO-*d*$_6$) δ 170.8, 127.0, 125.0, 122.9, 122.8, 119.4, 116.4, 14.6, 7.8. HRMS ESI $[M+H]^+$ Calculated for $C_{10}H_{12}NO_2$ 178.0868, found 178.0865.

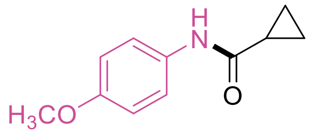

***N*-(4-methoxyphenyl)cyclopropanecarboxamide (3mm).** Following the general procedure, the title compound (9.5 mg) was obtained in 50%. **^{1}H NMR** (400 MHz, Chloroform-*d*) δ 7.41 (d, *J* = 8.9 Hz, 2H), 7.30 (s, 1H), 6.85 (d, *J* = 8.8 Hz, 2H), 3.78 (s, 3H), 1.46 (td, *J* = 8.0, 4.0 Hz, 1H), 1.09 – 1.05 (m, 2H), 0.82 (dt, *J* = 7.3, 3.7 Hz, 2H). **^{13}C NMR** (126 MHz, DMSO-*d*$_6$) δ 172.5, 156.7, 133.6, 122.5, 114.6, 55.3, 16.6, 8.6. HRMS ESI $[M+H]^+$ Calculated for $C_{11}H_{14}NO_2$ 192.1025, found 192.1024.

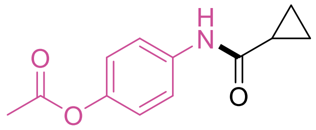

4-(cyclopropanecarboxamido)phenyl acetate (3nn). Following the general procedure, the title compound (14.6 mg) was obtained in 67%. **^{1}H NMR** (400 MHz, Chloroform-*d*) δ 7.52 – 7.50 (m, 1H), 7.49 – 7.47 (m, 2H), 7.04 – 7.01 (m, 2H), 2.28 (s, 3H), 1.49 (d, *J* = 4.3 Hz, 1H), 1.09 – 1.06 (m, 2H), 0.86 – 0.82 (m, 2H). **^{13}C NMR** (126 MHz, DMSO-*d*$_6$) δ 172.0, 169.8, 146.1, 137.4, 122.4, 120.3, 21.3, 15.0, 7.6. HRMS ESI $[M+H]^+$ Calculated for $C_{12}H_{14}NO_3$ 220.0974, found 220.0973.

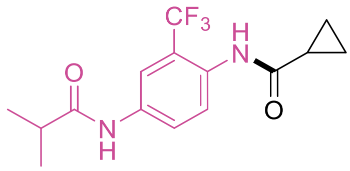

***N*-(4-isobutyramido-2-(trifluoromethyl)phenyl)cyclopropanecarboxamide (3oo).** Following the general procedure, the title compound (15.7 mg) was obtained in 50%.

^{1}H NMR (400 MHz, DMSO-d_6) δ 10.14 (s, 1H), 9.66 (s, 1H), 8.09 (d, *J* = 2.4 Hz, 1H), 7.77 (dd, *J* = 8.8, 2.5 Hz, 1H), 7.37 (d, *J* = 8.7 Hz, 1H), 2.59 (p, *J* = 6.8 Hz, 1H), 1.86 (s, 1H), 1.11 (d, *J* = 6.8 Hz, 6H), 0.78 – 0.75 (m, 4H). **^{13}C NMR** (126 MHz, DMSO-d_6) δ 176.2, 173.4, 138.2, 131.6, 130.6, 127.9, 124.0 (q, *J* = 274.68 Hz), 123.3, 116.7 (d, *J* = 5.6 Hz), 35.5, 19.9, 14.2, 7.5. **^{19}F NMR** (377 MHz, DMSO-d_6) δ -59.62. HRMS ESI $[M+H]^+$ Calculated for $C_{15}H_{18}F_3N_2O_2$ 315.1320, found 315.1322.

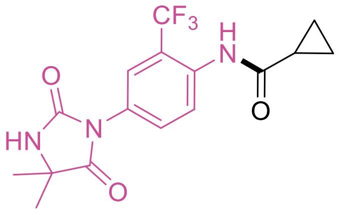

***N*-(4-(4,4-dimethyl-2,5-dioxoimidazolidin-1-yl)-2-(trifluoromethyl)phenyl)cyclopropanecarboxamide (3pp).** Following the general procedure, the title compound (23.6 mg) was obtained in 67%. **^{1}H NMR** (400 MHz, DMSO-d_6) δ 9.85 (s, 1H), 8.64 (s, 1H), 7.81 (d, *J* = 2.3 Hz, 1H), 7.68 (dd, *J* = 8.6, 2.4 Hz, 1H), 7.59 (d, *J* = 8.6 Hz, 1H), 1.92 (dd, *J* = 8.9, 3.8 Hz, 1H), 1.42 (s, 6H), 0.80 (tt, *J* = 4.9, 2.4 Hz, 4H). **^{13}C NMR** (126 MHz, DMSO-d_6) δ 176.7, 173.4, 154.3, 135.3, 131.4, 131.1, 130.6, 125.4, 125.4, 125.1 (q, *J* = 5.7 Hz), 58.4, 25.1, 14.2, 7.7. **^{19}F NMR** (377 MHz, DMSO-d_6) δ -59.49. HRMS ESI $[M+H]^+$ Calculated for $C_{16}H_{17}F_3N_3O_3$ 356.1222, found 356.1224.

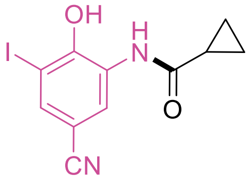

***N*-(5-cyano-2-hydroxy-3-iodophenyl)cyclopropanecarboxamide (3qq).** Following the general procedure, the title compound (9.8 mg) was obtained in 30%. **^{1}H NMR** (400 MHz, Chloroform-*d*) δ 8.03 (s, 1H), 7.82 (d, *J* = 1.9 Hz, 1H), 7.69 (d, *J* = 2.0 Hz, 1H), 1.66 (tt, *J* = 7.9, 4.5 Hz, 1H), 1.18 – 1.15 (m, 2H), 1.02 – 0.98 (m, 2H). **^{13}C NMR** (126 MHz, DMSO-d_6) δ 174.4, 153.3, 138.8, 127.3, 118.0, 104.0, 88.8, 14.9, 8.6. HRMS ESI $[M+H]^+$ Calculated for $C_{11}H_{10}IN_2O_2$ 328.9787, found 328.9788.

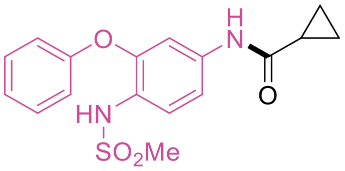

***N*-(4-(methylsulfonamido)-3-phenoxyphenyl)cyclopropanecarboxamide (3rr).** Following the general procedure, the title compound (21.4 mg) was obtained in 62%. **^{1}H NMR** (400 MHz, DMSO-d_6) δ 10.24 (s, 1H), 9.19 (s, 1H), 7.43 – 7.39 (m, 2H), 7.29 – 7.26 (m, 3H), 7.20 – 7.15 (m, 1H), 7.08 – 7.04 (m, 2H), 2.94 (s, 3H), 1.68 (td, *J* = 5.3, 4.5, 2.4 Hz, 1H), 0.80 – 0.72 (m, 4H). **^{13}C NMR** (126 MHz, DMSO-d_6) δ 172.2, 156.5, 151.7, 138.9, 130.6, 128.5, 124.4, 122.8,

119.8, 114.3, 109.1, 40.9, 15.0, 7.7. HRMS ESI $[M+H]^+$ Calculated for $C_{17}H_{19}N_2O_4S$ 347.1066, found 347.1069.

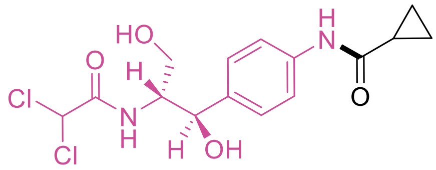

***N*-(4-((1*R*,2*R*)-2-(2,2-dichloroacetamido)-1,3-dihydroxypropyl)phenyl)cyclopropanecarboxamide (3ss).** Following the general procedure, the title compound (13.3 mg) was obtained in 37%. **^{1}H NMR** (400 MHz, DMSO-d_6) δ 10.13 (s, 1H), 8.40 (d, *J* = 9.4 Hz, 1H), 7.52 – 7.49 (m, 2H), 7.21 – 7.19 (m, 2H), 6.61 (s, 1H), 4.97 (d, *J* = 1.9 Hz, 1H), 4.44 (d, *J* = 5.8 Hz, 1H), 4.04 (ddd, *J* = 11.8, 5.5, 2.4 Hz, 1H), 3.85 (dd, *J* = 11.6, 1.6 Hz, 1H), 1.78 – 1.72 (m, 1H), 1.22 (dd, *J* = 7.9, 1.9 Hz, 2H), 0.53 – 0.45 (m, 4H). **^{13}C NMR** (126 MHz, DMSO-d_6) δ 172.0, 163.6, 138.9, 126.6, 118.7, 104.5, 78.6, 70.3, 66.7, 47.9, 15.1, 7.6. HRMS ESI $[M+H]^+$ Calculated for $C_{15}H_{19}Cl_2N_2O_4$ 361.0722, found 361.0724.

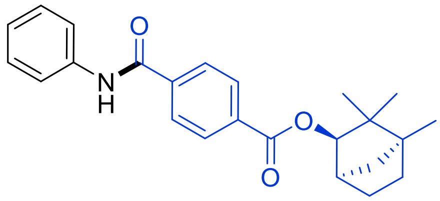

(1*R*,2*R*,4*S*)-3,3,4-trimethylbicyclo[2.2.1]heptan-2-yl 4-(phenylcarbamoyl)benzoate (3tt). Following the general procedure, the title compound (16.5 mg) was obtained in 44%. **^{1}H NMR** (400 MHz, Chloroform-*d*) δ 8.17 (d, *J* = 8.4 Hz, 2H), 7.95 (d, *J* = 8.4 Hz, 2H), 7.84 (s, 1H), 7.66 – 7.64 (m, 2H), 7.39 (dd, *J* = 8.5, 7.4 Hz, 2H), 7.20 – 7.16 (m, 1H), 1.97 – 1.90 (m, 2H), 1.83 – 1.77 (m, 3H), 1.68 (dd, *J* = 10.3, 2.1 Hz, 4H), 1.25 (s, 6H), 1.13 (s, 2H), 0.85 (3, 1H). **^{13}C NMR** (126 MHz, DMSO-d_6) δ 166.0, 165.3, 139.7, 139.4, 132.7, 129.6, 129.2, 128.6, 124.4, 120.9, 86.8, 48.6, 48.3, 41.3, 30.0, 26.9, 26.0, 20.6, 19.7. HRMS ESI $[M+H]^+$ Calculated for $C_{24}H_{28}NO_3$ 378.2069, found 378.2073.

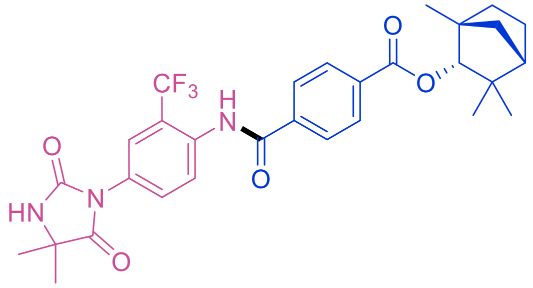

(1*R*,2*R*,4*S*)-1,3,3-trimethylbicyclo[2.2.1]heptan-2-yl 4-((4-(4,4-dimethyl-2,5-dioxoimidazolidin-1-yl)-2-(trifluoromethyl)phenyl)carbamoyl)benzoate (3uu). Following the general procedure, the title compound (23.4 mg) was obtained in 41%. **^{1}H NMR** (400 MHz, DMSO-d_6) δ 10.44 (s, 1H), 8.68 (s, 1H), 8.16 (d, *J* = 8.5 Hz, 2H), 8.09 (d, *J* = 8.4 Hz, 2H), 7.91 (d, *J* = 2.3 Hz, 1H), 7.79 (dd, *J* = 8.4, 2.7 Hz, 1H), 7.68 (d, *J* = 8.5 Hz, 1H), 4.57 (s, 1H), 1.78 – 1.62 (m, 4H), 1.44 (s, 6H), 1.38 – 1.32 (m, 2H), 1.15 (s, 3H), 1.09 (s, 3H), 0.80 (s, 2H), 0.80

– 0.69 (m, 2H). **^{13}C NMR** (126 MHz, DMSO-*d*$_6$) δ 176.7, 166.3, 166.0, 138.4, 135.1, 133.1, 132.3, 131.8, 131.7, 129.8, 128.7, 127.2 (q, *J* = 29.9 Hz), 125.4 (q, *J* = 5.04 Hz), 123.6 (d, *J* = 273.3 Hz), 86.9, 58.5, 48.6, 48.3, 41.3, 30.0, 26.9, 26.0, 25.2, 20.6, 19.7. **^{19}F NMR** (377 MHz, DMSO-*d*$_6$) δ -59.64. HRMS ESI [M+H]$^+$ Calculated for $C_{30}H_{33}F_3N_3O_5$ 572.2372, found 572.2369.

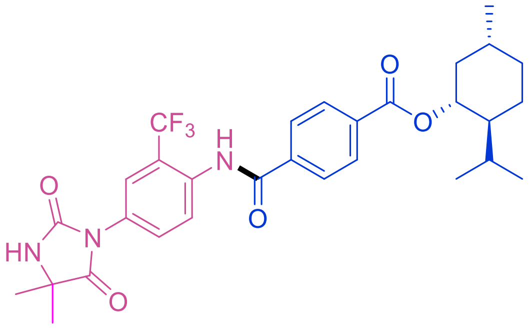

(1*R*,2*S*,5*R*)-2-isopropyl-5-methylcyclohexyl 4-((4-(4,4-dimethyl-2,5-dioxoimidazolidin-1-yl)-2-(trifluoromethyl)phenyl)carbamoyl)benzoate (3vv). Following the general procedure, the title compound (21.7 mg) was obtained in 38%. **^{1}H NMR** (400 MHz, DMSO-*d*$_6$) δ 10.45 (s, 1H), 8.69 (s, 1H), 8.09 (d, *J* = 4.7 Hz, 3H), 7.91 (d, *J* = 2.4 Hz, 1H), 7.80 – 7.73 (m, 2H), 7.67 (d, *J* = 8.5 Hz, 1H), 4.78 – 4.76 (m, 1H), 1.71 – 1.59 (m, 4H), 1.54 (p, *J* = 11.6 Hz, 4H), 1.43 (s, 6H), 1.36 (d, *J* = 9.7 Hz, 4H), 0.77 – 0.68 (m, 6H). **^{13}C NMR** (126 MHz, DMSO-*d*$_6$) δ 176.7, 166.3, 165.1, 154.3, 138.3, 135.1, 133.3, 132.3, 131.9, 131.7, 129.8, 129.8 (d, *J* = 61.4 Hz), 127.2 (q, *J* = 29.9 Hz), 125.4 (d, *J* = 5.4 Hz), 122.1 (d, *J* = 114.6 Hz), 75.2, 58.5, 47.1, 41.0, 34.2, 31.4, 26.8, 25.2, 23.8, 22.4, 21.0, 17.0. **^{19}F NMR** (377 MHz, DMSO-*d*$_6$) δ -59.63. HRMS ESI [M+H]$^+$ Calculated for $C_{30}H_{35}F_3N_3O_5$ 574.2529, found 574.2533.

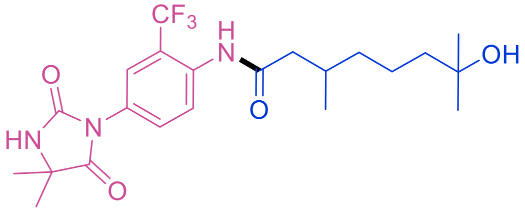

***N*-(4-(4,4-dimethyl-2,5-dioxoimidazolidin-1-yl)-2-(trifluoromethyl)phenyl)-7-hydroxy-3,7-dimethyloctanamide (3ww).** Following the general procedure, the title compound (23.7 mg) was obtained in 52%. **^{1}H NMR** (400 MHz, DMSO-*d*$_6$) δ 9.60 (s, 1H), 8.64 (s, 1H), 7.81 (d, *J* = 2.4 Hz, 1H), 7.69 (dd, *J* = 8.6, 2.4 Hz, 1H), 7.56 (d, *J* = 8.6 Hz, 1H), 2.36 – 2.31 (m, 1H), 2.22 – 2.14 (m, 1H), 1.42 (s, 6H), 1.38 (s, 3H), 1.07 – 1.01 (m, 11H), 0.93 (d, *J* = 6.6 Hz, 3H). **^{13}C NMR** (101 MHz, DMSO-*d*$_6$) δ 176.7, 172.3, 154.3, 135.3 (d, *J* = 1.60 Hz), 131.4 (d, *J* = 7.62 Hz), 128.4, 125.5 (q, *J* = 29.66 Hz), 125.1 (q, *J* = 6.6 Hz), 123.6 (q, *J* = 272.27 Hz), 113.9, 69.2, 58.4, 44.3, 37.4, 30.7, 29.8, 29.7, 25.1, 21.7, 19.8. **^{19}F NMR** (377 MHz, DMSO-*d*$_6$) δ -59.48. HRMS ESI [M+H]$^+$ Calculated for $C_{22}H_{31}F_3N_3O_4$ 458.2267, found 458.2268.

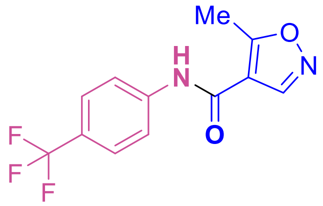

5-methyl-*N*-(4-(trifluoromethyl)phenyl)isoxazole-4-carboxamide (3xx). Following the general procedure, the title compound (16.2 mg) was obtained in 60%. **^{1}H NMR** (400 MHz, Chloroform-*d*) δ 8.48 (s, 1H), 7.71 (d, *J* = 8.6 Hz, 2H), 7.63 (d, *J* = 8.5 Hz, 2H), 7.48 (s, 1H), 2.79 (s, 1H). **^{13}C NMR** (126 MHz, DMSO-*d*$_6$) δ 168.1, 162.7, 150.8, 138.6, 126.6 (q, J = 4.1 Hz), 125.2 (q, J = 32.0 Hz), 123.9 (q, J = 268.1 Hz), 120.7 (q, J = 2.0 Hz), 115.1, 12.2. HRMS ESI $[M+H]^+$ Calculated for $C_{12}H_{10}F_3N_2O_2$ 271.0694, found 271.0697.

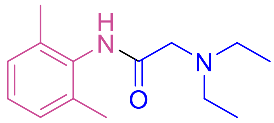

2-(diethylamino)-*N*-(2,6-dimethylphenyl)acetamide (3yy). Following the general procedure, the title compound (10.5 mg) was obtained in 45%. **^{1}H NMR** (400 MHz, Chloroform-*d*) δ 8.91 (s, 1H), 7.09-7.08 (m, 3H), 3.22 (s, 2H), 2.69 (q, J = 7.1 Hz, 4H), 2.23 (s, 6H), 1.14 (t, J = 7.1 Hz, 6H).**^{13}C NMR** (126 MHz, DMSO-*d*$_6$) δ 169.8, 135.7, 135.6, 128.1, 126.7, 57.5, 48.7, 18.7, 12.7. HRMS ESI $[M+H]^+$ Calculated for $C_{14}H_{23}N_2O$ 235.1810, found 235.1809.

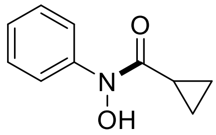

***N*-hydroxy-*N*-phenylcyclopropanecarboxamide (6).** **^{1}H NMR** (400 MHz, Chloroform-*d*) δ 7.47 (d, *J* = 7.0 Hz, 2H), 7.39 (t, *J* = 7.7 Hz, 2H), 7.31 – 7.22 (m, 1H), 1.63 – 1.48 (m, 1H), 1.06 (dt, *J* = 6.8, 3.5 Hz, 2H), 0.77 (dt, *J* = 8.0, 3.4 Hz, 2H). **^{13}C NMR** (100 MHz, Chloroform-*d*) δ 179.5, 138.9, 129.3, 128.4, 126.1, 11.4, 8.5. HRMS ESI $[M+H]^+$ Calculated for $C_{10}H_{12}NO_2$ 178.0868, found 178.0868.

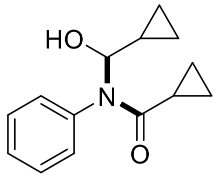

***N*-(cyclopropyl(hydroxy)methyl)-*N*-phenylcyclopropanecarboxamide**. **^{1}H NMR** (400 MHz, Chloroform-*d*) δ 7.18 – 7.14 (m, 2H), 6.73 (tt, *J* = 7.3, 1.1 Hz, 1H), 6.61 – 6.59 (m, 2H), 3.77 (d, *J* = 7.1 Hz, 1H), 2.23 (tt, *J* = 7.8, 4.5 Hz, 1H), 1.27 – 1.23 (m, 2H), 1.09 – 1.07 (m, 2H), 0.96 – 0.92 (m, 2H), 0.60 – 0.51 (m, 3H), 0.34 – 0.32 (m, 1H). **^{13}C NMR** (126 MHz,

Chloroform-*d*) δ 210.5, 147.2, 129.2, 118.1, 113.5, 66.0, 17.8, 13.1, 11.5, 11.5, 3.2, 2.3. HRMS ESI $[M+H]^+$ Calculated for $C_{14}H_{18}NO_2$ 232.1338, found 232.1337.

VII. NMR Spectra

Supplementary Figure 18a | ^{1}H NMR (400 MHz, Chloroform-*d*) of *N*-phenylcyclopropanecarboxamide (**3a**)

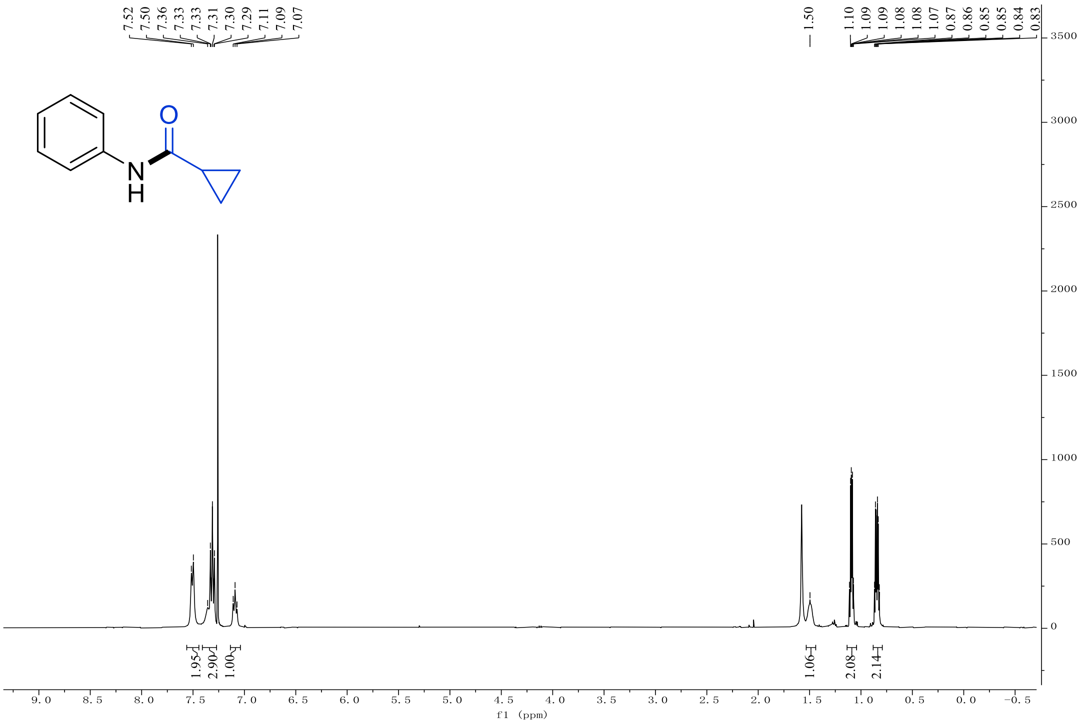

Supplementary Figure 18b | ^{13}C NMR (100 MHz, DMSO-d_6) of *N*-phenylcyclopropanecarboxamide (**3a**)

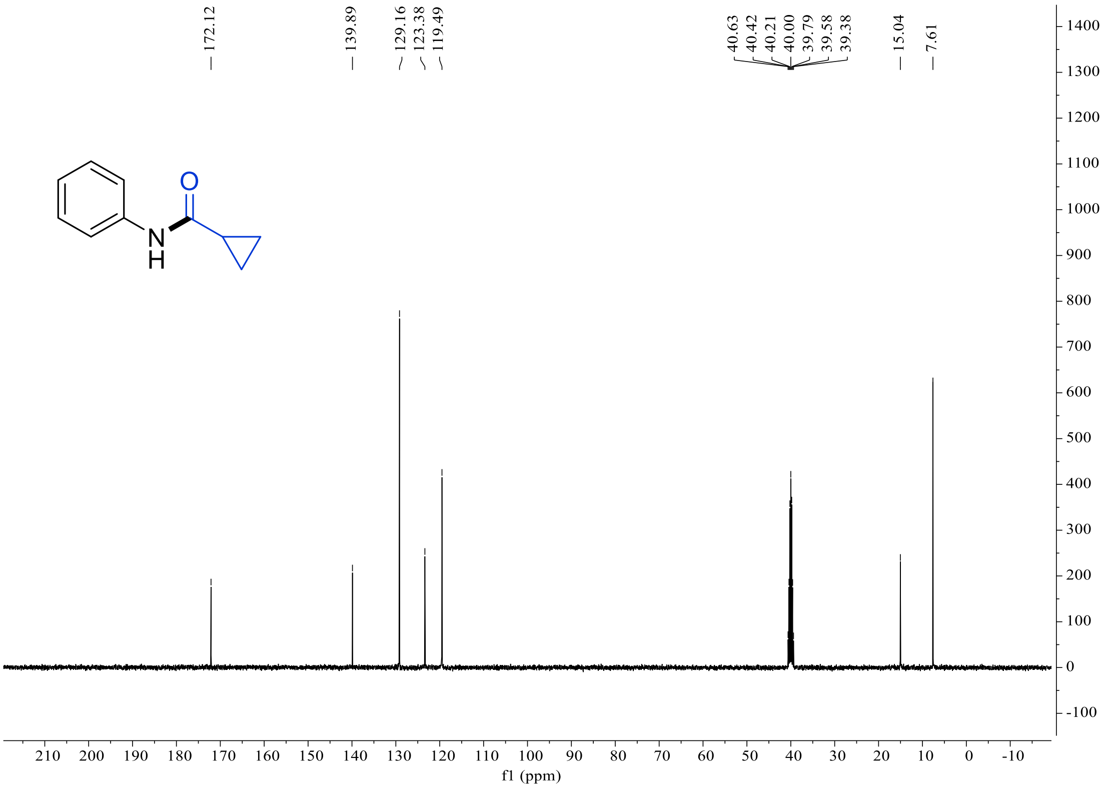

Supplementary Figure 19a | ^{1}H NMR (400 MHz, Chloroform-*d*) of *N*-phenylisobutyramide (**3b**).

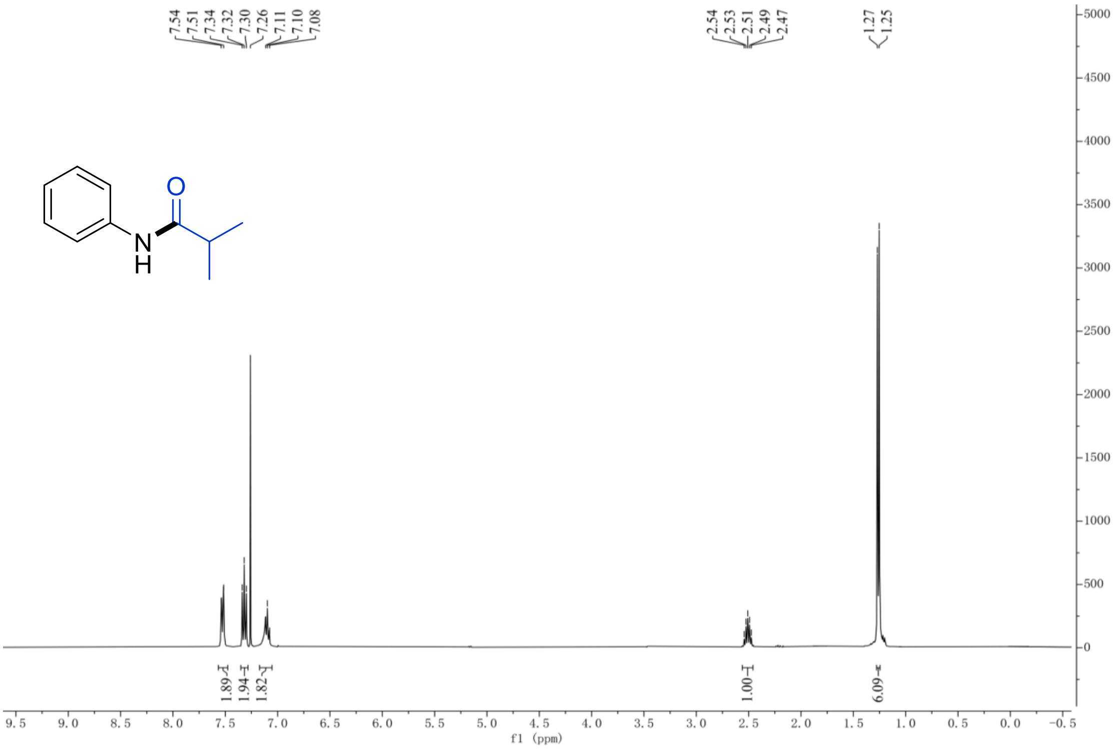

Supplementary Figure 19b | ^{13}C NMR (100 MHz, Chloroform-*d*) of *N*-phenylisobutyramide (**3b**).

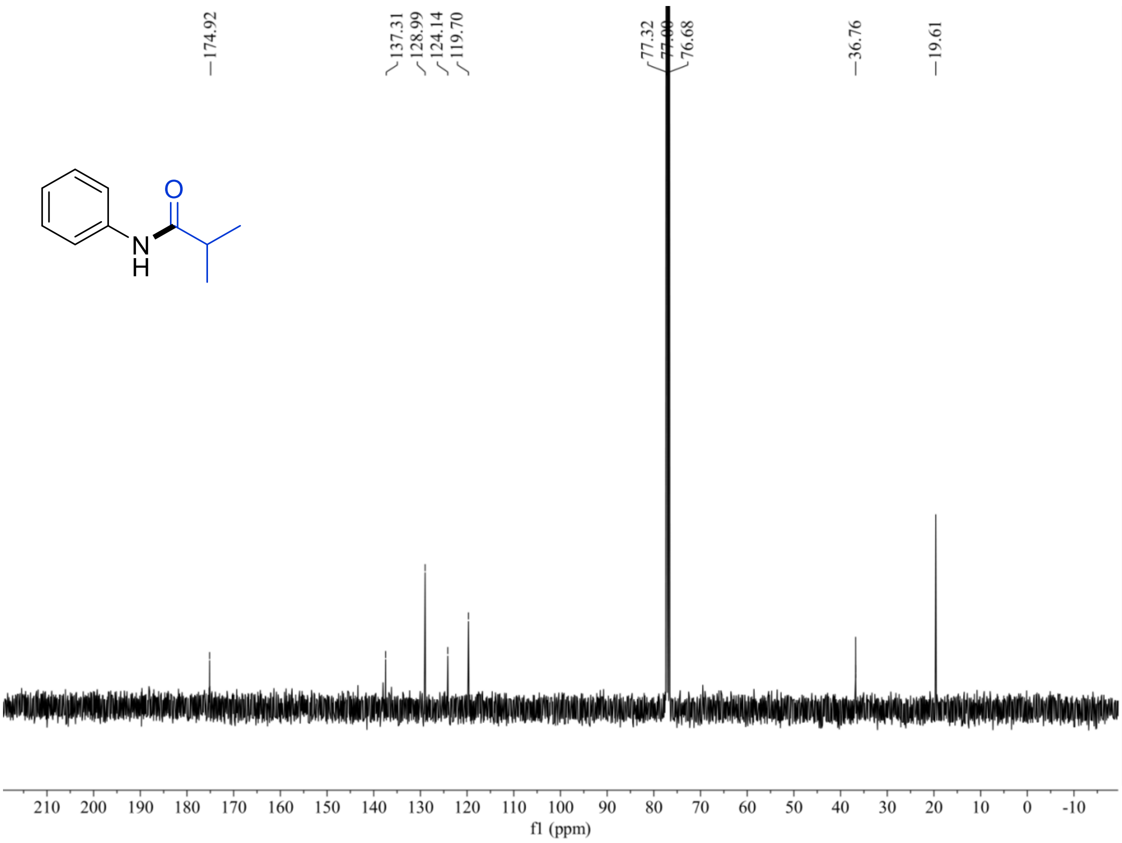

Supplementary Figure 20a | ¹H NMR (400 MHz, DMSO-d_6) of *N*-phenylisobutyramide (**3c**).

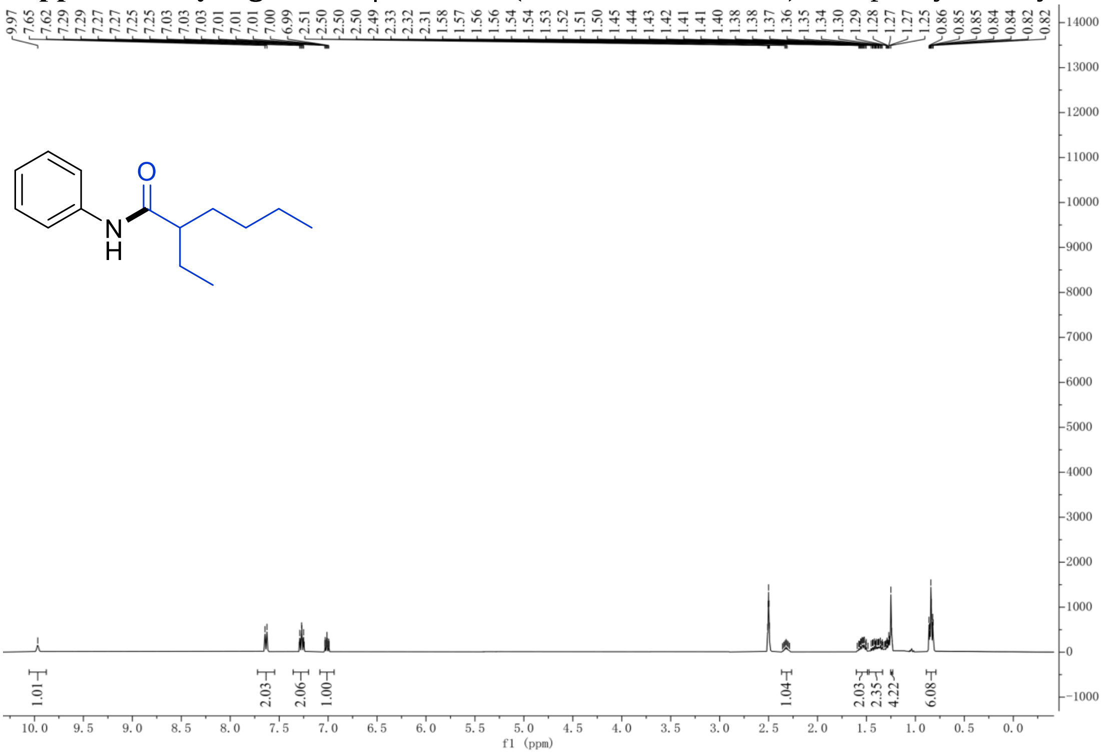

Supplementary Figure 20b | ¹³C NMR (100 MHz, DMSO-d_6) of *N*-phenylisobutyramide (**3c**).

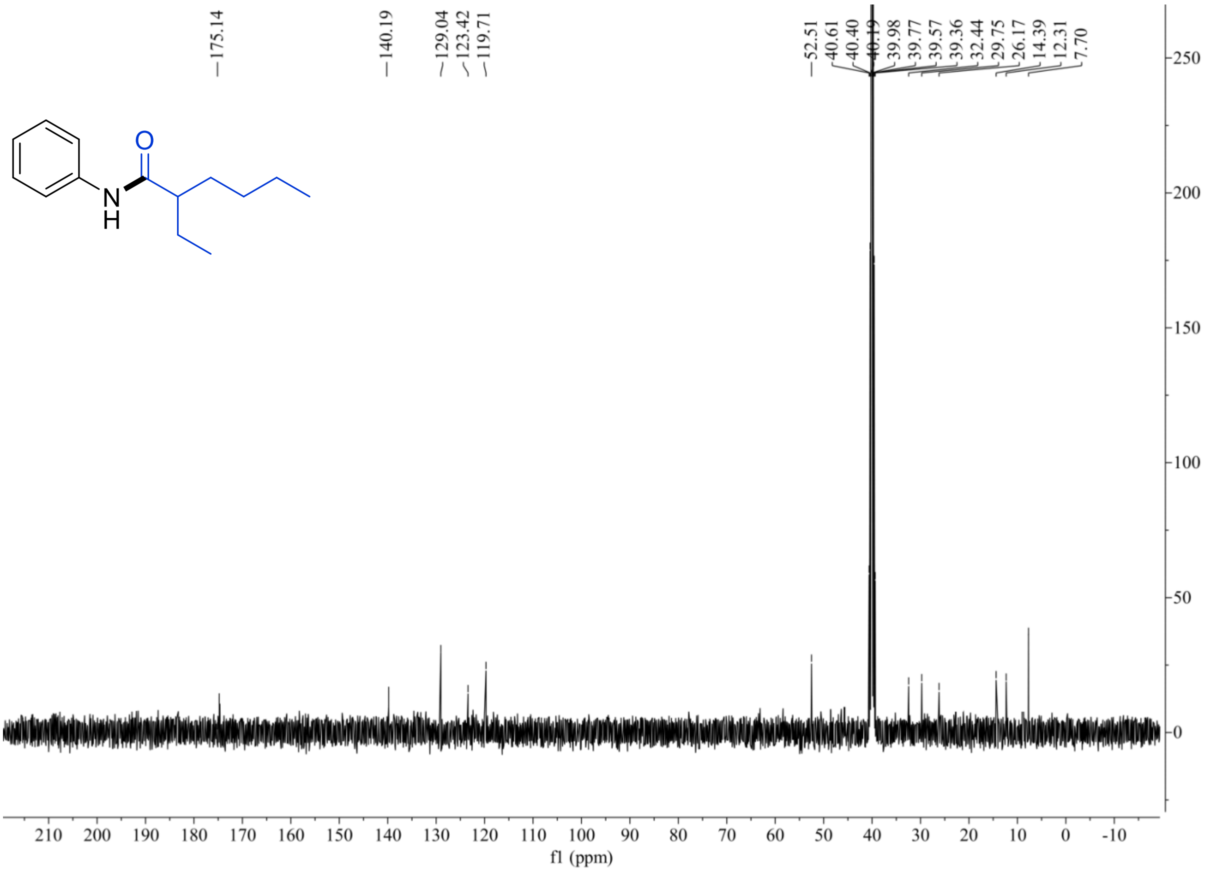

Supplementary Figure 21a | ¹H NMR (400 MHz, DMSO-d_6) of *N*-phenylcyclohexanecarboxamide (**3d**)

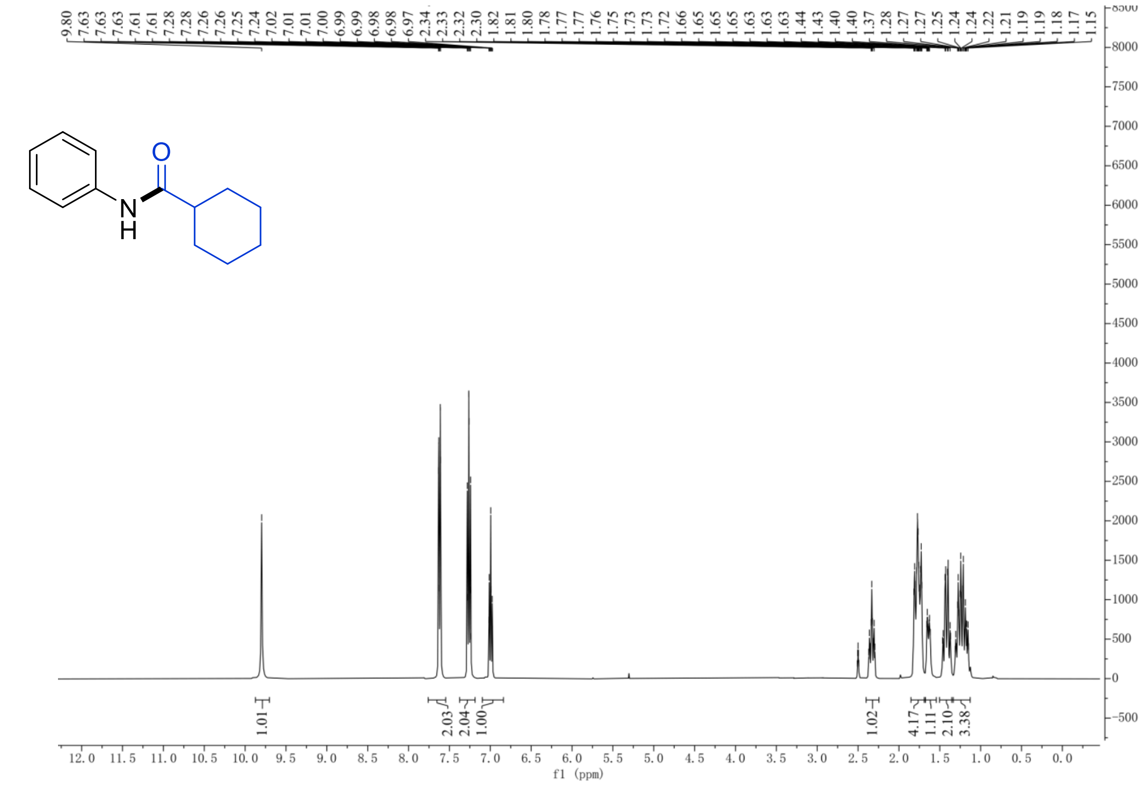

Supplementary Figure 21b | ^{13}C NMR (100 MHz, DMSO-d_6) of *N*-phenylcyclohexanecarboxamide (**3d**)

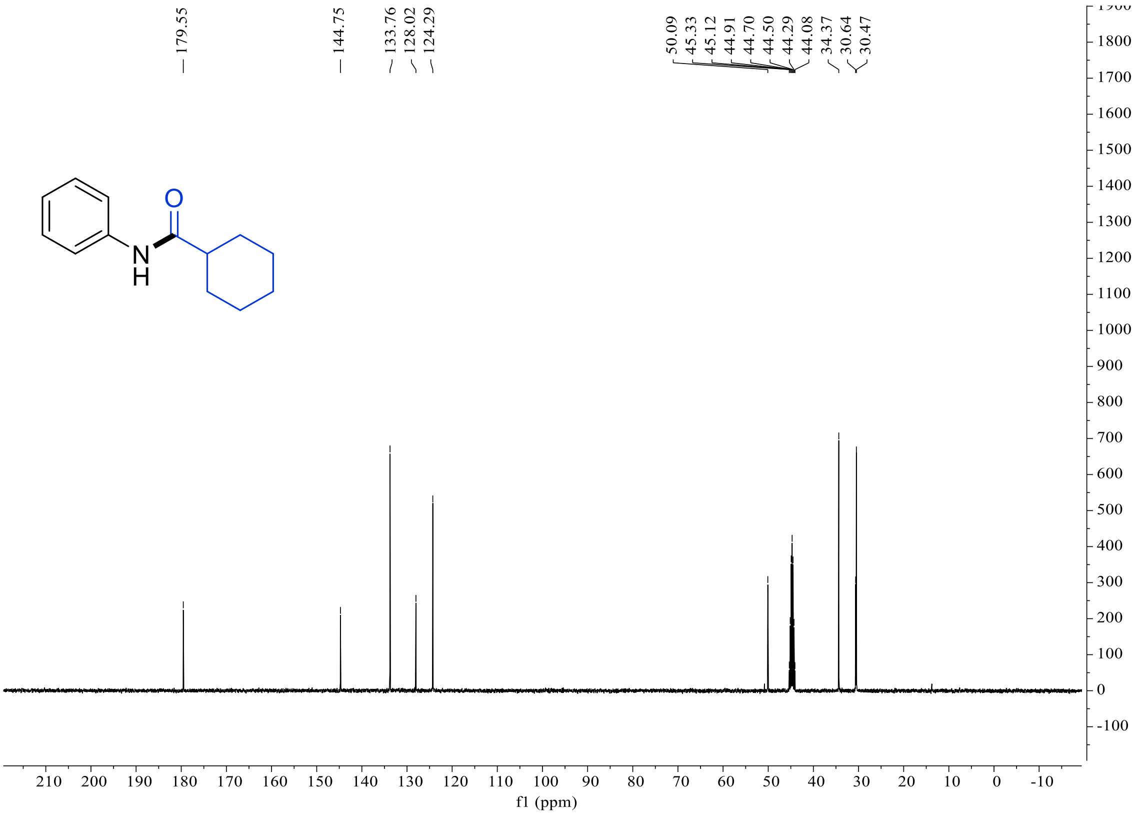

Supplementary Figure 22a | ^{1}H NMR (400 MHz, DMSO-d_6) of *N*-phenyltetrahydro-2*H*-pyran-4-carboxamide **(3e)**.

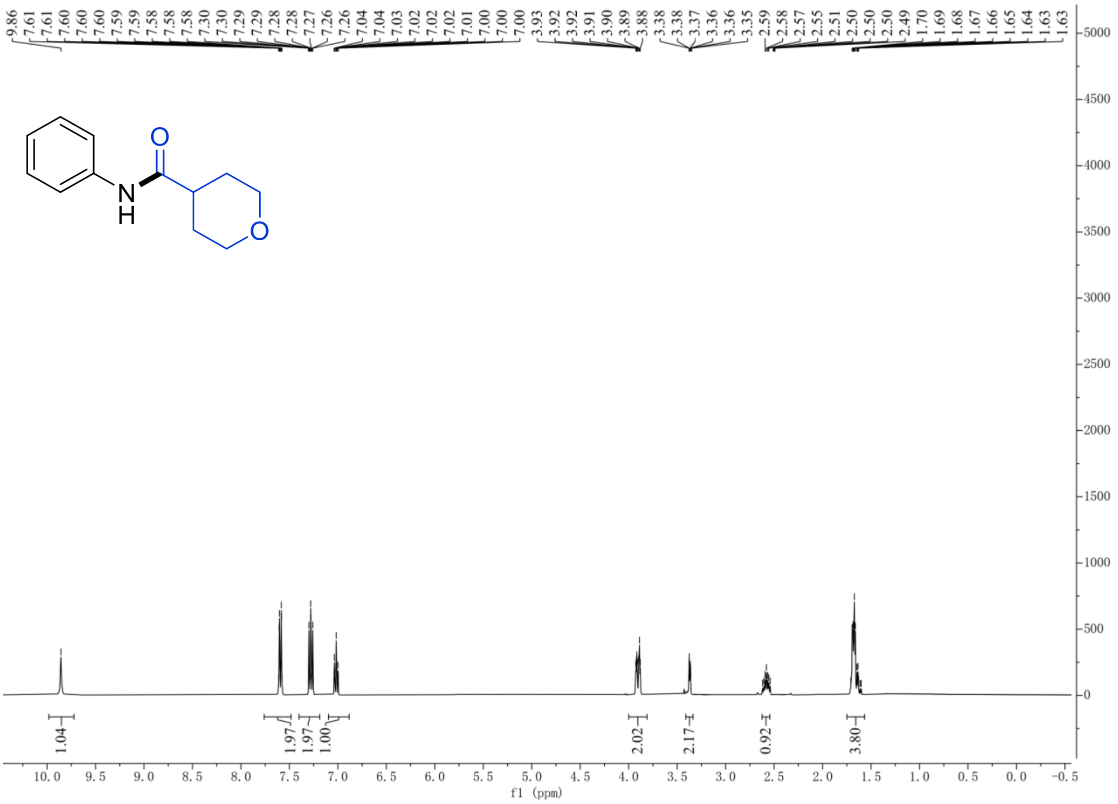

Supplementary Figure 22b | ^{13}C NMR (100 MHz, Chloroform-*d*) of *N*-phenyltetrahydro-2*H*-pyran-4-carboxamide **(3e)**.

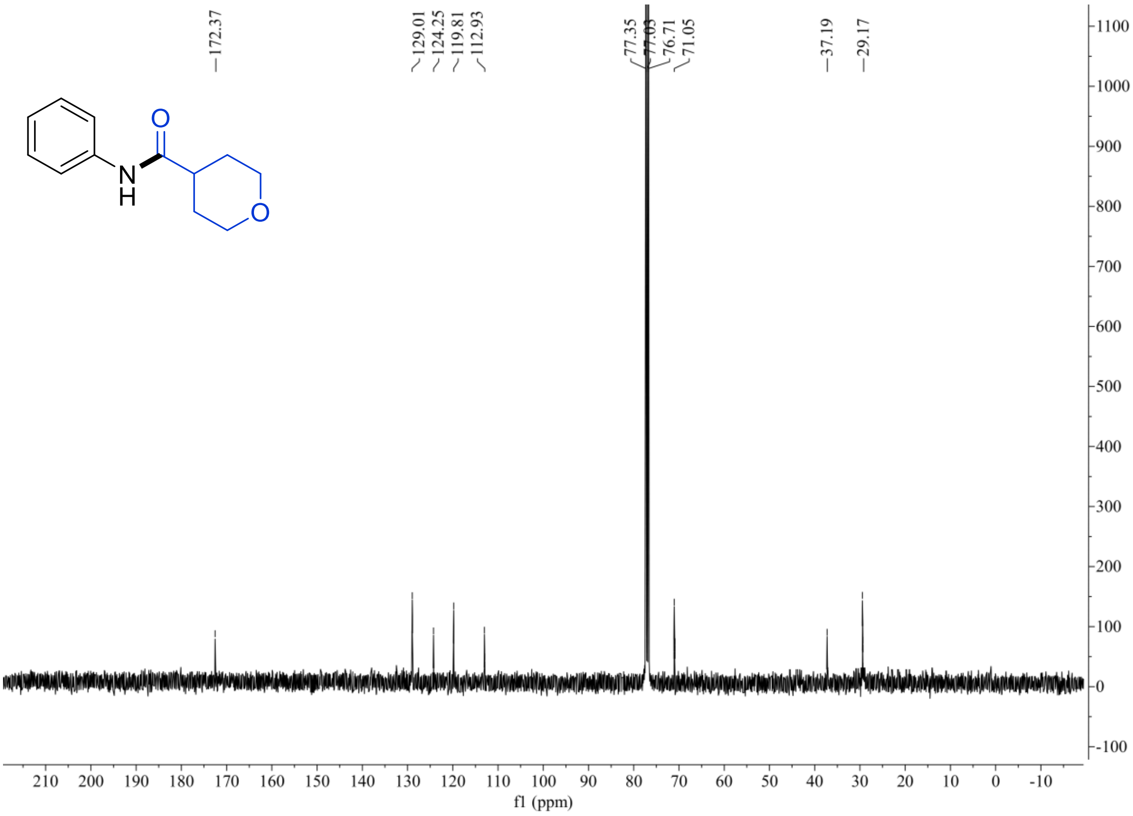

Supplementary Figure 23a | ^{1}H NMR (400 MHz, Chloroform-*d*) of *N*-phenylacetamide **(3f).**

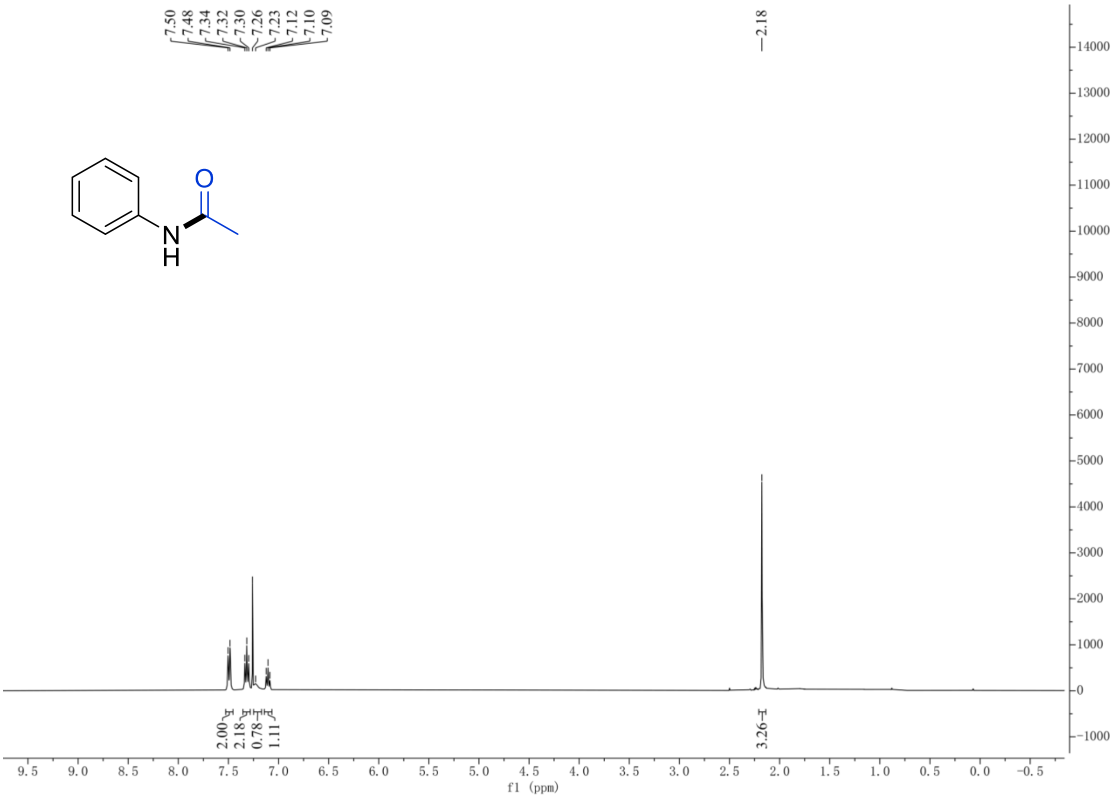

Supplementary Figure 23b | ^{13}C NMR (100 MHz, DMSO-d_6) of *N*-phenylacetamide **(3f).**

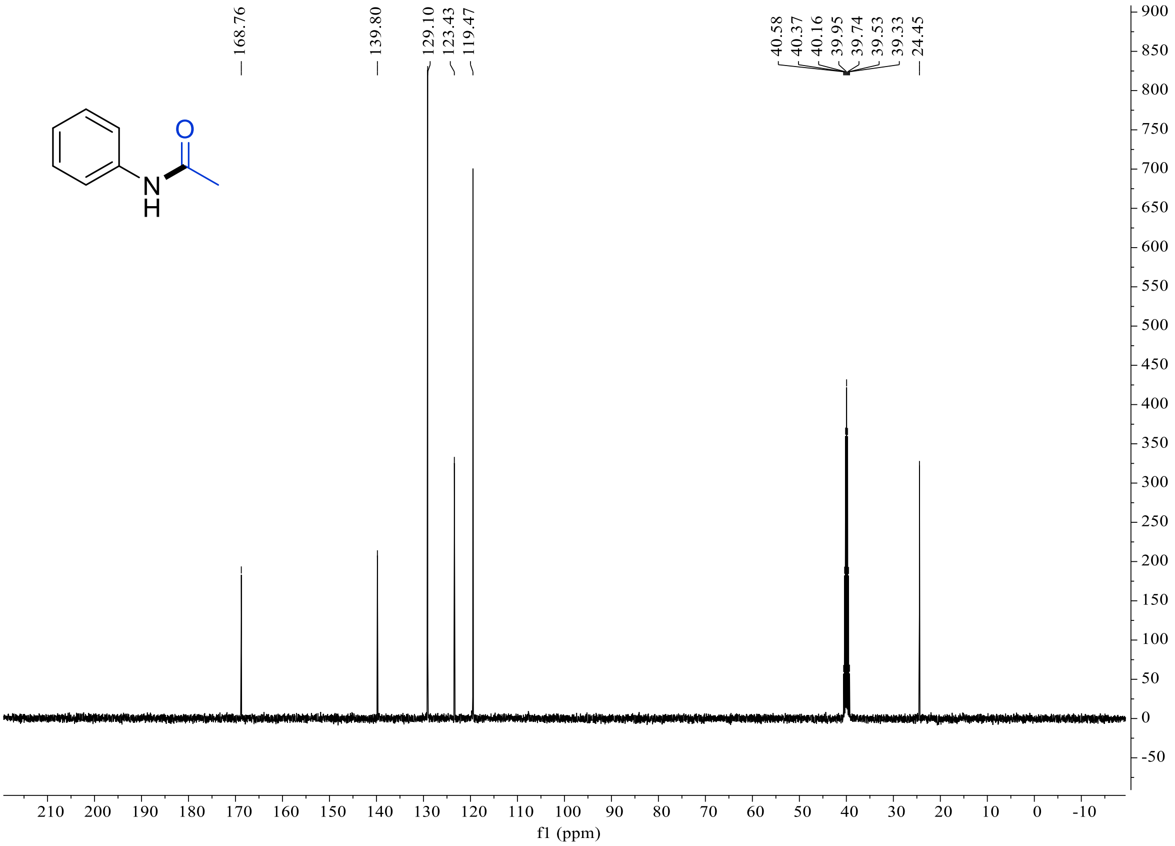

Supplementary Figure 24a | ^{1}H NMR (400 MHz, Chloroform-*d*) of *N*-phenylhexanamide **(3g).**

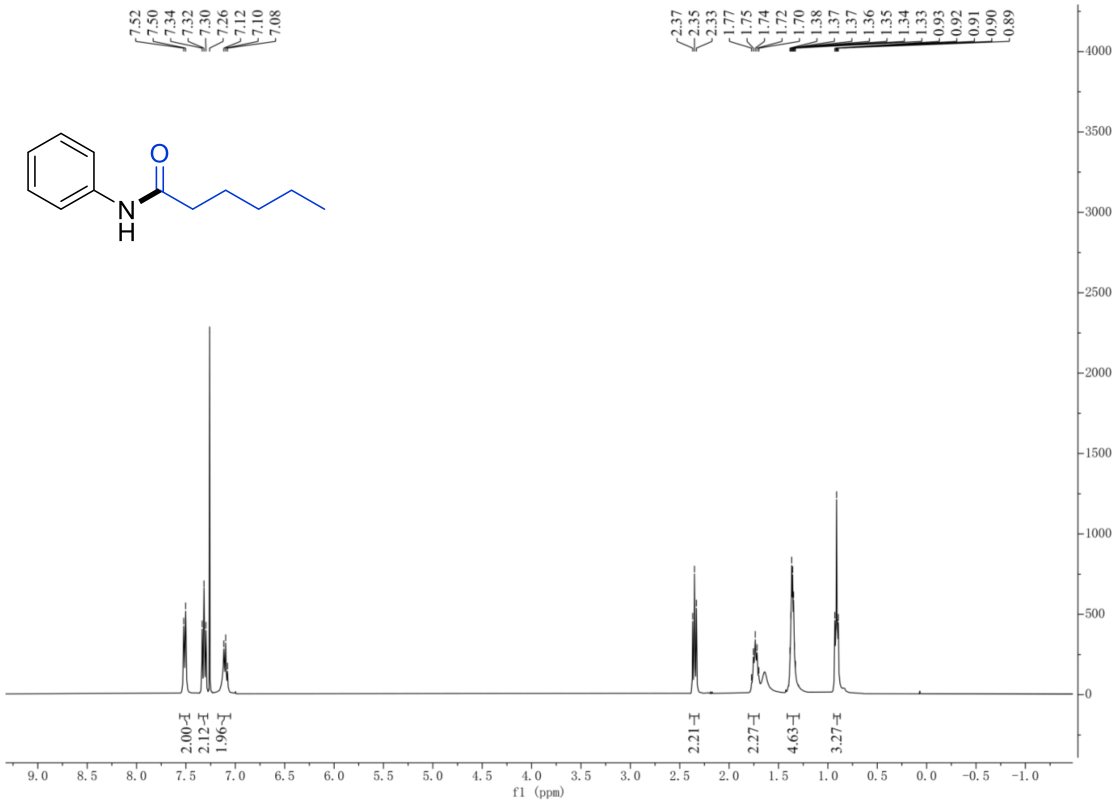

Supplementary Figure 24b | ^{13}C NMR (100 MHz, DMSO-d_6) of *N*-phenylhexanamide **(3g).**

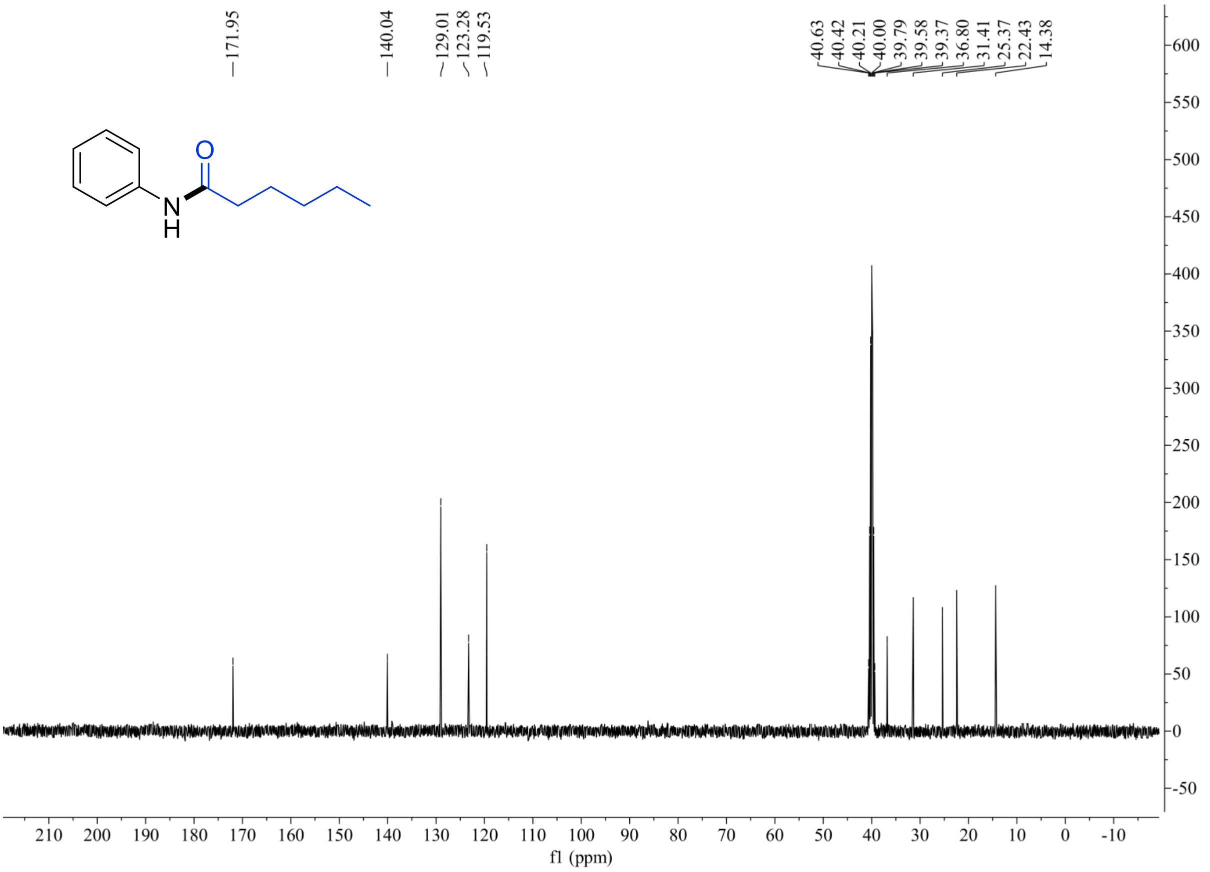

Supplementary Figure 25a | ^{1}H NMR (400 MHz, Chloroform-*d*) of 3-methyl-*N*-phenylbutanamide **(3h).**

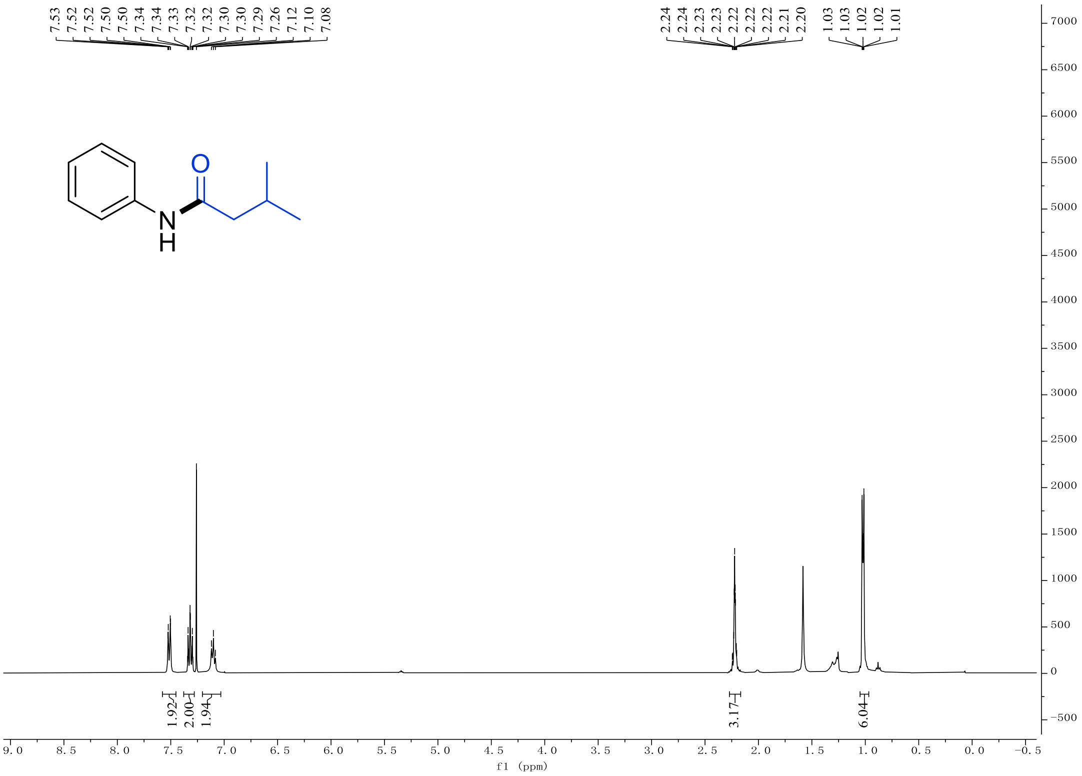

Supplementary Figure 25b | ^{13}C NMR (126 MHz, DMSO-*d$_6$*) of 3-methyl-*N*-phenylbutanamide **(3h).**

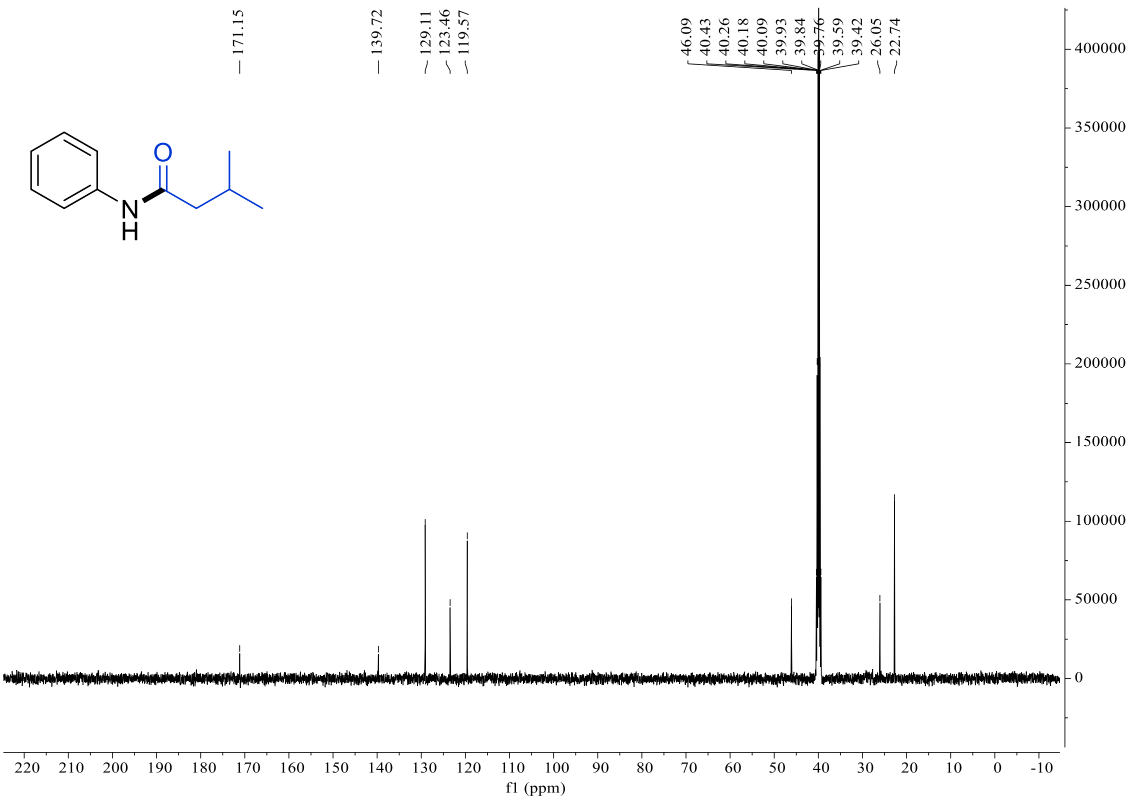

Supplementary Figure 26a | ^{1}H NMR (400 MHz, Chloroform-*d*) of *N*,2-diphenylacetamide **(3i).**

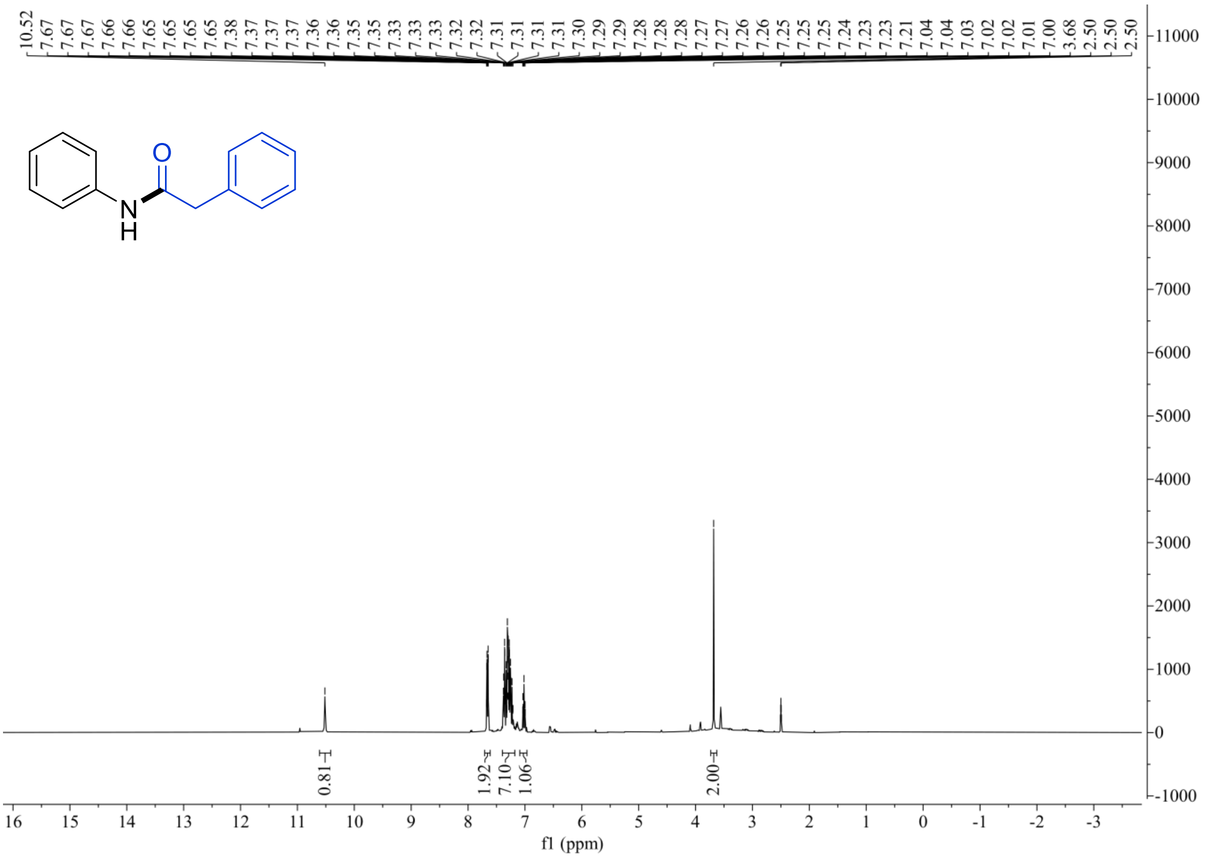

Supplementary Figure 26b | ^{13}C NMR (100 MHz, DMSO-d_6) of *N*,2-diphenylacetamide **(3i).**

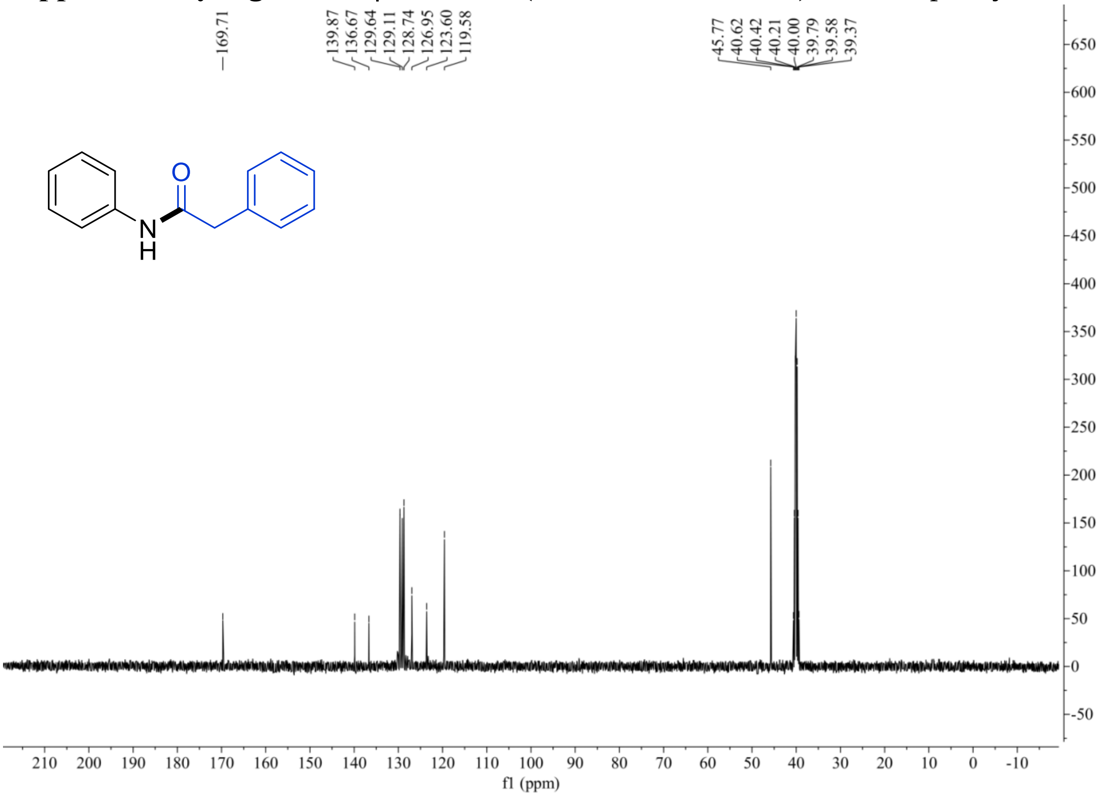

Supplementary Figure 27a | ^{1}H NMR (400 MHz, Chloroform-*d*) of 7-hydroxy-3,7-dimethyl-*N*-phenyloctanamide **(3j).**

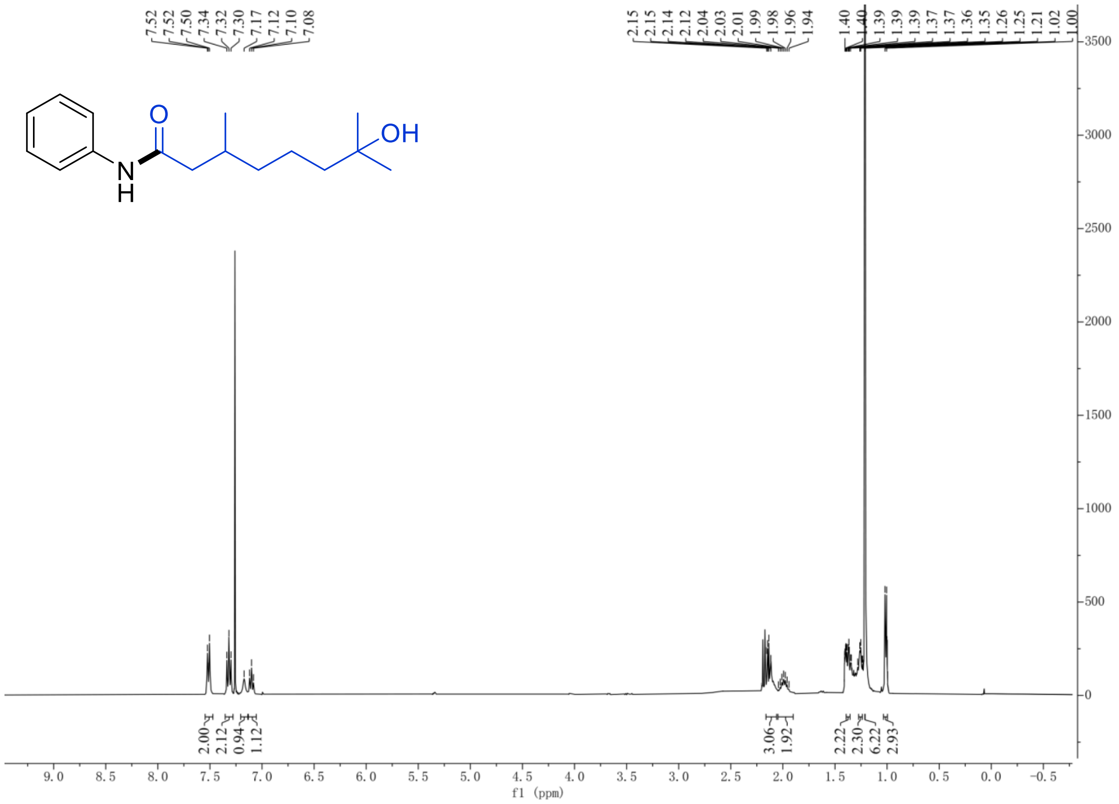

Supplementary Figure 27b | ^{13}C NMR (126 MHz, Chloroform-*d*) of 7-hydroxy-3,7-dimethyl-*N*-phenyloctanamide **(3j).**

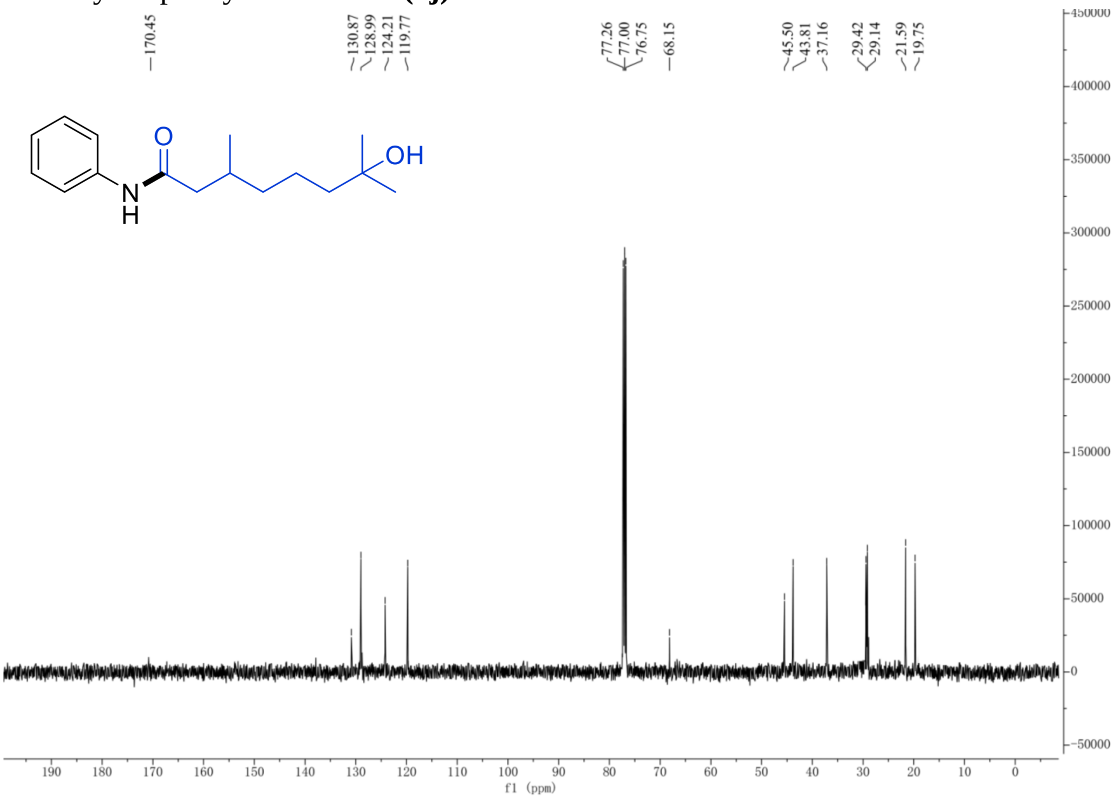

Supplementary Figure 28a | ¹H NMR (400 MHz, DMSO-d_6) of *N*-phenylbenzamide **(3k).**

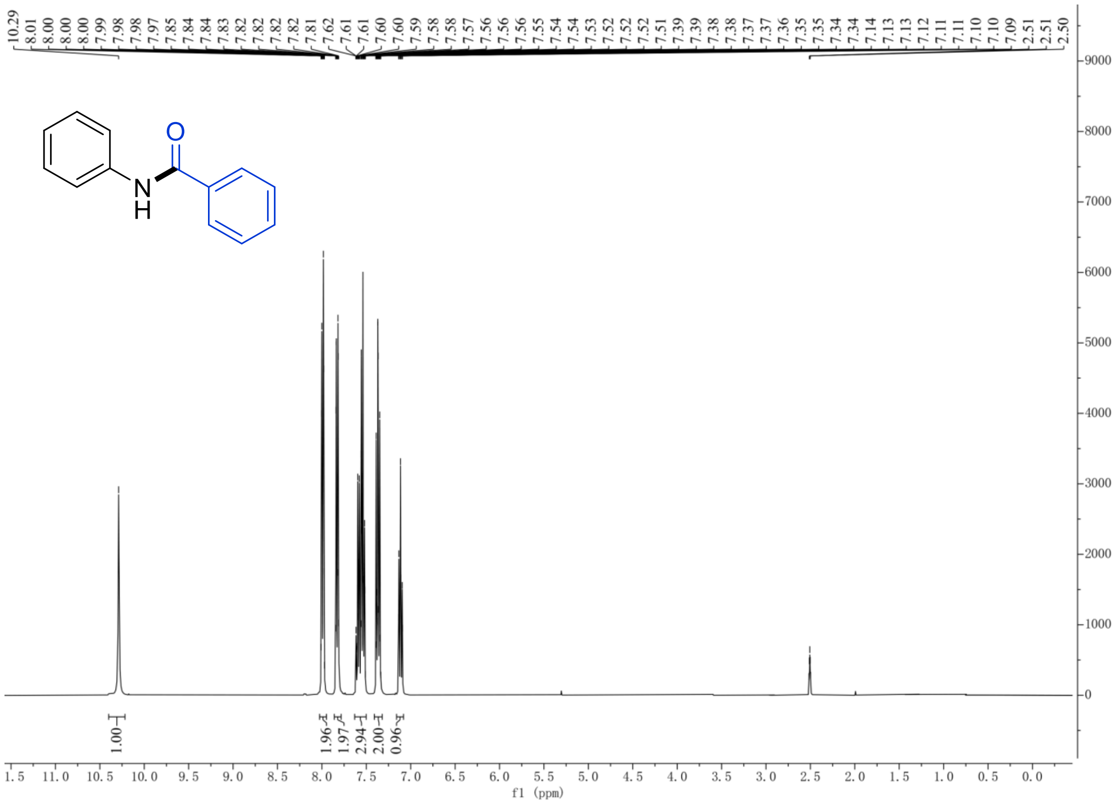

Supplementary Figure 28b | ¹³C NMR (126 MHz, DMSO-d_6) of *N*-phenylbenzamide **(3k).**

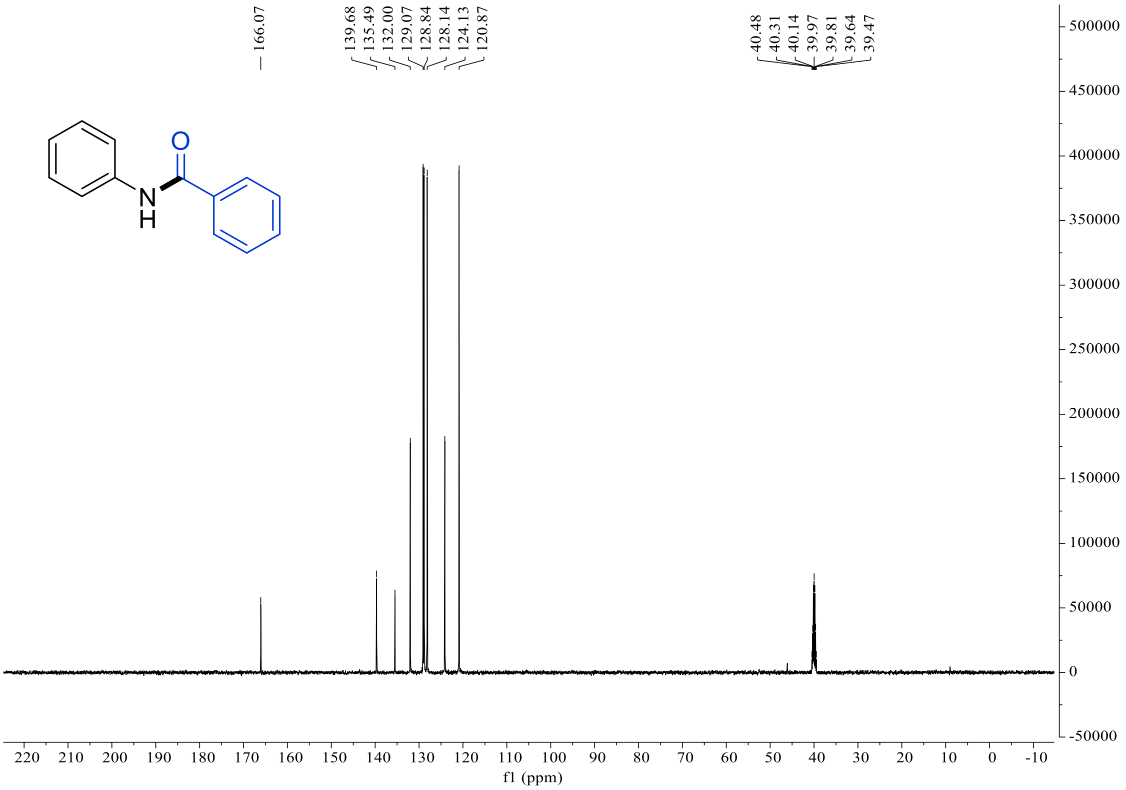

Supplementary Figure 29a | ^{1}H NMR (400 MHz, Chloroform-*d*) of 4-fluoro-*N*-phenylbenzamide **(3l).**

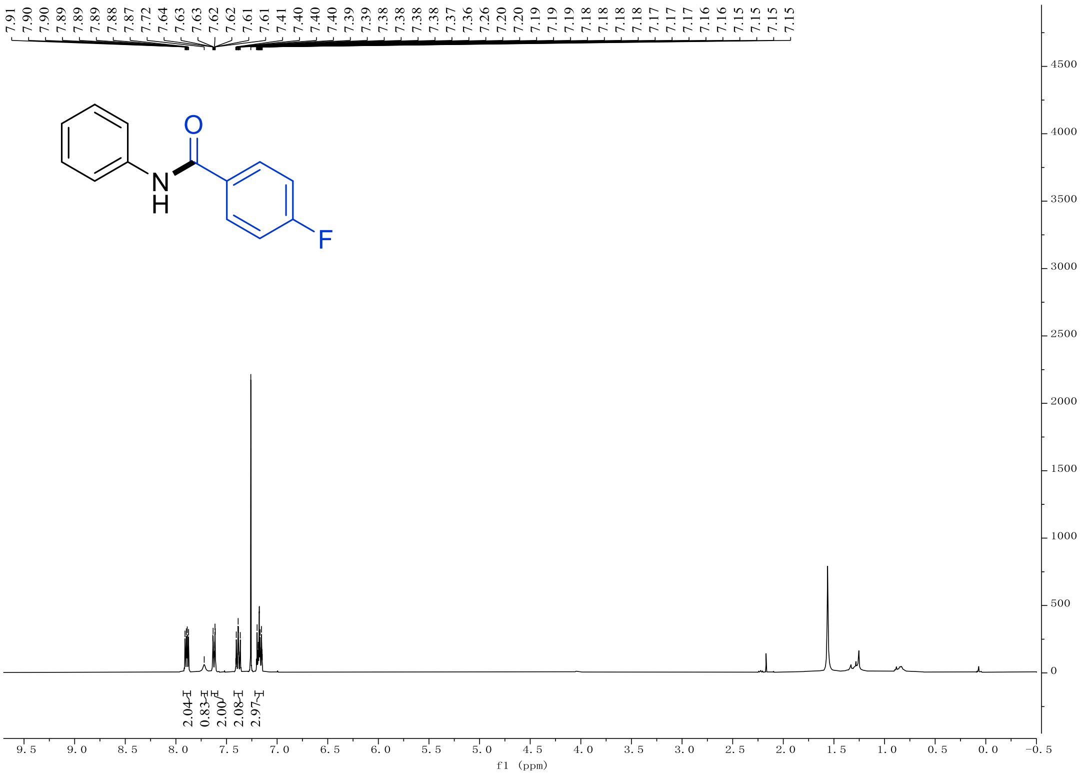

Supplementary Figure 29b | ^{13}C NMR (100 MHz, Chloroform-*d*) of 4-fluoro-*N*-phenylbenzamide **(3l).**

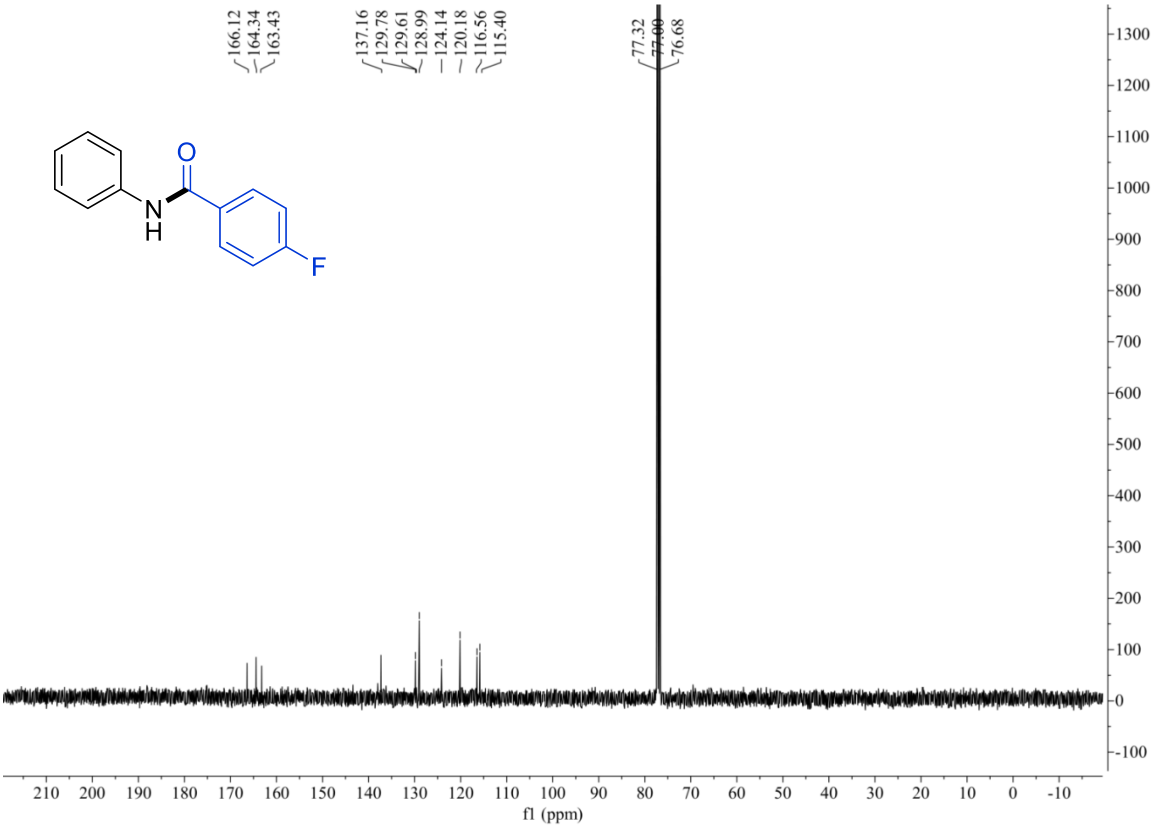

Supplementary Figure 30a | ^{1}H NMR (400 MHz, DMSO-d_6) of 4-chloro-*N*-phenylbenzamide **(3m).**

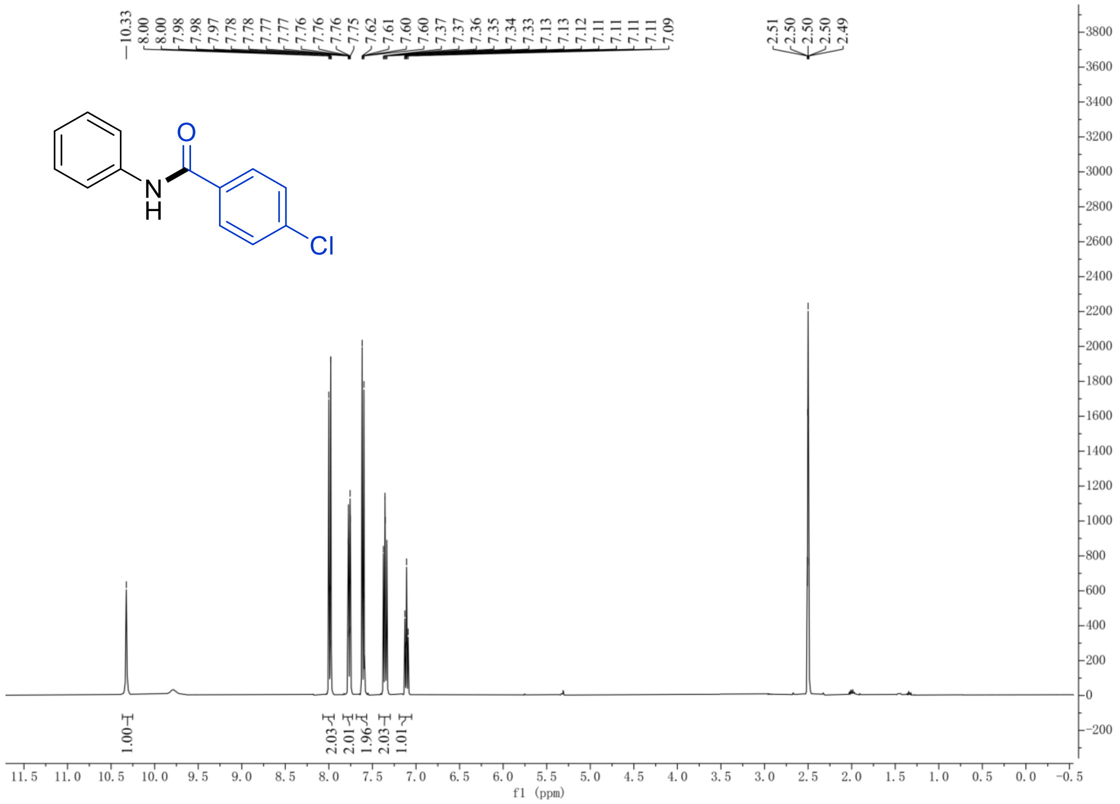

Supplementary Figure 30b | ^{13}C NMR (126 MHz, DMSO-d_6) of 4-chloro-*N*-phenylbenzamide **(3m).**

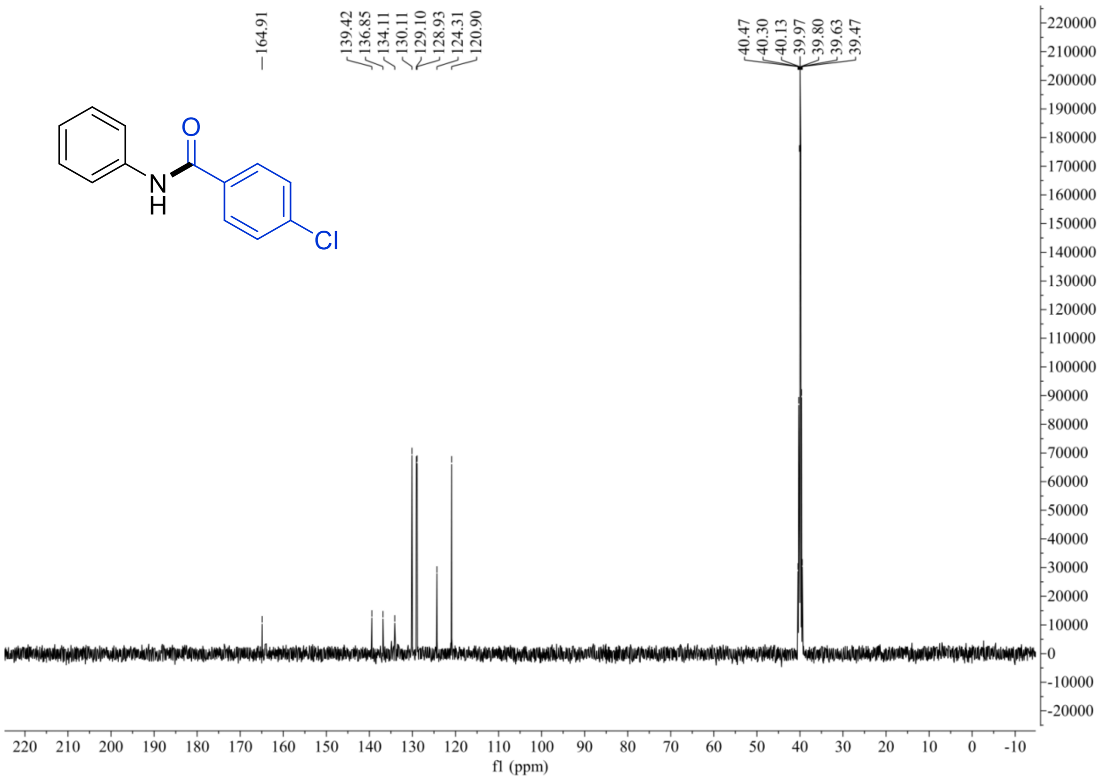

Supplementary Figure 31a | ^{1}H NMR (400 MHz, Chloroform-*d*) of 4-bromo-*N*-phenylbenzamide **(3n).**

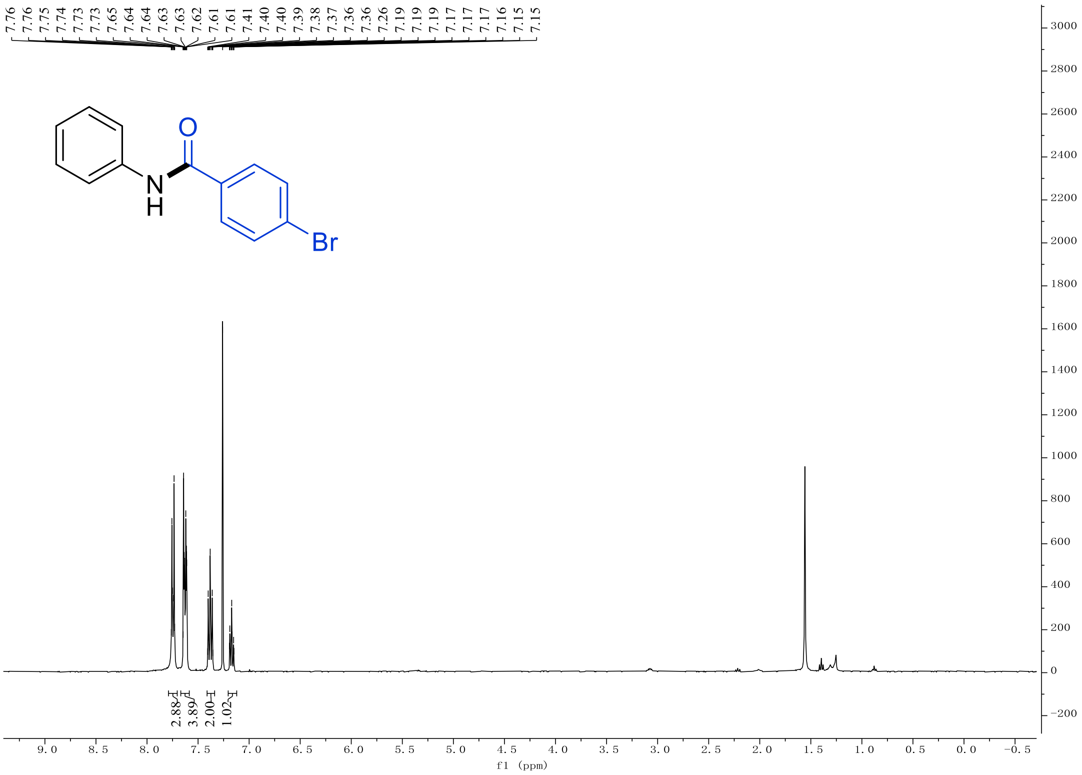

Supplementary Figure 31b | ^{13}C NMR (126 MHz, Chloroform-*d*) of 4-bromo-*N*-phenylbenzamide **(3n).**

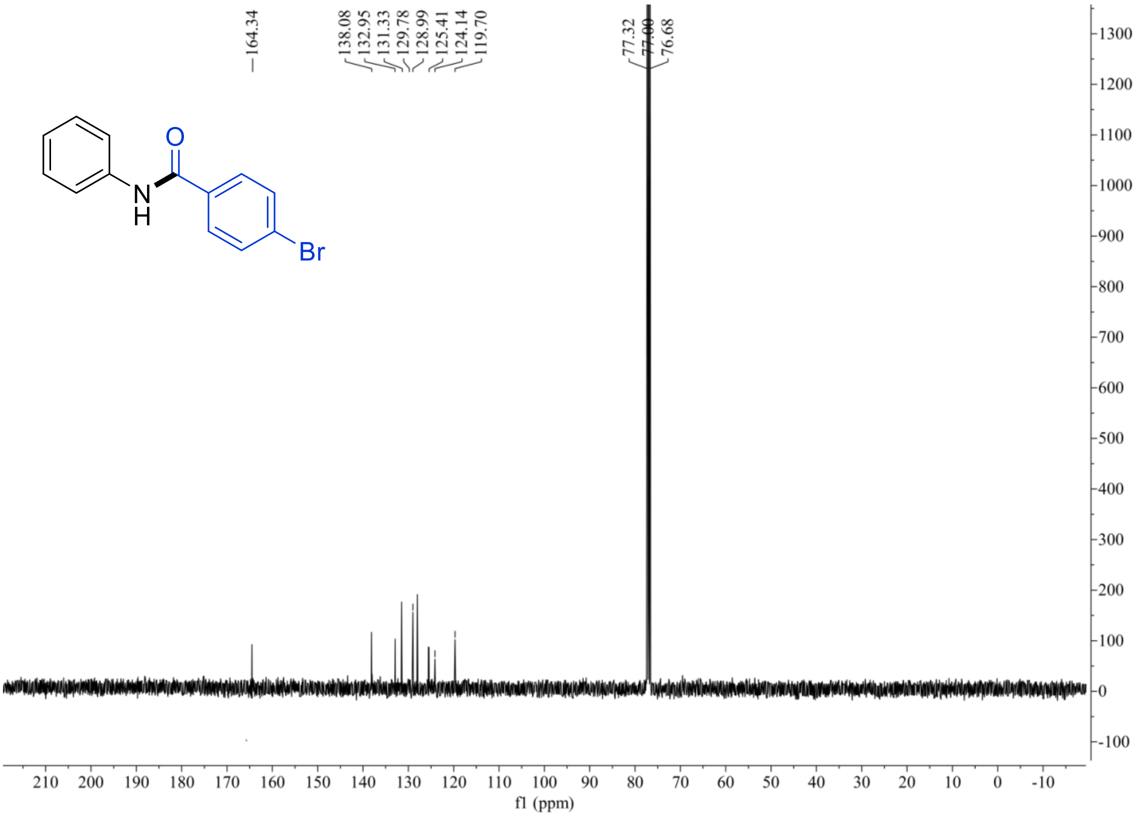

Supplementary Figure 32a | ^{1}H NMR (400 MHz, DMSO-d_6) of **4-iodo-*N*-phenylbenzamide (3o)**

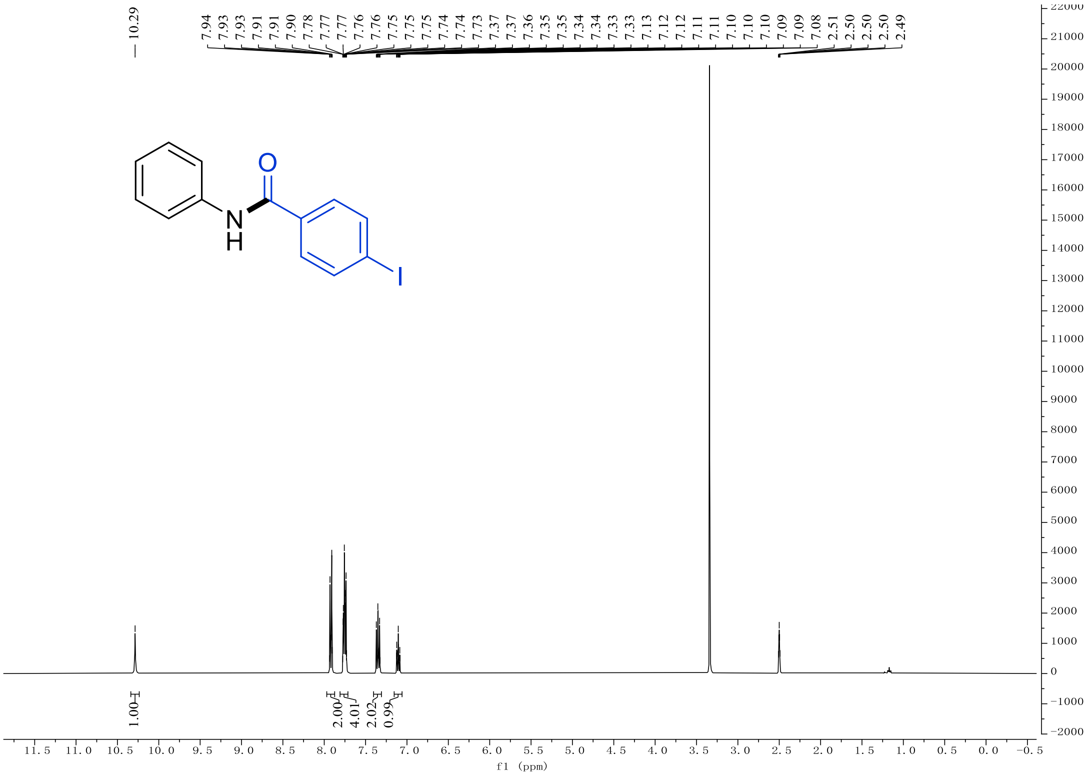

Supplementary Figure 32b | ^{13}C NMR (126 MHz, DMSO-d_6) of **4-iodo-*N*-phenylbenzamide (3o)**

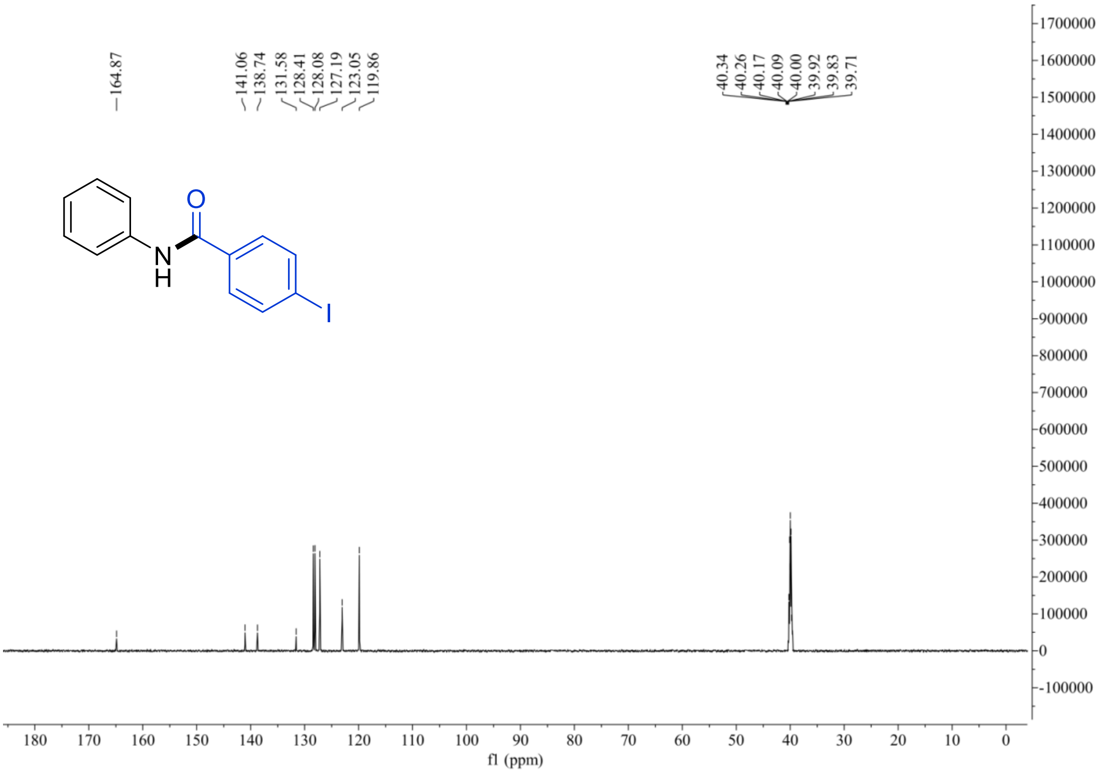

Supplementary Figure 33a | ^{1}H NMR (400 MHz, Chloroform-*d*) of 4-cyano-*N*-phenylbenzamide **(3p).**

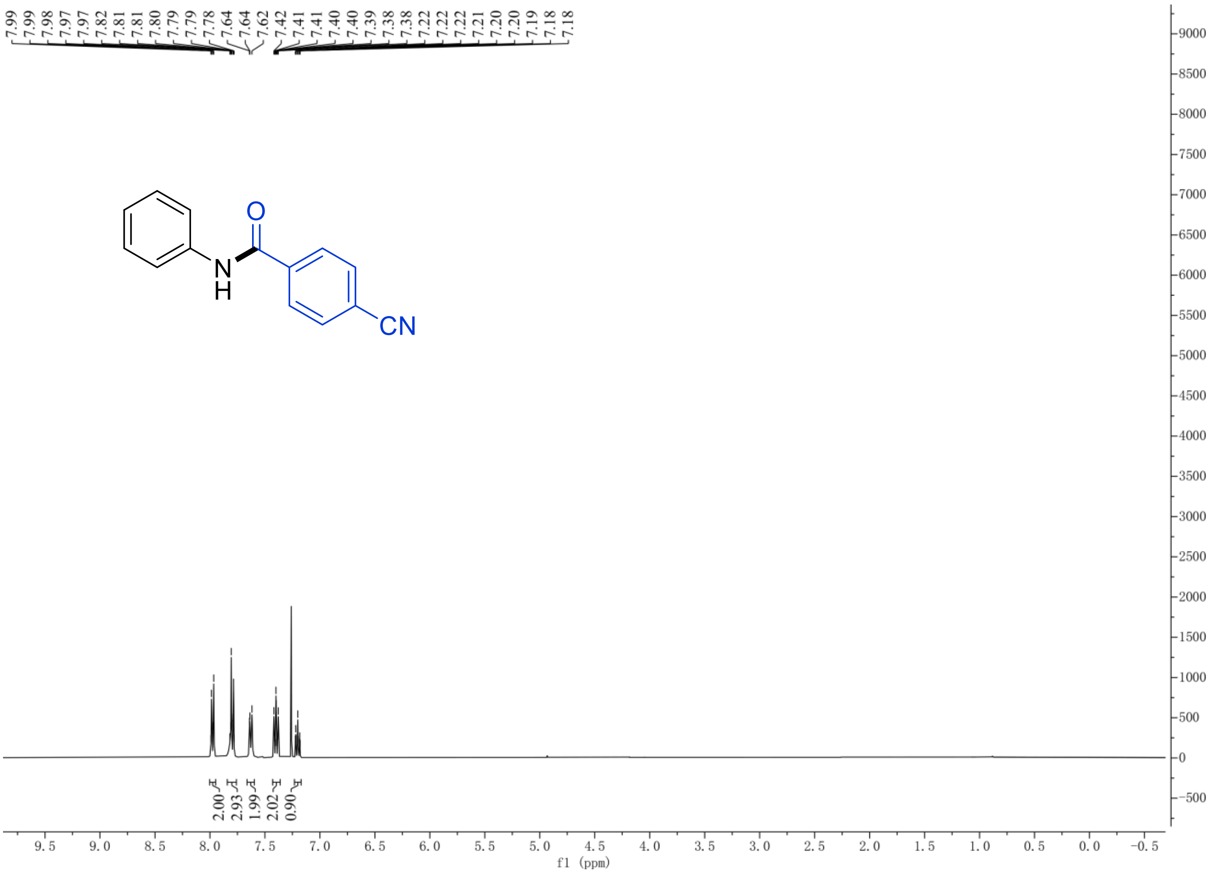

Supplementary Figure 33b | ^{13}C NMR (126 MHz, DMSO-*d*$_6$) of 4-cyano-*N*-phenylbenzamide **(3p).**

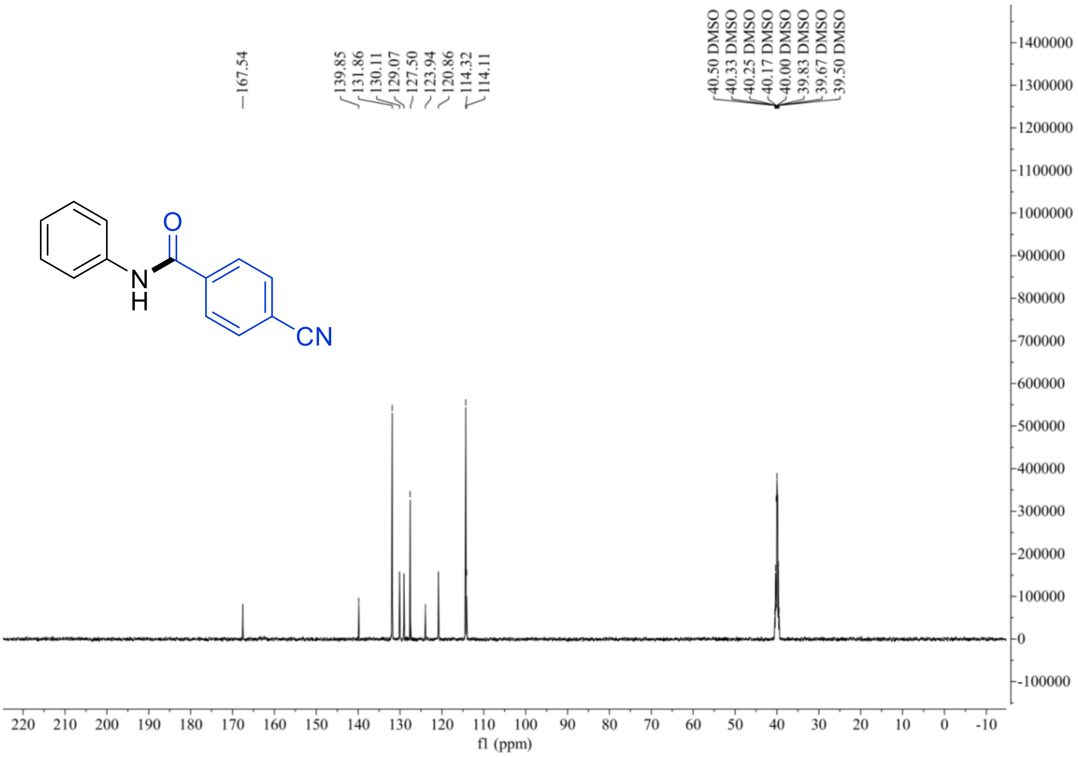

Supplementary Figure 34a | ^{1}H NMR (400 MHz, DMSO-d_6) of 3-cyano-*N*-phenylbenzamide **(3q).**

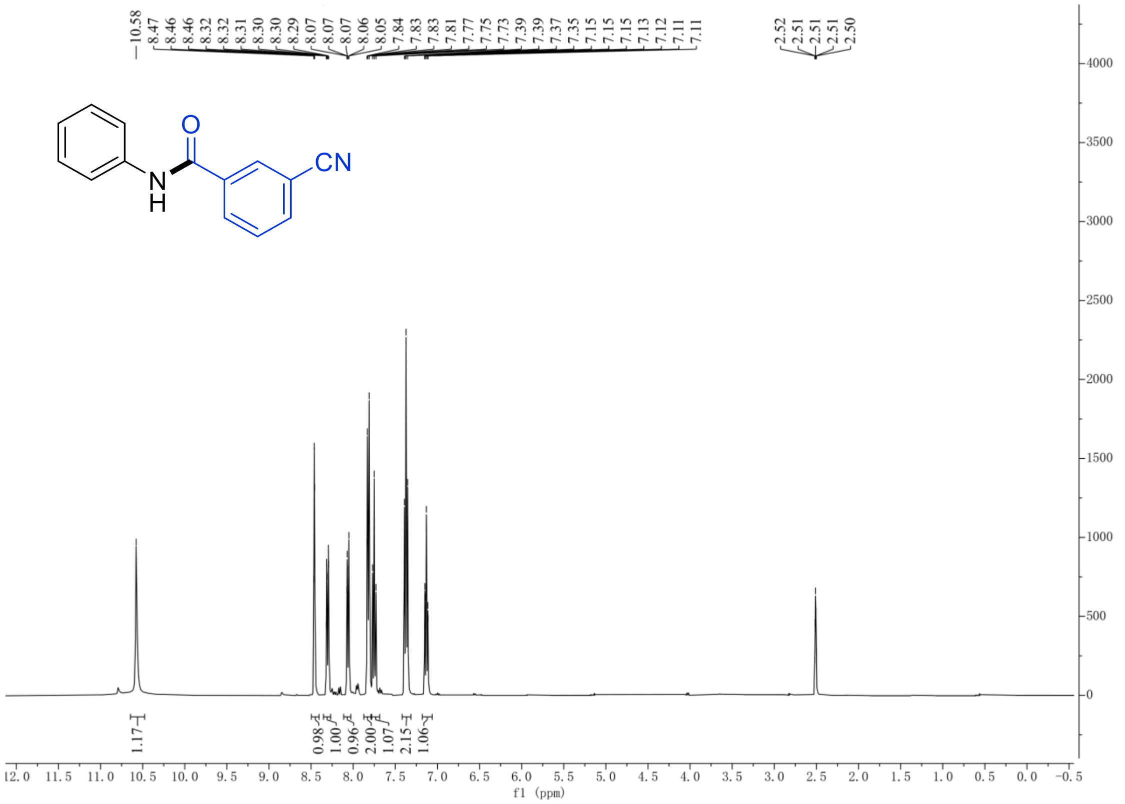

Supplementary Figure 34b | ^{13}C NMR (126 MHz, DMSO-d_6) of 3-cyano-*N*-phenylbenzamide **(3q).**

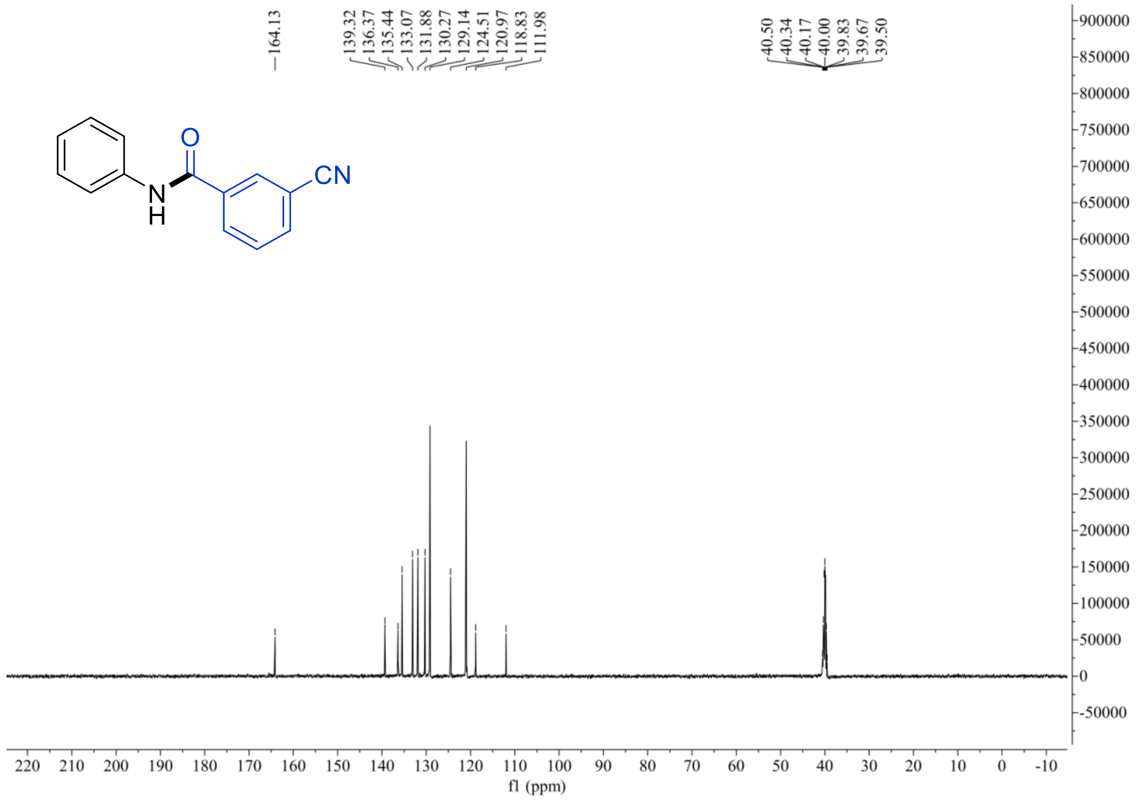

Supplementary Figure 35a | ^{1}H NMR (400 MHz, Chloroform-*d*) *N*-phenyl-4-(trifluoromethyl)benzamide **(3r).**

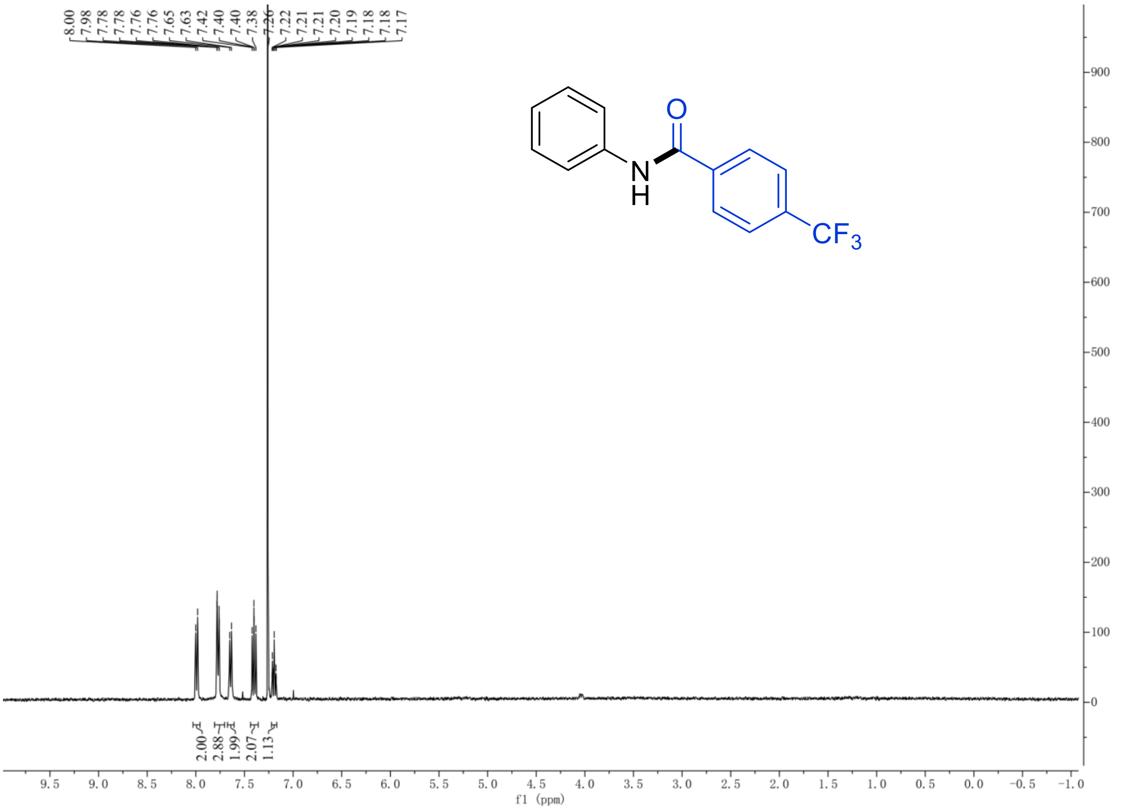

Supplementary Figure 35b | ^{13}C NMR (126 MHz, DMSO-*d*$_6$) *N*-phenyl-4-(trifluoromethyl)benzamide **(3r).**

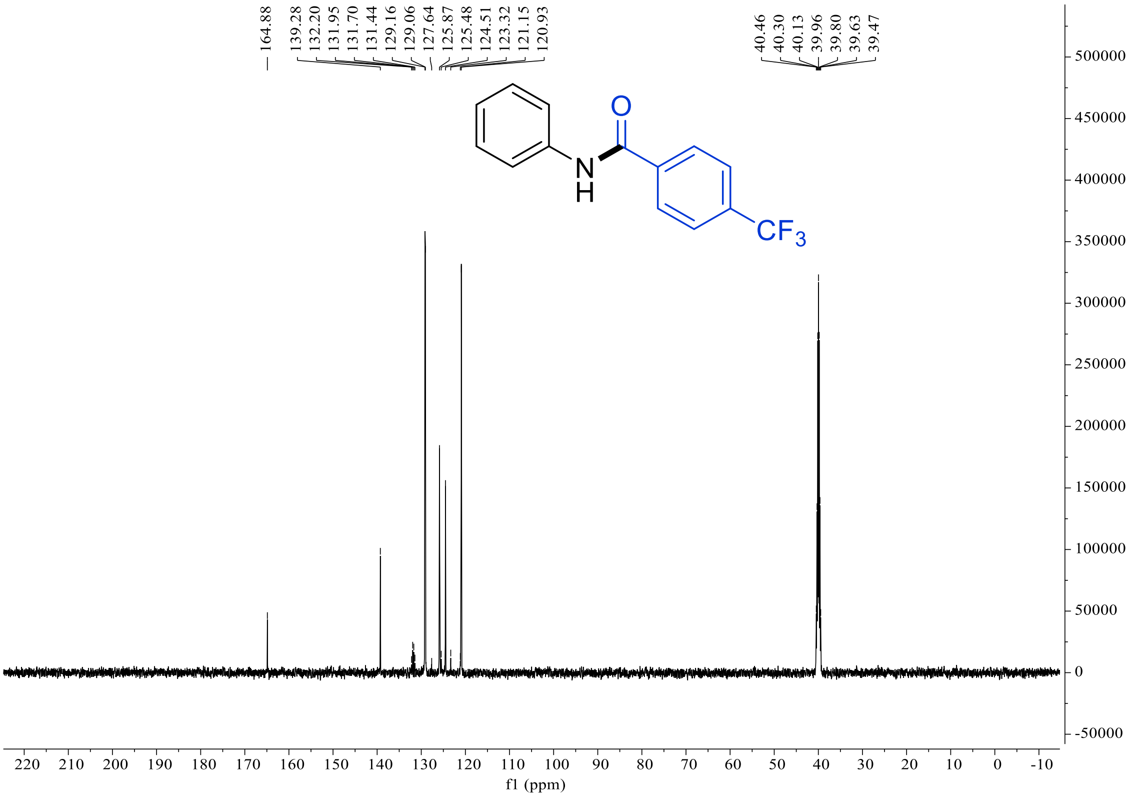

Supplementary Figure 36a | ^{1}H NMR (400 MHz, Chloroform-*d*) of 4-methyl-*N*-phenylbenzamide **(3s).**

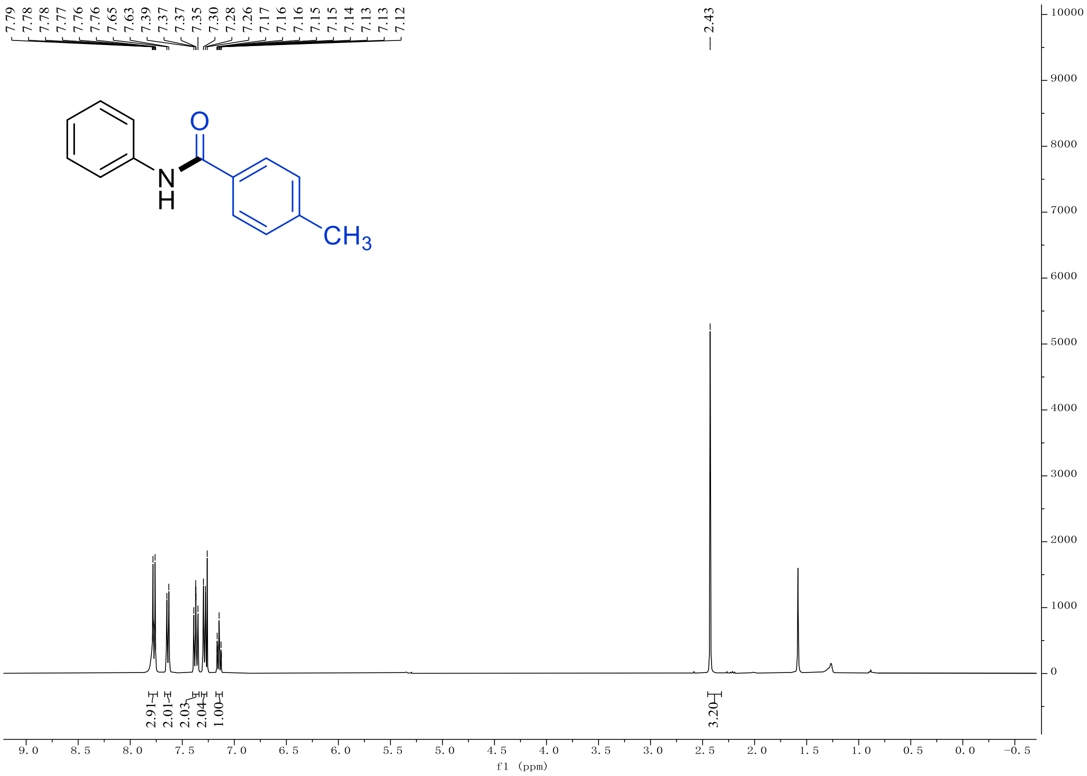

Supplementary Figure 36b | ^{13}C NMR (126 MHz, DMSO-d_6) of 4-methyl-*N*-phenylbenzamide **(3s).**

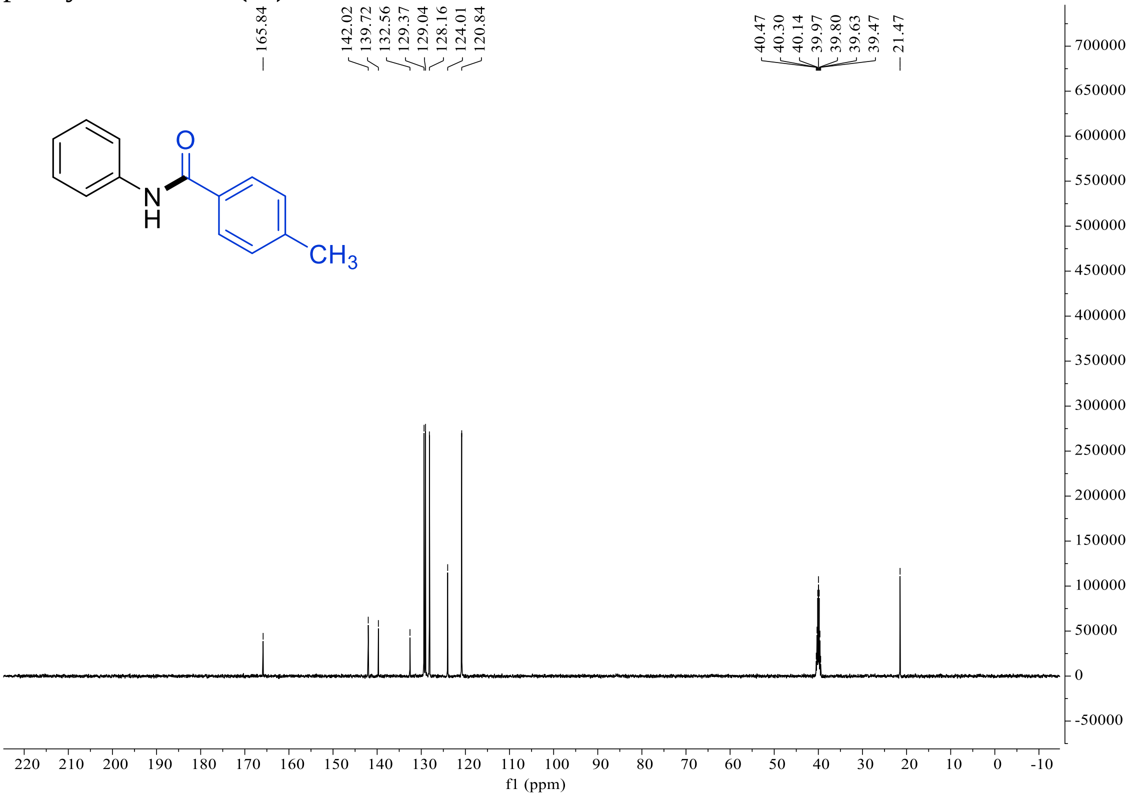

Supplementary Figure 37a | ^{1}H NMR (400 MHz, DMSO-d_6) of 4-methoxy-*N*-phenylbenzamide **(3t).**

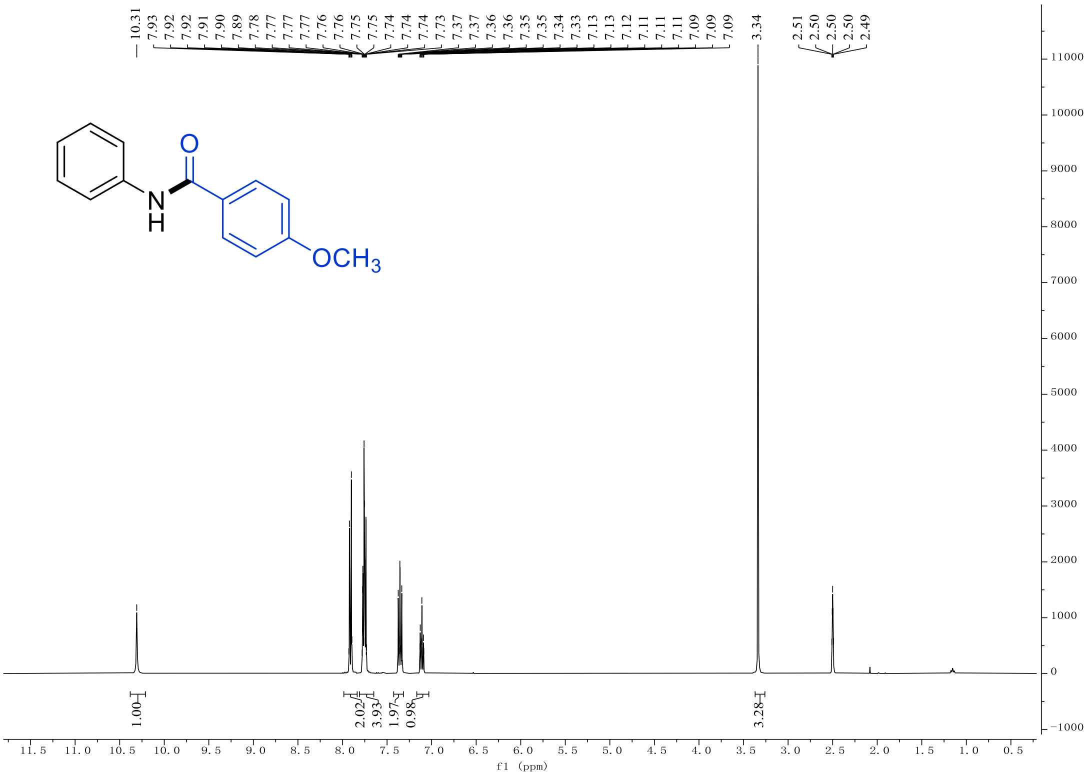

Supplementary Figure 37b | ^{13}C NMR (126 MHz, DMSO-d_6) of 4-methoxy-*N*-phenylbenzamide **(3t).**

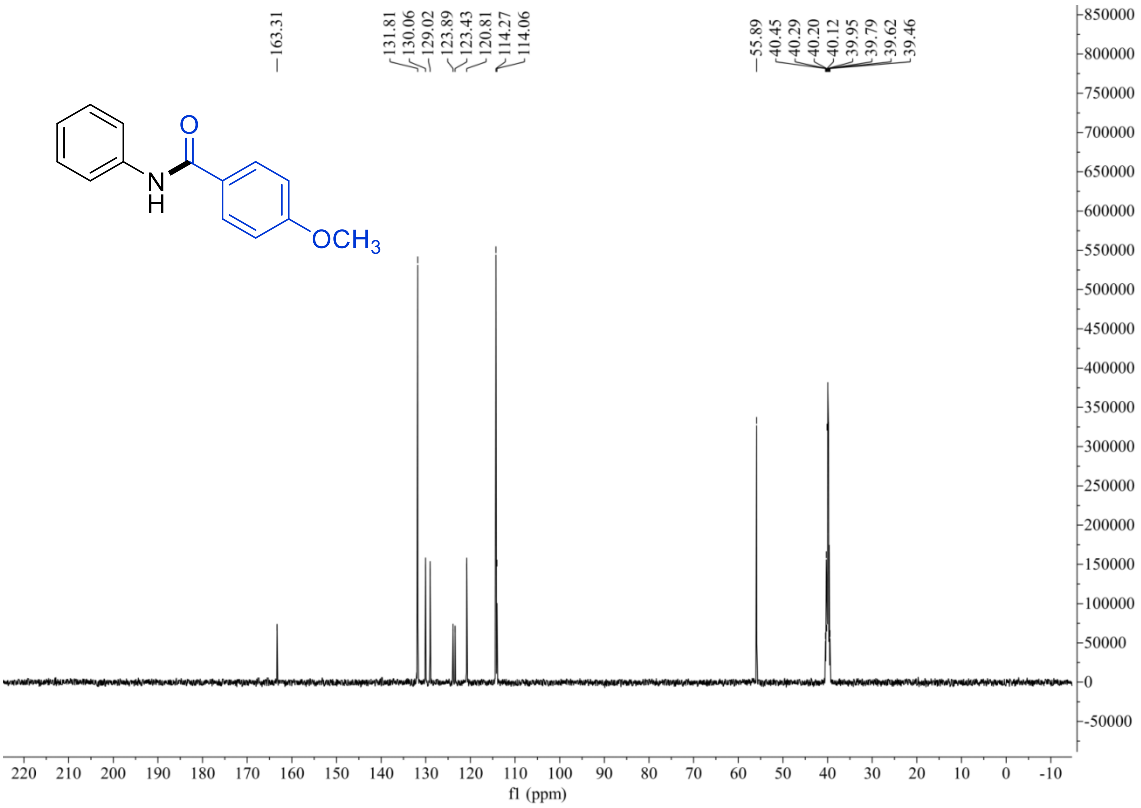

Supplementary Figure 38a | ^{1}H NMR (400 MHz, Chloroform-*d*) of 4-hydroxy-*N*-phenylbenzamide **(3u).**

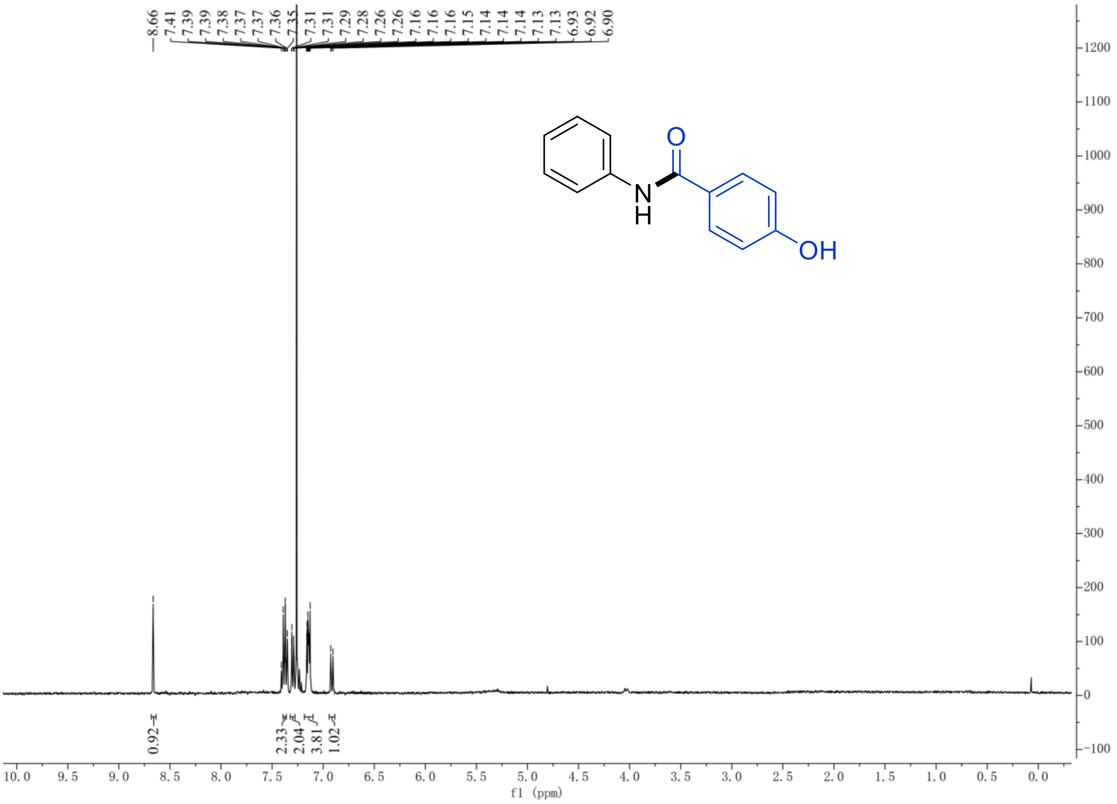

Supplementary Figure 38b | ^{13}C NMR (126 MHz, DMSO-d_6) of 4-hydroxy-*N*-phenylbenzamide **(3u).**

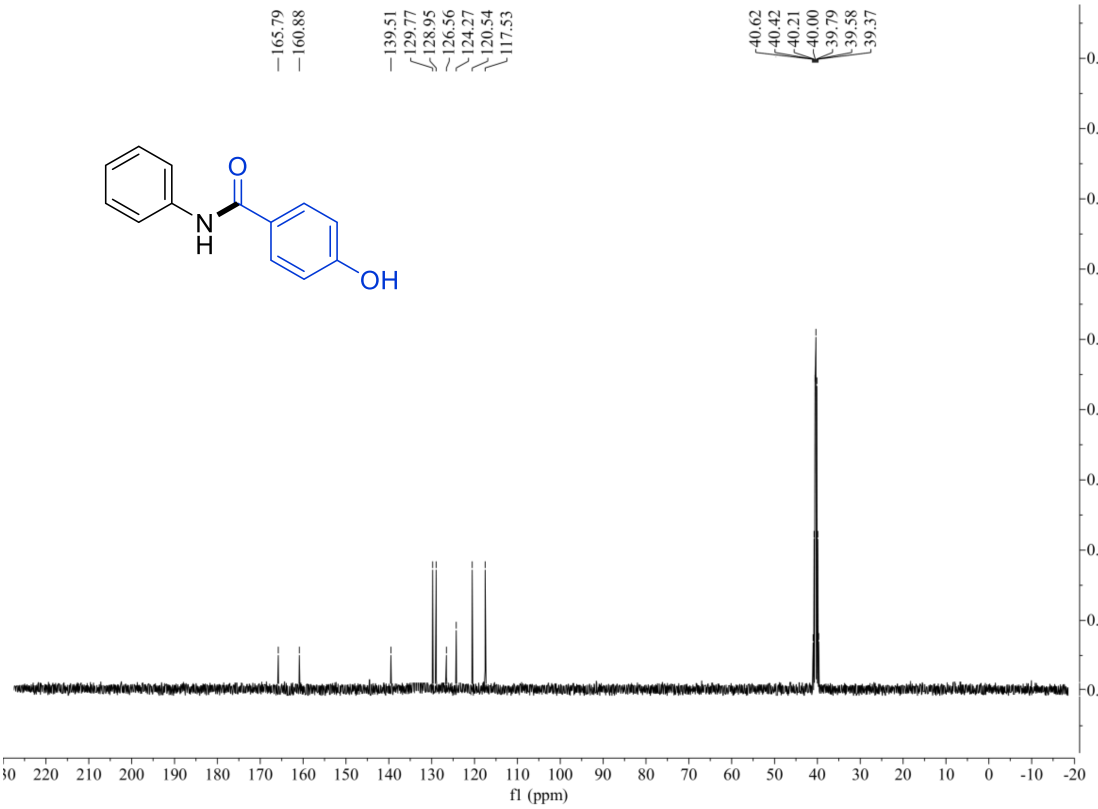

Supplementary Figure 39a | ¹H NMR (400 MHz, Chloroform-*d*) of *N*-phenyl-1-naphthamide **(3v).**

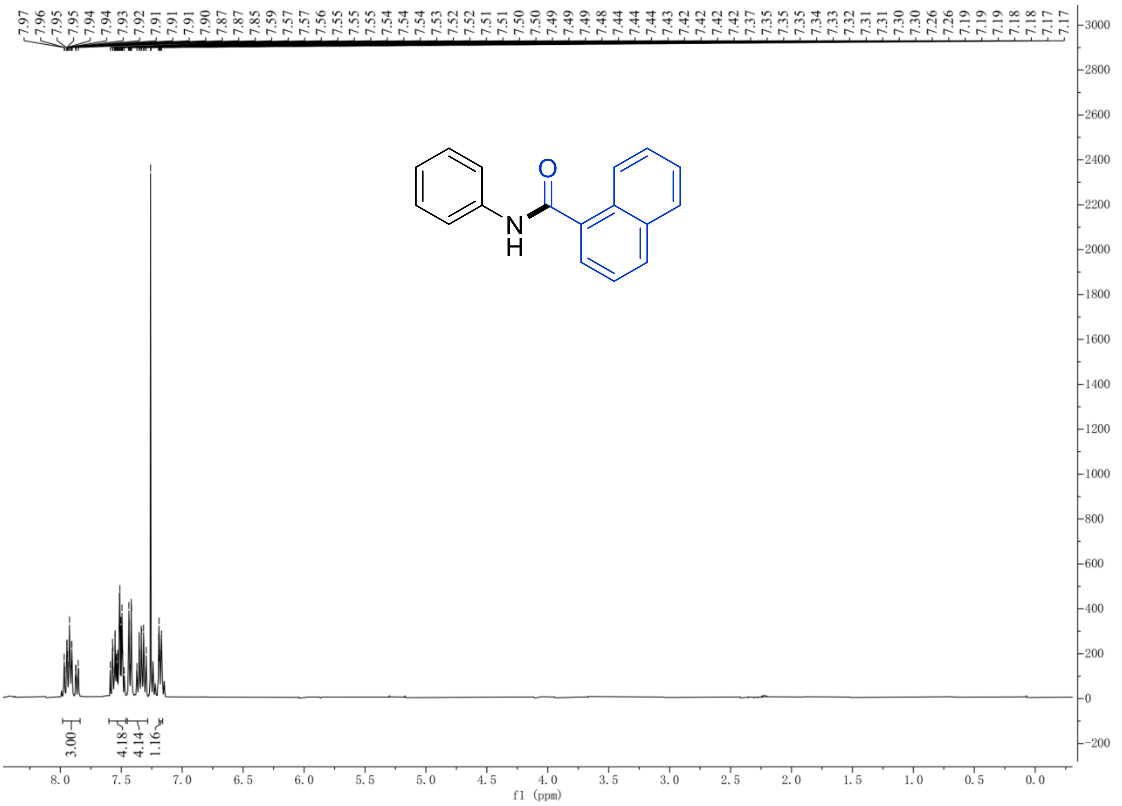

Supplementary Figure 39b | ¹³C NMR (126 MHz, DMSO-d_6) of *N*-phenyl-1-naphthamide **(3v).**

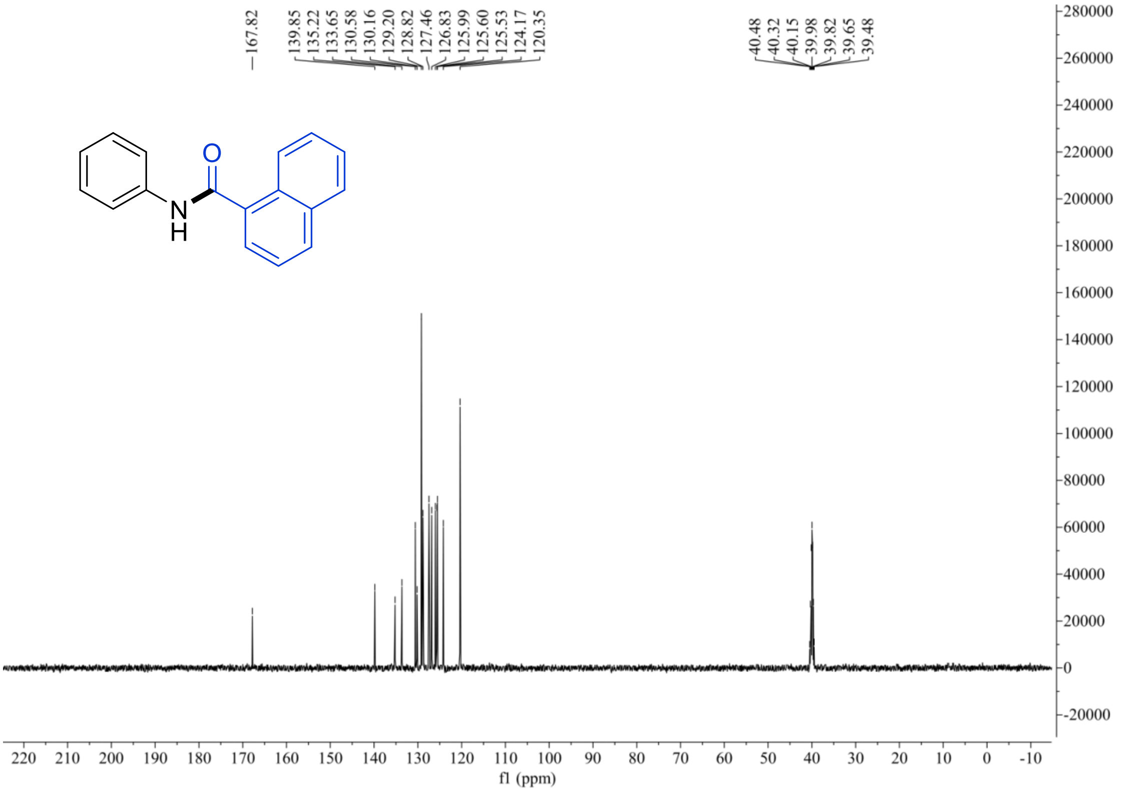

Supplementary Figure 40a | ¹H NMR (400 MHz, Chloroform-*d*) of *N*-phenylthiophene-2-carboxamide **(3w).**

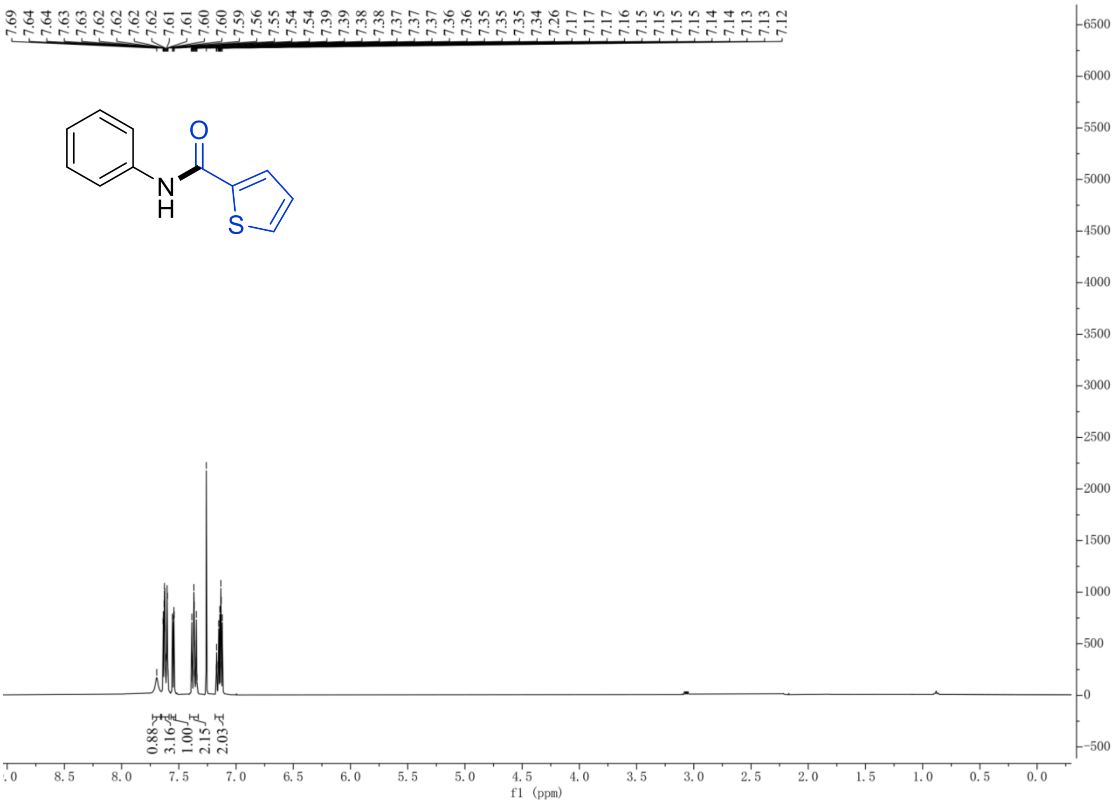

Supplementary Figure 40b | ¹³C NMR (126 MHz, DMSO-d_6) of *N*-phenylthiophene-2-carboxamide **(3w).**

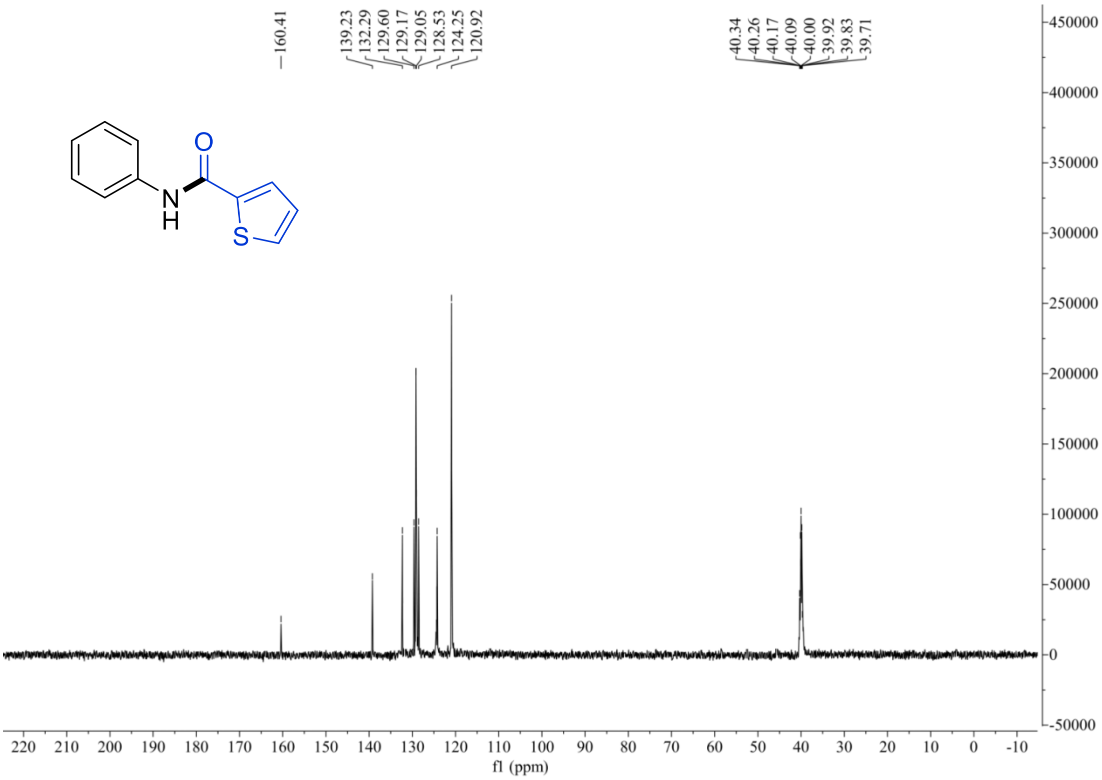

Supplementary Figure 41a | ^{1}H NMR (400 MHz, Chloroform-*d*) of *N*-phenylfuran-2-carboxamide **(3x).**

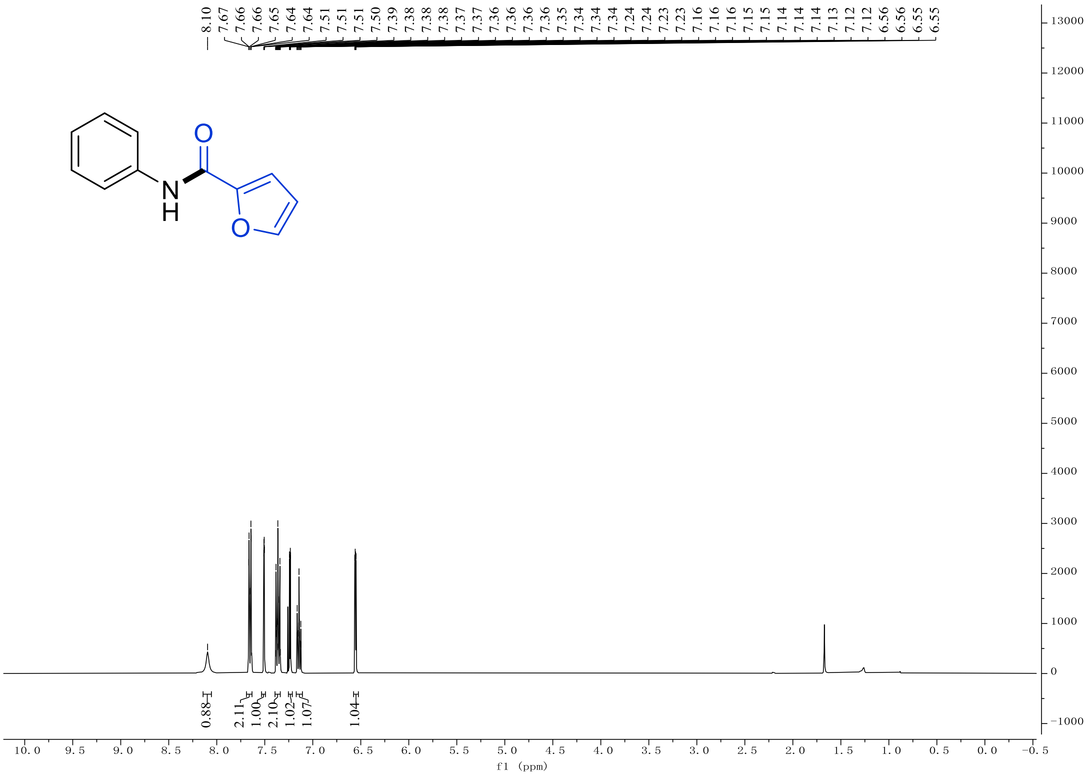

Supplementary Figure 41b | ^{13}C NMR (126 MHz, DMSO-d_6) of *N*-phenylfuran-2-carboxamide **(3x).**

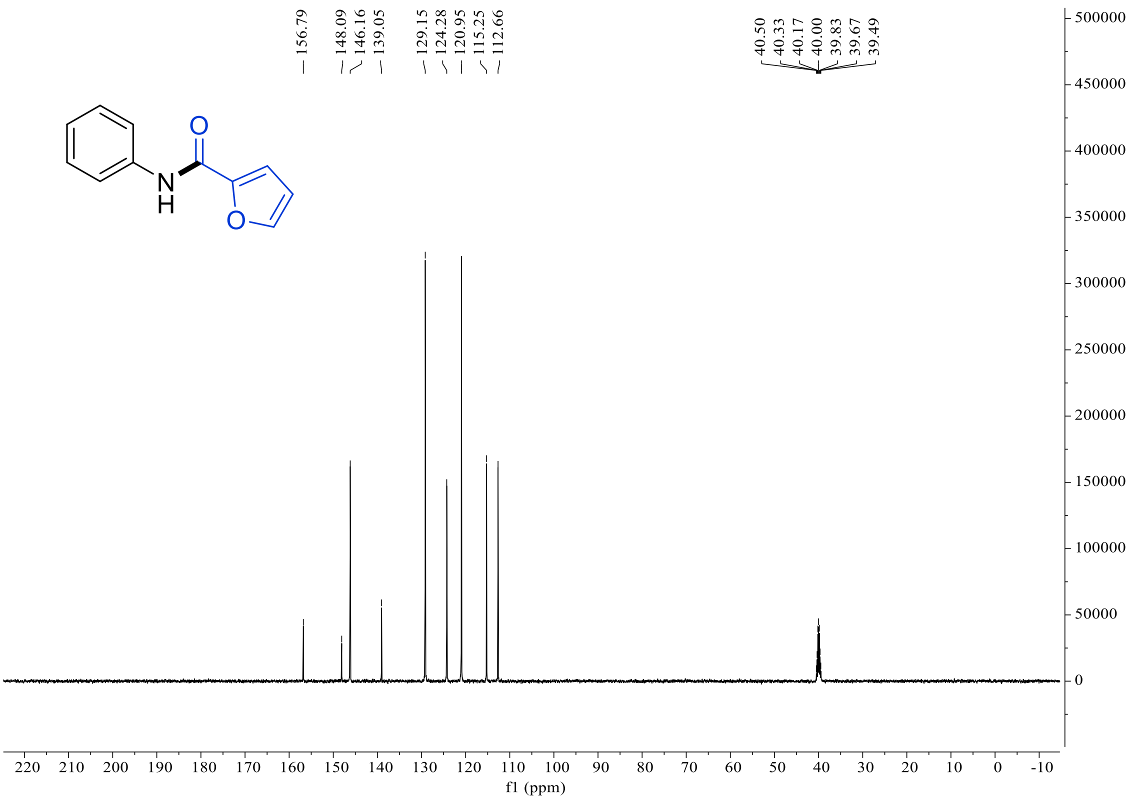

Supplementary Figure 42a | ¹H NMR (400 MHz, Chloroform-*d*) of *N*-phenylisonicotinamide **(3y).**

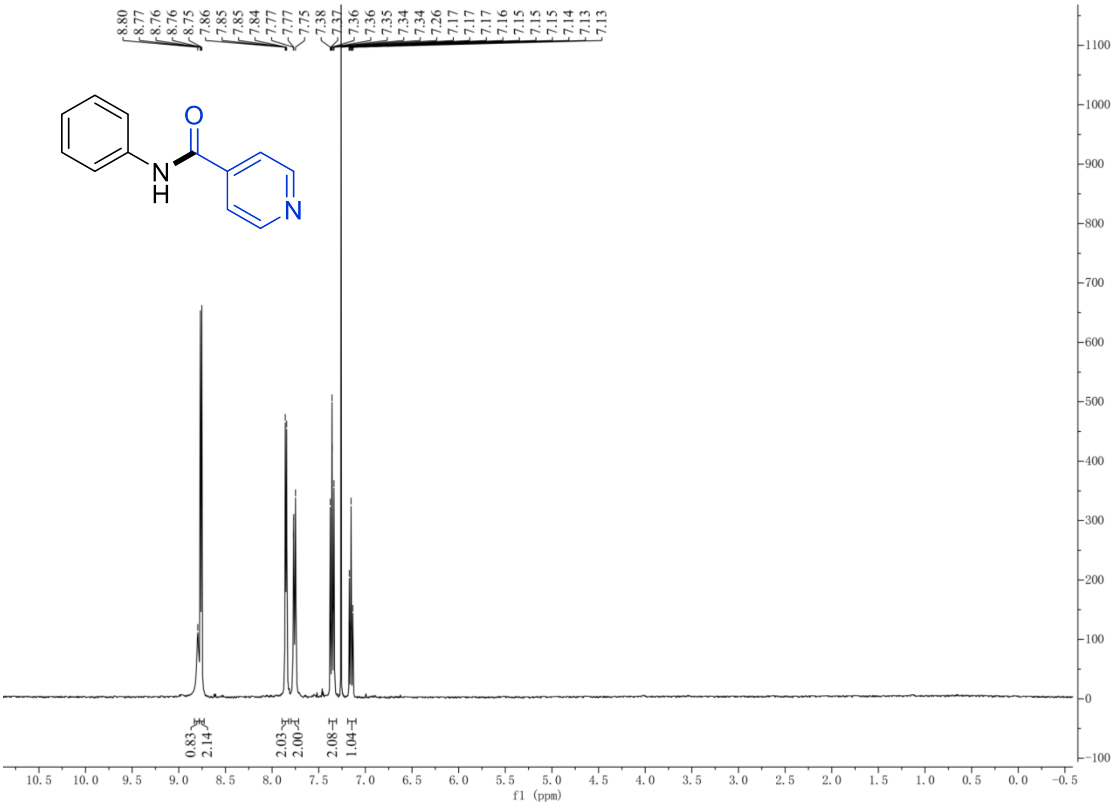

Supplementary Figure 42b | ¹³C NMR (126 MHz, DMSO-d_6) of *N*-phenylisonicotinamide **(3y).**

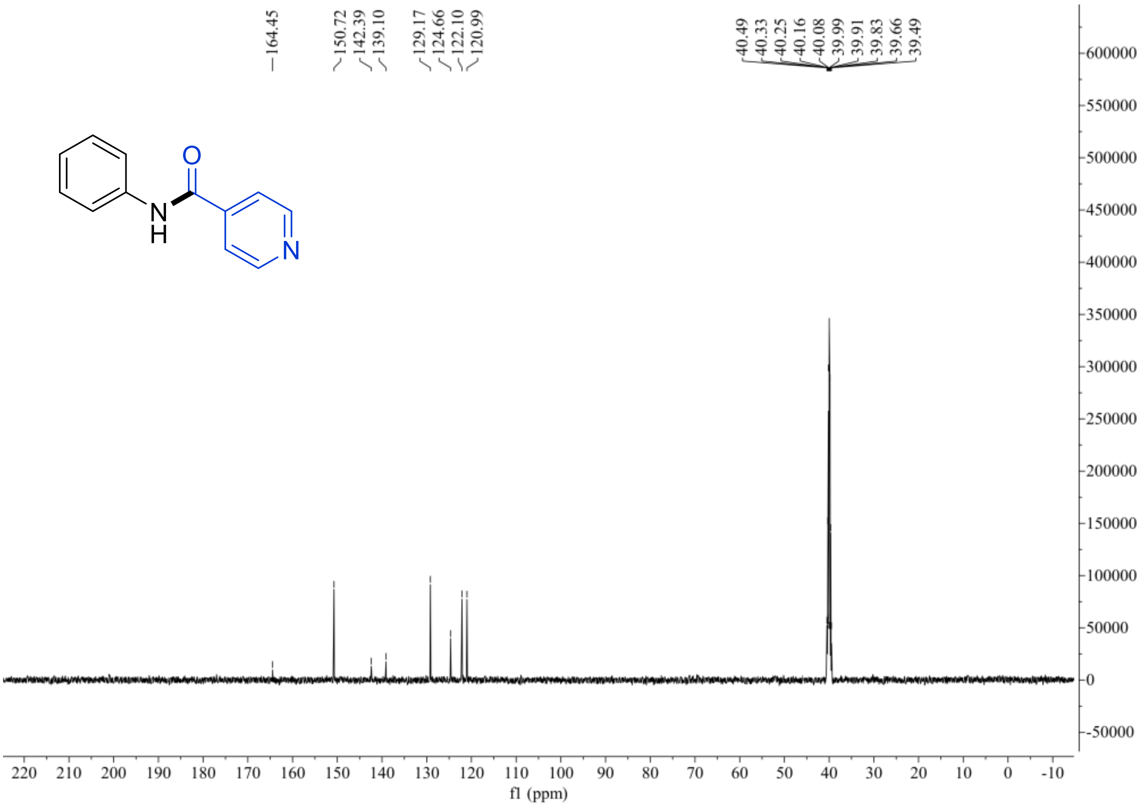

Supplementary Figure 43a | ^{1}H NMR (400 MHz, Chloroform-*d*) of *N*-(4-fluorophenyl)cyclopropanecarboxamide **(3z).**

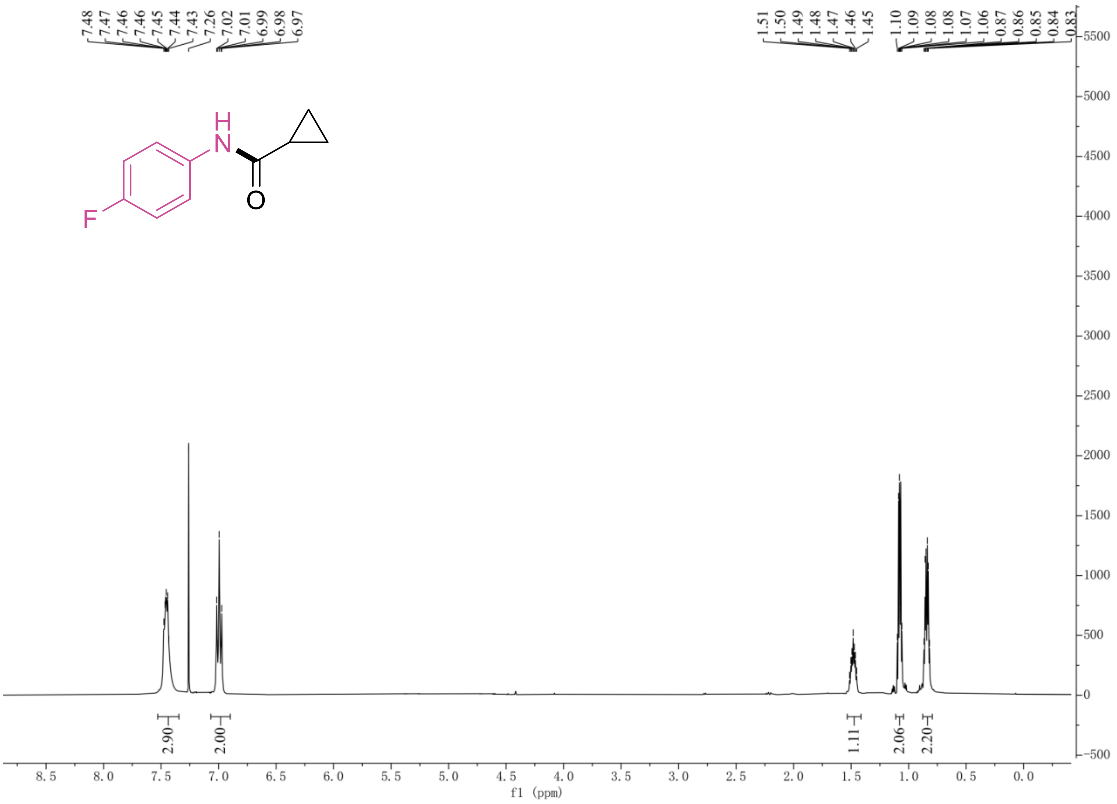

Supplementary Figure 43b | ^{13}C NMR (126 MHz, DMSO-d_6) of *N*-(4-fluorophenyl)cyclopropanecarboxamide **(3z).**

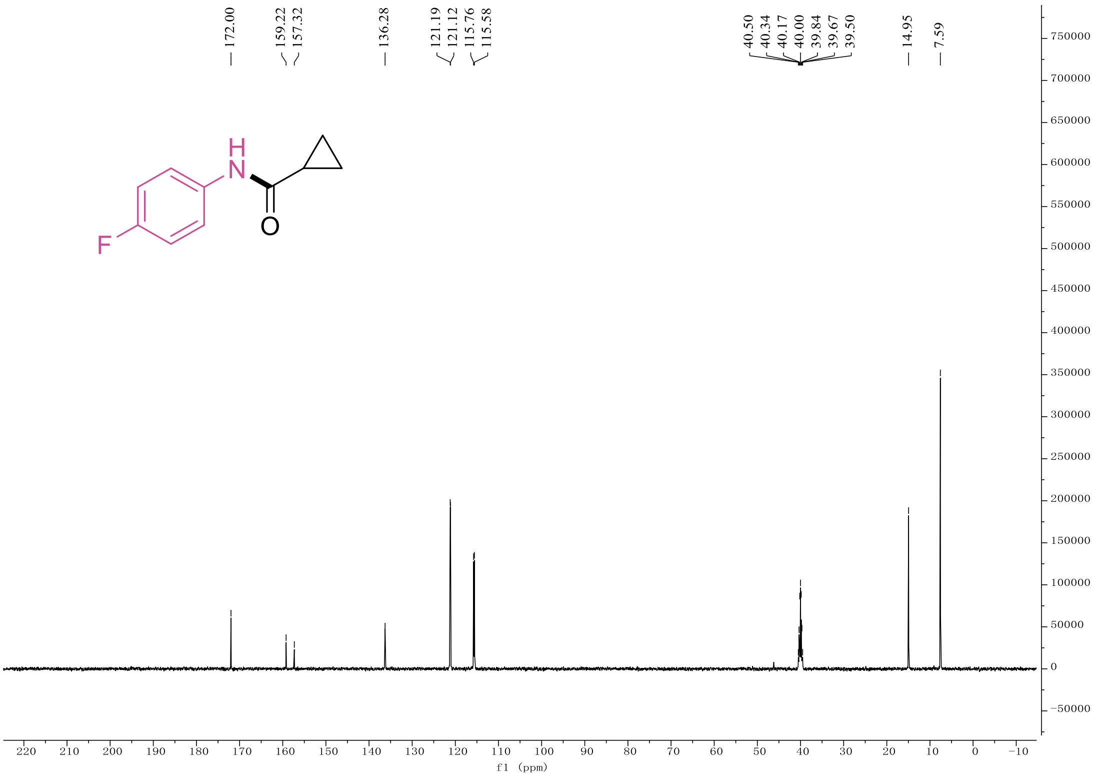

Supplementary Figure 43c | ^{19}F NMR (377 MHz, DMSO-d_6) of *N*-(4-fluorophenyl)cyclopropanecarboxamide **(3z).**

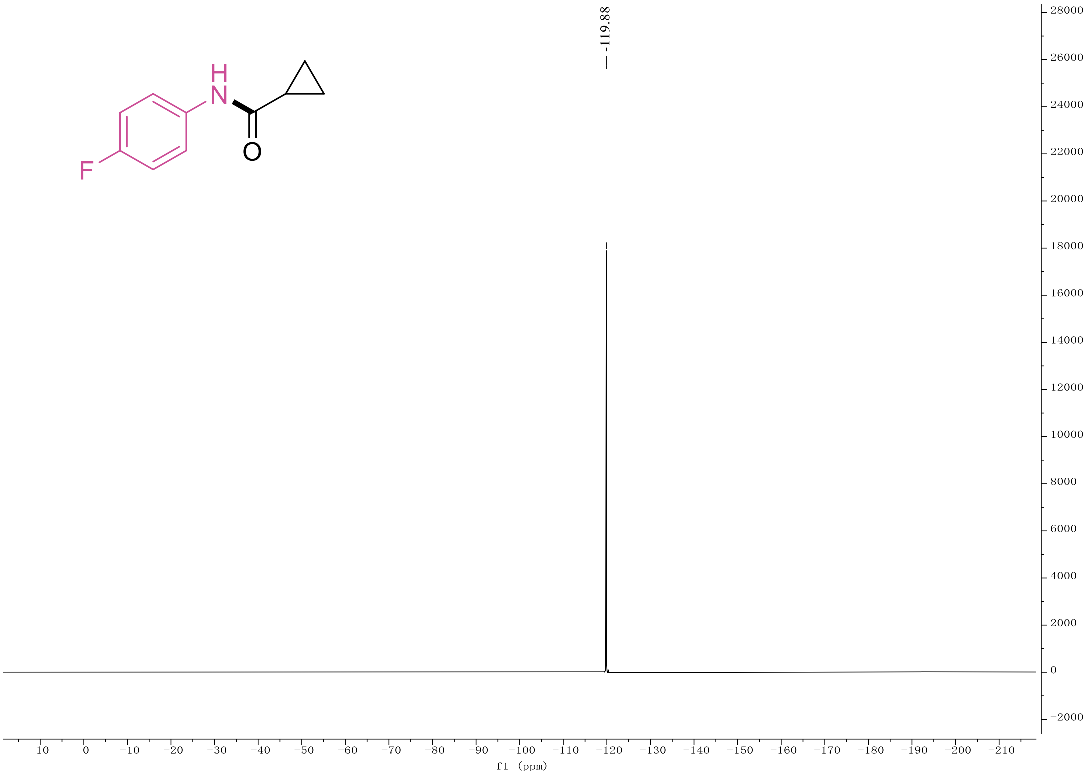

Supplementary Figure 44a | ^{1}H NMR (400 MHz, Chloroform-*d*) of *N*-(4-chlorophenyl)cyclopropanecarboxamide **(3aa).**

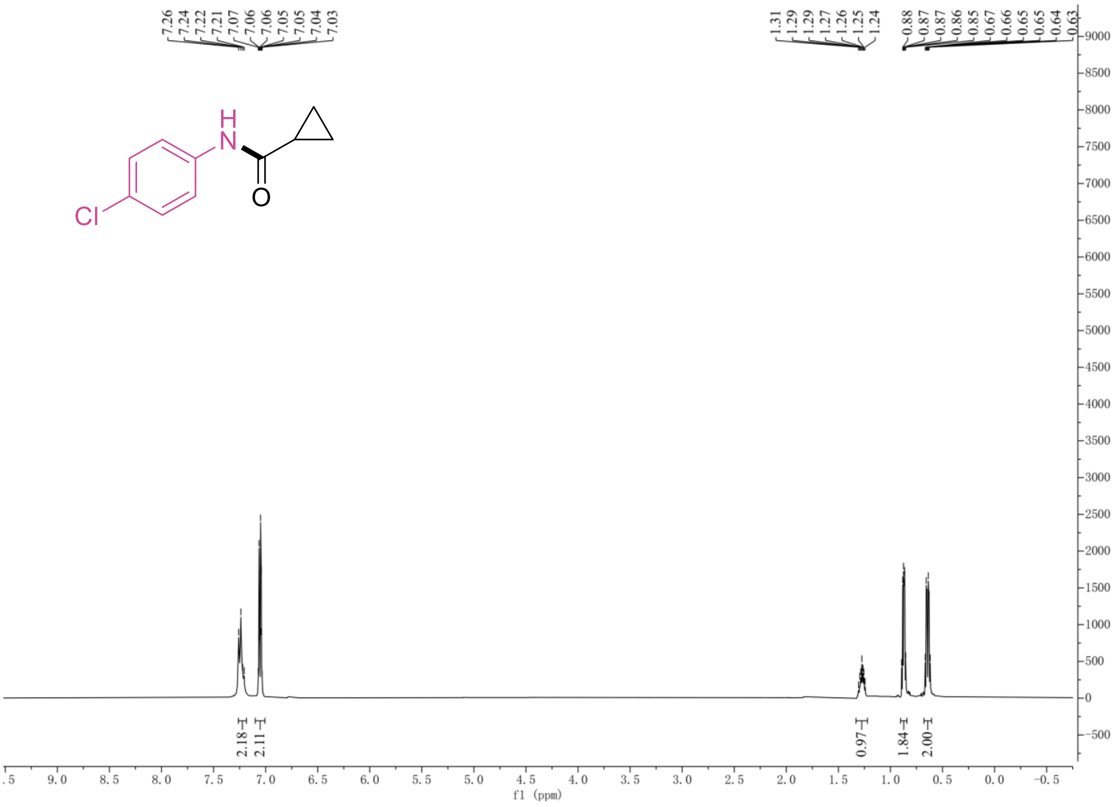

Supplementary Figure 44b | ^{13}C NMR (100 MHz, DMSO-d_6) of *N*-(4-chlorophenyl)cyclopropanecarboxamide **(3aa).**

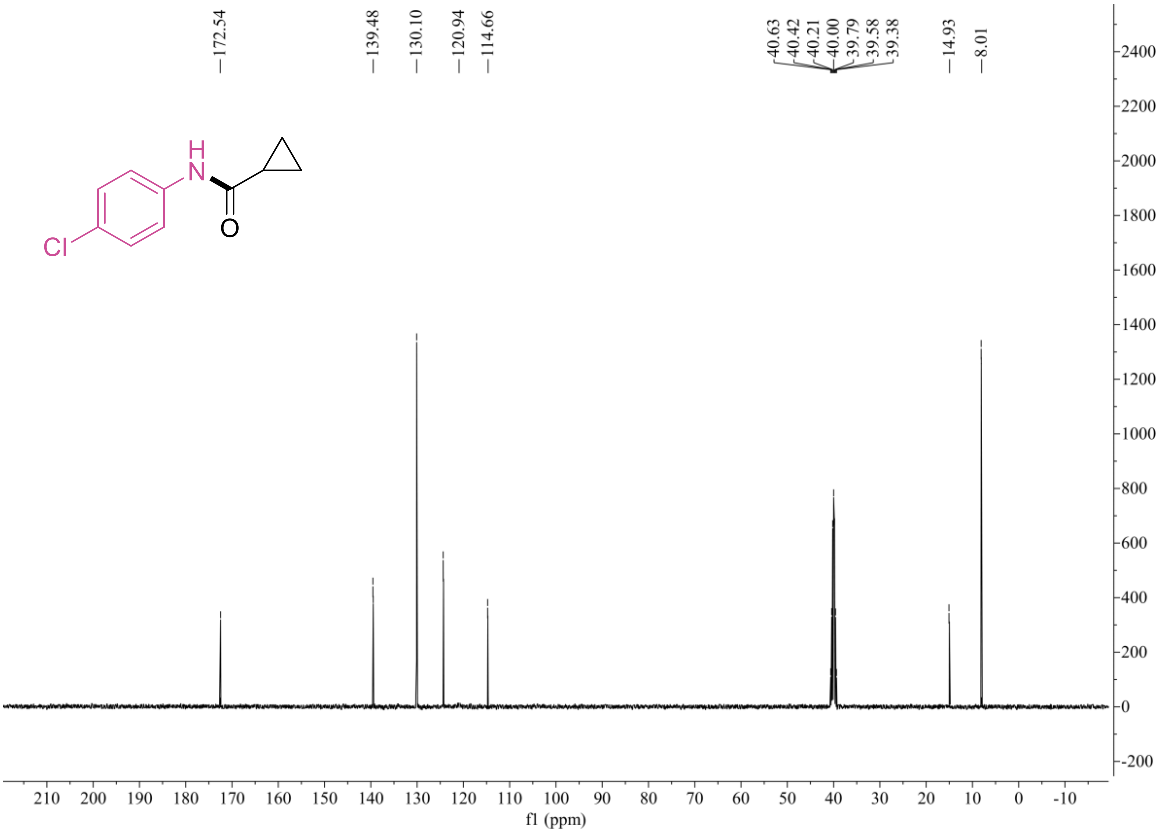

Supplementary Figure 45a | ^{1}H NMR (400 MHz, DMSO-d_6) of *N*-(4-bromophenyl) cyclopropanecarboxamide **(3bb).**

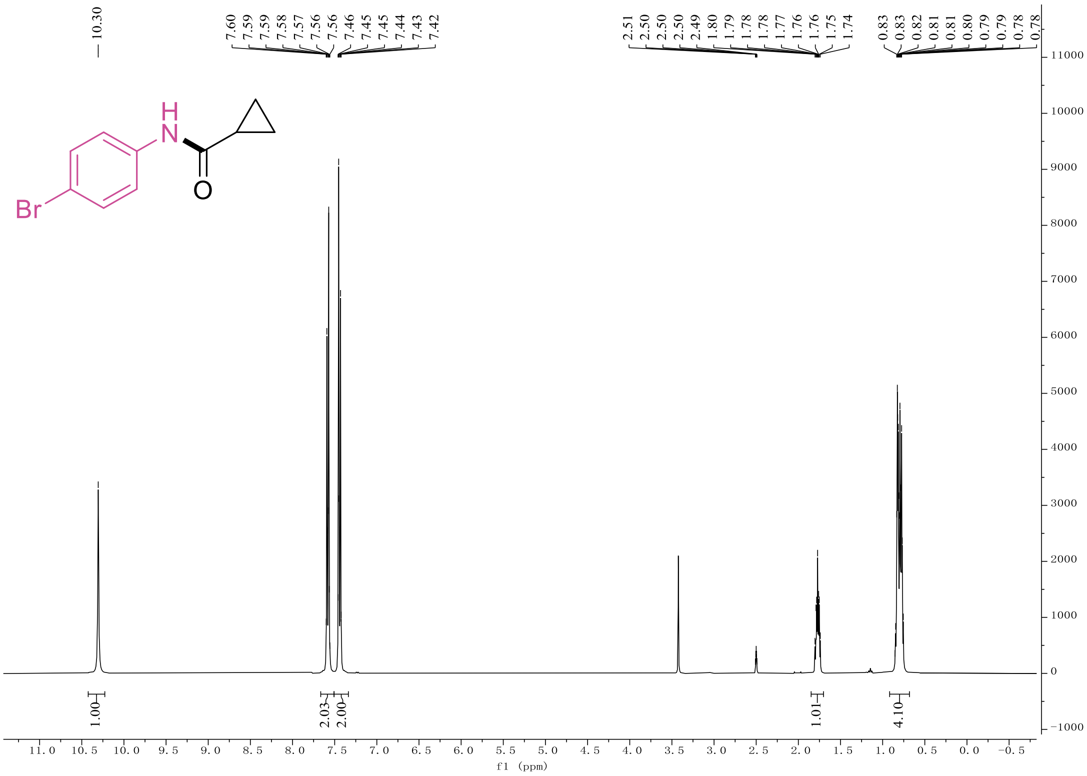

Supplementary Figure 45b | ^{13}C NMR (126 MHz, DMSO-d_6) of *N*-(4-bromophenyl) cyclopropanecarboxamide **(3bb).**

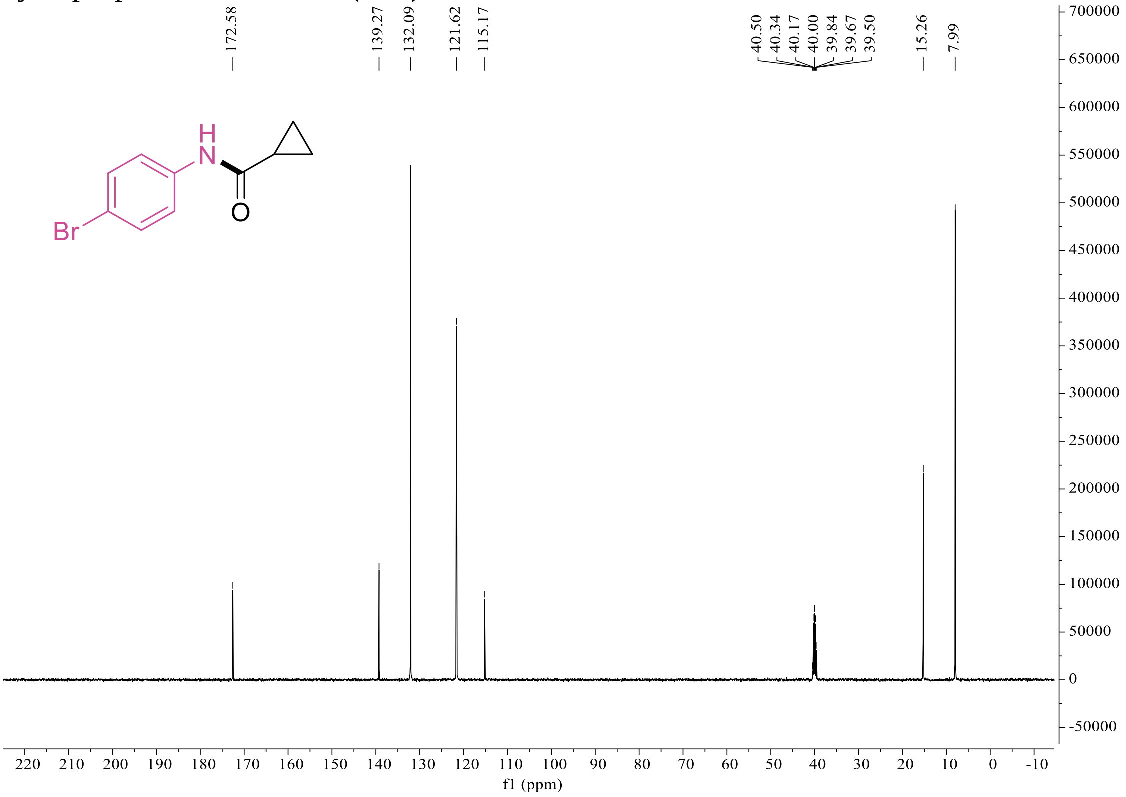

Supplementary Figure 46a | ^{1}H NMR (400 MHz, Chloroform-*d*) of *N*-(4-iodophenyl) cyclopropanecarboxamide **(3cc).**

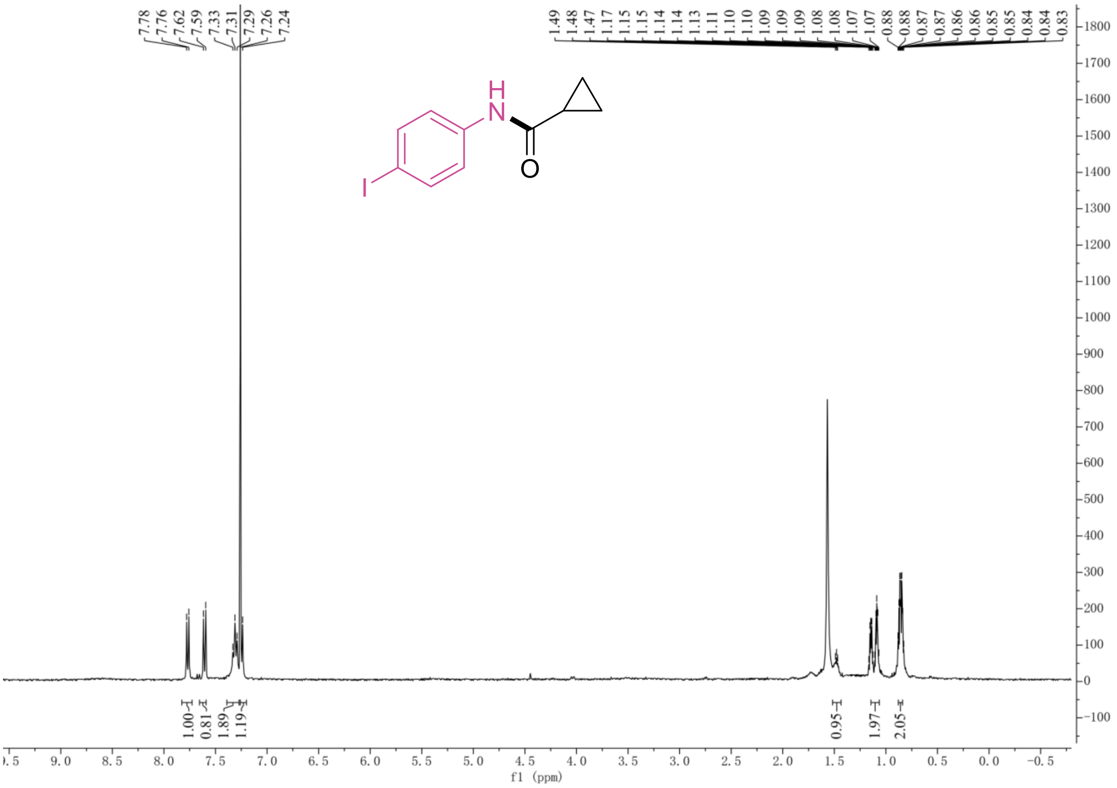

Supplementary Figure 46b | ^{13}C NMR (126 MHz, DMSO-d_6) of *N*-(4-iodophenyl) cyclopropanecarboxamide **(3cc).**

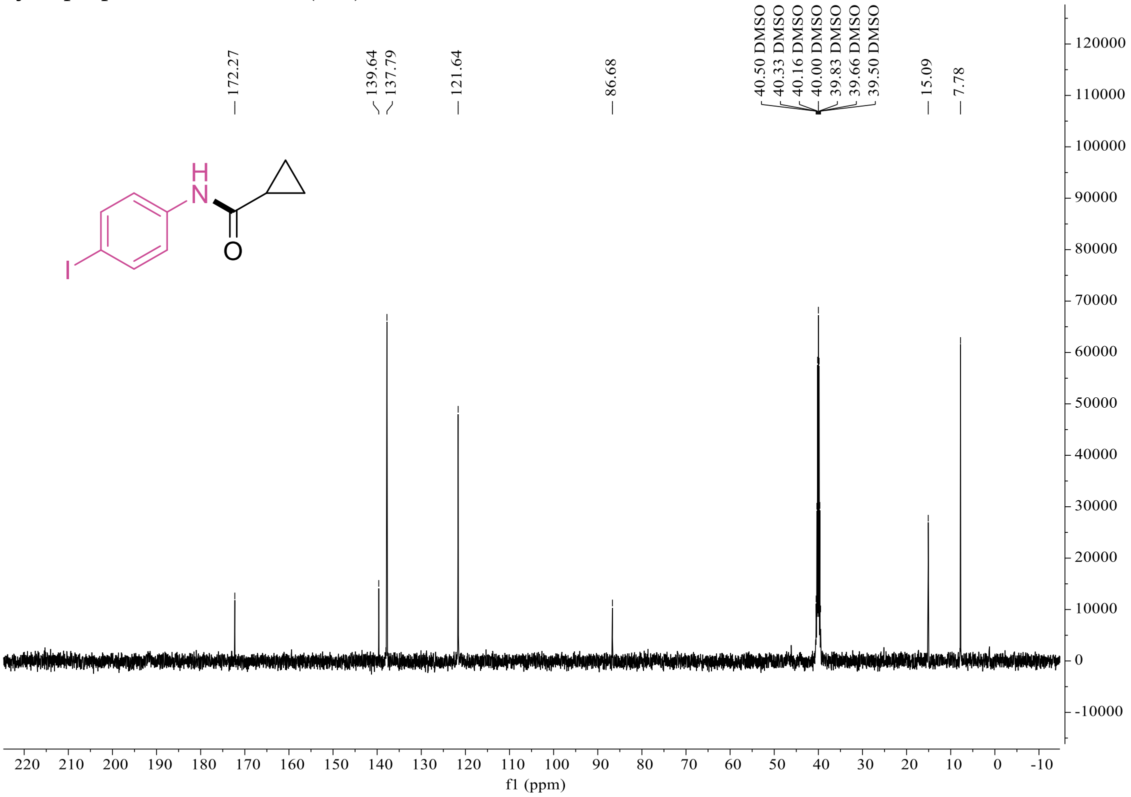

Supplementary Figure 47a | ^{1}H NMR (400 MHz, Chloroform-*d*) of *N*-(2-fluorophenyl) cyclopropanecarboxamide **(3dd)**

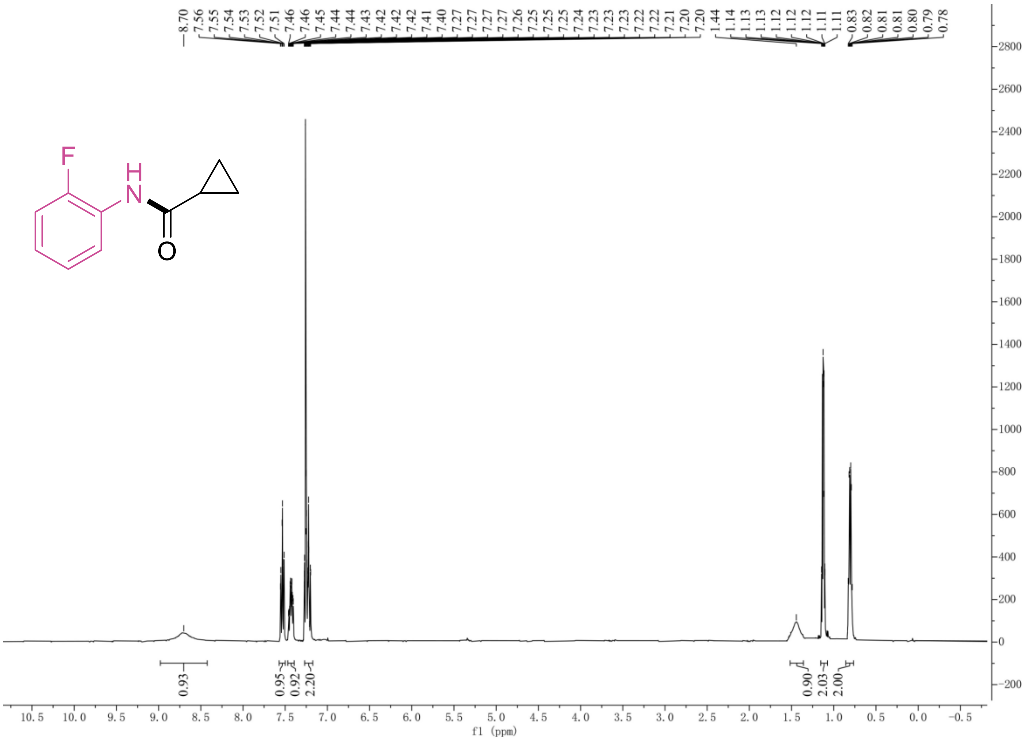

Supplementary Figure 47b | ^{13}C NMR (126 MHz, DMSO-d_6) of *N*-(2-fluorophenyl) cyclopropanecarboxamide **(3dd)**

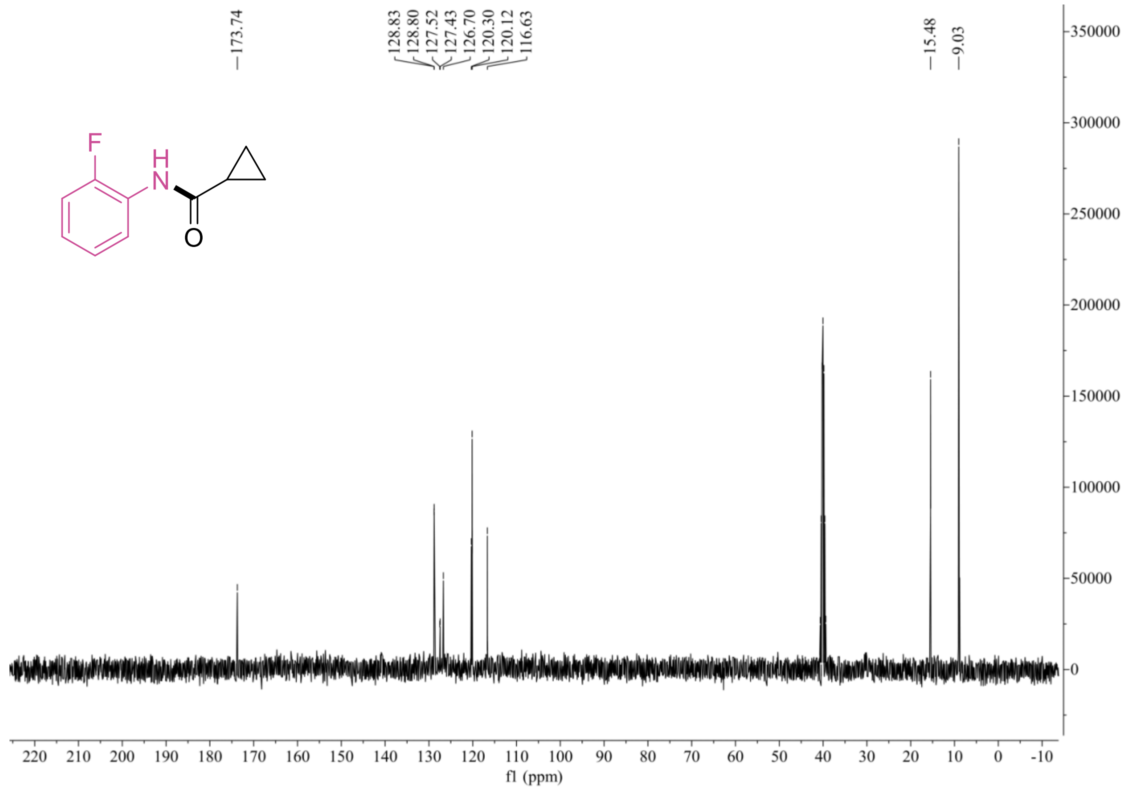

Supplementary Figure 48a | ^{1}H NMR (400 MHz, DMSO-d_6) of *N*-(4-bromo-2-fluorophenyl) cyclopropanecarboxamide **(3ee).**

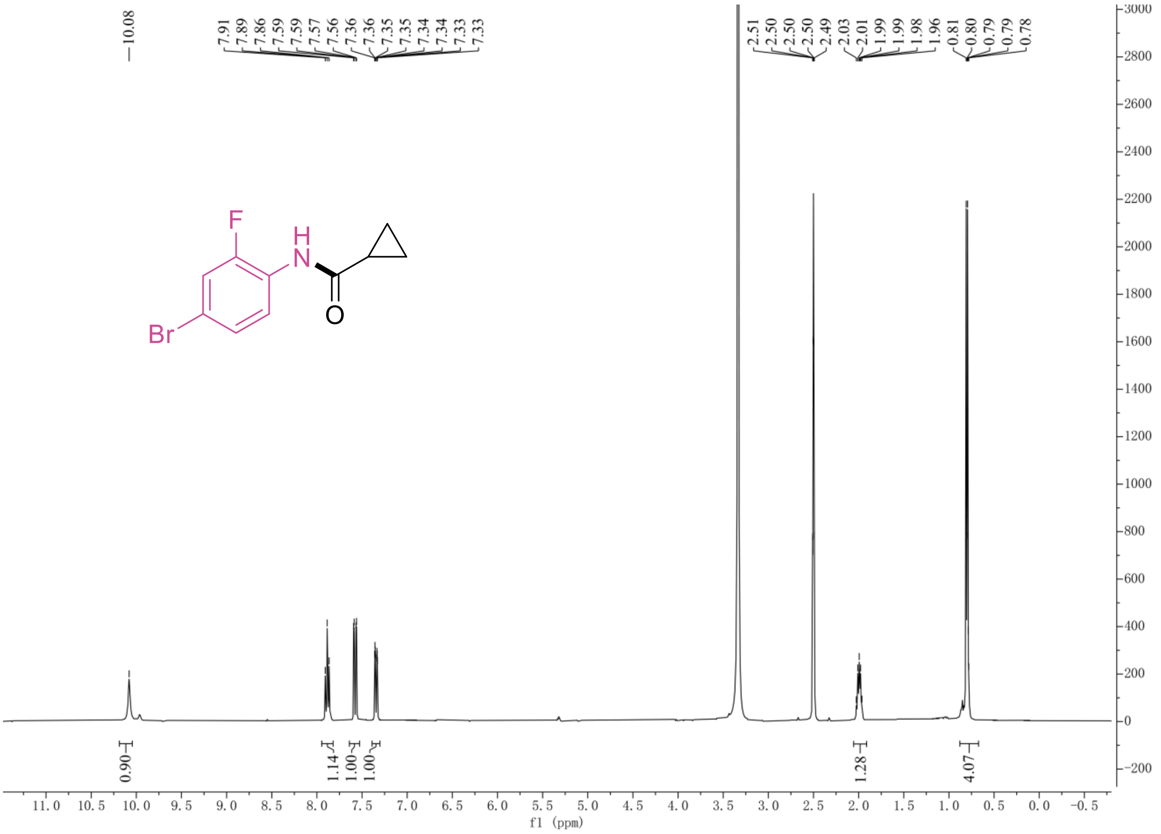

Supplementary Figure 48b | ^{13}C NMR (126 MHz, DMSO-d_6) of *N*-(4-bromo-2-fluorophenyl) cyclopropanecarboxamide **(3ee).**

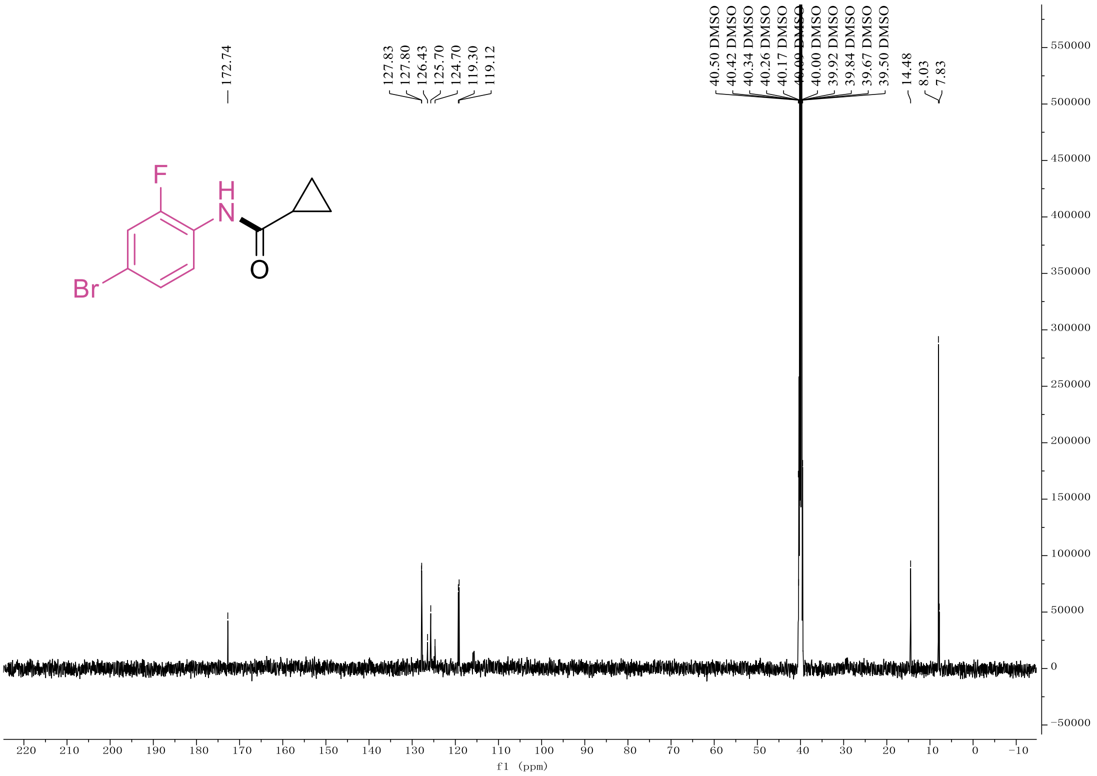

Supplementary Figure 48c | ^{19}F NMR (377 MHz, DMSO-d_6) of *N*-(4-bromo-2-fluorophenyl)cyclopropanecarboxamide **(3ee).**

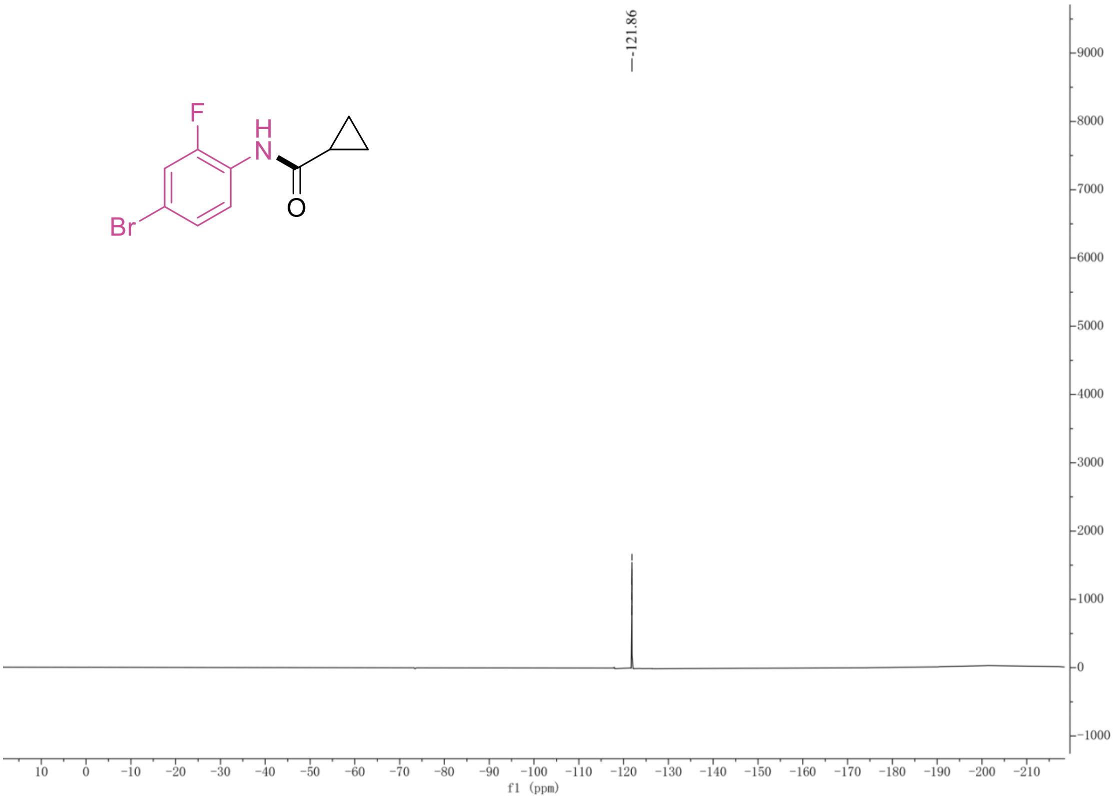

Supplementary Figure 49a | ^{1}H NMR (400 MHz, Chloroform-*d*) of *N*-(4-chloro-2-(trifluoromethyl)phenyl)cyclopropanecarboxamide **(3ff).**

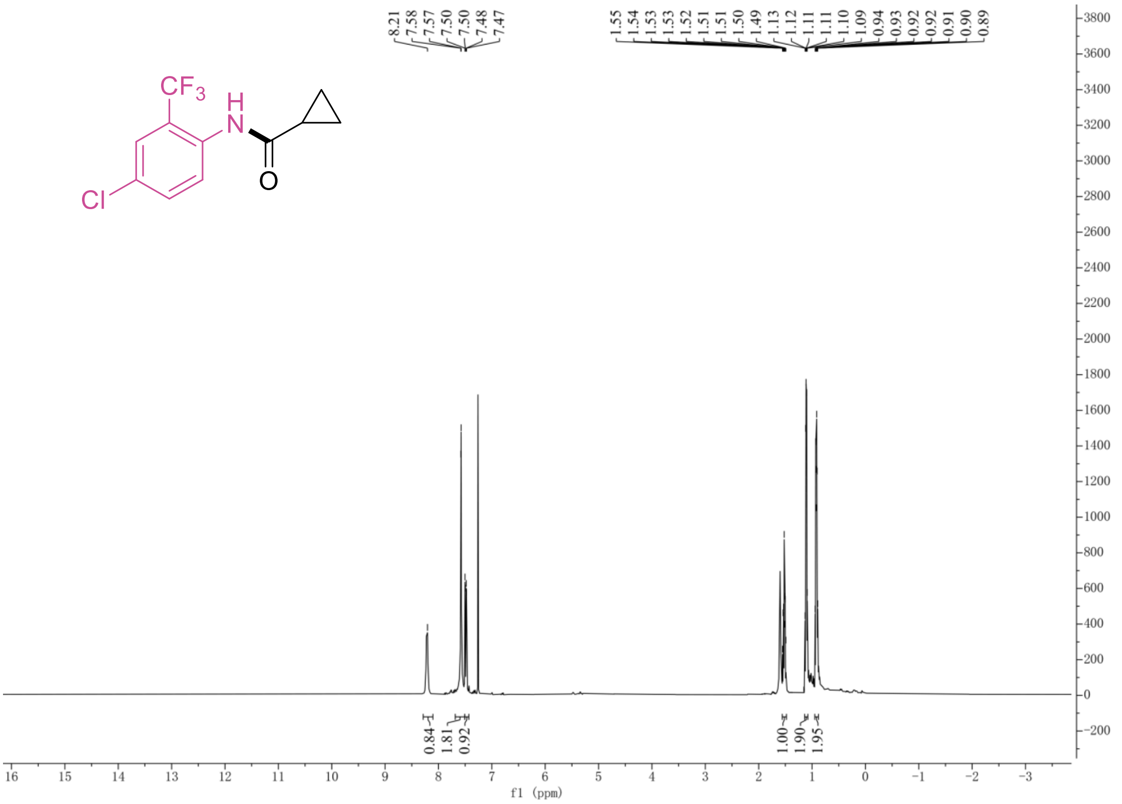

Supplementary Figure 49b | ^{13}C NMR (126 MHz, DMSO-*d*$_6$) of *N*-(4-chloro-2-(trifluoromethyl)phenyl)cyclopropanecarboxamide **(3ff).**

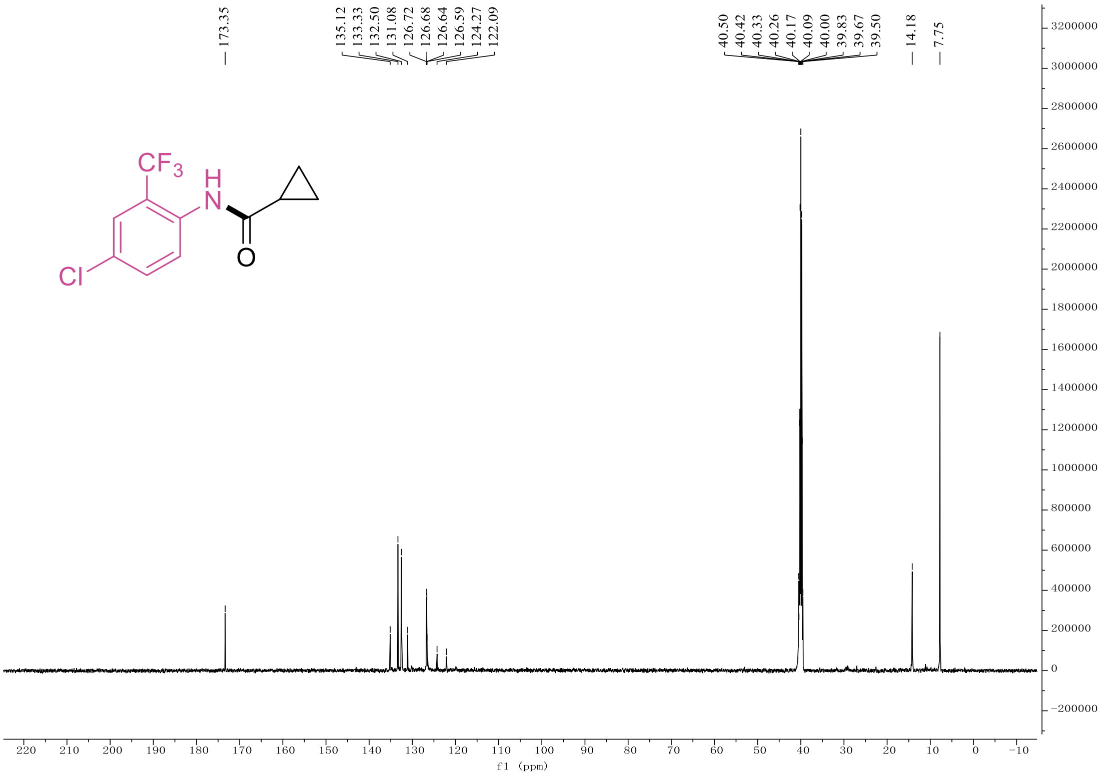

Supplementary Figure 49c | ^{19}F NMR (377 MHz, DMSO-d_6) of *N*-(4-chloro-2-(trifluoromethyl)phenyl)cyclopropanecarboxamide **(3ff).**

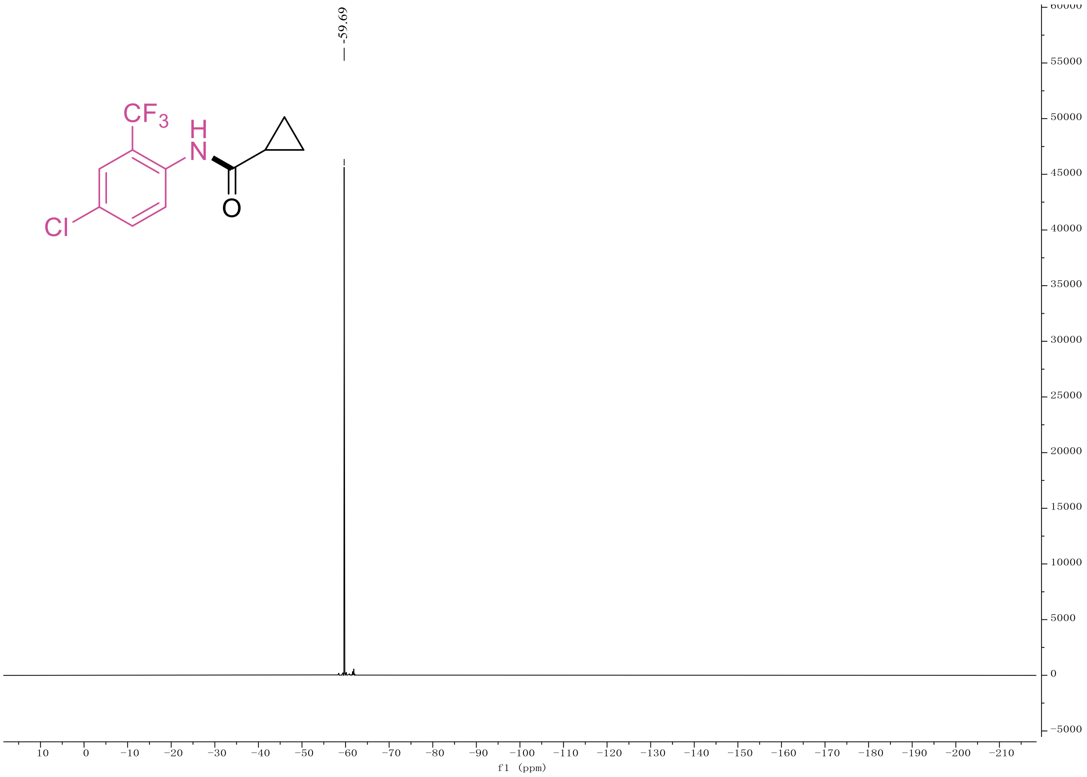

Supplementary Figure 50a | ^{1}H NMR (400 MHz, Chloroform-*d*) of *N*-(4-benzoylphenyl)cyclopropanecarboxamide **(3gg).**

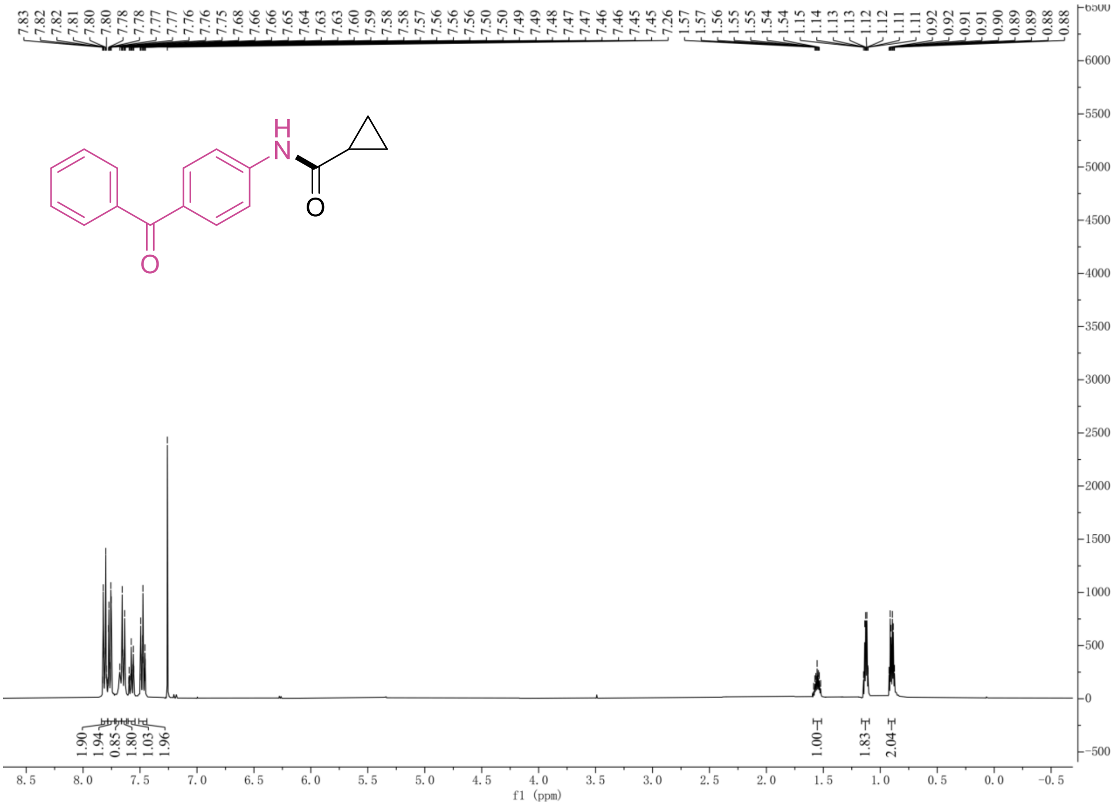

Supplementary Figure 50b | ^{13}**C NMR** (126 MHz, DMSO-d_6) of *N*-(4-benzoylphenyl)cyclopropanecarboxamide **(3gg).**

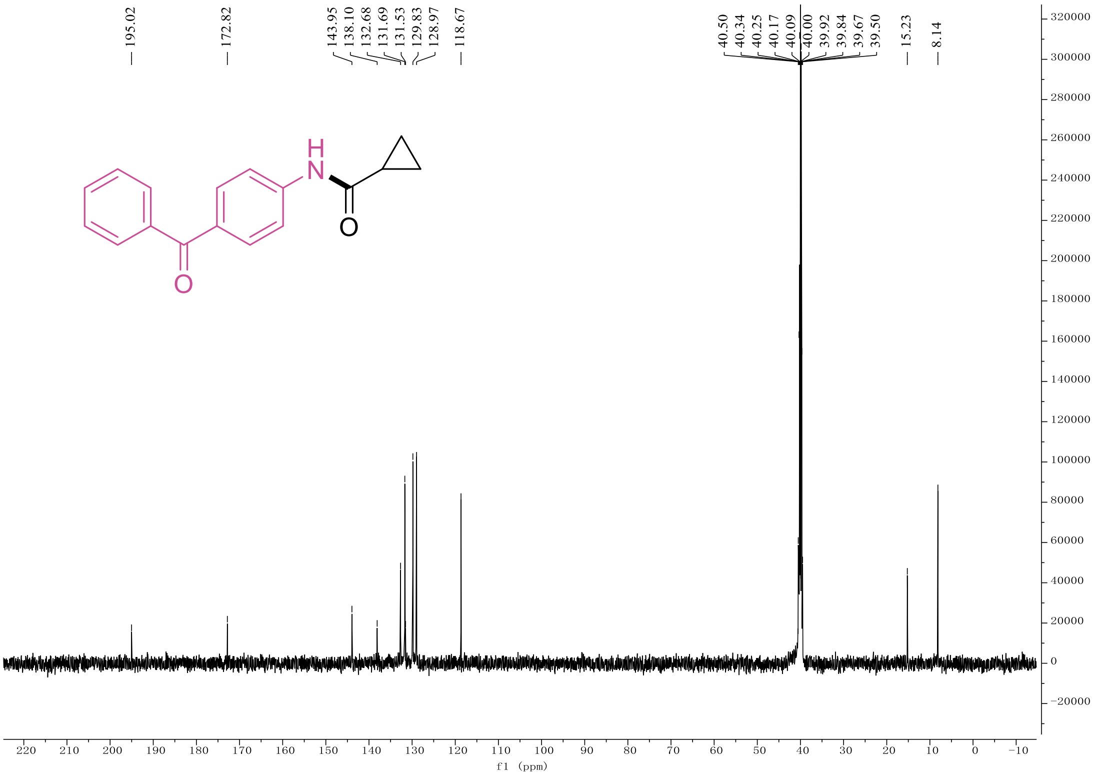

Supplementary Figure 51a |^{1}**H NMR** (400 MHz, Chloroform-*d*) of *N*-(4-formylphenyl)cyclopropanecarboxamide **(3hh).**

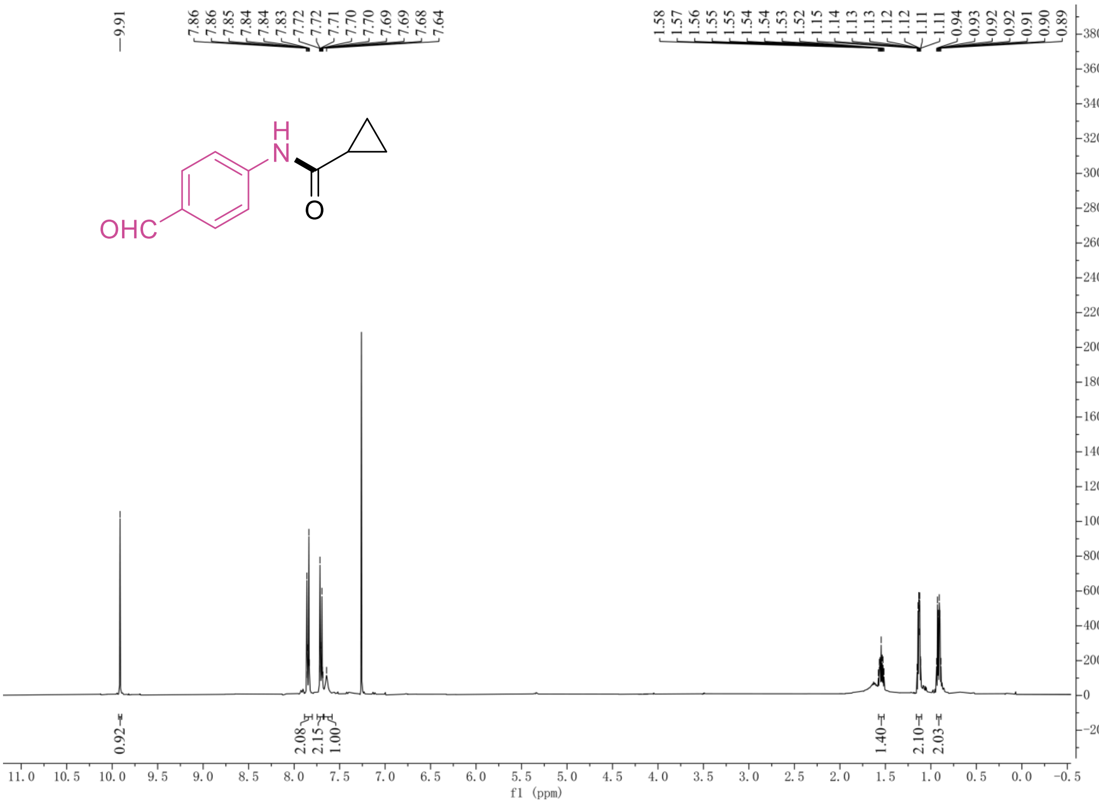

Supplementary Figure 51b | ^{13}C NMR (126 MHz, DMSO-d_6) of *N*-(4-formylphenyl)cyclopropanecarboxamide **(3hh).**

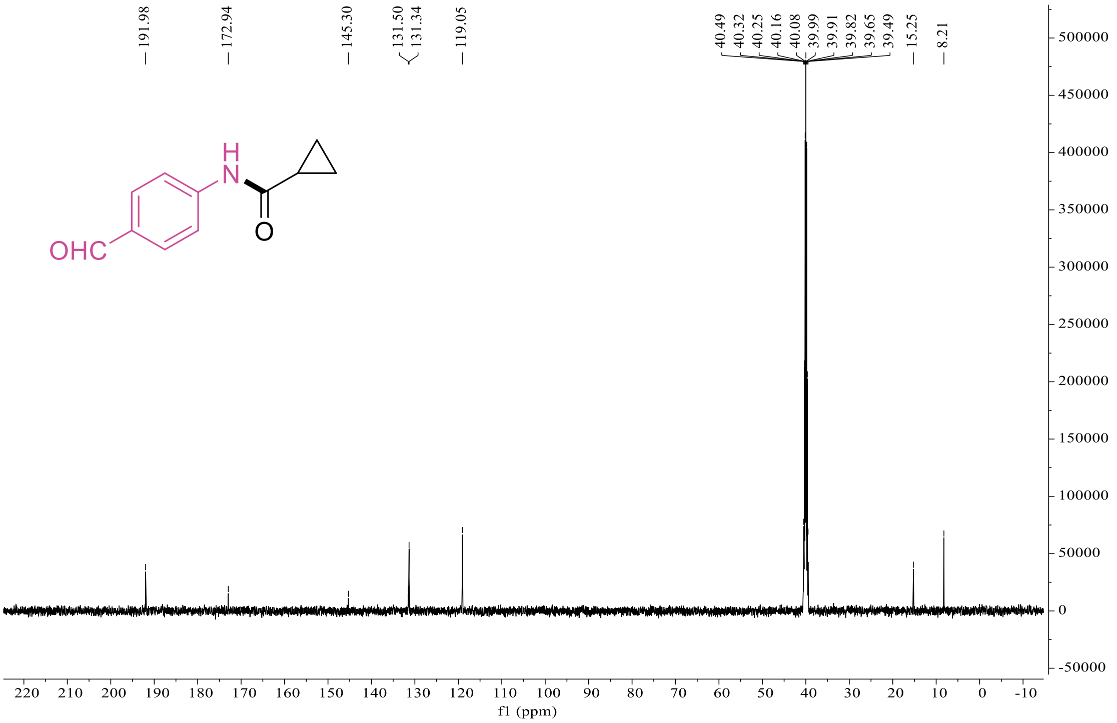

Supplementary Figure 52a | ^{1}H NMR (400 MHz, Chloroform-*d*) of *N*-(p-tolyl)cyclopropanecarboxamide **(3ii).**

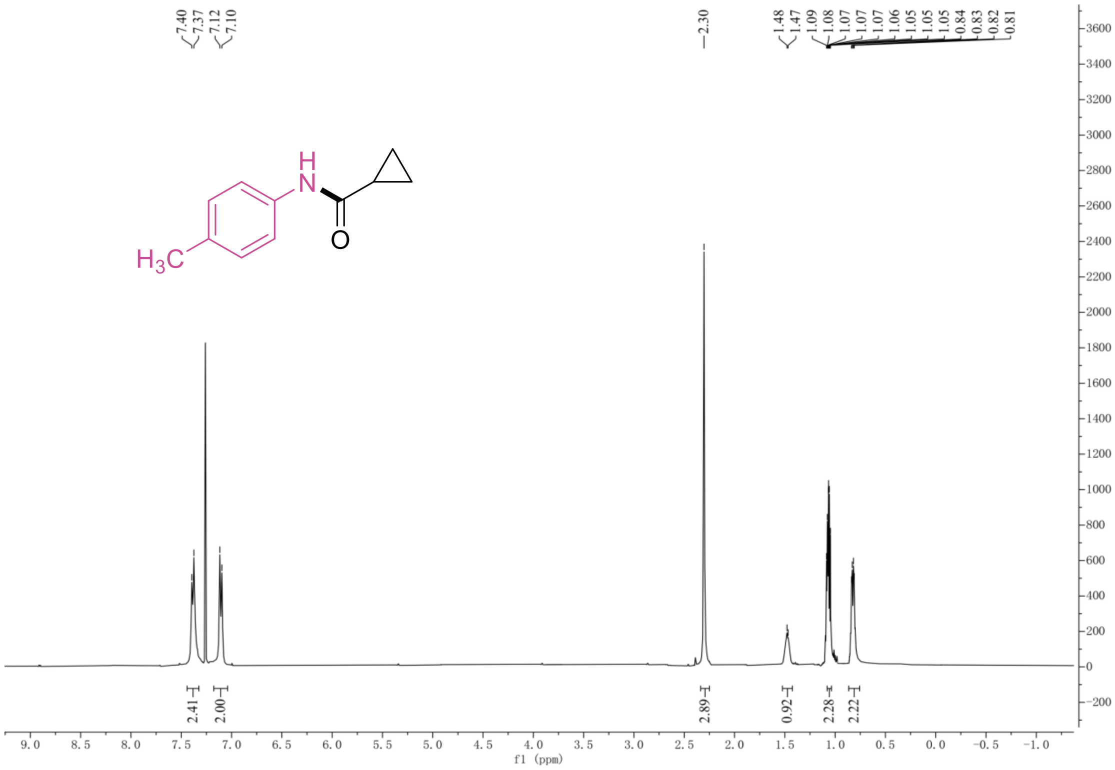

Supplementary Figure 52b | ^{13}C NMR (126 MHz, DMSO-d_6) of *N*-(p-tolyl)cyclopropanecarboxamide **(3ii).**

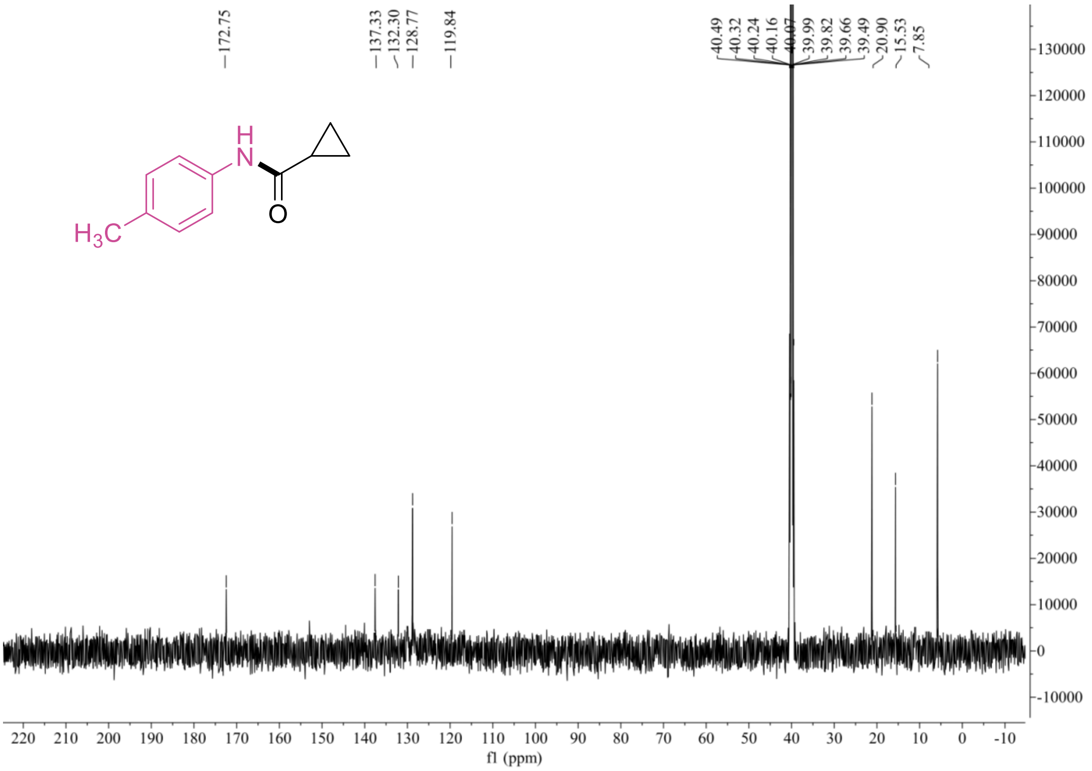

Supplementary Figure 53a | ^{1}H NMR (400 MHz, Chloroform-*d*) of *N*-(4-(cyanomethyl)phenyl)cyclopropanecarboxamide **(3jj).**

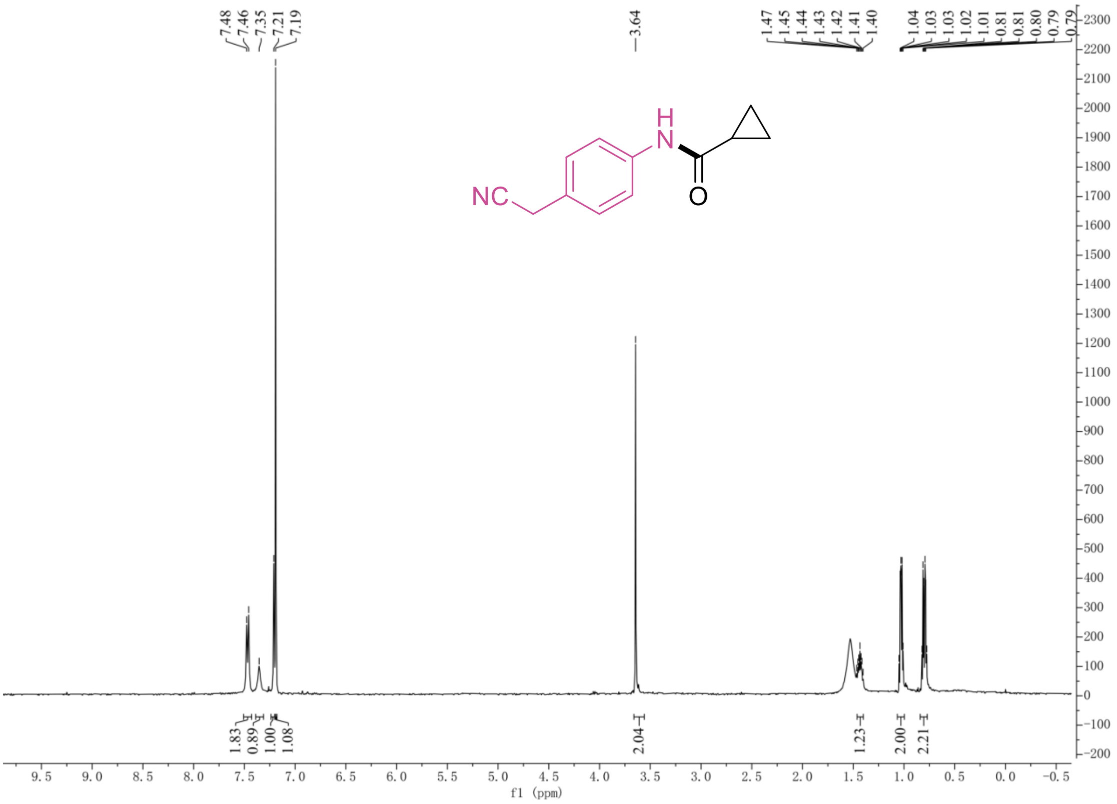

Supplementary Figure 53b | ^{13}C NMR (126 MHz, DMSO-d_6) of *N*-(4-(cyanomethyl)phenyl)cyclopropanecarboxamide **(3jj).**

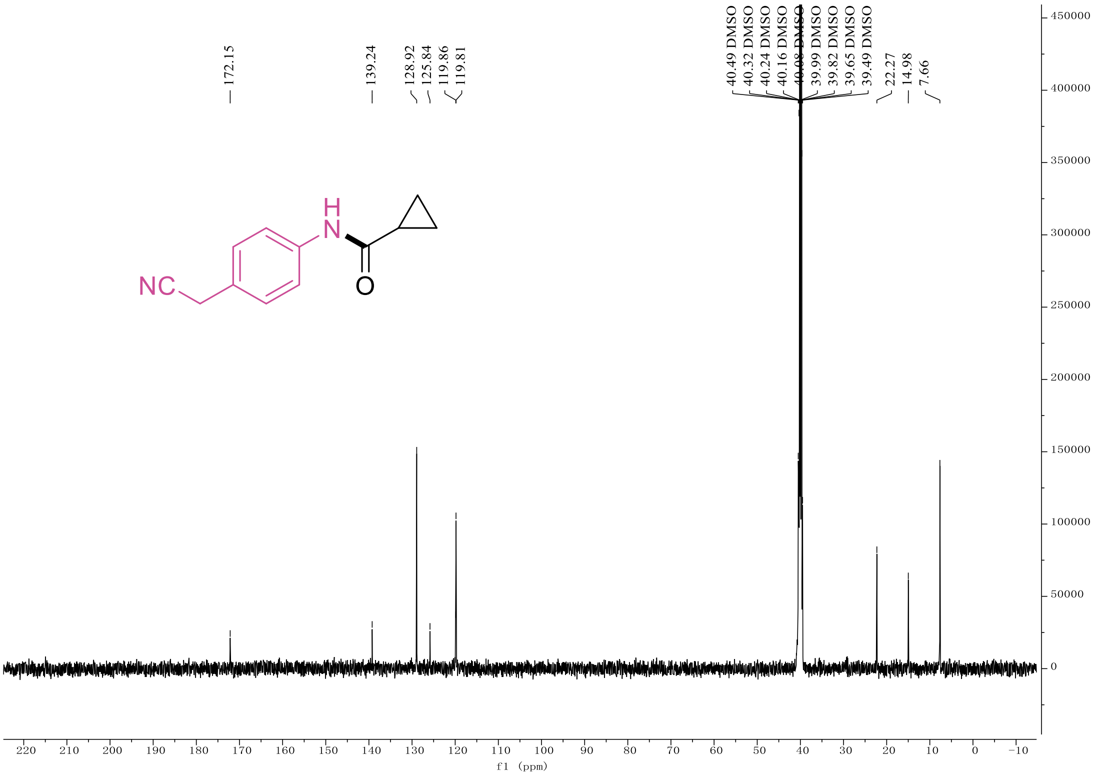

Supplementary Figure 54a | ^{1}H NMR (400 MHz, Chloroform-*d*) of *N*-(4-hydroxyphenyl)cyclopropanecarboxamide **(3kk).**

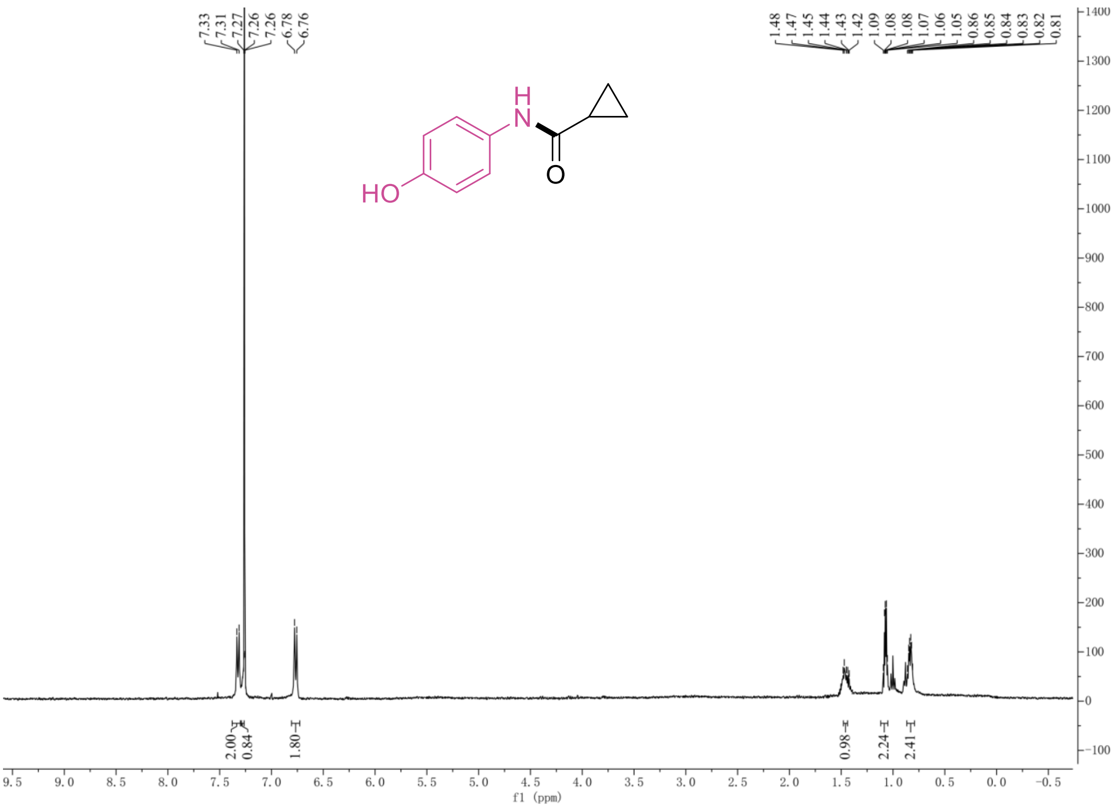

Supplementary Figure 54b | ^{13}C NMR (126 MHz, DMSO-d_6) of *N*-(4-hydroxyphenyl)cyclopropanecarboxamide **(3kk).**

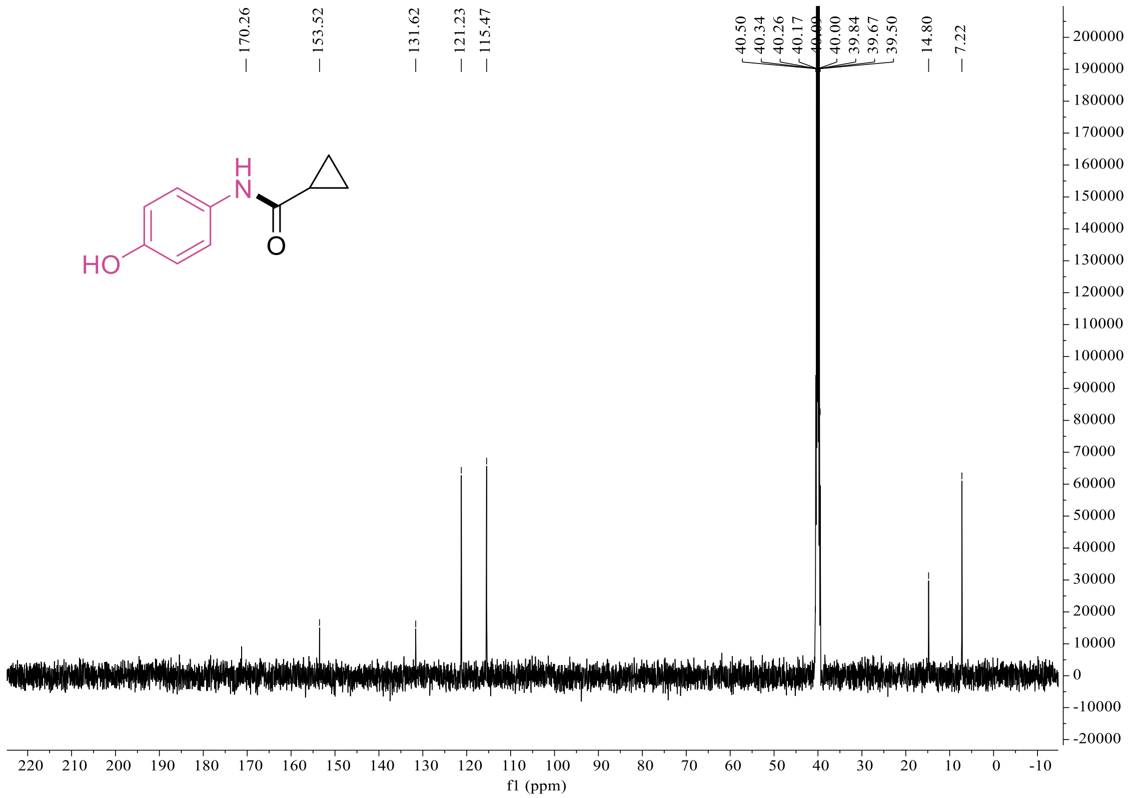

Supplementary Figure 55a | ^{1}H NMR (400 MHz, Chloroform-*d*) of *N*-(2-hydroxyphenyl)cyclopropanecarboxamide **(3ll).**

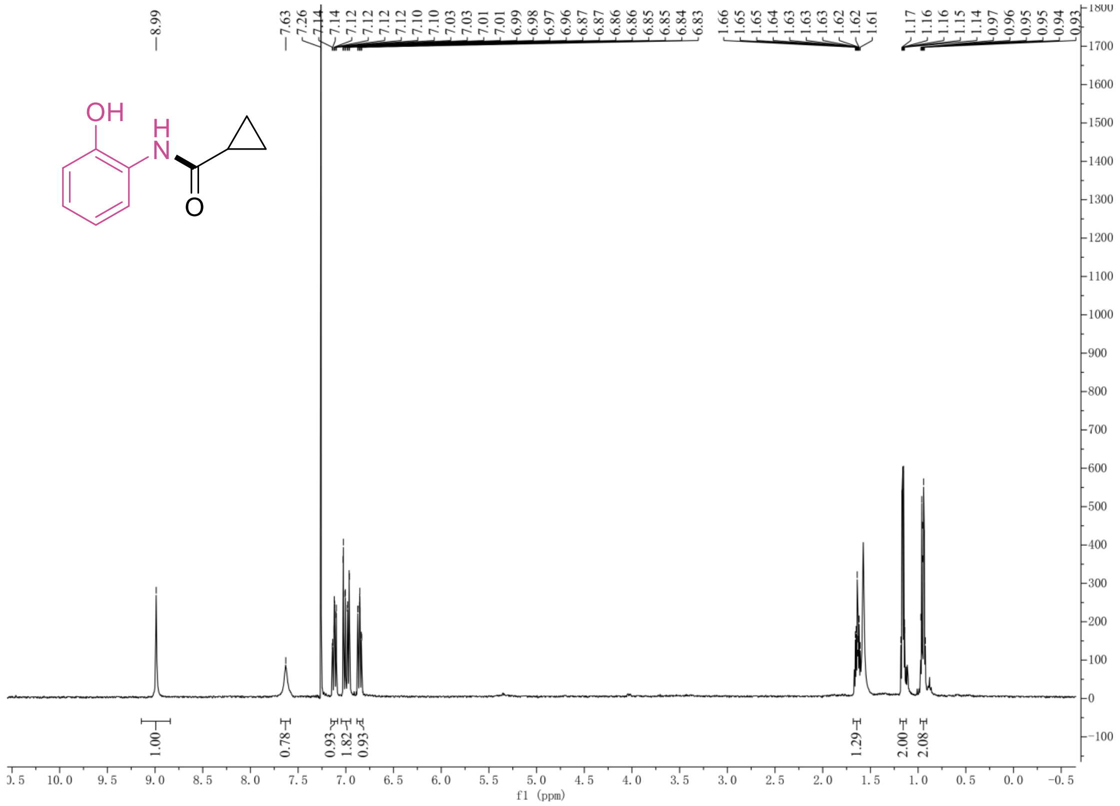

Supplementary Figure 55b |^{13}C NMR (126 MHz, DMSO-d_6) of *N*-(2-hydroxyphenyl)cyclopropanecarboxamide **(3ll).**

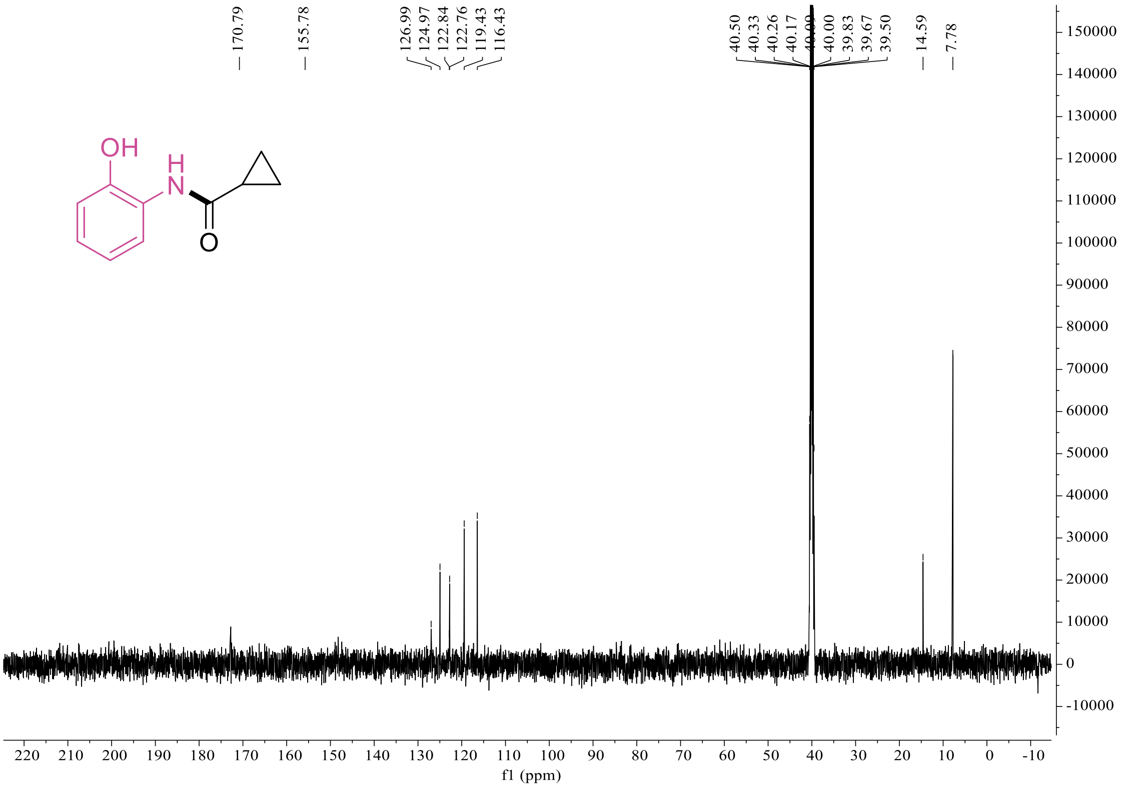

Supplementary Figure 56a |^{1}H NMR (400 MHz, Chloroform-*d*) of *N*-(4-methoxyphenyl)cyclopropanecarboxamide **(3mm).**

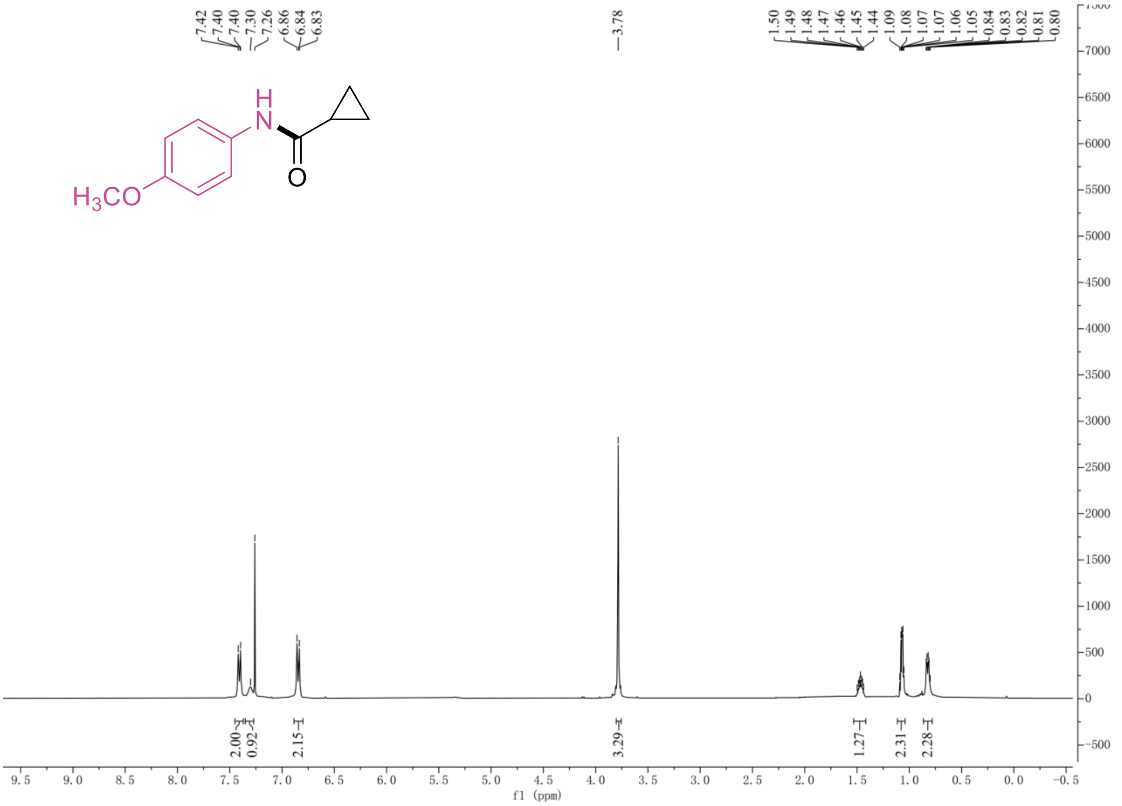

Supplementary Figure 56b | ^{13}C NMR (126 MHz, DMSO-d_6) of *N*-(4-methoxyphenyl)cyclopropanecarboxamide **(3mm).**

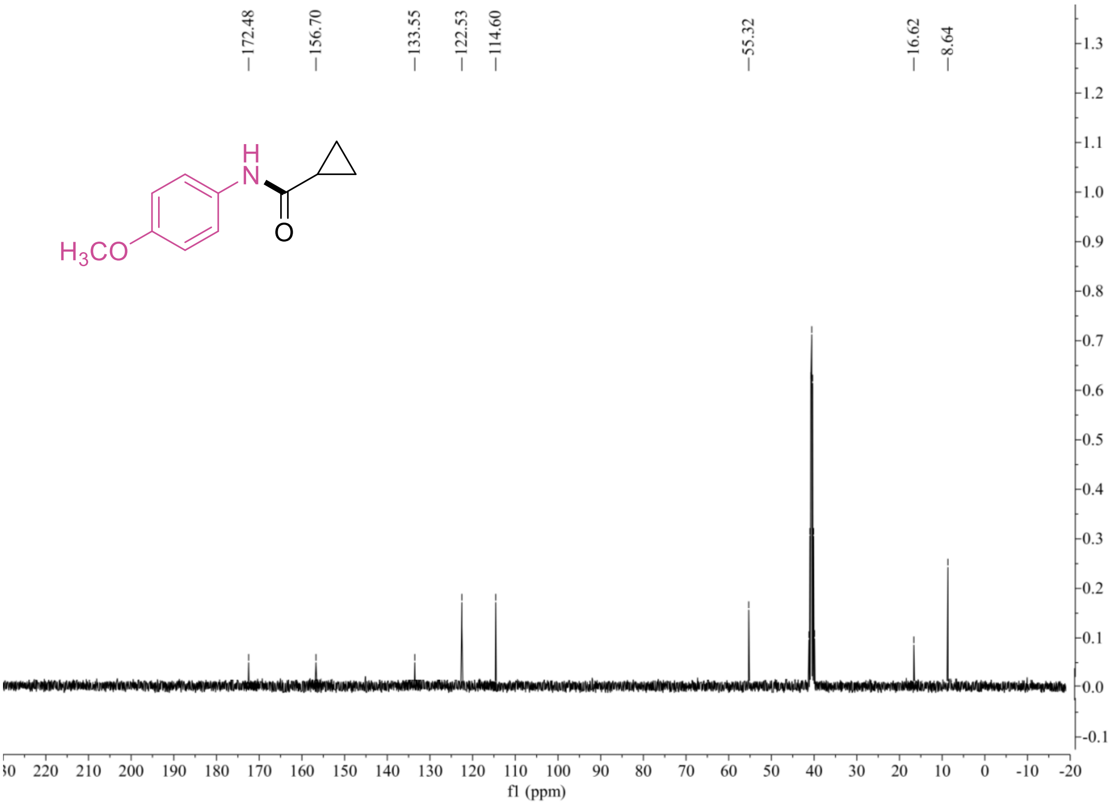

Supplementary Figure 57a | ^{1}H NMR (400 MHz, Chloroform-*d*) of 4-(cyclopropanecarboxamido)phenyl acetate **(3nn).**

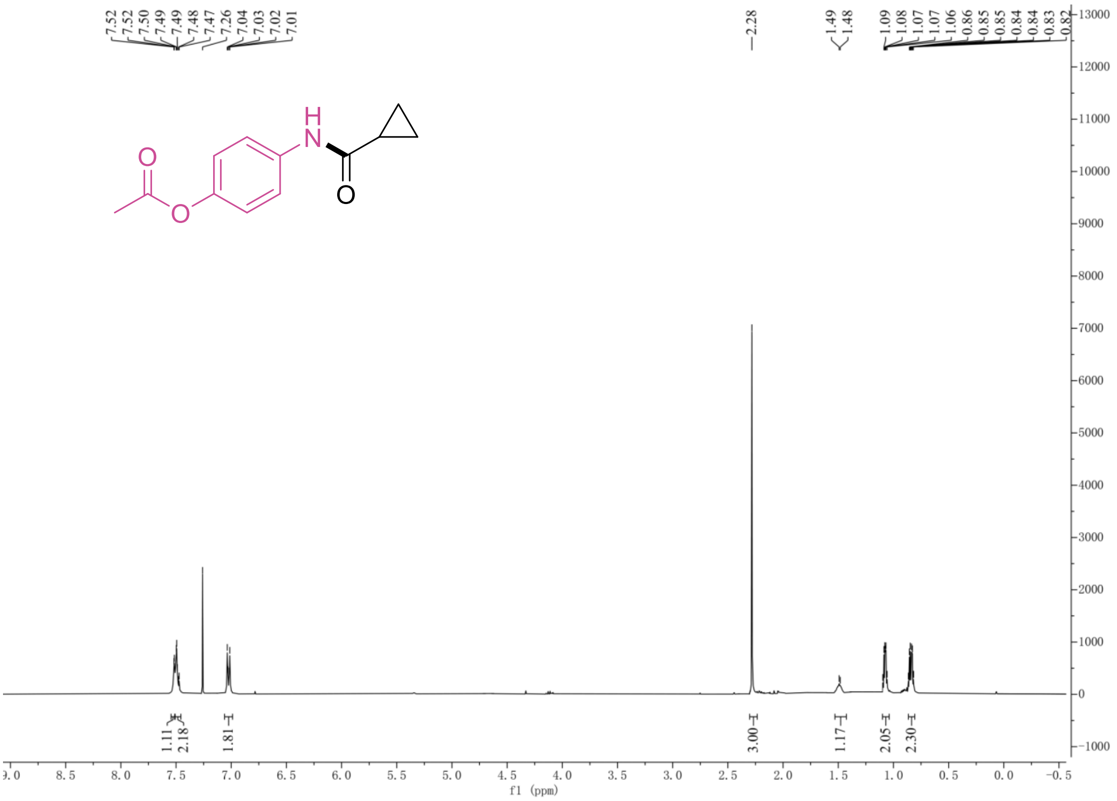

Supplementary Figure 57b | ^{13}C NMR (126 MHz, DMSO-d_6) of 4-(cyclopropanecarboxamido)phenyl acetate **(3nn).**

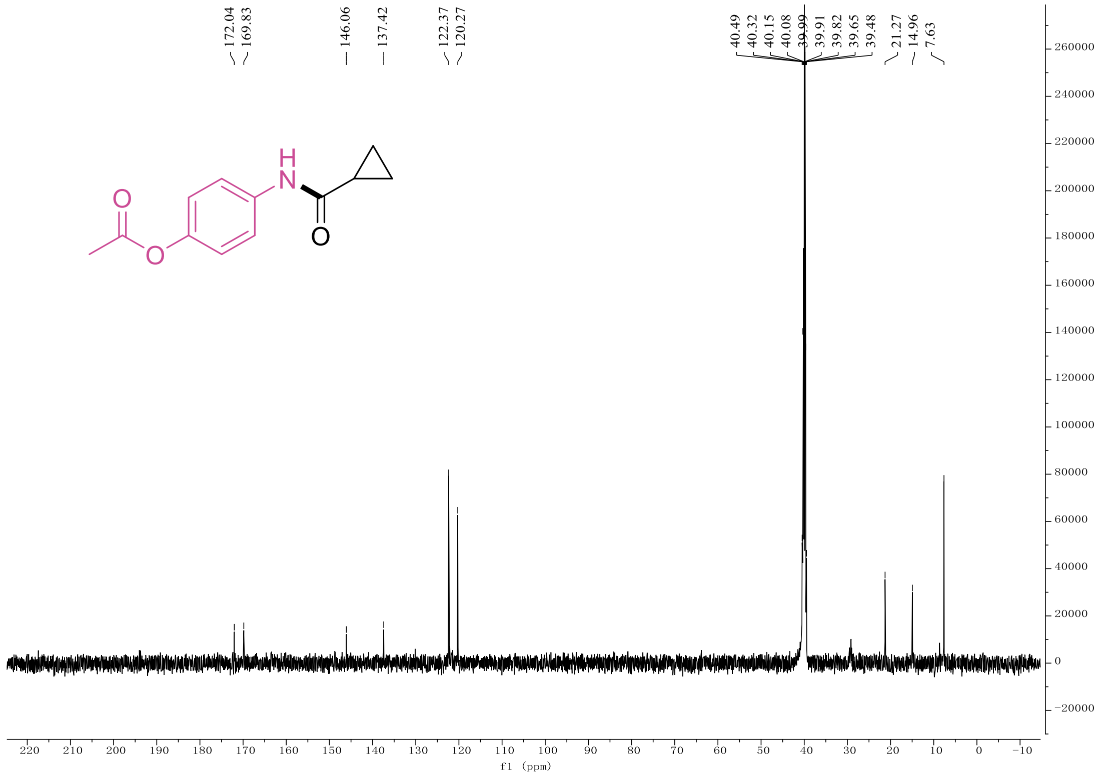

Supplementary Figure 58a | ^{1}H NMR (400 MHz, DMSO-d_6) of *N*-(4-isobutyramido-2-(trifluoromethyl)phenyl)cyclopropanecarboxamide **(3oo).**

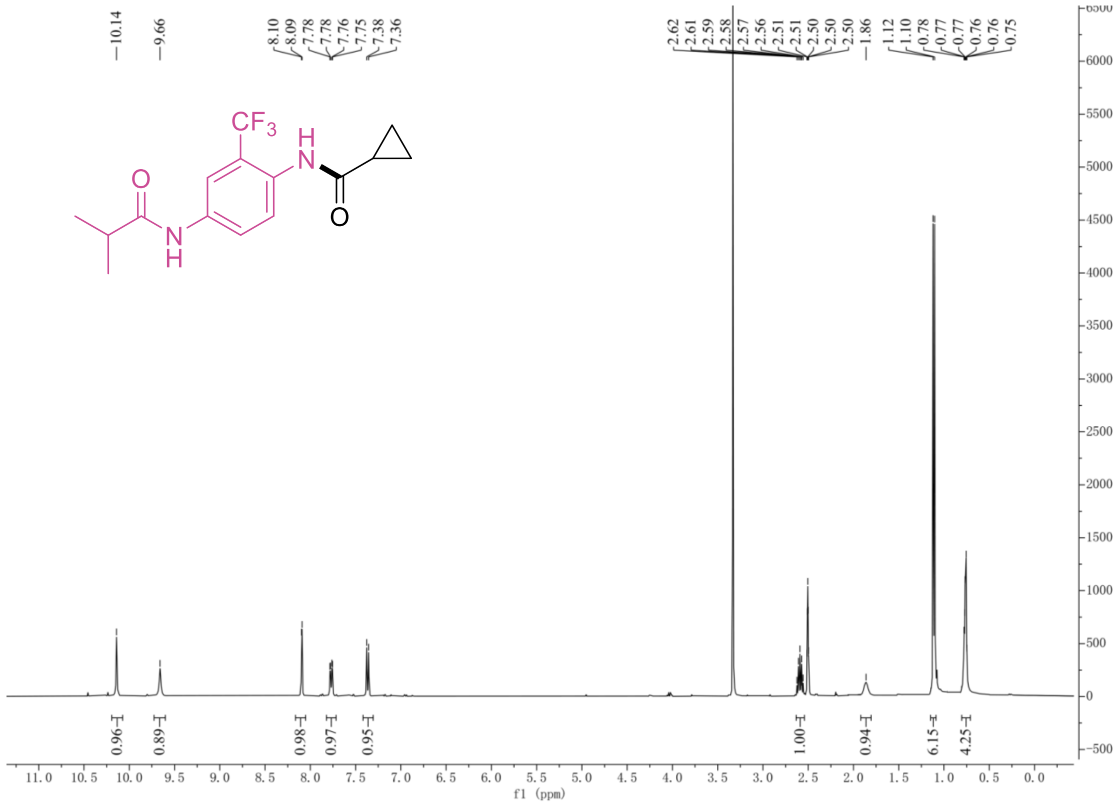

Supplementary Figure 58b | ^{13}C NMR (126 MHz, DMSO-d_6) of *N*-(4-isobutyramido-2-(trifluoromethyl)phenyl)cyclopropanecarboxamide **(3oo).**

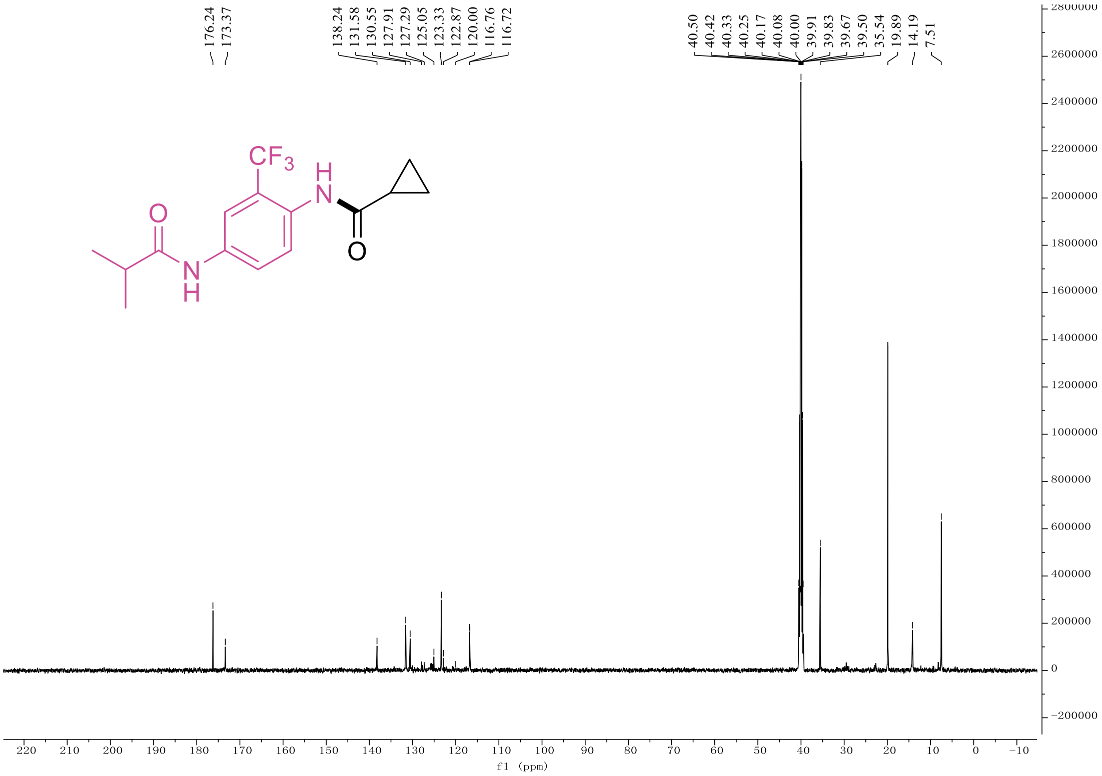

Supplementary Figure 58c | ^{19}F NMR (377 MHz, DMSO-d_6) of *N*-(4-isobutyramido-2-(trifluoromethyl)phenyl)cyclopropanecarboxamide **(3oo).**

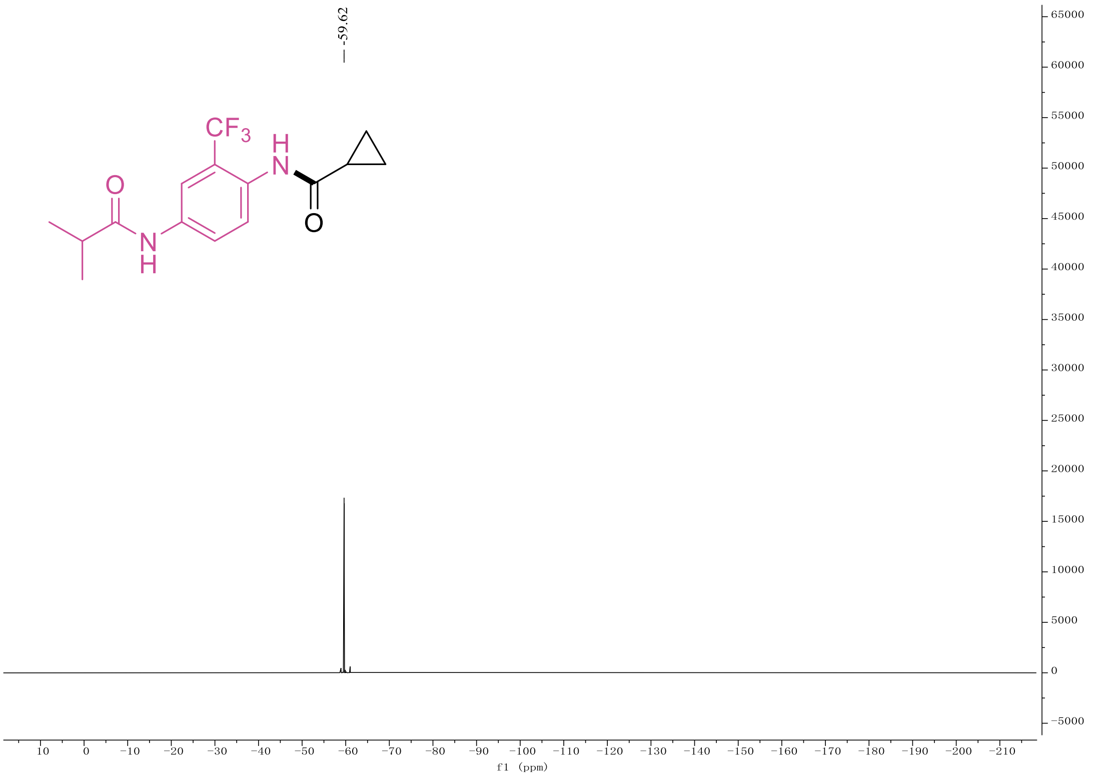

Supplementary Figure 59a | ^{1}H NMR (400 MHz, DMSO-d_6) of *N*-(4-(4,4-dimethyl-2,5-dioxoimidazolidin-1-yl)-2-(trifluoromethyl)phenyl)cyclopropanecarboxamide **(3pp).**

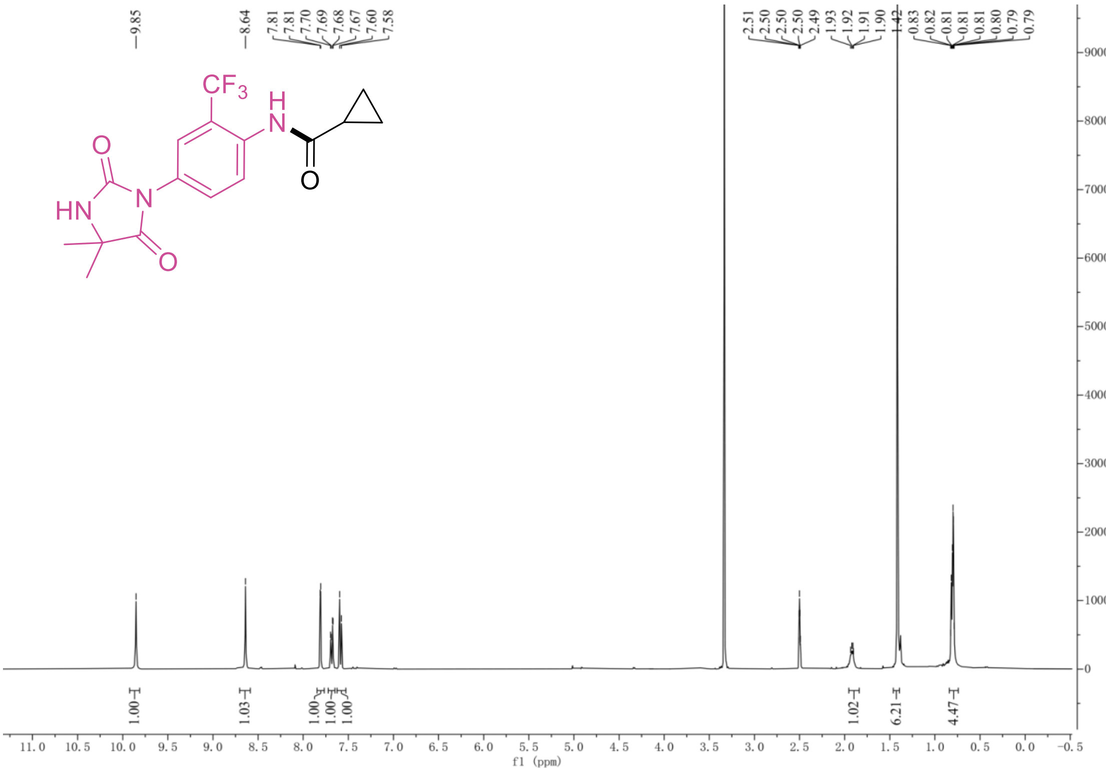

Supplementary Figure 59b | ^{13}C NMR (126 MHz, DMSO-d_6) of *N*-(4-(4,4-dimethyl-2,5-dioxoimidazolidin-1-yl)-2-(trifluoromethyl)phenyl)cyclopropanecarboxamide **(3pp).**

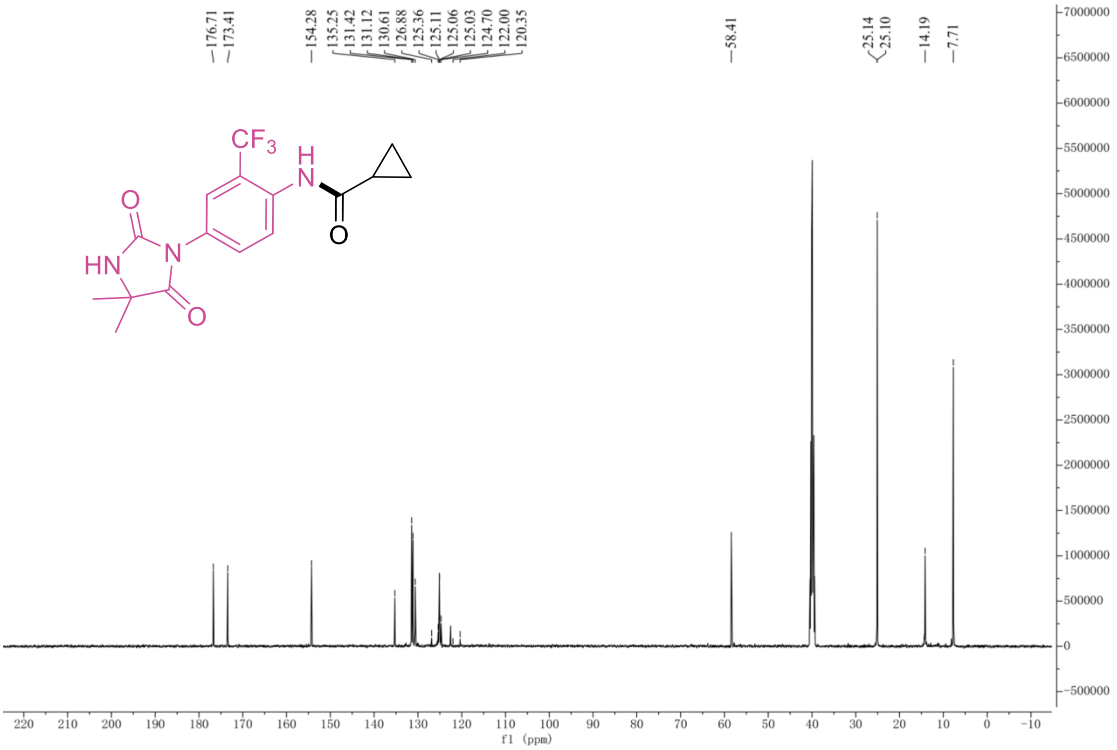

Supplementary Figure 59c | ^{19}F NMR (377 MHz, DMSO-d_6) of *N*-(4-(4,4-dimethyl-2,5-dioxoimidazolidin-1-yl)-2-(trifluoromethyl)phenyl)cyclopropanecarboxamide **(3pp).**

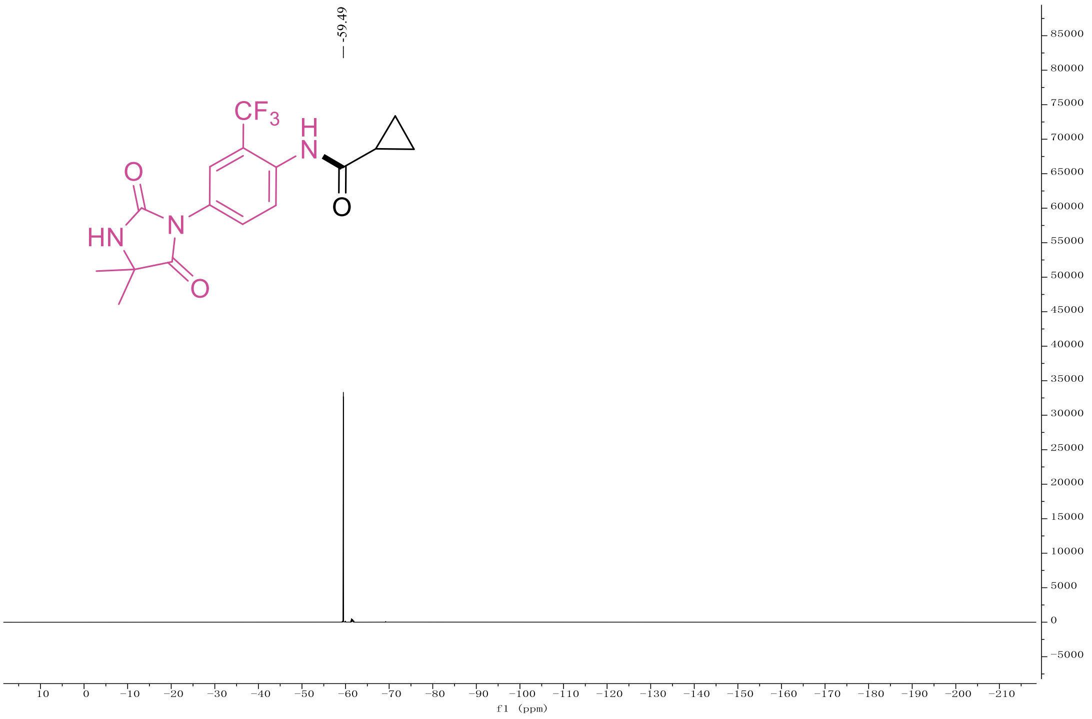

Supplementary Figure 60a | ^{1}H NMR (400 MHz, DMSO-d_6) of *N*-(5-cyano-2-hydroxy-3-iodophenyl)cyclopropanecarboxamide **(3qq).**

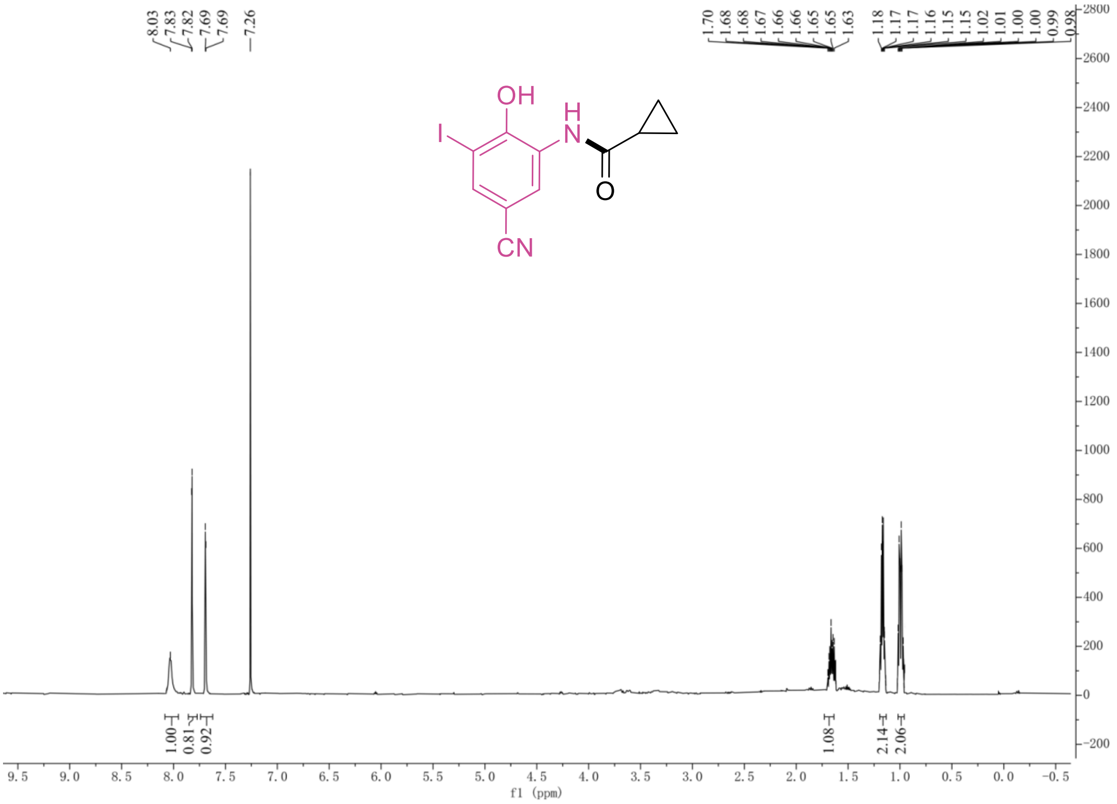

Supplementary Figure 60b | ^{13}C NMR (126 MHz, DMSO-d_6) of *N*-(5-cyano-2-hydroxy-3-iodophenyl)cyclopropanecarboxamide **(3qq).**

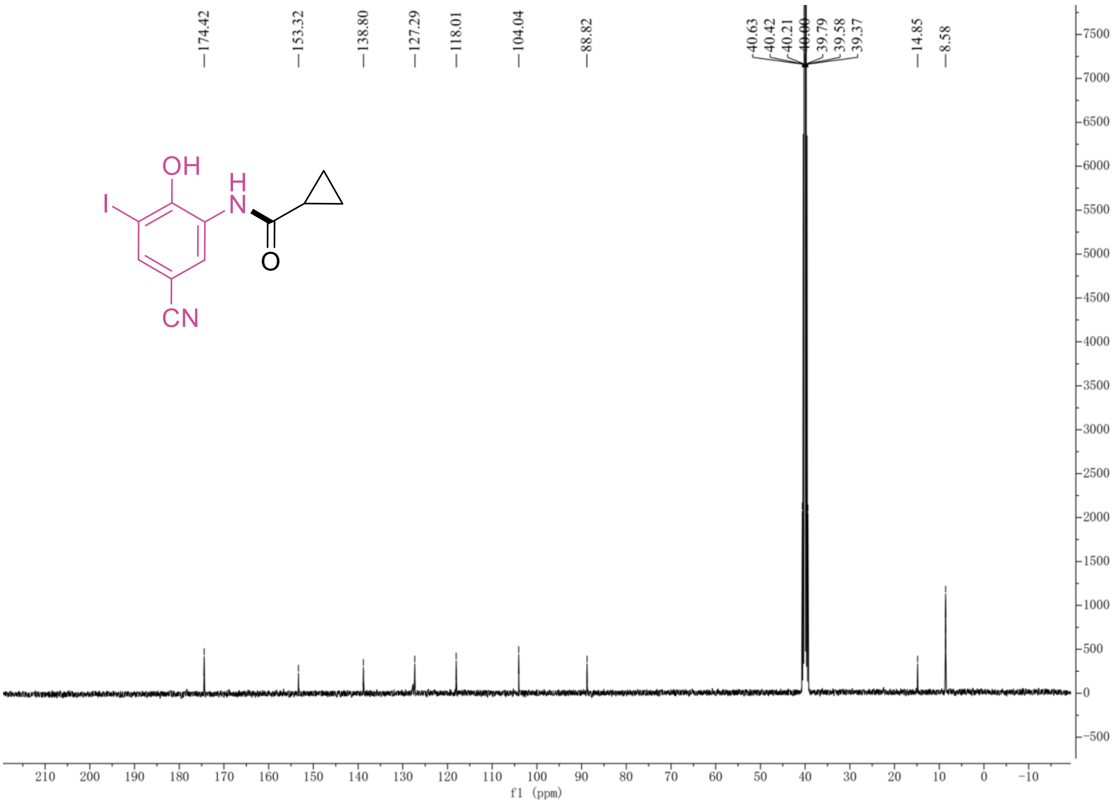

Supplementary Figure 61a | ^{1}H NMR (400 MHz, DMSO-d_6) of *N*-(4-(methylsulfonamido)-3-phenoxyphenyl)cyclopropanecarboxamide **(3rr).**

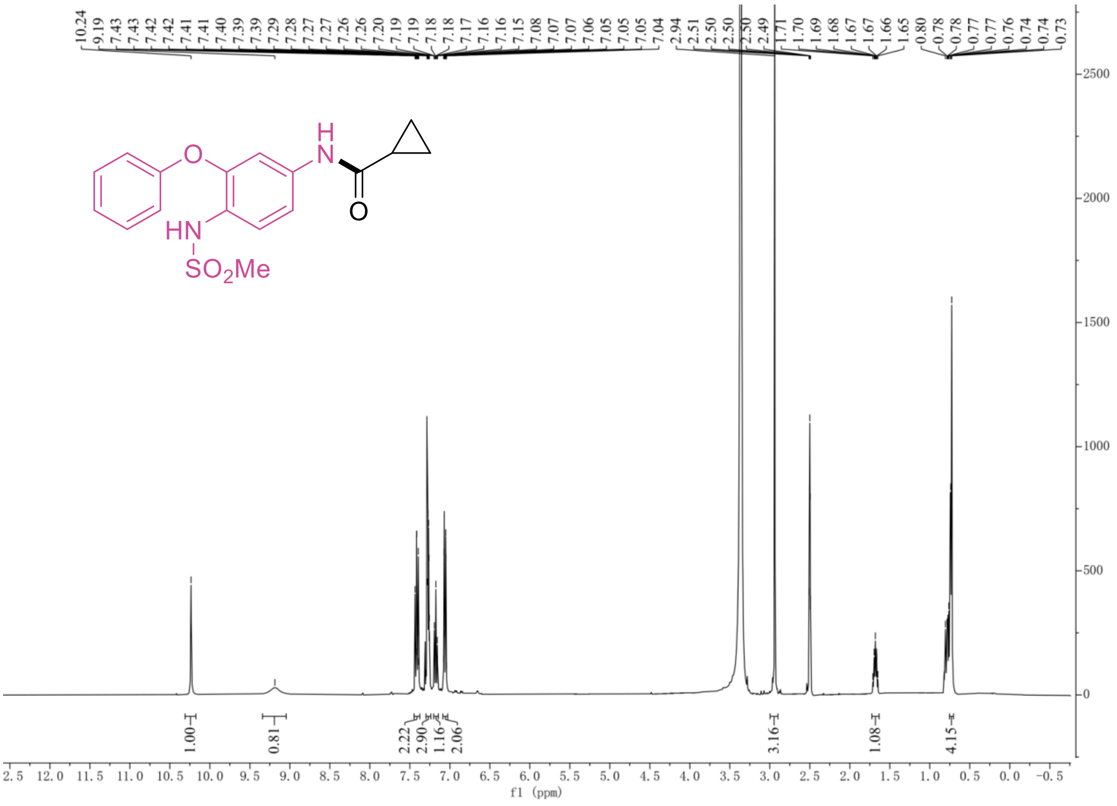

Supplementary Figure 61b | ^{13}C NMR (126 MHz, DMSO-d_6) of *N*-(4-(methylsulfonamido)-3-phenoxyphenyl)cyclopropanecarboxamide **(3rr).**

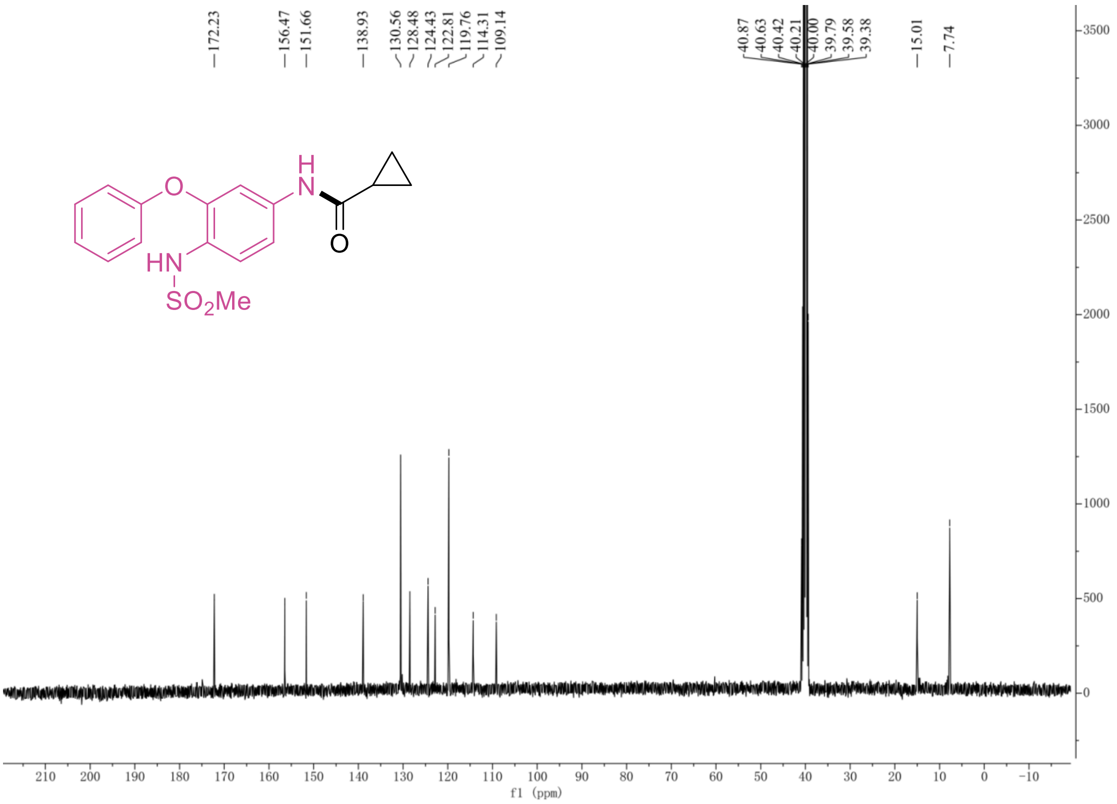

Supplementary Figure 62a | ^{1}H NMR (400 MHz, DMSO-d_6) of *N*-(4-((1*R*,2*R*)-2-(2,2-dichloroacetamido)-1,3-dihydroxypropyl)phenyl)cyclopropanecarboxamide **(3ss).**

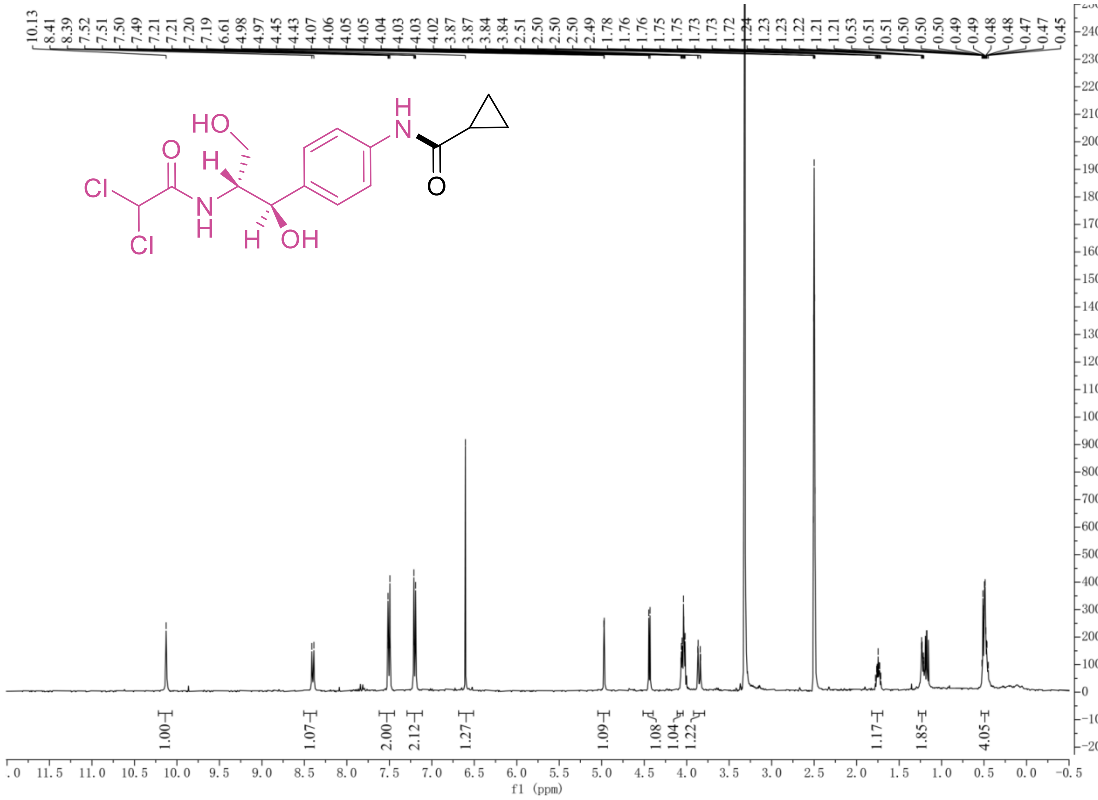

Supplementary Figure 62b | ^{13}C NMR (126 MHz, DMSO-d_6) of *N*-(4-((1*R*,2*R*)-2-(2,2-dichloroacetamido)-1,3-dihydroxypropyl)phenyl)cyclopropanecarboxamide **(3ss).**

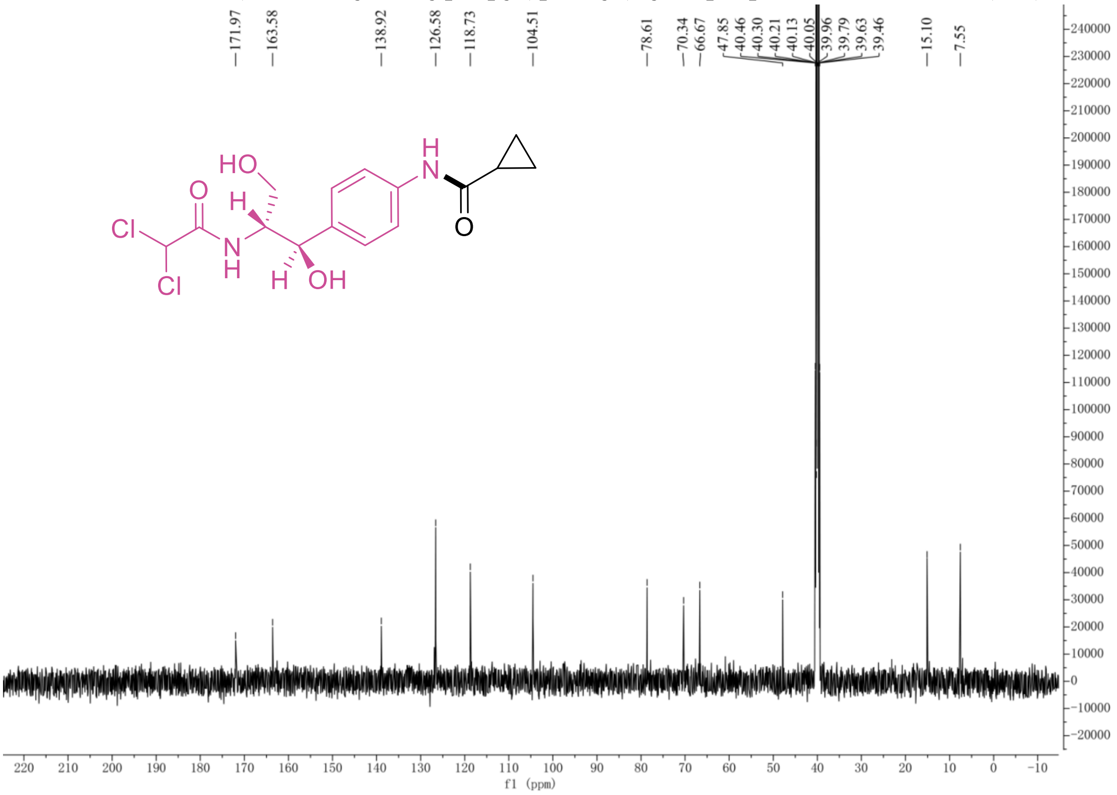

Supplementary Figure 63a | ^{1}H NMR (400 MHz, Chloroform-*d*) of (1*R*,2*R*,4*S*)-3,3,4-trimethylbicyclo[2.2.1]heptan-2-yl 4-(phenylcarbamoyl)benzoate **(3tt).**

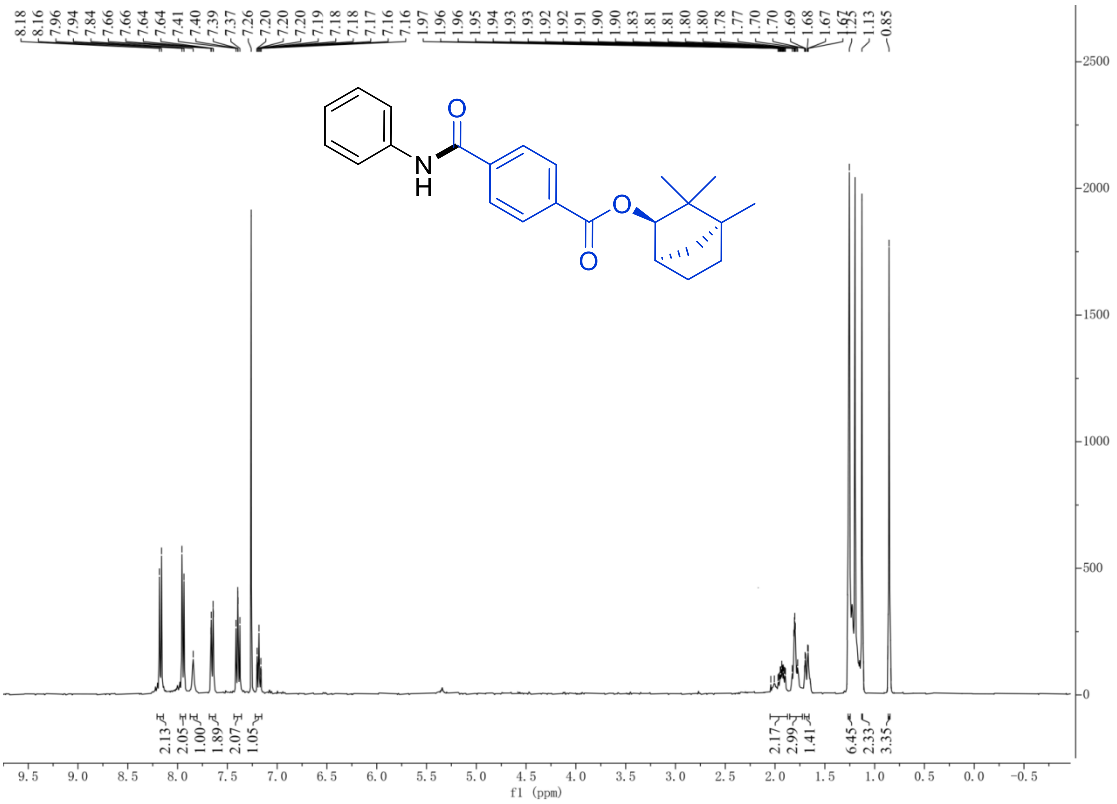

Supplementary Figure 63b | ^{13}C NMR (126 MHz, DMSO-d_6) of (1*R*,2*R*,4*S*)-3,3,4-trimethylbicyclo[2.2.1]heptan-2-yl 4-(phenylcarbamoyl)benzoate **(3tt).**

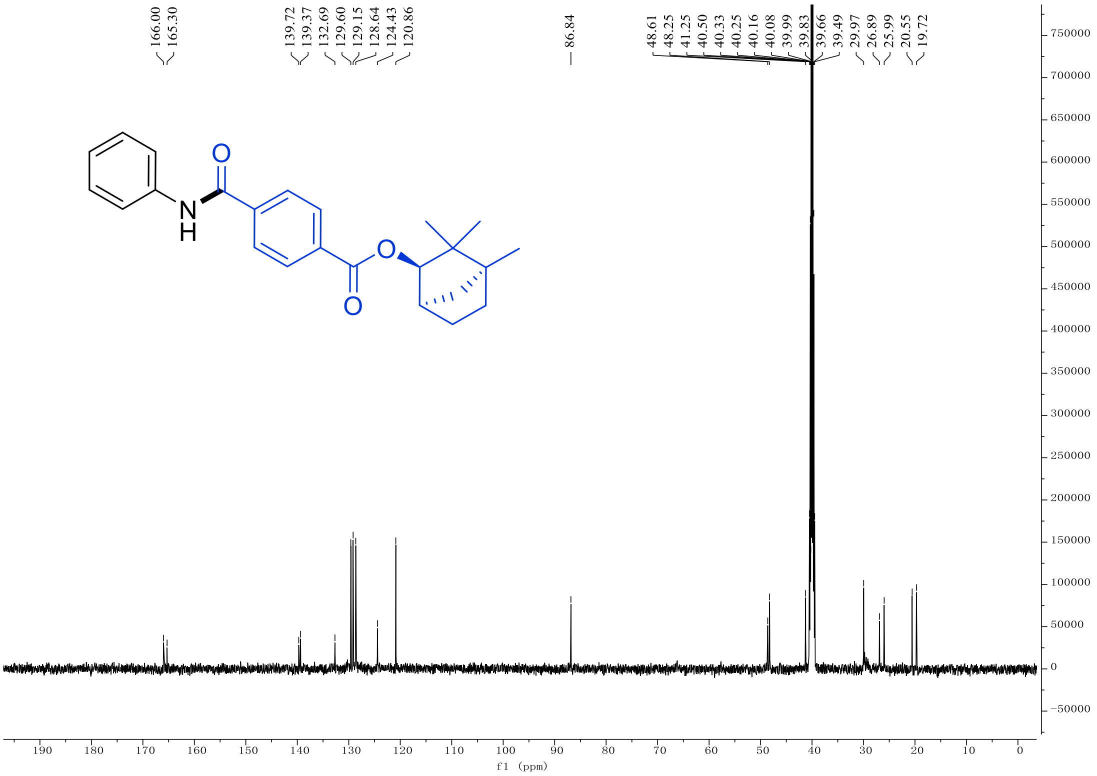

Supplementary Figure 64a | ^{1}H NMR (400 MHz, DMSO-d_6) of (1*R*,2*R*,4*S*)-1,3,3-trimethylbicyclo[2.2.1]heptan-2-yl 4-((4-(4,4-dimethyl-2,5-dioxoimidazolidin-1-yl)-2-(trifluoromethyl)phenyl)carbamoyl)benzoate **(3uu).**

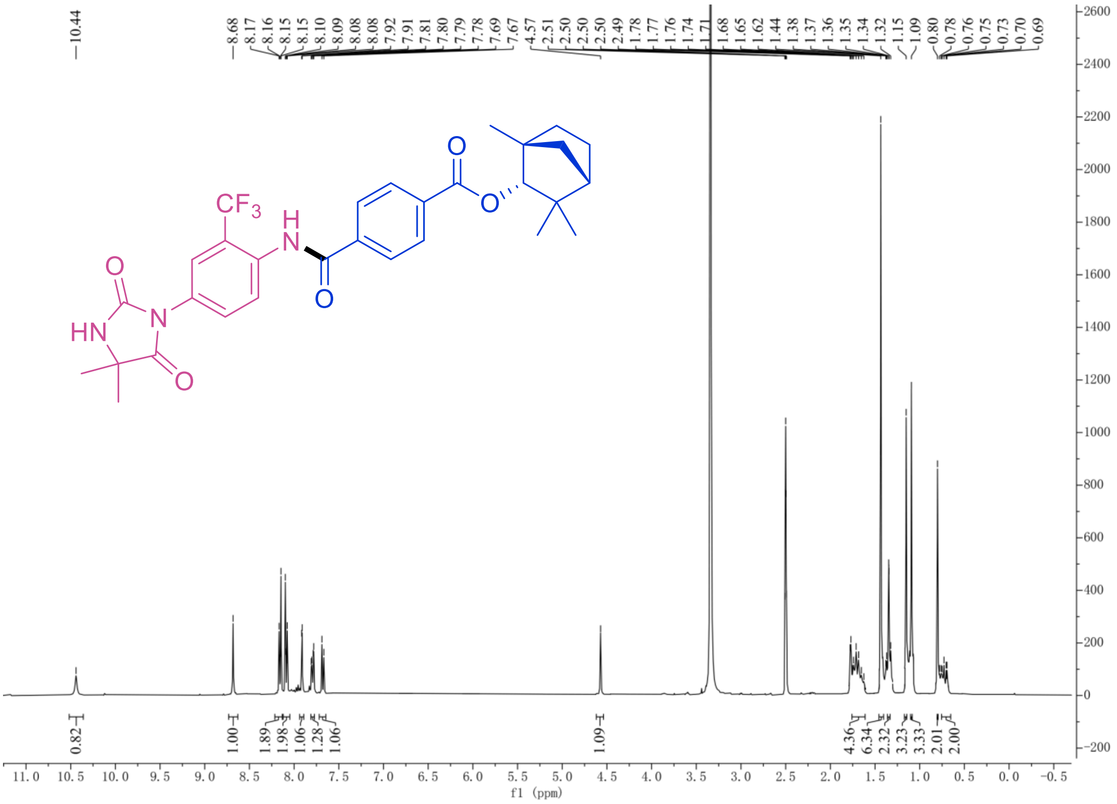

Supplementary Figure 64b | ^{13}C NMR (126 MHz, DMSO-d_6) of (1*R*,2*R*,4*S*)-1,3,3-trimethylbicyclo[2.2.1]heptan-2-yl 4-((4-(4,4-dimethyl-2,5-dioxoimidazolidin-1-yl)-2-(trifluoromethyl)phenyl)carbamoyl)benzoate **(3uu).**

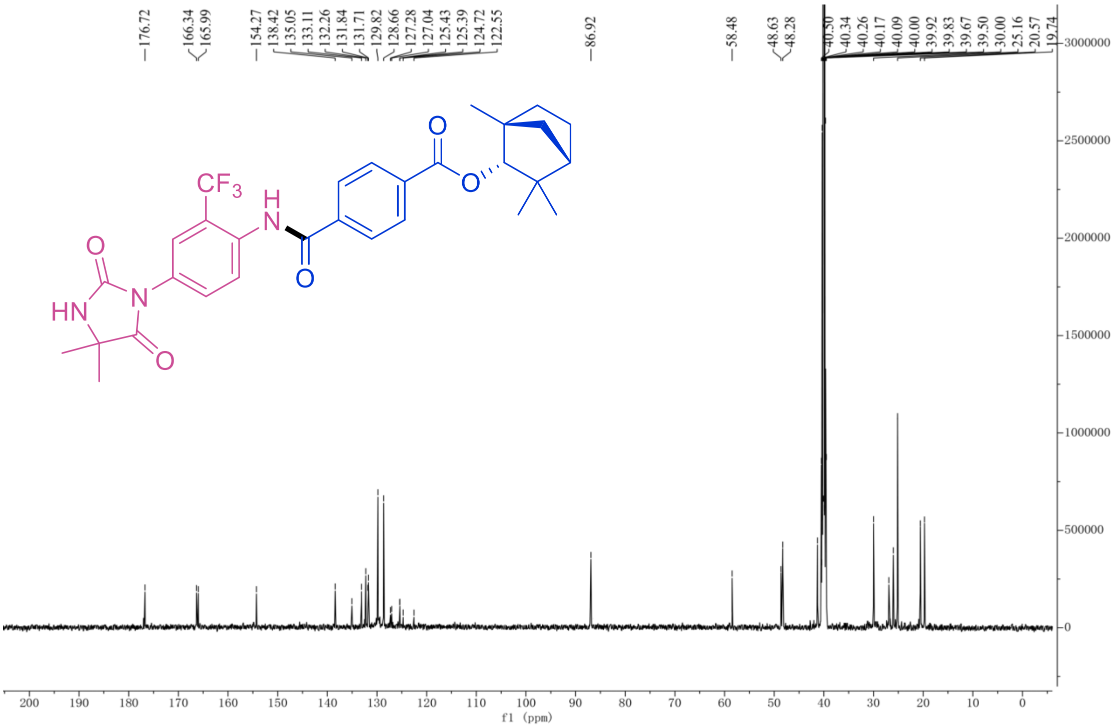

Supplementary Figure 64c | ^{19}F NMR (377 MHz, DMSO-d_6) of (1*R*,2*R*,4*S*)-1,3,3-trimethylbicyclo[2.2.1]heptan-2-yl 4-((4-(4,4-dimethyl-2,5-dioxoimidazolidin-1-yl)-2-(trifluoromethyl)phenyl)carbamoyl)benzoate **(3uu).**

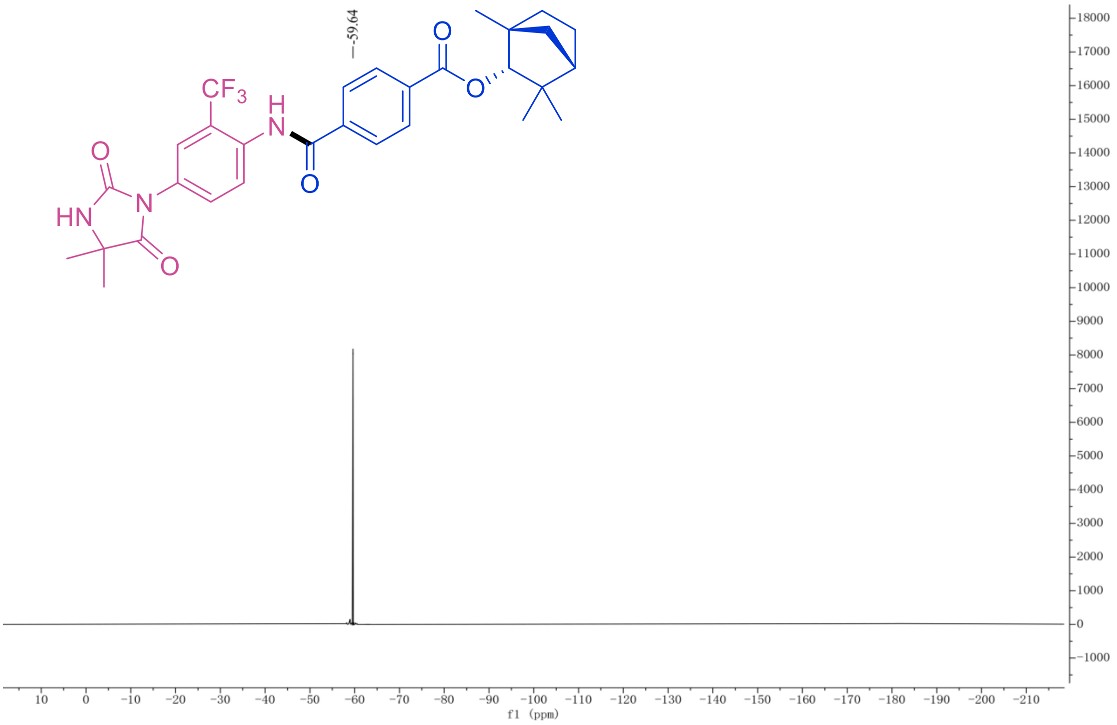

Supplementary Figure 65a | **^{1}H NMR** (400 MHz, DMSO-d_6) of (1*R*,2*S*,5*R*)-2-isopropyl-5-methylcyclohexyl 4-((4-(4,4-dimethyl-2,5-dioxoimidazolidin-1-yl)-2-(trifluoromethyl)phenyl)carbamoyl)benzoate **(3vv).**

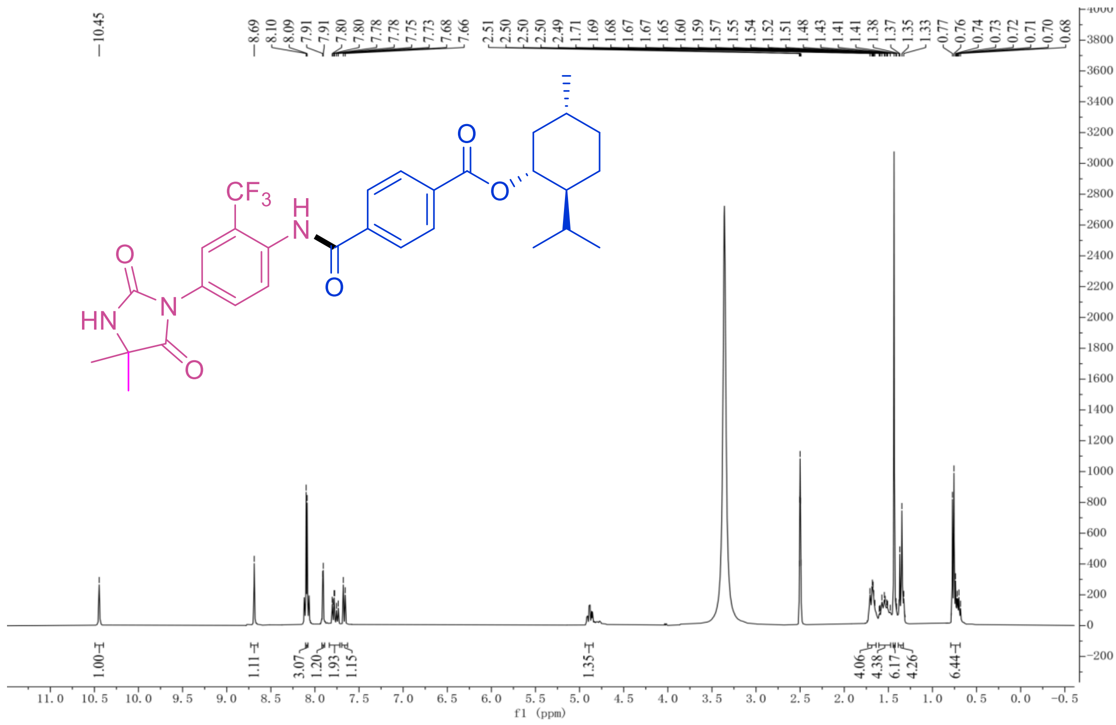

Supplementary Figure 65b | **^{13}C NMR** (126 MHz, DMSO-d_6) of (1*R*,2*S*,5*R*)-2-isopropyl-5-methylcyclohexyl 4-((4-(4,4-dimethyl-2,5-dioxoimidazolidin-1-yl)-2-(trifluoromethyl)phenyl)carbamoyl)benzoate **(3vv).**

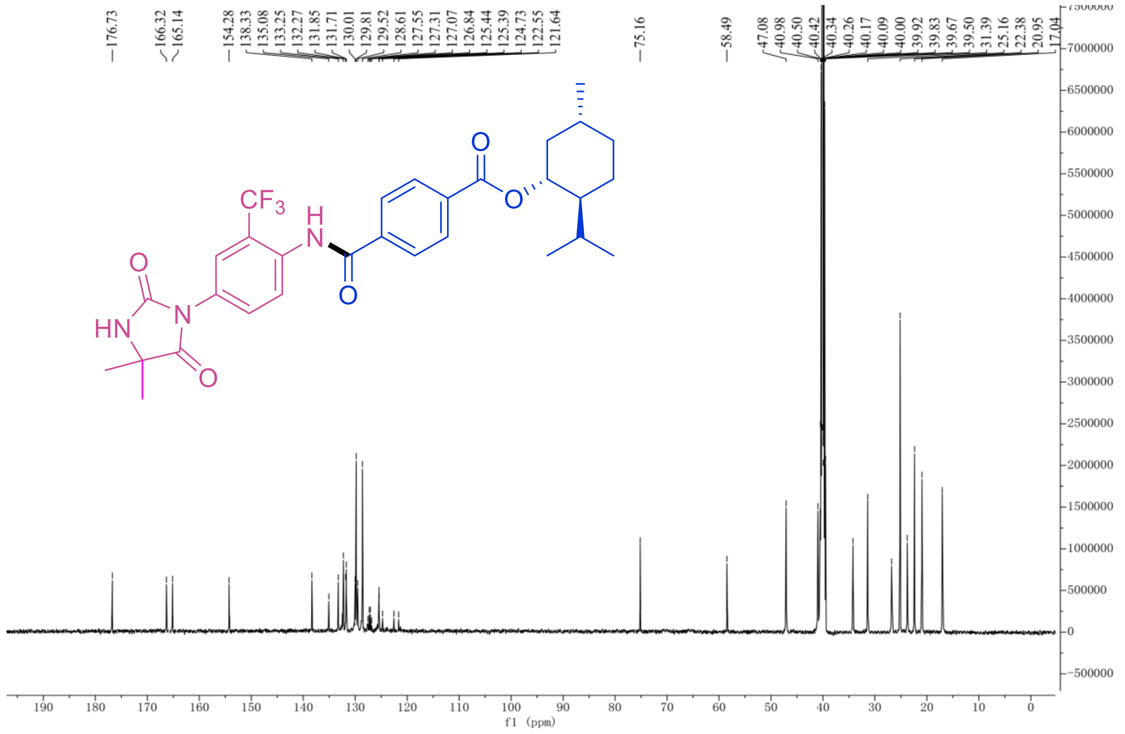

Supplementary Figure 65c | **^{19}F NMR** (377 MHz, DMSO-d_6) of (1*R*,2*S*,5*R*)-2-isopropyl-5-methylcyclohexyl 4-((4-(4,4-dimethyl-2,5-dioxoimidazolidin-1-yl)-2-(trifluoromethyl)phenyl)carbamoyl)benzoate **(3vv).**

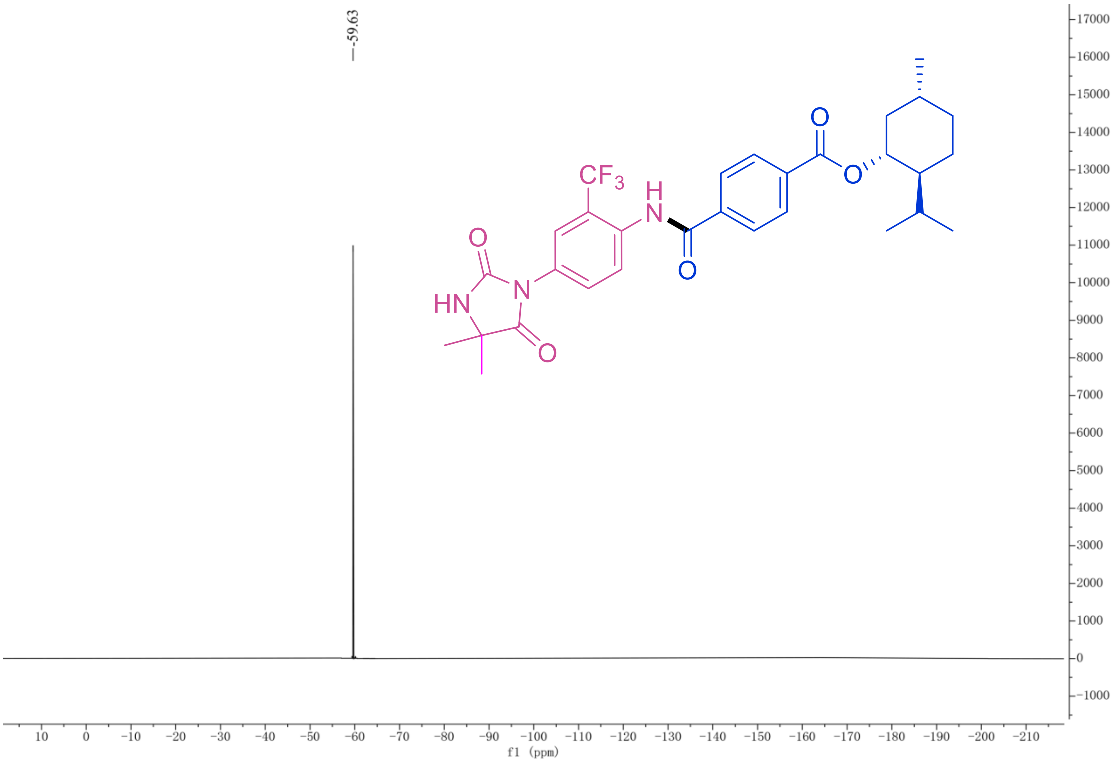

Supplementary Figure 66a | **^{1}H NMR** (400 MHz, DMSO-d_6) of *N*-(4-(4,4-dimethyl-2,5-dioxoimidazolidin-1-yl)-2-(trifluoromethyl)phenyl)-7-hydroxy-3,7-dimethyloctanamide **(3ww).**

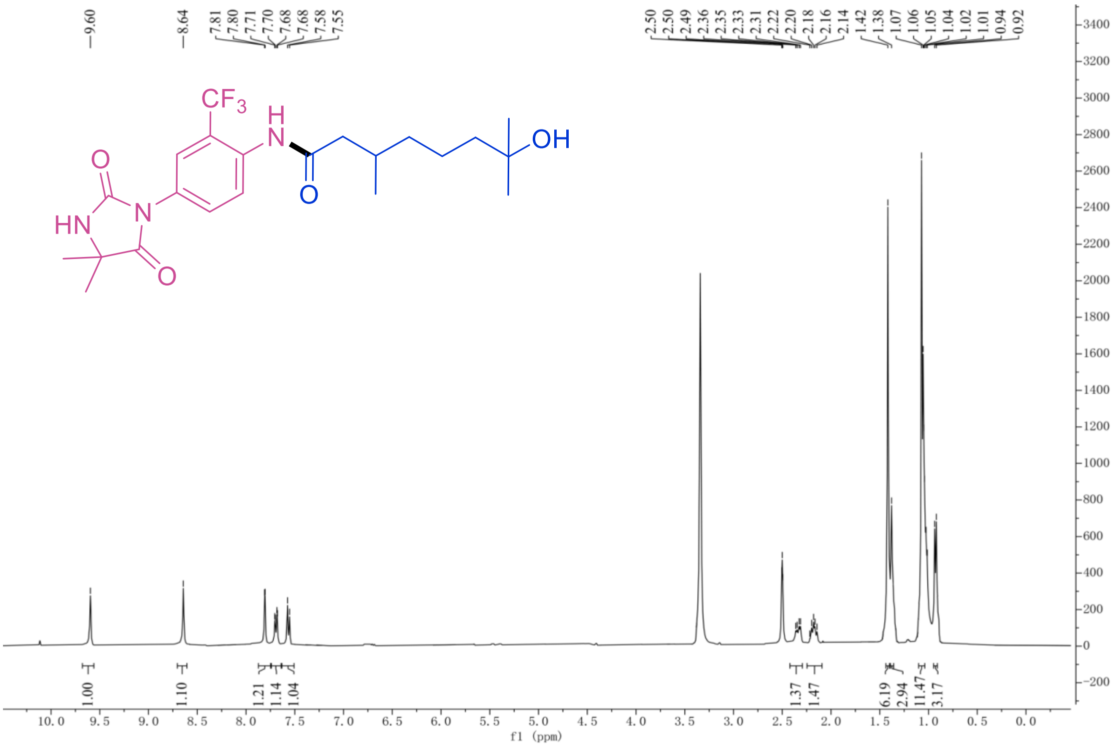

Supplementary Figure 66b | **^{13}C NMR** (101 MHz, DMSO-d_6) of *N*-(4-(4,4-dimethyl-2,5-dioxoimidazolidin-1-yl)-2-(trifluoromethyl)phenyl)-7-hydroxy-3,7-dimethyloctanamide **(3ww).**

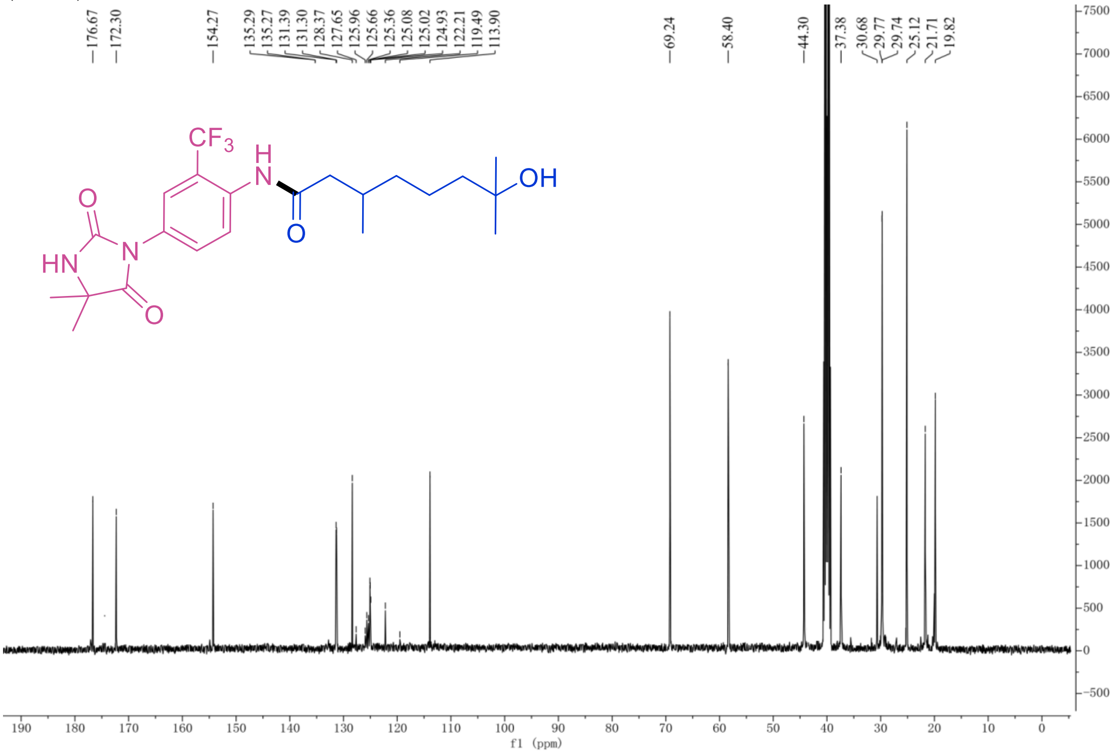

Supplementary Figure 66c | **^{19}F NMR** (377 MHz, DMSO-d_6) of *N*-(4-(4,4-dimethyl-2,5-dioxoimidazolidin-1-yl)-2-(trifluoromethyl)phenyl)-7-hydroxy-3,7-dimethyloctanamide **(3ww).**

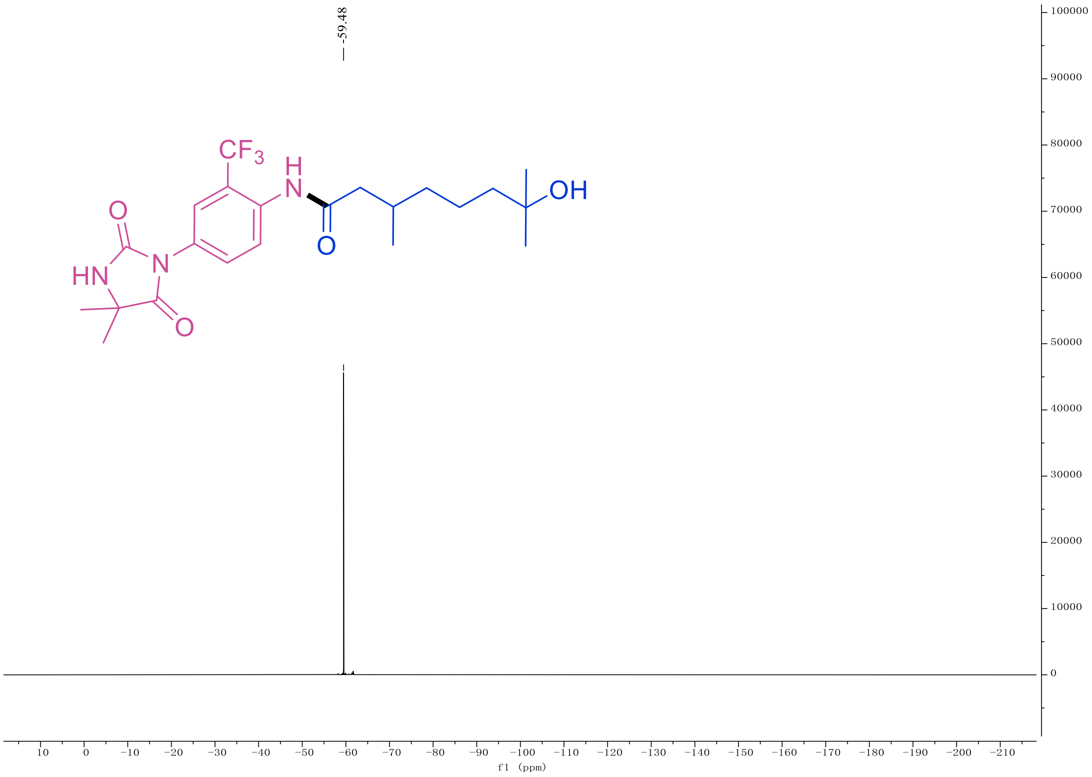

Supplementary Figure 67a | ^{1}H NMR (400 MHz, Chloroform-*d*) of **5-methyl-*N*-(4-(trifluoromethyl)phenyl)isoxazole-4-carboxamide (3xx).**

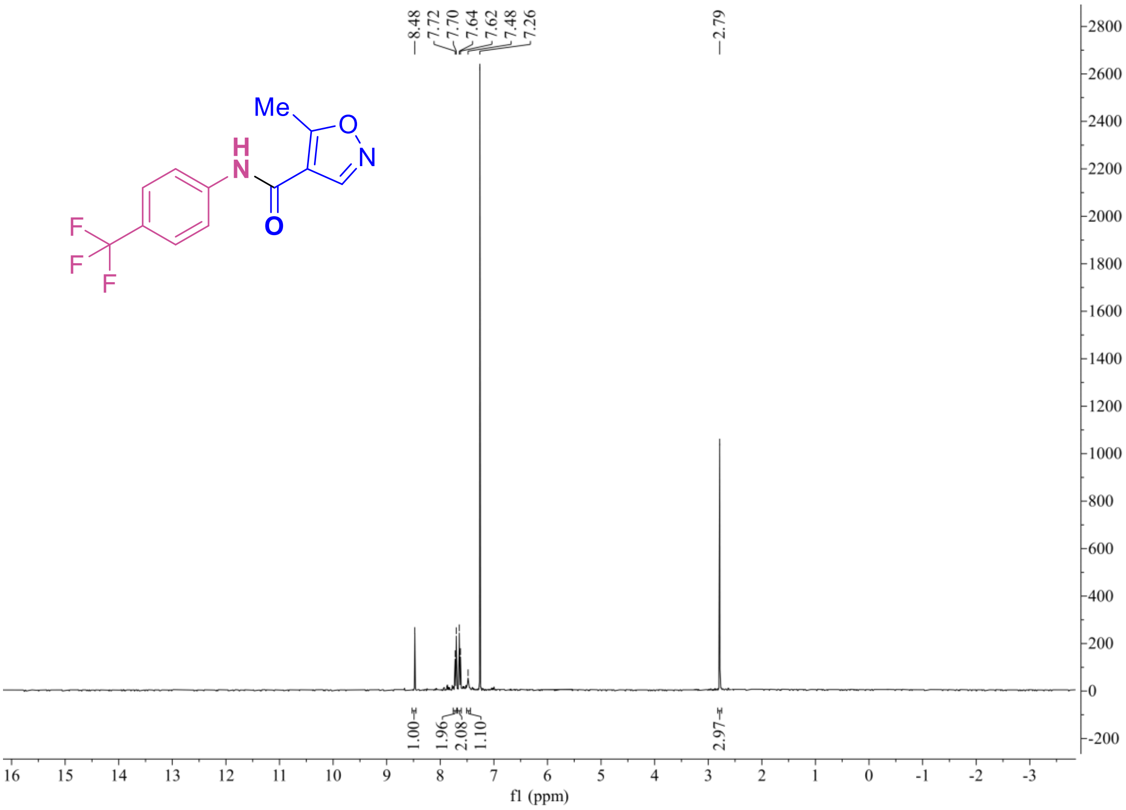

Supplementary Figure 67b | ^{13}C NMR (126 MHz, DMSO-d_6) of **5-methyl-*N*-(4-(trifluoromethyl)phenyl)isoxazole-4-carboxamide (3xx).**

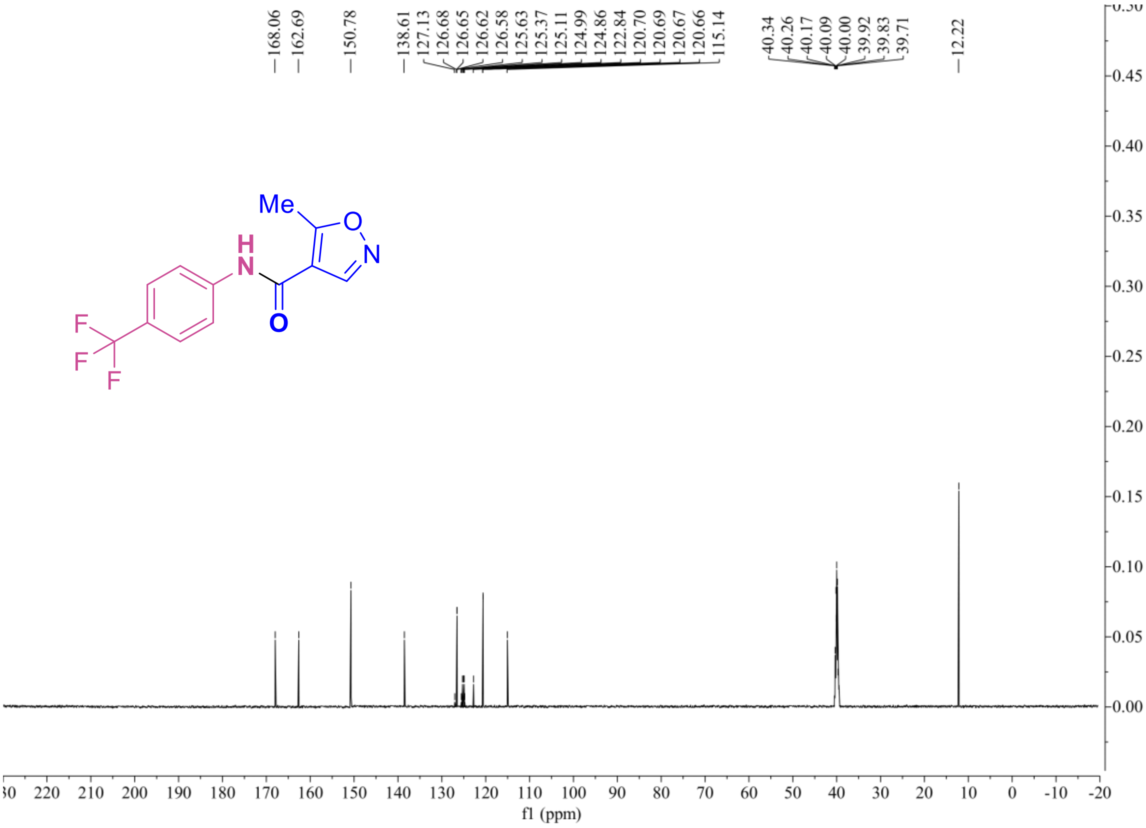

Supplementary Figure 68a | ^{1}H NMR (400 MHz, Chloroform-*d*) of **2-(diethylamino)-*N*-(2,6-dimethylphenyl)acetamide (3yy).**

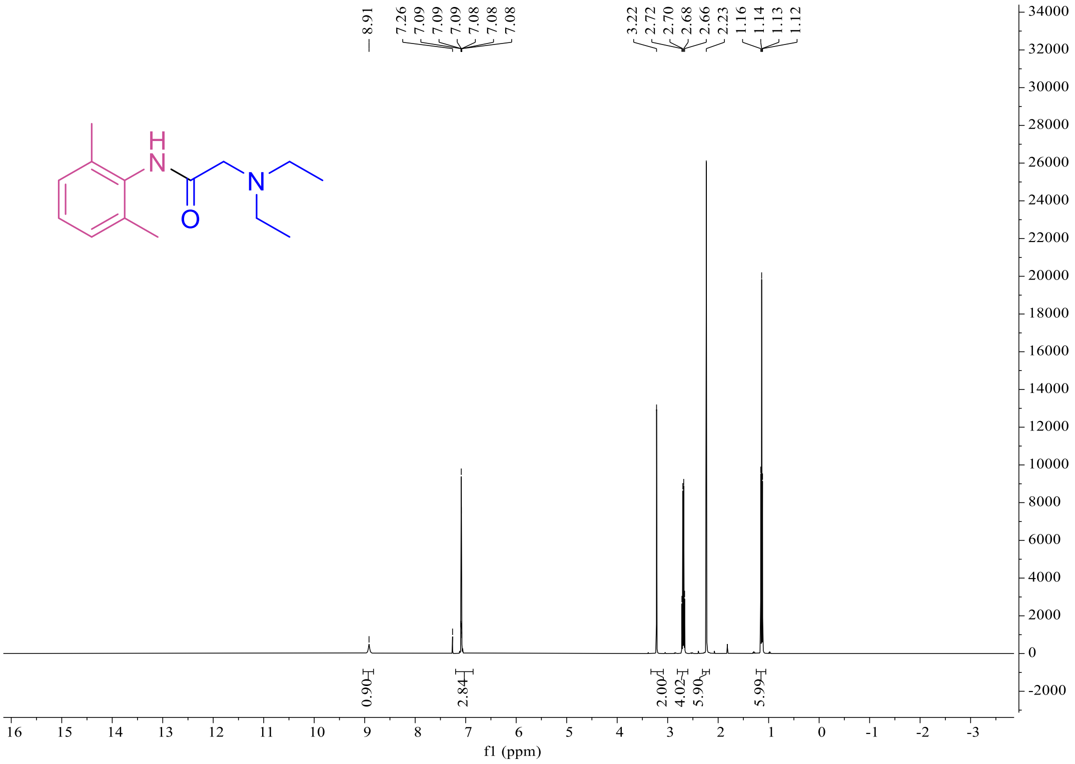

Supplementary Figure 68b | ^{13}C NMR (126 MHz, DMSO-*d*$_6$) of **2-(diethylamino)-*N*-(2,6-dimethylphenyl)acetamide (3yy).**

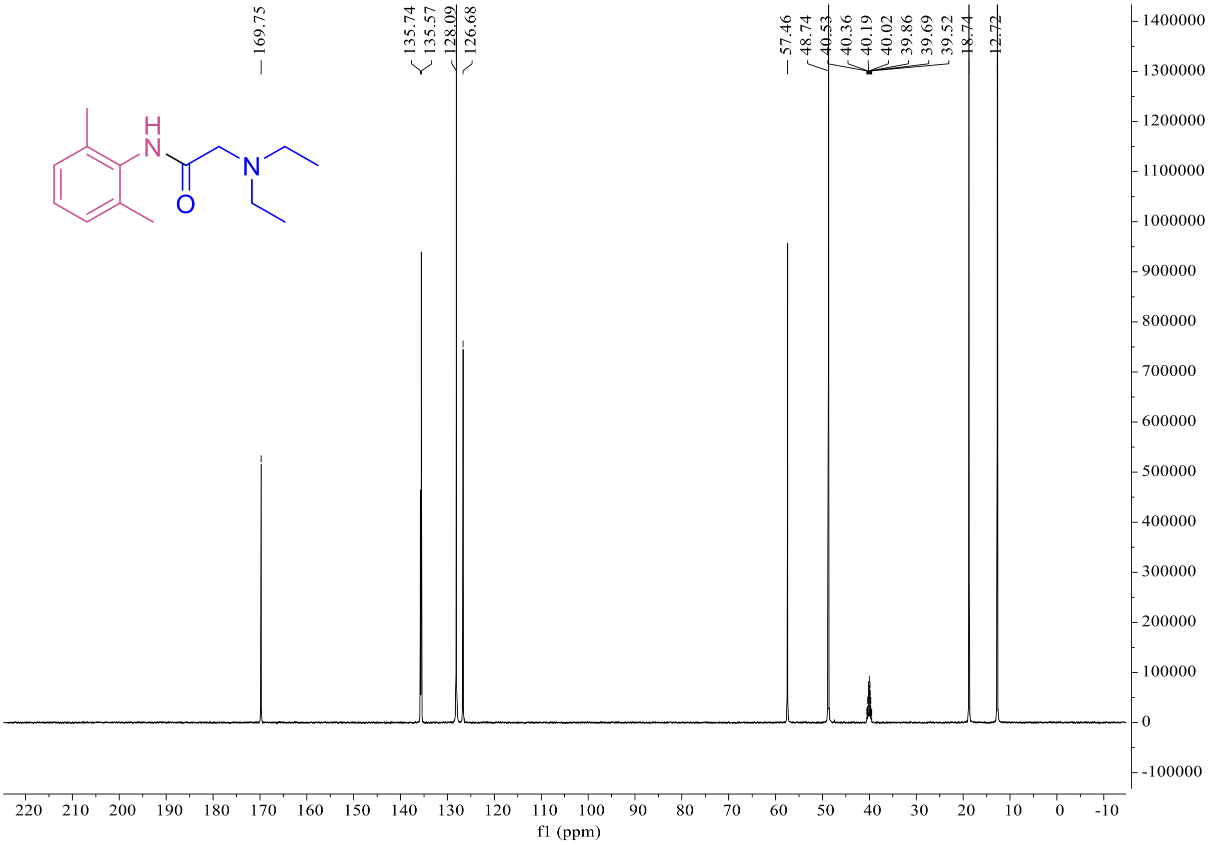

Supplementary Figure 69a | ^{1}H NMR (400 MHz, Chloroform-*d*) of ***N*-hydroxy-*N*-phenylcyclopropanecarboxamide (6).**

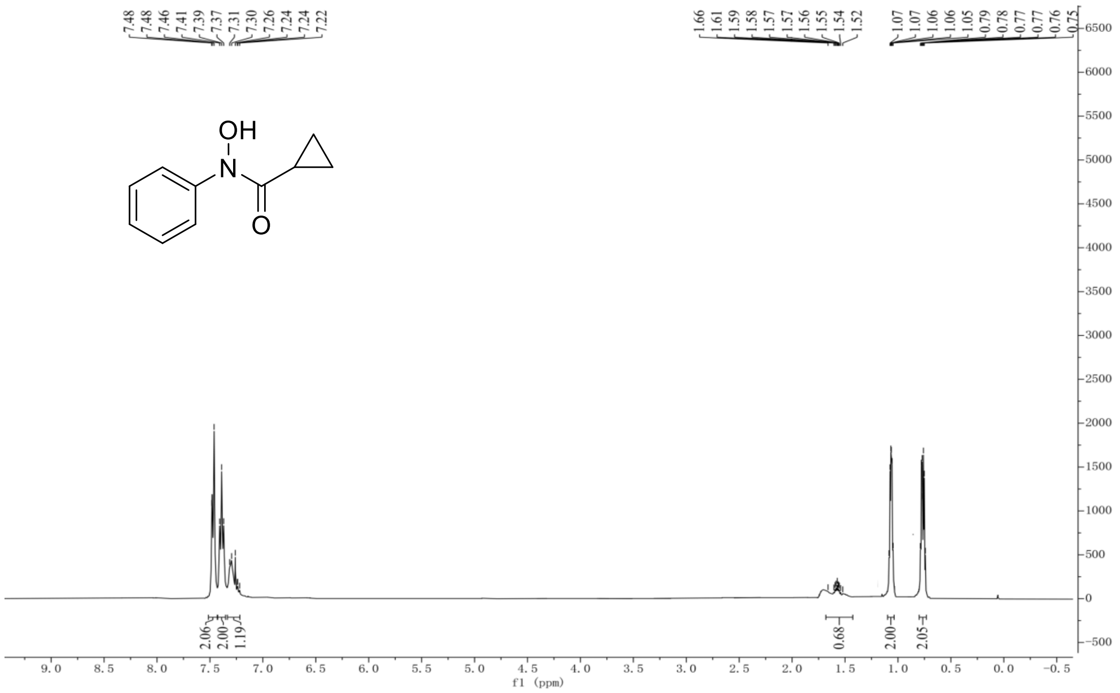

Supplementary Figure 69b | ^{13}C NMR (126 MHz, Chloroform-*d*) of ***N*-hydroxy-*N*-phenylcyclopropanecarboxamide (6).**

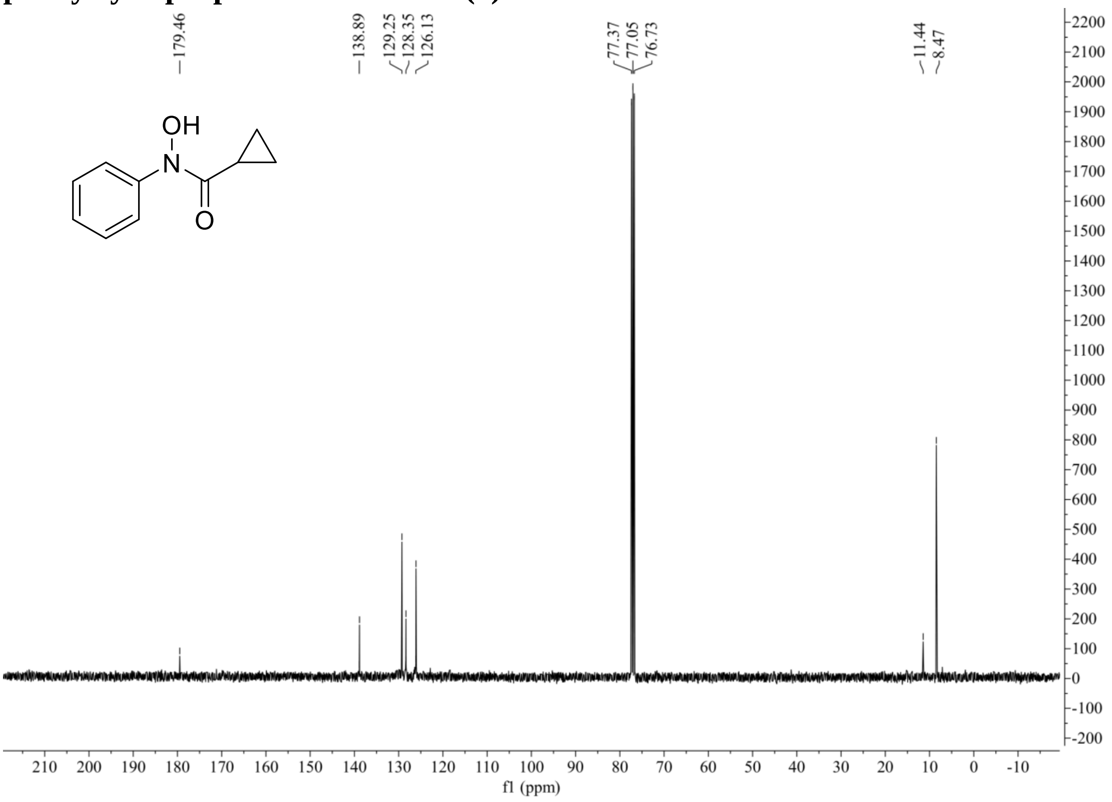

Supplementary Figure 70a | ^{1}H NMR (400 MHz, Chloroform-*d*) of ***N*-(cyclopropyl(hydroxy)methyl)-*N*-phenylcyclopropanecarboxamide (9)**.

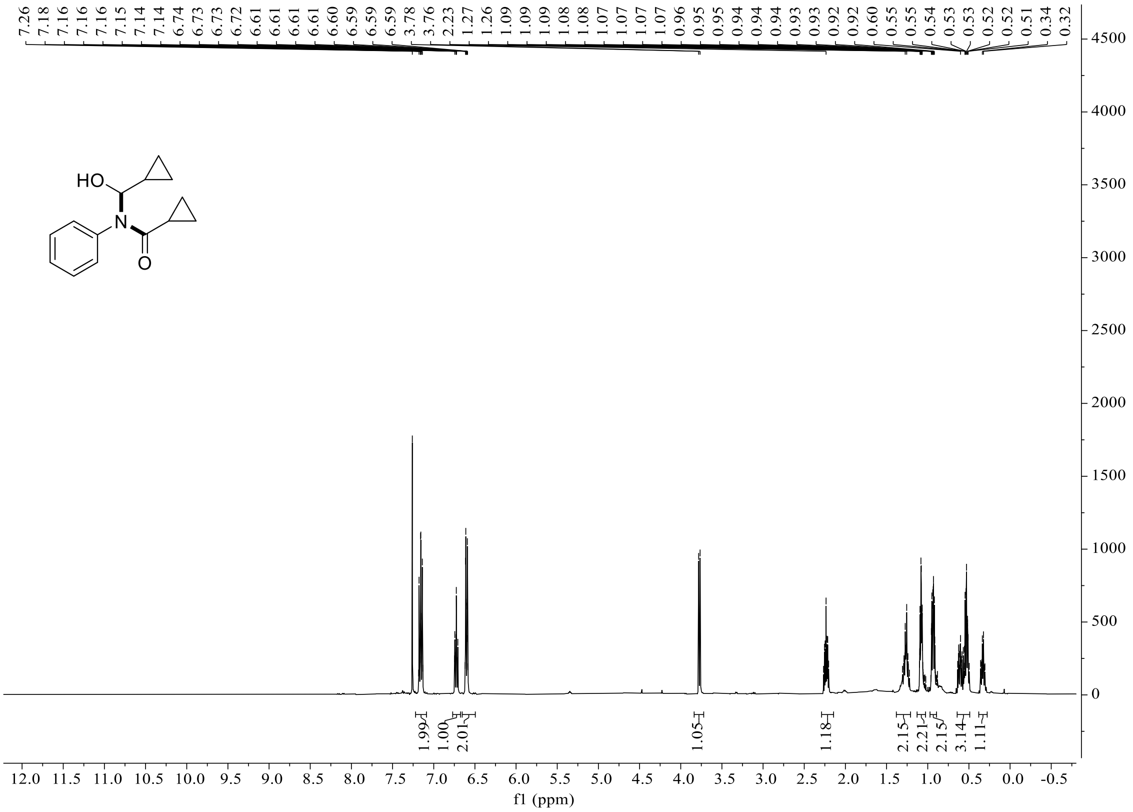

Supplementary Figure 70b | ^{13}C NMR (126 MHz, Chloroform-*d*) of ***N*-(cyclopropyl(hydroxy)methyl)-*N*-phenylcyclopropanecarboxamide (9)**.

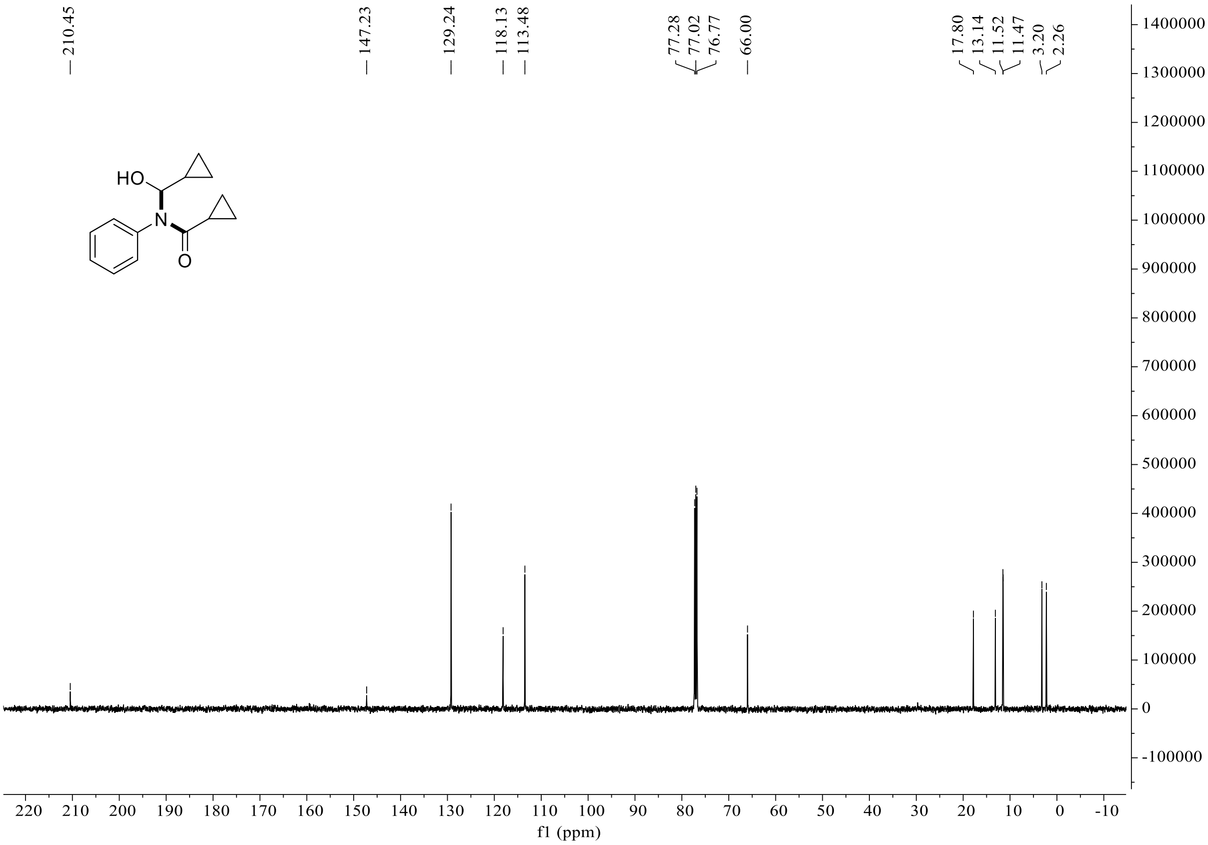

VIII. Supplementary References

1. J. Yan, H. W. Cheo, W. K. Teo X. Shi, H. Wu, S. B. Idres, L. W. Deng and J. Wu, *J. Am. Chem. Soc.,* 2020, **142**, 11357-11362.
2. *(a)* F. Weigend, *Chem. Chem. Phys.,* 2006, **8**, 1057-1065. *(b)* F. Weigend and R. Ahlrichs, *Phys. Chem. Chem. Phys.,* 2005, **7**, 3297-3305.
3. *(a)* M. Cossi, N. Rega, G. Scalmani and V. Barone, *J. Comput. Chem.,* 2003, **24**, 669-681 (2003). *(b)* V. Barone and M. Cossi, *J. Phys. Chem. A.,* 1998, **102**, 1995-2001.
4. H. Roth, N. Romero and D. Nicewicz, *Synlett.,* 2016, **27**, 714-723.
5. E. G. Janzen and U. M. Oehler, *Tetrahedron Lett.,* 1983, **24**, 669-672.
6. S. Mukherjee, R. A. Garza-Sanchez, A. Tlahuext-Aca and F. Glorius, *Angew. Chem. Int. Ed.,* 2017, **56**, 14723-14726.
7. F. Xu, H. W. Deussen, B. Lopez, L. Lam and K. Li, *Eur. J. Biochem.,* 2001, **268**, 4169.
8. L. Buzzetti, G. E. M. Crisenza and P. Melchiorre, *Angew. Chem. Int. Ed.,* 2019, **58**, 3730-3747.
9. H. Liang, L. Wang, Y. Ji, H. Wang and B. Zhang, *Angew. Chem. Int. Ed.,* 2021, **60**, 1839-1844.
10. E. Kochany, B. H. Iwamura and J. Kochany, *Monatshefte für Chemie*, 1987, **118**, 1345-1348.
